# Microwave-Assisted One-Pot Telescoped Synthesis of 2‑Amino-1,3-thiazoles, Selenazoles, Imidazo[1,2‑*a*]pyridines, and Other Heterocycles from Alcohols

**DOI:** 10.1021/acs.joc.3c02903

**Published:** 2024-03-18

**Authors:** Pablo Macías-Benítez, Alfonso Sierra-Padilla, Francisco M. Guerra, F. Javier Moreno-Dorado

**Affiliations:** Departamento de Química Orgánica e Instituto de Biomoléculas, Facultad de Ciencias, 16727 Universidad de Cádiz, Polígono Río San Pedro s/n., 11510 Puerto Real, Cádiz, Spain

## Abstract

Primary and secondary alcohols have been converted into 2-amino-1,3-thiazoles under microwave irradiation, employing trichloroisocyanuric acid (TCCA) as a dual oxidant and chlorine source, TEMPO as a co-oxidant, and thiourea. Secondary alcohols underwent a single-stage, one-pot conversion process, while primary alcohols required a two-stage, one-pot procedure. Both transformations were completed within minutes (25–45 min). The versatility of this protocol extends to the synthesis of other heterocycles, including 1,3-selenazoles, 2-aminoimidazoles, imidazo­[1,2-*a*]­pyridines, quinoxalines, and hydrazino thiazoles by replacing thiourea with the appropriate surrogates.

## Graphical abstract

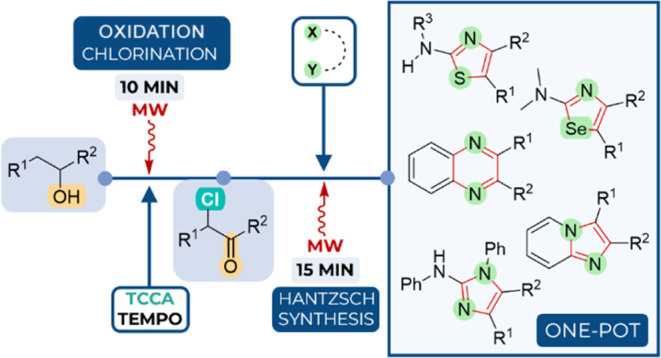

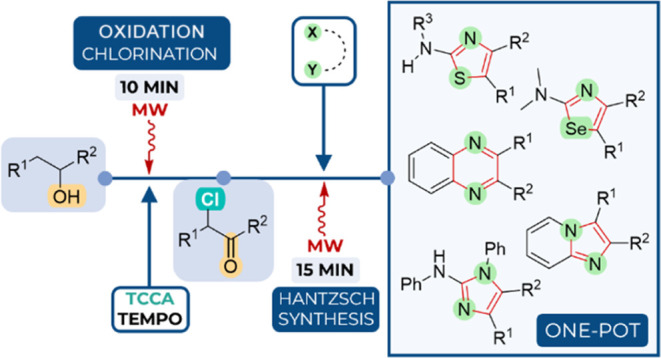

## Introduction

The development of novel and environmentally friendly methods for the synthesis of heterocycles with pharmacological activity is of paramount importance. Heterocycles, a privileged class of compounds, have a broad range of therapeutic potential. 1,3-Thiazole, a small five-membered aromatic heterocycle containing sulfur and nitrogen atoms, is particularly significant in medical applications due to its broad-spectrum pharmacological activities.^1,2^ 1,3-Thiazole serves as a core structural motif in essential drugs, including dasatinib, ritonavir, alpelisib, sulfathiazole, and the vital enzyme-cofactor vitamin B1 (Figure 1).^3^ Furthermore, the 2-aminothiazole functionality is a common feature in compounds with anticancer properties.^4^

**Figure 1:**
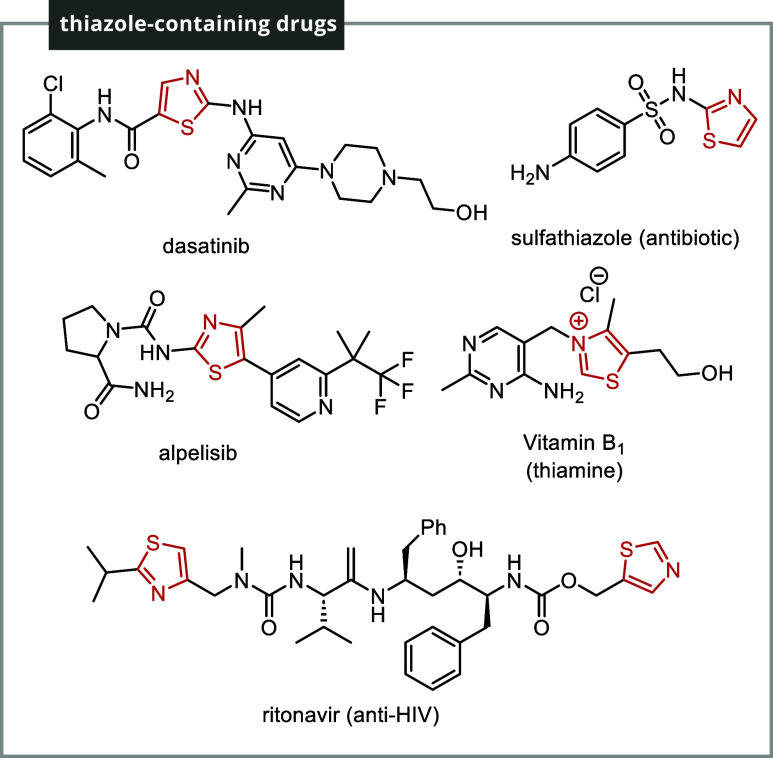
Thiazole-containing active molecules.

Despite extensive research devoted to the formation of 1,3-thiazole heterocycles, the available synthetic methodologies are relatively limited.^5–8^ Commonly employed procedures include the Robinson–Gabriel and the Cook–Heilbron syntheses (Scheme 1).^9^ An interesting alternative method developed by Zhang, Yu, and co-workers involves the use of vinyl azides and potassium thiocyanate.^10^

**Scheme 1:**
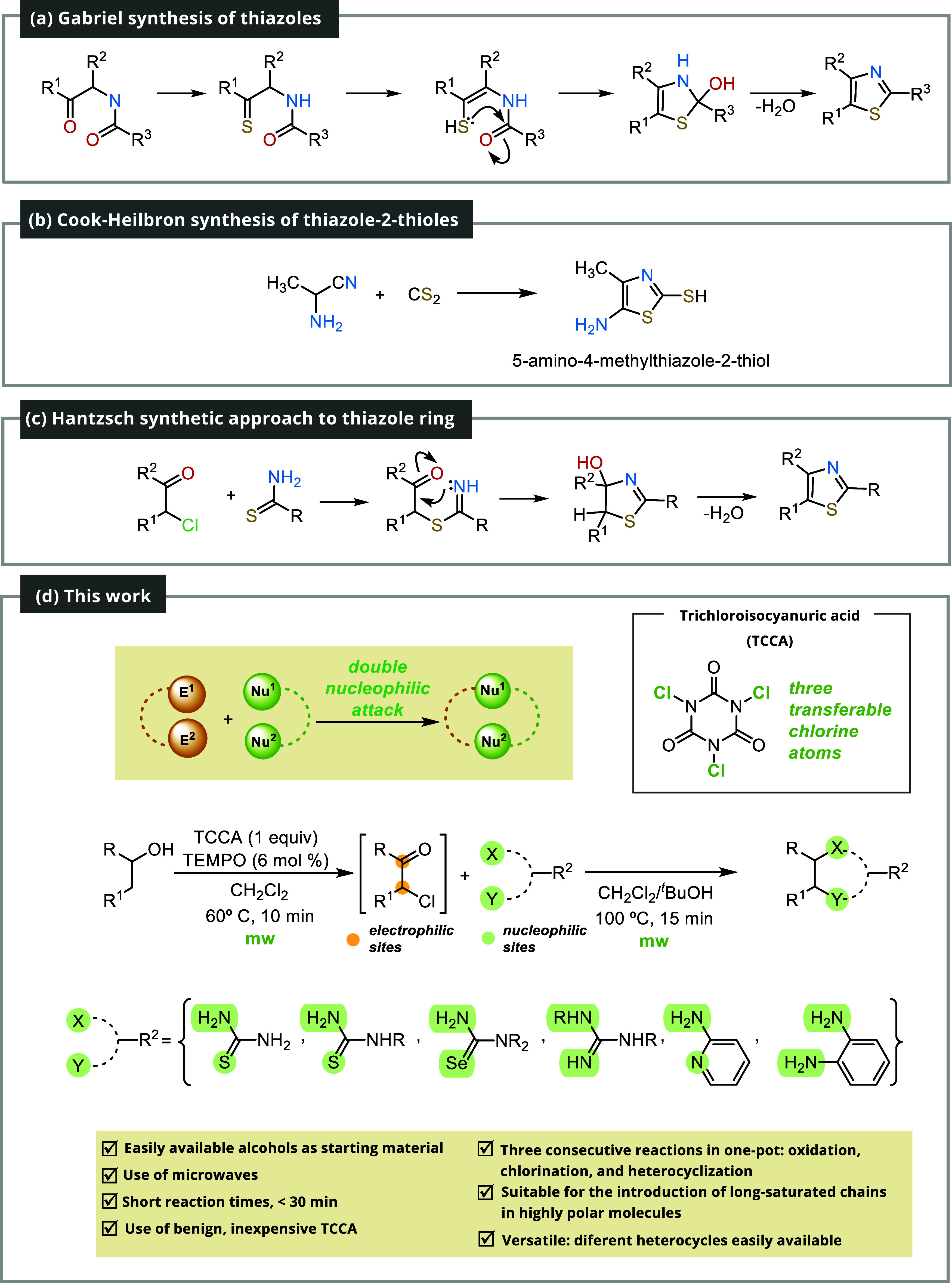
Synthetic Approaches to 2-Aminothiazoles and Other Heterocycles^a^

a (a) Ref 9. (b) Ref 9. (c) Ref 11.

However, the Hantzsch thiazole synthesis is recognized as the primary pathway to this unit.^11^ This venerable reaction, known for more than a century,^12^ has different variants, including ball-milling,^13^ microwave,^14^ and solvent-free^15^ versions. The standard form entails the reaction between a phenacyl halide and a thiourea, leading to the formation of a 1,3-thiazole.

The Hantzsch reaction represents a beautiful illustration of cooperative nucleophilic behavior, where nucleophilic sulfur and nitrogen atoms of thiourea engage with the α-haloaldehyde or α-haloketone, which, in turn, possess two electrophilic sites. This cooperative attack on both positions leads to the formation of a heterocyclic ring. Conceptually, it is possible to utilize any molecule endowed with two nucleophilic groups on a single carbon atom instead of thiourea, providing flexibility and opening up new possibilities for heterocyclic synthesis (Scheme 1d).

One constraint of the Hantzsch reaction is the narrow range of substrates often relying on benzophenones or their brominated derivatives, limiting the variety of substituents in the resulting thiazole. In addition, the low stability of α-haloaldehydes hinders their use as substrates,^16^ reducing the applicability of the reaction. To overcome these issues, the approach here considered involves generating the halocarbonyl compound *in situ* and reacting it with a dinucleophile already present in the medium. Employing microwave irradiation could enhance the reaction, enabling rapid heterocycle formation within minutes.

In 2014, Studer and co-workers described the conversion of primary and secondary alcohols into α-chloroaldehydes and α-chloroketones, respectively,^17^ using trichloroisocyanuric acid (TCCA),^18,19^ a widely used and affordable large-scale water disinfectant. This discovery allows for the consecutive oxidation and chlorination of a wide range of commercially available alcohols. The resulting halogenated compounds can be conveniently combined with different double nucleophiles, such as thiourea, selenourea, or guanidine, in a one-pot reaction procedure, yielding diverse functionalized heterocycles. This methodology holds the potential to produce novel compounds with biological activity, making it a compelling approach to heterocycle synthesis.

The current research aims to explore the application of Studer’s methodology using microwave irradiation to generate α-chloroaldehydes or α-chloroketones *in situ*, which are then captured by a double nucleophile to form the corresponding heterocycle. The entire pathway comprises three reaction steps (oxidation of the alcohol, chlorination of the resulting carbonyl compound, and cyclization) in a single one-pot process.

## Results and Discussion

The study commenced by selecting a benchmark reaction to identify optimal conditions. Following a series of preliminary experiments, octan-1-ol was selected as the test substrate. The resulting thiazole exhibited advantageous solubility properties, addressing a common issue associated with typical thiazoles. Various chlorine sources, oxidants, and solvent combinations were investigated during the study. Two different reaction setups were also explored: the first involved a one-pot approach. The second introduced thiourea in a separate stage after the formation of the corresponding α-chloroaldehyde, without isolating the intermediate. The results of these studies are summarized in Table 1.

**Table 1:**
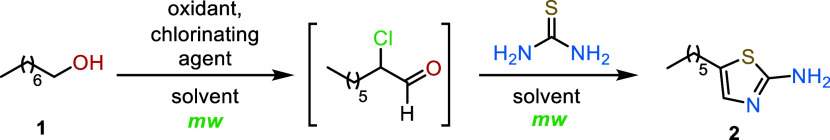
Screening of Conditions Using Octan-1-ol as a Starting Material^a^

entry	chlorinating agent (equiv)	oxidant (equiv)	solvent	yield (%)
One-pot Sequential Approach (Two Stages)				
1	TCCA (1)	TEMPO (1)	H_2_O	NR
2	TCCA (1)	TEMPO (1)	CH_2_Cl_2_/CH_2_Cl_2_/EtOH (1:2)^b^	40
3	TCCA (1)	NMO (1)	CH_2_Cl_2_/CH_2_Cl_2_/EtOH (1:2)^b^	30
4	TCCA (1)	TEMPO (1)	CH_2_Cl_2_/CH_2_Cl_2_/*t*-BuOH (1:2)^b^	90
5	TCCA (1)	TEMPO (0.25)	CH_2_Cl_2_/CH_2_Cl_2_/*t*-BuOH (1:2)^b^	89
**6**	**TCCA (1)**	**TEMPO (0.06)**	**CH**_**2**_ **Cl**_**2**_ **/CH**_**2**_ **Cl**_**2**_ **/** ***t*** **-BuOH** (1:2)^b^	**95**
7	TCCA (1)	TEMPO (0.06)	CH_3_CN	70
8	TsCl (1)	TEMPO (0.06)	CH_2_Cl_2_/CH_2_Cl_2_/*t*-BuOH (1:2)^b^	NR
9	Ca(OCl)_2_ (3)	TEMPO (0.06)	CH_2_Cl_2_/CH_2_Cl_2_/*t*-BuOH (1:2)^b^	NR
10	NaOCl (6)	TEMPO (0.06)	CH_2_Cl_2_/CH_2_Cl_2_/*t*-BuOH (1:2)^b^	NR
One-pot Concurrent Approach (One Single Stage)				
11	TCCA (1)	TEMPO (0.06)	CH_2_Cl_2_	10
12	TCCA (1)	TEMPO (0.06)	*t*-BuOH	31
13	TCCA (1)	TEMPO (0.06)	CH_3_CN	44
14	TCCA (1)	TEMPO (0.06)	CH_2_Cl_2_/*t*-BuOH (2:1)	54
15	TCCA (1)	TEMPO (0.06)	CH_2_Cl_2_/*t*-BuOH (1:2)	47
Conventional Heating				
16	TCCA (1)	TEMPO (0.06)	CH_2_Cl_2_/CH_2_Cl_2_/*t*-BuOH (1:2)	complex mixture

a Reaction conditions. ***Procedure A*** (entries 1–10), step 1: mw (60 °C, 10 min); step 2: thiourea addition, mw (100 °C, 15 min). ***Procedure B*** (entries 11–15): mw (60 °C, 10 min, then 100 °C, 15 min).

b In the second stage, EtOH or *t*-BuOH is added as a cosolvent over the CH_2_Cl_2_ from the first stage in the indicated ratio.

Initially, reactions were conducted in a one-pot, two-stage manner (entries 1–10). In the first stage, the alcohol was treated with the oxidant and the chlorinating agent, leading to the formation of the corresponding α-chloroaldehyde. The reactor was then opened, and thiourea was added in a second stage without further manipulation. In specific cases, a secondary solvent was included in the reaction mixture. Water failed to produce the desired thiazole (entry 1). When dichloromethane (DCM) was employed as a solvent for the initial stage, followed by the addition of thiourea and twice the volume of ethanol in the second stage, a moderate yield was obtained (entry 2). Using NMO as the oxidant (entry 3) gave slightly lower yields. The best result was obtained when the second stage of the reaction was carried out in a mixture of dichloromethane and *t*-butanol (entry 4).

Next, we examined the impact of varying the amount of TEMPO (entries 5 and 6). Reducing the amount to 0.06 equiv resulted in a remarkable 95% yield. Changing the solvent to acetonitrile (entry 7) under the same conditions significantly decreased the yield to 70%. Entries 8–10 present the results using different chlorine sources extracted from the literature, none of which displayed any visible reaction under the examined conditions.

Entries 11–15 present the outcome obtained when the reaction was conducted in a single stage with optimized amounts of TCCA and TEMPO. Despite obtaining thiazole **2**, the yields were only moderate. It is thus preferable to perform the reaction in two stages without isolating the α-chloroaldehyde intermediate and by adding the thiourea and *t*-butanol.

To conclude the optimization process, we evaluated the results under conventional thermal heating. Initial tests conducted using conventional heating proved unsatisfactory. In entry 16, a run employing the same optimal quantities established for irradiation is detailed, yielding a complex mixture with only traces of thiazole **2**.

After establishing the optimal reaction conditions, we found it interesting to introduce a lipophilic moiety to the thiazole by exploring alcohols with varying chain lengths. This modification aims to enhance its ability to modulate cellular entry through membranes.^20,21^ Additionally, we tested different symmetrical and asymmetrical thioureas, and the results of these investigations are summarized in Scheme 2.

**Scheme 2:**
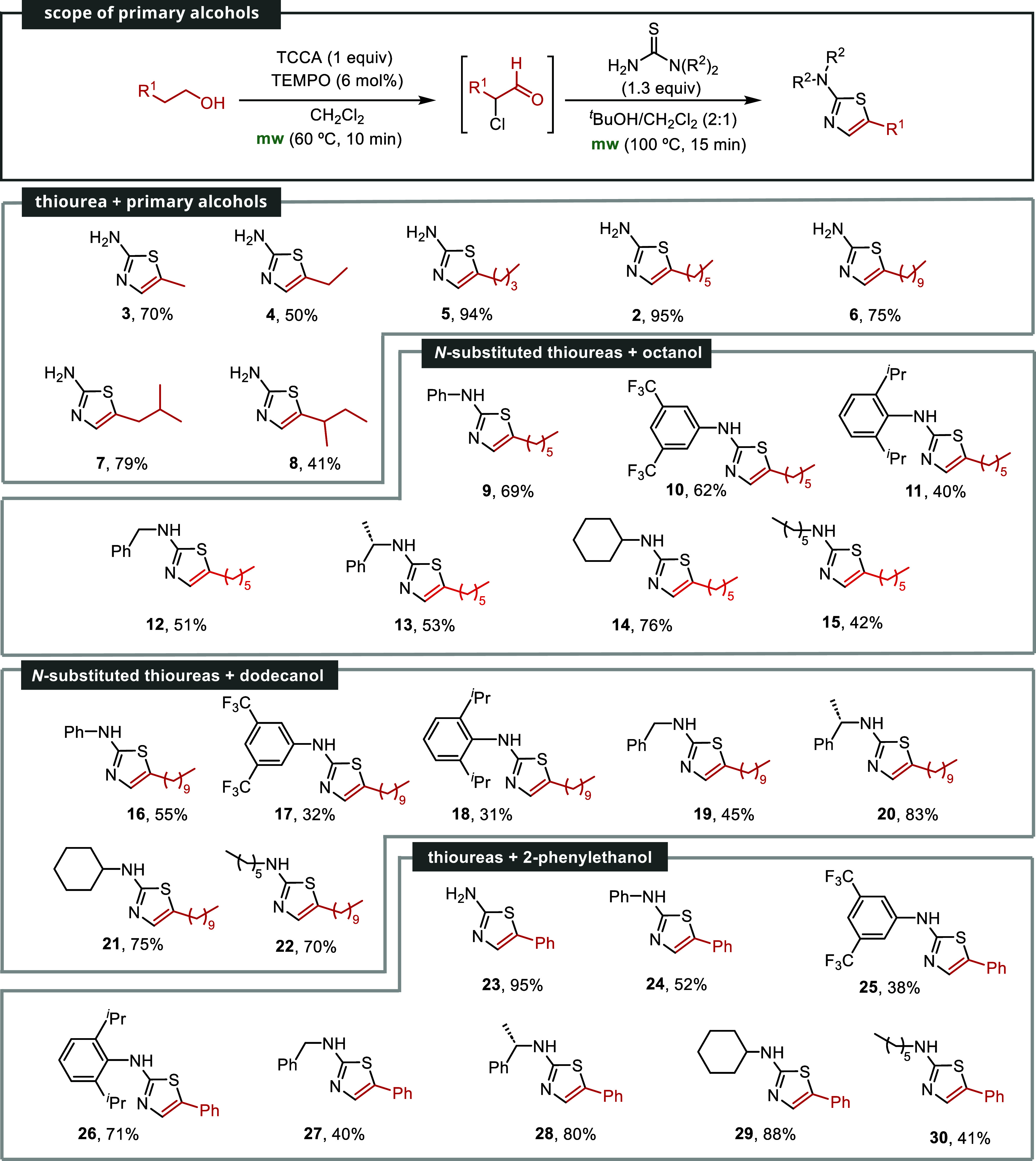
Scope of Primary Alcohols for 2-Aminothiazole Formation^a^

a Reaction conditions: step 1: alcohol (1 mmol, 1 equiv), TCCA (1 mmol, 1 equiv), TEMPO (6 mol %) in CH_2_Cl_2_ (4 mL), mw irradiation 10 min, 60 °C. Step 2: thiourea (1.3 mmol, 1.3 equiv) in *t*-BuOH (8 mL), mw irradiation, 15 min, 100 °C. Isolated yields.

Treatment of various simple alcohols with thiourea allowed for the synthesis of 2-amino-1,3-thiazoles with varying yields, and no discernible trend could be deduced. In instances such as the utilization of butanol and 3-methylpentan-1-ol, the yields were moderate (**4**, 50%; **8**, 41%). Other alcohols, including propan-1-ol (**3**, 70%), hexan-1-ol (**5**, 94%), dodecan-1-ol (**6**, 75%), and 4-methylpentan-1-ol (**7**, 79%), exhibited satisfactory yields.

When paired with octan-1-ol, various thioureas produced the corresponding thiazoles with moderate to good yields. Notably, cyclohexylthiourea **14** exhibited a 76% yield, phenyl thiourea **9** showed a 69% yield, and (3,5-bis­(trifluoromethyl)-phenyl)­thiourea **10** resulted in a 62% yield. Sterically hindered (2,6-diisopropylphenyl)­thiourea yielded 40% of thiazole **11**. Additionally, benzylthiourea and (*S*)-1-phenyl-ethylthiourea provided moderate yields of **12** and **13** (51 and 53%, respectively). Hexylthiourea yielded similarly, resulting in a 42% yield for thiazole **15**. Similar results were observed with dodecan-1-ol, with yields ranging from 31% for sterically hindered thiazole **18** to 83% for derivative **20** bearing a phenylethyl substituent.

We also explored the reactivity of 2-phenylethanol, which contains an aromatic ring. Similar trends to the previous alcohols were observed. Moderate yields were obtained with phenyl, benzyl, or hexylthiourea (**24**, 52%; **27**, 40%; **30**, 41%). Likewise, the presence of two CF_3_ groups also gave moderate yields (**25**, 38%). Thioureas with bulky *i*-propyl groups produced better results, yielding **26** with a 71% yield. Optically active 1-phenylethylthiourea gave **28** with an 80% yield, demonstrating good reactivity. The introduction of a cyclohexyl group led to excellent results, with thiazole **29** obtained in an 88% yield. Remarkably, when using thiourea itself as the partner, compound **23** was synthesized with an excellent yield of 95%.

The use of secondary alcohols posed some challenges; nonetheless, the corresponding thiazoles could be obtained in specific cases (Scheme 3). The reaction with this type of substrate was complicated by regioselectivity issues upon introducing the chlorine atom, resulting in a mixture of tough-to-separate regioisomers. In this type of substrate, tailored conditions were required for each individual substrate. A one-pot procedure, involving the addition of thiourea from the start, proved advantageous in most cases. For instance, under these conditions, 1-phenylethanol yielded thiazole **31** (a regioisomer of **23**) with an 89% yield. This yield highlights the versatility of the process in introducing different functionalities into the heterocycle depending on the alcohol used.

**Scheme 3:**
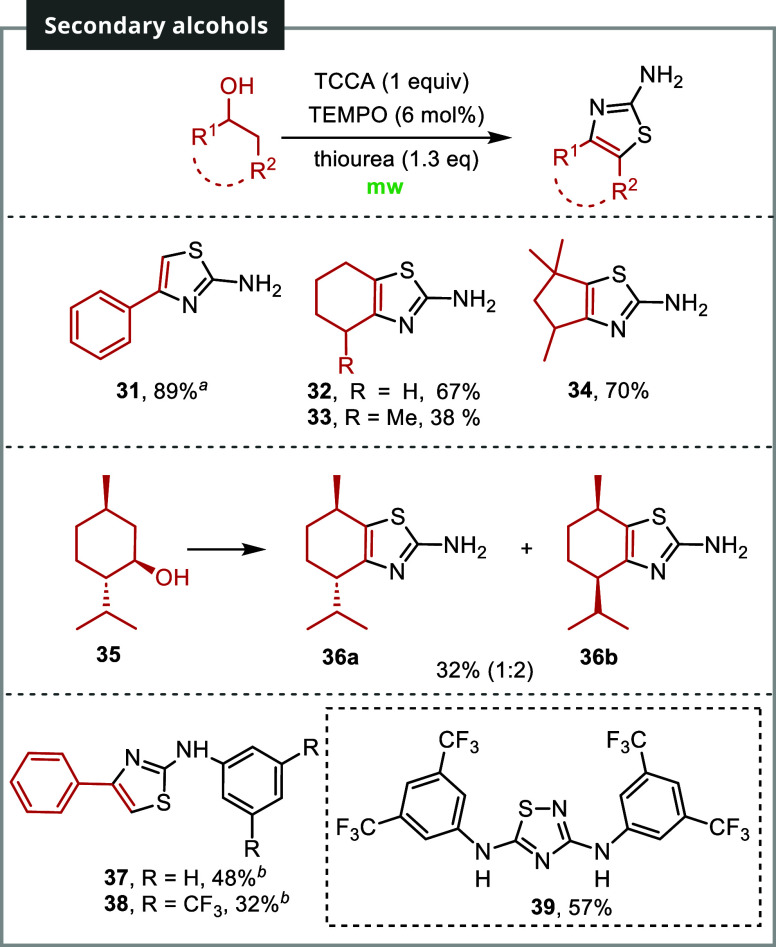
Substrate Scope of Secondary Alcohols^c^

a Two equiv. TCCA.
b Two-stage. Stage 1: alcohol (1 mmol, 1 equiv), TCCA (1 mmol, 1 equiv), TEMPO (6 mol %) in CH_2_Cl_2_ (4 mL), mw irradiation (15 min, 70 °C); Stage 2: thiourea (1.3 mmol, 1.3 equiv) in *t*-BuOH/CH_2_Cl_2_ (1:2, 12 mL), mw irradiation (15 min, 100 °C). Isolated yields.
c Reaction conditions: alcohol (1 mmol, 1 equiv), TCCA (1 mmol, 1 equiv), TEMPO (6 mol %), thiourea (1.3 mmol, 1.3 equiv) in *t*-BuOH/CH_2_Cl_2_ (1:2, 4 mL), mw irradiation (15 min, 70 °C, then 15 min, 100 °C).

Cyclohexanol produced thiazole **32** with a 67% yield, while the introduction of a methyl group resulted in a lower yield of 38% for compound **33**. Interestingly, a densely functionalized five-membered ring afforded the corresponding thiazole **34** in a respectable 70% yield. In the case of (−)-menthol **35**, thiazoles **36a** and **36b** were isolated in a 1:2 ratio with a yield of 32%. The primary isomer in this case is derived from the epimerization of the isopropyl group. This fact could be explained in terms of conformational stability of the resulting chlorinated intermediate, which would force the isopropyl group to adopt an equatorial position through an enolization process.

When substituted thioureas were used, the results varied. For instance, when phenyl-substituted thiourea was employed as a partner for 1-phenylethanol, thiazole **37** was isolated in a mere 7% yield. However, changing the procedure to a two-stage process increased the yield to 48%. It is worth mentioning that substituted thioureas may undergo dimerization processes under oxidative conditions. When employing 3,5-bis­(trifluoromethyl)-phenyl thiourea as the nucleophile in a one-pot procedure, the corresponding 1,2,4-thiadiazole **39** was produced in a 57% yield instead of the expected thiazole.^22^ Changing to the two-stage protocol, as in the case of primary alcohols, led to **38** in a 32% yield, with the corresponding thiadiazole **39** not being detected in this case.

The suitability of the process for the preparation of dimers was investigated at this stage (Scheme 4). Certain thiazole-containing dimers are known to exhibit intriguing pharmacological properties. In a notable example from 2009, Drozdowska and co-workers explored the potential of thiazole-containing dimers as DNA minor groove binders. The synthesized dimers showed properties similar to those of distamycin A and furamidin B. These compounds displayed significant *in vitro* cytotoxicity against two human breast cancer cells and exhibited activity against DNA topoisomerases I and II.^23^

**Scheme 4:**
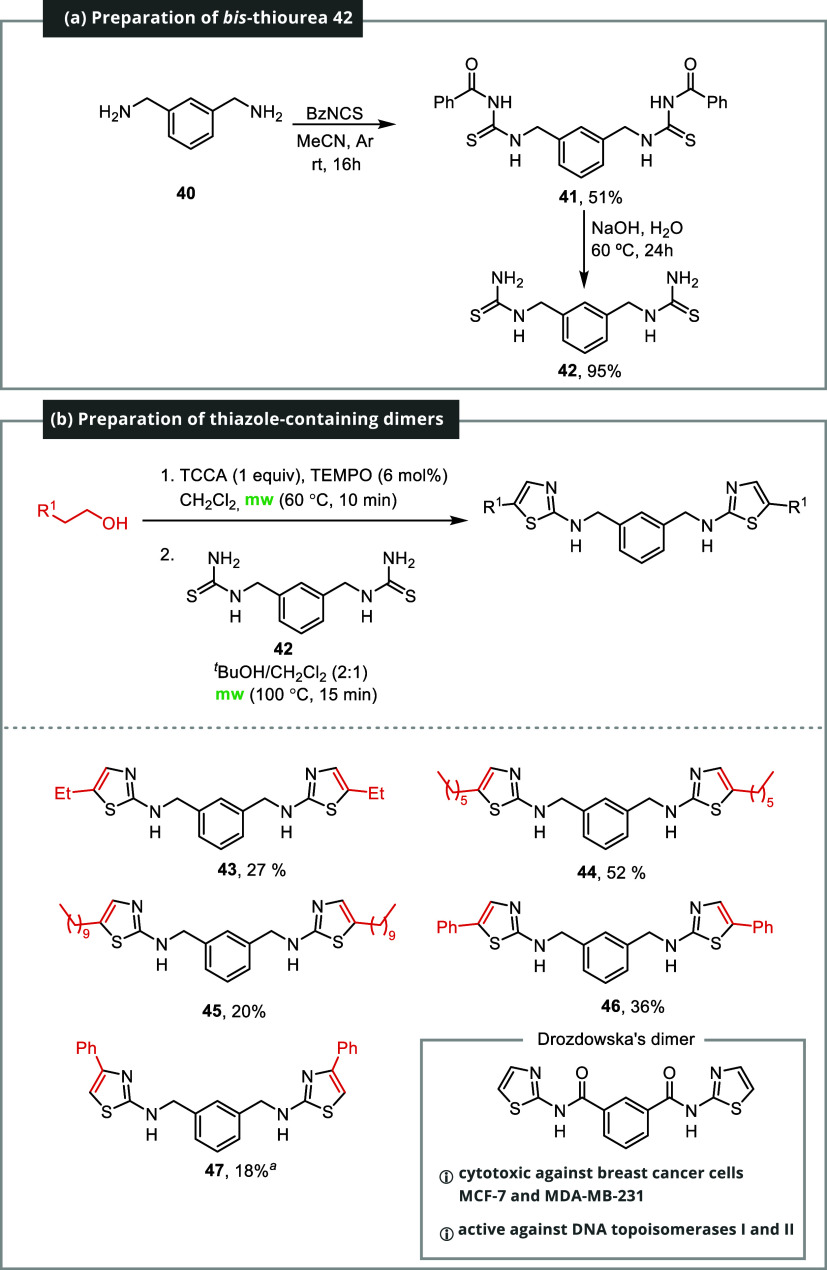
Synthesis of Thiazole-Containing Dimers^b^

a Step 1: alcohol (2.5 mmol), TCCA (2.5 mmol), TEMPO (6 mol %) in CH_2_Cl_2_ (5 mL), mw irradiation (15 min, 70 °C). Step 2: *bis*-thiourea **42** (0.5 mmol) in *t*-BuOH/CH_2_Cl_2_ (2:1, 15 mL), mw irradiation (30 min, 100 °C). Isolated yields.
b Reaction conditions: step 1: alcohol (2.5 mmol), TCCA (2.5 mmol), TEMPO (6 mol %) in CH_2_Cl_2_ (5 mL), mw irradiation (10 min, 60 °C). Step 2: bis-thiourea 42 (0.5 mmol) in *t*-BuOH/CH_2_Cl_2_ (2:1, 15 mL), mw irradiation (15 min, 100 °C).

The viability of utilizing the described technique for producing dimers similar to those described by Drozdowska was assessed by synthesizing the *bis*-thiourea **42**. *m*-Xylylenediamine **40** was reacted with benzoyl isothiocyanate, yielding symmetric *bis*-substituted thiourea **41** in a 51% yield. This thiourea was hydrolyzed with NaOH in water, resulting in *bis*-thiourea **42** with a 95% yield. *Bis*-thiourea **42** was then subjected to a reaction with various alcohols using the two-stage protocol mentioned earlier. Dimers **43**–**46** were formed with yields ranging from 20 to 52%. For dimer **47**, the method required adjustments including a longer reaction time and higher temperature.

Selenazoles are a class of heterocyclic compounds with potential medical applications. The 2-aminoselenazole ring structure is an important pharmacophore contributing to the therapeutic properties of its derivatives.^24^ Selenazofurin, exemplifying this structure, is *C*-glycosylated selenazole known for displaying both antitumor and antiviral activities (Scheme 5).^25^

**Scheme 5:**
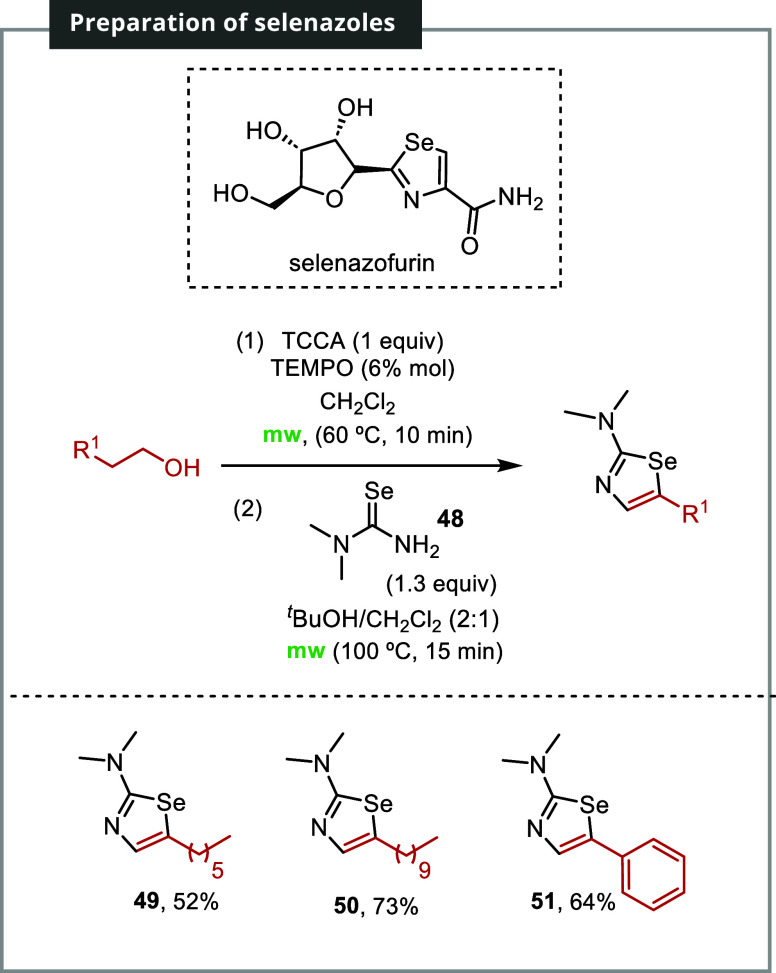
Synthesis of Selenazoles^a^

a Reaction conditions: step 1: alcohol (1 mmol, 1 equiv), TCCA (1 mmol, 1 equiv), TEMPO (6 mol %) in CH_2_Cl_2_ (4 mL), mw irradiation (10 min, 60 °C). Step 2: selenourea (1.3 mmol, 1.3 equiv) in *t*-BuOH (8 mL), mw irradiation (15 min, 100 °C). Isolated yields.

However, synthesizing selenazoles can be more challenging than their sulfur siblings due to the higher toxicity of organoselenium reagents and the lower stability of selenium heterocycles. Existing methods for selenazole synthesis often require multiple stages and/or harsh reaction conditions. To overcome these challenges, dimethyl selenourea **48** was explored as a dinucleophile using the described procedure. Scheme 5 demonstrates the successful and smooth progression of the reaction. Treatment of octan-1-ol with dimethyl selenourea **48** yielded selenazole **49** with a yield of 52%, whereas dodecan-1-ol and 1-phenylethanol resulted in selenazoles **50** and **51** with yields of 73 and 64%, respectively.

The investigation aimed to broaden the scope of heterocycles accessible through the described method, specifically focusing on the synthesis of imidazo­[1,2-*a*]­pyridines. The synthesis of this type of heterocycles is of great significance due to their diverse biological activities. Compounds of this type have demonstrated anti-inflammatory, anticancer, and antiviral properties. Imidazo­[1,2-*a*]­pyridines are essential components of various pharmaceutical drugs, including olprinone, zolmidine, and zolpidem.^26^

Traditional methods for synthesizing substituted imidazo­[1,2-*a*]­pyridines involve palladium-catalyzed couplings, requiring prefunctionalization of the ring as an iodide or bromide, which can be cumbersome.^27^ The one-pot, two-stage reaction method introduced in this work is straightforward, efficient, and particularly suitable for synthesizing imidazo­[1,2-*a*]­pyridines with long alkyl chains (Scheme 6). Imidazo­[1,2-*a*]­pyridine **53** was obtained with an 89% yield through the reaction of 1-phenylethanol and 2-aminopyridine. Imidazo­[1,2-*a*]­pyridines **54**–**56** were formed with yields of 58%, 51%, and 60%, respectively, by reacting 2-aminopyridine **52** with hexan-1-ol, octan-1-ol, and dodecan-1-ol, respectively.

**Scheme 6:**
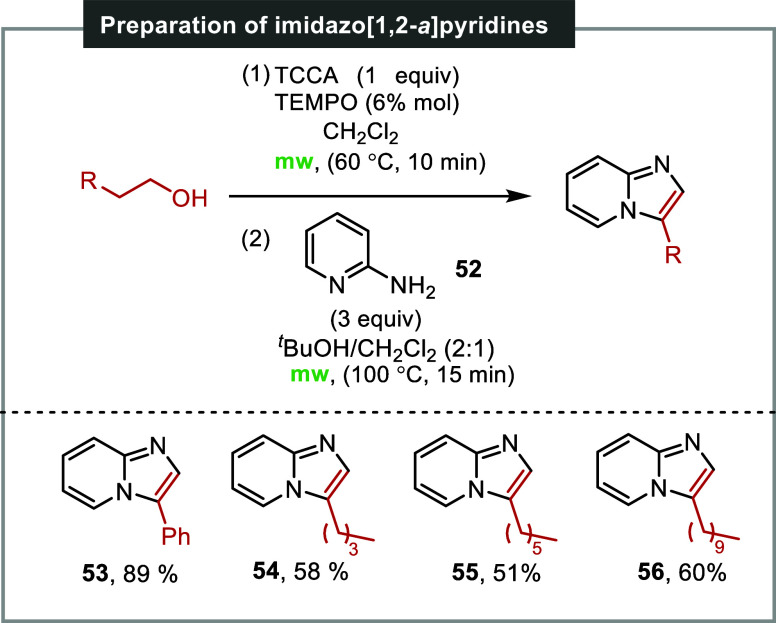
Synthesis of Imidizo­[1,2-*a*]­pyridines^a^

a Reaction conditions: step 1: alcohol (1 mmol, 1 equiv), TCCA (1 mmol, 1 equiv), TEMPO (6 mol %) in CH_2_Cl_2_ (4 mL), mw irradiation (10 min, 60 °C). Step 2:2-aminopyridine (3 mmol, 3 equiv) in *t*-BuOH/CH_2_Cl_2_ (2:1, 12 mL), mw irradiation (15 min, 100 °C). Isolated yields.

Finally, we examined the suitability of the approach by testing other types of nucleophiles: guanidines, o diaminopyridines, and hydrazine carbothiamides. These nucleophiles were found to be compatible with the reaction conditions (Scheme 7). Treatment of 1-phenylethanol and octan-1-ol with diphenylguanidine **57** produced 2-aminoimidazoles **58** and **59**, with yields of 50 and 36%, respectively. Similar outcomes were achieved using *o*-diaminobenzene **60**, resulting in the production of quinoxalines **61** and **62**, with yields of 62 and 31%, respectively. Hydrazineyl thiazoles **64** and **65** were obtained with yields of 5 and 20%, respectively, through the treatment of (*E*)-2-(1-phenylethylidene)­hydrazine-1-carbothioamide **63** with phenylethanol and octan-1-ol, respectively. These findings demonstrate the versatility of the methodology for the synthesis of diverse nitrogen heterocycles in a single stage.

**Scheme 7:**
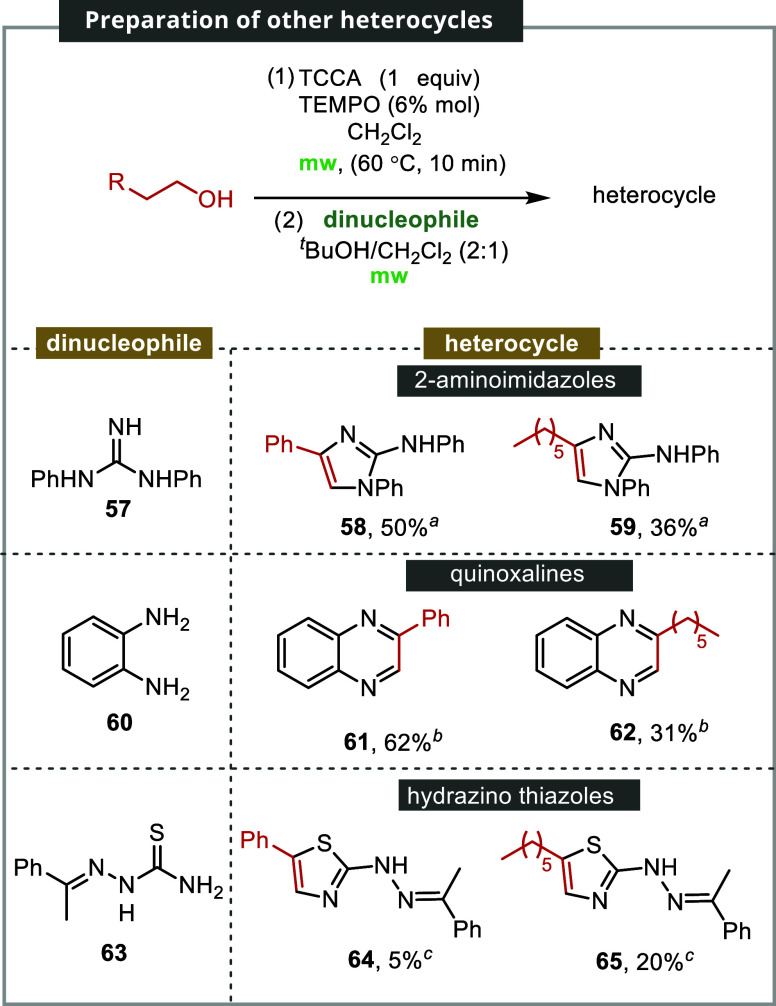
Synthesis of 2-Aminoimidazoles, Quinoxalines, and Hydrazino Thiazoles

a Reaction conditions: step 1: alcohol (1 mmol, 1 equiv), TCCA (1 mmol, 1 equiv), TEMPO (6 mol %) in CH_2_Cl_2_ (4 mL), mw irradiation (10 min, 60 °C). Step 2: diphenylguanidine **57** (3 mmol, 3 equiv) in *t*-BuOH/CH_2_Cl_2_ (2:1, 12 mL), mw irradiation (30 min, 100 °C).
b Step 1: alcohol (1 mmol, 1 equiv), TCCA (1 mmol, 1 equiv), TEMPO (6 mol %) in CH_2_Cl_2_ (4 mL), mw irradiation (10 min, 60 °C). Step 2: *o*-diaminobenzene **60** (6 mmol, 6 equiv) in *t*-BuOH/CH_2_Cl_2_ (2:1, 12 mL), mw irradiation (15 min, 100 °C).
c Step 1: alcohol (1 mmol, 1 equiv), TCCA (1 mmol, 1 equiv), TEMPO (6 mol %) in CH_2_Cl_2_ (4 mL), mw irradiation (10 min, 60 °C). Step 2: (*E*)-2-(1-phenylethyliden)­hydrazine-1-carbothioamide **63** (1.3 mmol, 1.3 equiv) in *t*-BuOH (8 mL), mw irradiation (15 min, 100 °C).

A proposed mechanism, based on previous work by Giacomelli and co-workers for the oxidation step^19^ and Li and co-workers for the chlorination step,^28^ is shown in Scheme 8. First, the TEMPO radical is oxidized by TCCA to form the cationic intermediate I. This intermediate incorporates alcohol II to give rise to intermediate III, which rearranges to yield the corresponding ketone V and the N–OH intermediate IV. The former is reoxidized by TCCA to regenerate intermediate I, initiating a new cycle. On the other hand, the enolic form, VI, of the generated ketone, attacks an electrophilic chlorine, potentially provided by TCCA via the protonated intermediate VIII. Eventually, the chlorinated intermediate VII undergoes a Hantzsch cyclization to produce the corresponding thiazole.

**Scheme 8:**
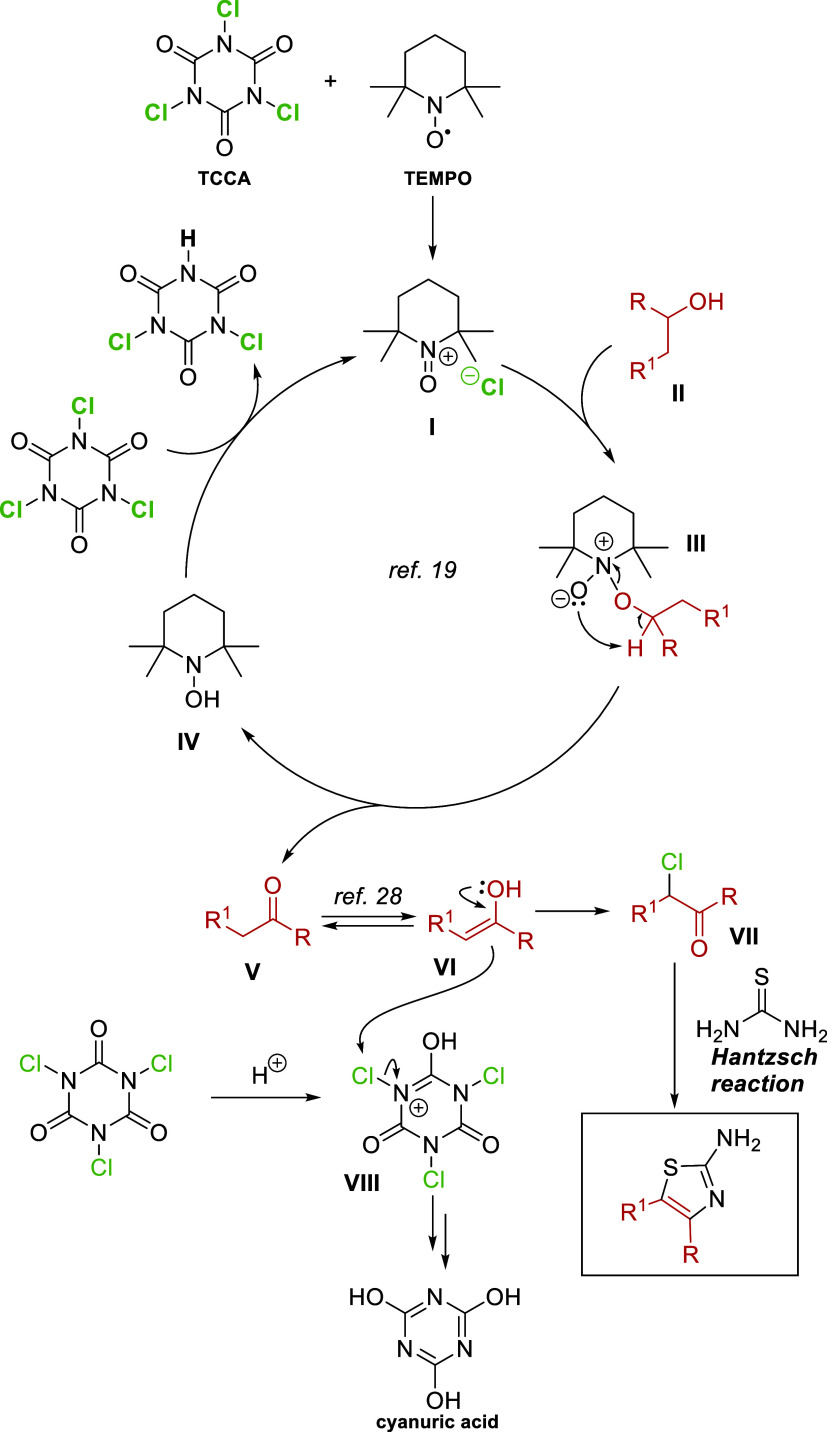
Proposed Mechanism for the Formation of 2-Aminothiazoles

## Conclusions

In conclusion, we have described a simple and efficient method for synthesizing a variety of heterocyclic compounds using alcohols as starting materials in a one-pot procedure. The availability of a vast variety of commercially available alcohols enables the preparation of a wide range of heterocycles. Additionally, the use of microwave irradiation results in shorter reaction times as opposed to conventional heating. Furthermore, chlorination and oxidation rely on trichloroisocyanuric acid (TCCA), a mild and inexpensive chlorinating agent, making the process cost-effective. The procedure seems especially appropriate for introducing long saturated chains into highly polar molecules, a challenge for conventional methods. The versatility is demonstrated by the ability to easily access a variety of heterocycles, highlighting its potential for the rapid and environmentally friendly synthesis of complex heterocycles.

## Experimental Section

### General Information

All reagents and solvents were purchased from commercial suppliers and used without further purification. Melting points were recorded in a Reichert Thermovar apparatus and are uncorrected. Reactions were monitored through TLC on commercial silica gel plates precoated with silica gel F_254_. Visualization was performed by fluorescence quenching and the Dragendorff reagent. Column chromatography was performed employing 230–400 mesh silica gel. HPLC purification was carried out in a Merck-Hitachi L6270 chromatograph equipped with a silica gel column (LiChrosorb Si 60, 10 μm particle size).

^1^H NMR, ^13^C NMR, and ^19^F-NMR spectra were recorded on a Bruker Avance Neo 400 or Bruker Avance Neo 500 instruments and calibrated using residual undeuterated solvent as an internal reference for ^1^H NMR and to the central peak of CDCl_3_ for ^13^C NMR. ^19^F-NMR spectra were referenced from CFCl_3_. Structural assignments were made with additional information from gCOSY, gHSQC, and NOESY experiments.

IR spectra of the organic compounds were recorded in a PerkinElmer Spectrum BX spectrophotometer in attenuated total reflection mode (ATR).

High-resolution mass spectra were recorded on a HRMS SYNAPT G2 (Waters) with a QTOF analyzer or in a XEVO QTOF for ESI ionization.

Microwave-promoted reactions were carried out in a SynthWave MA167 reactor pressurized with nitrogen limited to 45 bar of maximum pressure. The reactions were run on 50 mL glass vials immersed in 200 mL of water as a charge solvent with magnetic stirring.

### General One-Pot Sequential Approach (Two-Stage) Procedure for the Preparation of Heterocycles

The alcohol (1 mmol) was added to a mixture of TCCA (232 mg, 1 mmol, 1 equiv) and TEMPO (9 mg, 0.06 mmol) in CH_2_Cl_2_ (4 mL). The mixture was subjected to stirring under microwave irradiation (for the corresponding microwave irradiation time and temperature, see footnotes in Schemes 2–7 and the Supporting Information). Upon the formation of the α-chlorocarbonyl compound, 8 mL of *t*-butanol and the nucleophile (1.3 equiv. for thiourea, dimethyl selenourea **48**, and (*E*)-2-(1-phenylethyliden)-hydrazine-1-carbothioamide **63**; 3 equiv. for 2-amino-pyridine **52** and diphenylguanidine **57**; 6 equiv. for *o*-aminobenzene **60**) were added. Additional stirring was performed using the optimal microwave method. The solvent was removed by rotary evaporation. The reaction was treated with a saturated aqueous K_2_CO_3_ solution (3 × 20 mL) and extracted with CH_2_Cl_2_ (3 × 20 mL). The organic layer was desiccated over anhydrous Na_2_SO_4_ and evaporated under vacuum. The crude mixture was purified by column chromatography using silica gel, resulting in the corresponding heterocycle.

### General One-Pot Concurrent Approach (One Single Stage) for the Preparation of Thiazoles

The alcohol (1 mmol) and the thiourea (1.3 equiv) were sequentially added to a mixture of TCCA (232 mg, 1 mmol, 1 equiv) and TEMPO (9 mg, 0.06 mmol) in 4 mL of *t*-BuOH/CH_2_Cl_2_ (1:2). The resulting mixture was stirred under microwave irradiation (see footnote in Scheme 3). The solvent was removed under vacuum. The reaction was treated with a saturated aqueous K_2_CO_3_ solution (20 mL) and extracted with CH_2_Cl_2_ (3 × 20 mL). The organic layer was desiccated over anhydrous Na_2_SO_4_ and evaporated under vacuum. The crude mixture was purified by column chromatography using silica gel to obtain the corresponding thiazole.

**Caution!** Addition of *N*-substituted thioureas in the second stage must be performed at 0 °C. Adding thiourea at room temperature causes an increase in the solvent temperature, leading to a drop in yield.

### General Procedure for the Preparation of Thiazole-Containing Dimers

The alcohol (2.5 mmol) was added to a mixture of TCCA (2.5 mmol, 1 equiv) and TEMPO (0.15 mmol, 0.06 equiv) in CH_2_Cl_2_ (5 mL), and the mixture was stirred under microwave irradiation. After the first irradiation stage, *t*-BuOH (10 mL) and *bis*-thiourea **42** (0.5 mmol, 0.2 equiv) were added. The mixture was stirred under microwave according to the corresponding irradiation method. Solvent removal was carried out by rotary evaporation. The reaction was treated with a saturated aqueous K_2_CO_3_ solution (20 mL) and extracted with CH_2_Cl_2_ (3 × 20 mL). The organic layer was dried over anhydrous Na_2_SO_4_ and evaporated under vacuum. The crude mixture was purified by column chromatography using silica gel to obtain the corresponding dimer.

### Synthesis of Dibenzamide **41**

A 100 mL round-bottom flask was charged with *m*-xylylenediamine (1.238 g, 9.1 mmol) in 20 mL of CH_3_CN under an argon atmosphere. Benzoyl isothiocyanate (3.297 g, 20.1 mmol, 2.2 equiv) dissolved in 10 mL of CH_3_CN was added dropwise, resulting in the formation of a white precipitate. The reaction mixture was stirred overnight at room temperature. The formed solid was collected by filtration and washed with cold CH_3_CN. This process yielded compound **41** (2.155 g, 51% yield).

### Synthesis of 1,1′-(1,3-Phenylenebis­(methylene))-bis­(thiourea) **42**

A 250 mL round-bottom flask was charged with compound **41** (2.155 g, 4.66 mmol), distilled water (60 mL), and NaOH (1.242 g, 31.1 mmol, 6.7 equiv). The mixture was stirred at 60 °C for 24 h. After completion of the reaction, the white solid was collected by filtration. The collected solid furnished the *bis*-thiourea **42** (1.126 g, 95% yield)
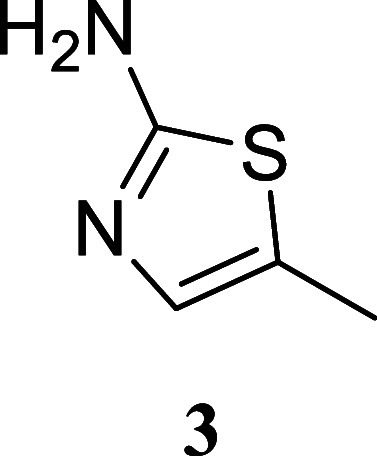
.

#### 5-Methylthiazol-2-amine (**3**)^29^

It was obtained as a brown oil (79.9 mg, 70%). Column chromatography eluent, EtOAc/hexane gradient from 1:9 to 3:2. *R*_f_ 0.21 (3:2 v/v EtOAc/hexanes). ^1^H NMR (500 MHz, CDCl_3_) δ 6.71 (q, *J* = 1.3 Hz, 1H), 4.69 (br s, 2H), 2.29 (d, *J* = 1.3 Hz, 3H). ^13^C­{^1^H} NMR (125 MHz, CDCl_3_) δ 166.5, 135.2, 123.5, 12.0. HRMS (ESI) *m*/*z*: [M + H]^+^ calcd for C_4_H_7_N_2_S 115.0330; found 115.0330. IR (ATR, cm^–1^) 3424, 3264, 3111, 2922, 1694, 1614, 1560, 1513, 1432, 1381, 1333, 1047, 770, 522, 506
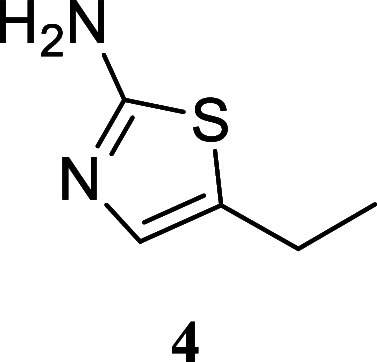
.

#### 5-Ethylthiazol-2-amine (**4**)^30^

It was obtained as a yellow oil (64.1 mg, 50%). Column chromatography eluent, EtOAc/hexane gradient from 1:9 to 3:2. *R*_f_ 0.10 (2:3 v/v EtOAc/hexanes). ^1^H NMR (400 MHz, CDCl_3_) δ 6.73 (t, *J* = 1.2 Hz, 1H), 4.76 (br s, 2H), 2.66 (qd, *J* = 7.5, 1.2 Hz, 2H), 1.23 (t, *J* = 7.5 Hz, 3H). ^13^C­{^1^H} NMR (100 MHz, CDCl_3_) δ 166.1, 133.7, 131.4, 20.6, 15.7. HRMS (ESI) *m*/*z*: [M + H]^+^ calcd for C_5_H_9_N_2_S 129.0486; found 129.0487. IR (ATR, cm^–1^) 3422, 3264, 3125, 2961, 2927, 1609, 1560, 1515, 1314, 1208, 1060, 1034, 846, 563, 504
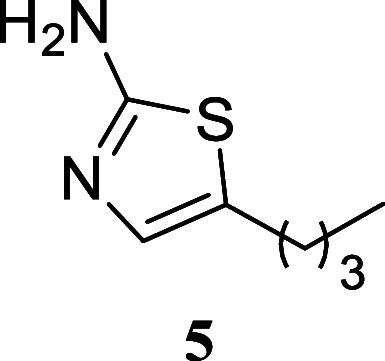
.

#### 5-Butylthiazol-2-amine (**5**)^30^

It was obtained as a brown oil (146.9 mg, 94%). Column chromatography eluent, EtOAc/hexane gradient from 1:9 to 3:2. *R*_f_ 0.33 (3:2 v/v EtOAc/hexanes). ^1^H NMR (500 MHz, CDCl_3_) δ 6.72 (t, *J* = 1.1 Hz, 1H), 4.83 (br s, 2H), 2.62 (td, *J* = 7.4, 1.1 Hz, 2H), 1.56 (tt, *J* = 7.4, 7.4 Hz, 2H), 1.36 (tq, *J* = 7.4, 7.4 Hz, 2H), 0.91 (t, *J* = 7.4 Hz, 3H). ^13^C­{^1^H} NMR (125 MHz, CDCl_3_) δ 166.3, 134.3, 129.8, 33.4, 26.7, 22.0, 13.7. HRMS (ESI) *m*/*z*: [M + H]^+^ calcd for C_7_H_13_N_2_S 157.0799; found 157.0797. IR (ATR, cm^–1^) 3298, 3181, 2957, 2930, 2871, 2859, 1691, 1618, 1519, 1464
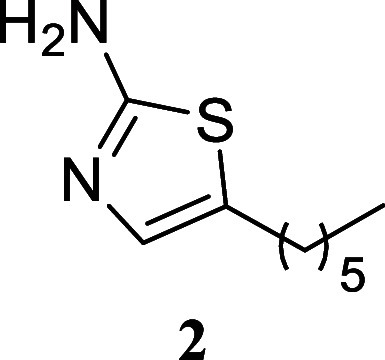
.

#### 5-Hexylthiazol-2-amine (**2**)

It was obtained as a brown oil (175.1 mg, 95%). Column chromatography eluent, EtOAc/hexane gradient from 1:9 to 3:2. *R*_f_ 0.38 (3:2 v/v EtOAc/hexanes). ^1^H NMR (500 MHz, CDCl_3_) δ 6.72 (t, *J* = 1.1 Hz, 1H), 4.73 (br s, 2H), 2.62 (td, *J* = 7.7, 1.1 Hz, 2H), 1.57 (tt, *J* = 7.7, 7.7 Hz, 2H), 1.37–1.23 (m, 6H), 0.88 (t, *J* = 6.9 Hz, 3H). ^13^C­{^1^H} NMR (125 MHz, CDCl_3_) δ 166.4, 133.7, 129.7, 31.5, 31.2, 28.6, 27.1, 22.5, 14.0. HRMS (ESI) *m*/*z*: [M + H]^+^ calcd for C_9_H_17_N_2_S 185.1112; found 185.1116. IR (ATR, cm^–1^) 3423, 2956, 2919, 2849, 1609, 1516, 846
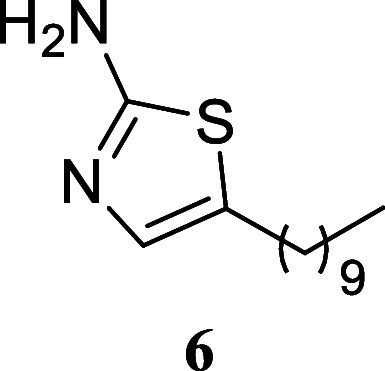
.

#### 5-Decylthiazol-2-amine (**6**)^31^

It was obtained as an amorphous solid (180.3 mg, 75%). Column chromatography eluent, EtOAc/hexane gradient from 1:9 to 3:2. *R*_f_ 0.29 (2:3 v/v EtOAc/hexanes). ^1^H NMR (400 MHz, CDCl_3_) δ 6.71 (s, 1H), 4.92 (br s, 2H), 2.61 (td, *J* = 7.5, 0.8 Hz, 2H), 1.56 (tt, *J* = 7.5, 7.5 Hz, 2H), 1.37–1.20 (m, 14H), 0.87 (t, *J* = 6.6 Hz, 3H). ^13^C­{^1^H} NMR (100 MHz, CDCl_3_) δ 166.4, 134.2, 129.7, 31.9, 31.3, 29.6, 29.5, 29.3, 28.9, 27.1, 22.7, 14.1. HRMS (ESI) *m*/*z*: [M + H]^+^ calcd for C_13_H_25_N_2_S 241.1738; found 241.1746. IR (ATR, cm^–1^) 3416, 3265, 3154, 2953, 2918, 2848, 1608, 1561, 1516, 1463, 1376, 1360, 1211, 848, 525
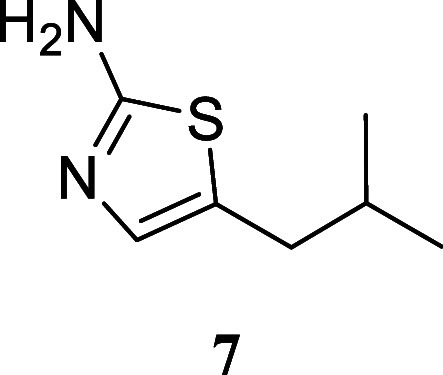
.

#### 5-Isobutylthiazol-2-amine (**7**)

It was obtained as a yellow oil (123.4 mg, 79%). Column chromatography eluent, EtOAc/hexane gradient from 1:9 to 3:2. *R*_f_ 0.16 (2:3 v/v EtOAc/hexanes). ^1^H NMR (400 MHz, CDCl_3_) δ 6.71 (t, *J* = 0.9 Hz, 1H), 4.89 (br s, 2H), 2.48 (dd, *J* = 7.0, 1.0 Hz, 2H), 1.56 (hept, *J* = 6.7, 6.7 Hz, 1H), 0.92 (d, *J* = 6.7 Hz, 6H). ^13^C­{^1^H} NMR (125 MHz, CDCl_3_) δ 166.6, 135.2, 128.2, 36.2, 30.1, 22.1. HRMS (ESI) *m*/*z*: [M + H]^+^ calcd for C_7_H_13_N_2_S 157.0799; found 157.0804. IR (ATR, cm^–1^) 3272, 3152, 2954, 2926, 2868, 2835, 1606, 1555, 1516, 1464, 1321, 1205, 1050, 1846, 519
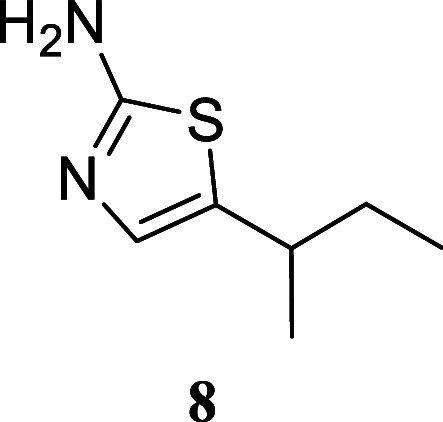
.

#### 5-(*sec*-Butyl)­thiazol-2-amine (**8**)

It was obtained as a yellow oil (64.1 mg, 41%). Column chromatography eluent, EtOAc/hexane gradient from 1:9 to 3:2. *R*_f_ 0.21 (2:3 v/v EtOAc/hexanes).^1^H NMR (400 MHz, CDCl_3_) δ 6.72 (d, *J* = 0.8 Hz, 1H), 4.94 (br s, 2H), 2.74 (tq, *J* = 6.8, 6.8 Hz, 1H), 1.53 (qdd, *J* = 7.4, 7.4, 1.7 Hz, 2H), 1.23 (d, *J* = 6.8 Hz, 3H), 0.87 (t, *J* = 7.4 Hz, 3H). ^13^C­{^1^H} NMR (125 MHz, CDCl_3_) δ 166.3, 135.7, 133.1, 34.7, 31.6, 22.2, 11.7. HRMS (ESI) *m*/*z*: [M + H]^+^ calcd for C_7_H_13_N_2_S 157.0799; found 157.0790. IR (ATR, cm^–1^) 3295, 3173, 2961, 2925, 2873, 1614, 1514, 1454, 1376, 1308, 1055, 837, 580
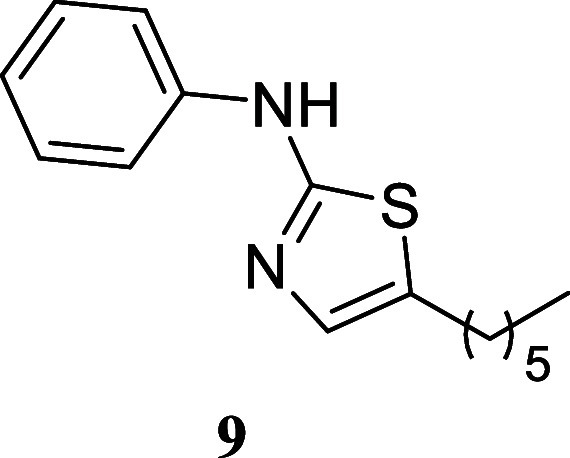
.

#### 5-Hexyl-*N*-phenylthiazol-2-amine (**9**)

It was obtained as a white crystal (179.7 mg, 69%), mp 57.9–59.7 °C. Column chromatography eluent, EtOAc/hexane gradient from 1:9 to 3:2. *R*_f_ 0.57 (1:4 v/v EtOAc/hexanes).^1^H NMR (500 MHz, CDCl_3_) δ 7.89 (br s, 1H), 7.36–7.29 (m, 4H), 7.06–7.01 (m, 1H), 6.94 (t, *J* = 1.1 Hz, 1H), 2.69 (td, *J* = 7.5, 1.1 Hz, 2H), 1.61 (tt, *J* = 7.5, 7.5 Hz, 2H), 1.40–1.26 (m, 6H), 0.88 (t, *J* = 6.9 Hz, 3H). ^13^C­{^1^H} NMR (125 MHz, CDCl_3_) δ 163.7, 140.7, 134.3, 129.4, 128.4, 122.5, 117.8, 31.5, 31.3, 28.6, 27.0, 22.5, 14.1. HRMS (ESI) *m*/*z*: [M + H]^+^ calcd for C_15_H_21_N_2_S 261.1425; found 261.1443. IR (ATR, cm^–1^) 3241, 2952, 2931, 2855, 1614, 1574, 1500, 1464, 1429, 1371, 1345, 1322, 1161, 833, 744, 687
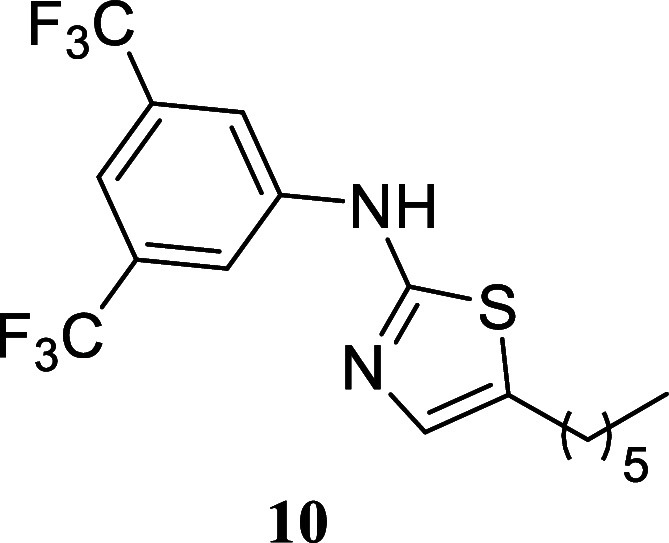
.

#### *N*-(3,5-Bis­(trifluoromethyl)­phenyl)-5-hexylthiazol-2-amine (**10**)

It was obtained as a yellow crystal (245.6 mg, 62%), mp 74.6–75.9 °C. Column chromatography eluent, EtOAc/hexane gradient from 1:9 to 3:2. *R*_f_ 0.70 (1:4 v/v EtOAc/hexanes). ^1^H NMR (500 MHz, CDCl_3_) δ 8.64 (br s, 1H), 7.85 (s, 2H), 7.46 (s, 1H), 7.02 (s, 1H), 2.74 (td, *J* = 7.5, 0.9 Hz, 2H), 1.64 (tt, *J* = 7.5, 7.5 Hz, 2H), 1.45–1.24 (m, 6H), 0.89 (t, *J* = 7.0 Hz, 3H). ^13^C­{^1^H} NMR (125 MHz, CDCl_3_) δ 161.4, 142.1, 134.5, 132.6 (q, ^2^ *J*_C–F_ = 33.3 Hz), 130.7, 123.20 (q, ^1^ *J*_C–F_ = 272.8 Hz), 116.5–116.3 (m), 115.0–114.8 (m), 31.5, 31.2, 28.6, 26.9, 22.5, 14.0. ^19^F­{^1^H} NMR (376 MHz, CDCl_3_) δ – 63.10. HRMS (ESI) *m*/*z*: [M + H]^+^ calcd for C_17_H_19_N_2_F_6_S 397.1173; found 397.1187. IR (ATR, cm^–1^) 3236, 3084, 2931, 2858, 1624, 1595, 1471, 1383, 1275, 1166, 1130, 1120, 1110, 998, 967, 872, 847, 818, 772, 684
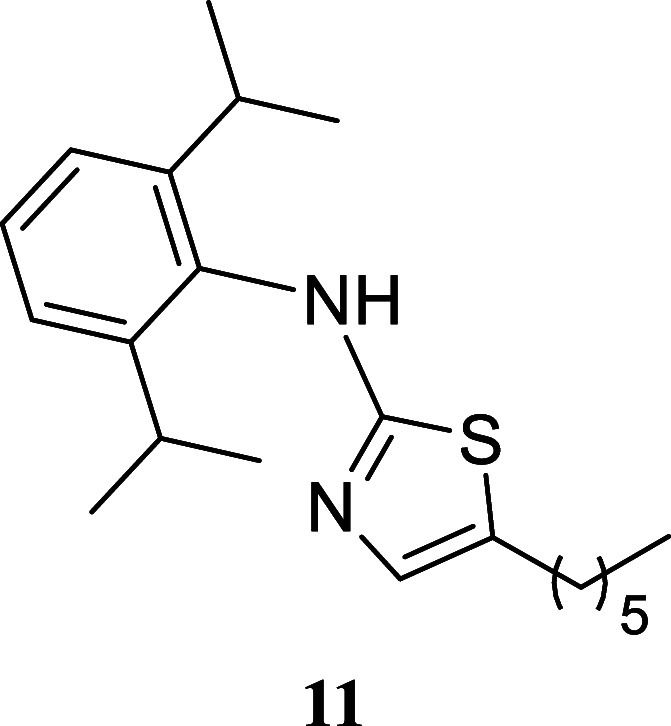
.

#### *N*-(2,6-Diisopropylphenyl)-5-hexylthiazol-2-amine (**11**)

It was obtained as a yellow oil (137.7 mg, 40%). Column chromatography eluent, EtOAc/hexane gradient from 1:9 to 3:2. *R*_f_ 0.51 (1:4 v/v EtOAc/hexanes). ^1^H NMR (500 MHz, CDCl_3_) δ 7.35 (br t, *J* = 7.7 Hz, 1H), 7.23 (d, *J* = 7.7 Hz, 2H), 6.79 (t, *J* = 1.0 Hz, 1H), 3.28 (hept, *J* = 6.9 Hz, 2H), 2.56 (td, *J* = 7.5, 1.0 Hz, 2H), 1.50 (tt, *J* = 7.5, 7.5 Hz, 2H), 1.33–1.20 (m, 6H), 1.18 (d, *J* = 6.9 Hz, 12H), 0.85 (t, *J* = 6.9 Hz, 3H). ^13^C­{^1^H} NMR (125 MHz, CDCl_3_) δ 170.3, 147.7, 134.7, 134.3, 128.8, 127.9, 124.2, 31.5, 31.1, 28.5, 28.3, 27.1, 23.9, 22.5, 14.0. HRMS (ESI) *m*/*z*: [M + H]^+^ calcd for C_21_H_33_N_2_S 345.2364; found 345.2382. IR (ATR, cm^–1^) 3163, 3064, 2960, 2927, 2867, 1551, 1460, 1433, 1383, 1362, 1330, 1255, 1152, 1058, 812, 748
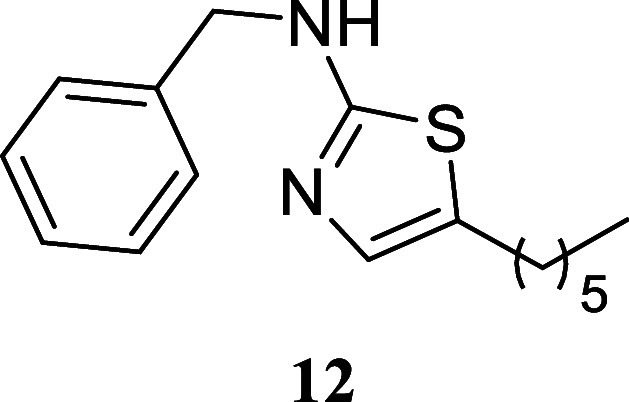
.

#### *N*-Benzyl-5-hexylthiazol-2-amine (**12**)

It was obtained as an amorphous solid (139.8 mg, 51%). Column chromatography eluent, EtOAc/hexane gradient from 1:9 to 3:2. *R*_f_ 0.35 (1:4 v/v EtOAc/hexanes). ^1^H NMR (400 MHz, CDCl_3_) δ 7.39–7.27 (m, 5H), 6.74 (s, 1H), 5.53 (br s, 1H), 4.44 (s, 2H), 2.61 (t, *J* = 7.5 Hz, 2H), 1.56 (tt, *J* = 7.4, 7.4 Hz, 2H), 1.39–1.22 (m, 6H), 0.88 (t, *J* = 6.8 Hz, 3H). ^13^C­{^1^H} NMR (100 MHz, CDCl_3_) δ 168.5, 137.9, 134.5, 128.9, 128.7, 127.6, 127.6, 49.7, 31.5, 31.2, 28.6, 27.1, 22.5, 14.1. HRMS (ESI) *m*/*z*: [M + H]^+^ calcd for C_16_H_23_N_2_S 275.1582; found 275.1600. IR (ATR, cm^–1^) 3210, 3030, 2928, 2855, 1694, 1551, 1496, 1454, 1351, 1150, 731, 698
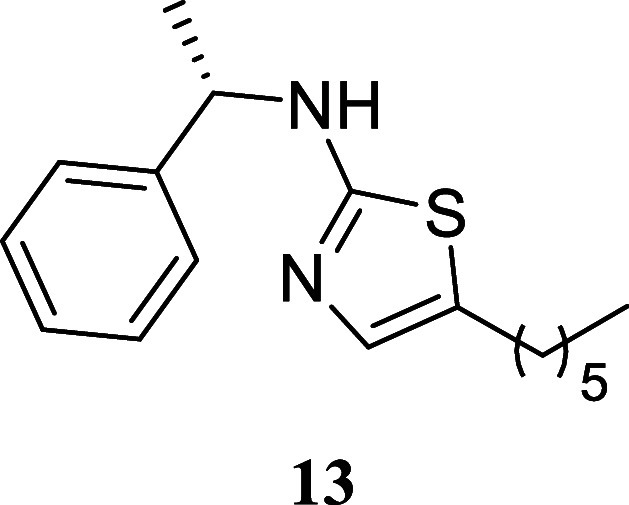
.

#### (*S*)-5-Hexyl-*N*-(1-phenylethyl)­thiazol-2-amine (**13**)

It was obtained as a white crystal (152.7 mg, 53%), mp 45.9–47.8 °C. α_[D]_^20^ – 66.0 (*c* 0.10, CHCl_3_). Column chromatography eluent, EtOAc/hexane gradient from 1:9 to 3:2. *R*_f_ 0.35 (1:4 v/v EtOAc/hexanes). ^1^H NMR (400 MHz, CDCl_3_) δ 7.40–7.31 (m, 4H), 7.29–7.24 (m, 1H), 6.72 (s, 1H), 6.03 (br s, 1H), 4.55 (q, *J* = 6.5 Hz, 1H), 2.55 (t, *J* = 7.4 Hz, 2H), 1.57 (d, *J* = 6.5 Hz, 3H), 1.49 (tt, *J* = 7.5, 7.5 Hz, 2H), 1.35–1.18 (m, 6H), 0.86 (t, *J* = 6.7 Hz, 3H). ^13^C­{^1^H} NMR (100 MHz, CDCl_3_) δ 168.0, 143.6, 134.3, 128.6, 127.6, 127.3, 126.1, 55.7, 31.5, 31.2, 28.6, 27.0, 24.0, 22.5, 14.0. HRMS (ESI) *m*/*z*: [M + H]^+^ calcd for C_17_H_25_N_2_S 289.1738; found 289.1756. IR (ATR, cm^–1^) 3192, 3084, 2966, 2925, 2853, 1589, 1575, 1558, 1493, 1463, 1451, 1372, 1354, 1319, 1277, 1216, 1169, 1129, 1085, 762, 700, 558
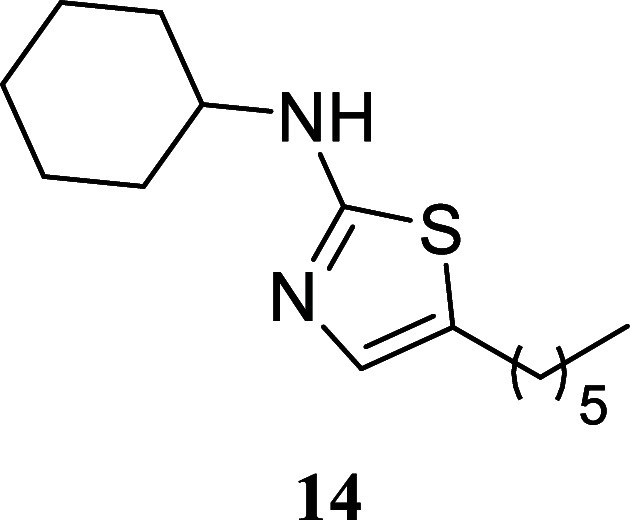
.

#### *N*-Cyclohexyl-5-hexylthiazol-2-amine (**14**)

It was obtained as a brown crystal (202.3 mg, 76%), mp 64.4–66.3 °C. Column chromatography eluent, EtOAc/hexane gradient from 1:9 to 3:2. *R*_f_ 0.49 (1:4 v/v EtOAc/hexanes). ^1^H NMR (400 MHz, CDCl_3_) δ 6.71 (t, *J* = 0.9 Hz, 1H), 5.19 (br s, 1H), 3.36–3.23 (m, 1H), 2.60 (td, *J* = 7.6, 0.9 Hz, 2H), 2.12–2.01 (m, 2H), 1.79–1.68 (m, 2H), 1.66–1.50 (m, 3H), 1.43–1.14 (m, 11H), 0.87 (t, *J* = 6.9 Hz, 3H). ^13^C­{^1^H} NMR (100 MHz, CDCl_3_) δ 167.9, 134.4, 126.6, 54.7, 33.2, 31.5, 31.3, 28.6, 27.1, 25.6, 24.8, 22.5, 14.0. HRMS (ESI) *m*/*z*: [M + H]^+^ calcd for C_15_H_27_N_2_S 267.1895; found 267.1909. IR (ATR, cm^–1^) 3191, 2925, 2852, 1566, 1533, 1509, 1450, 1248, 1161, 1132, 891, 833, 596, 523
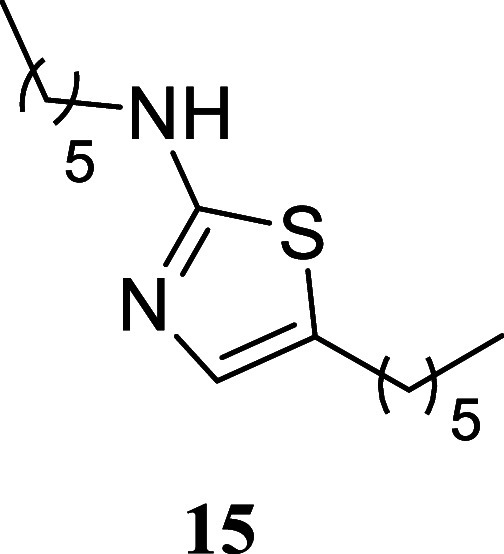
.

#### *N*,5-Dihexylthiazol-2-amine (**15**)

It was obtained as a brown oil (112.6 mg, 42%). Column chromatography eluent, EtOAc/hexane gradient from 1:9 to 3:2. *R*_f_ 0.50 (1:4 v/v EtOAc/hexanes). ^1^H NMR (400 MHz, CDCl_3_) δ 6.74 (s, 1H), 5.14 (br s, 1H), 3.21 (t, *J* = 7.0, 2H), 2.62 (t, *J* = 7.5 Hz, 2H), 1.62 (tt, *J* = 7.5, 7.5 Hz, 2H), 1.57 (tt, *J* = 7.5, 7.5 Hz, 2H), 1.42–1.23 (m, 12H), 0.89 (t, *J* = 6.8 Hz, 3H), 0.88 (t, *J* = 6.8 Hz, 3H). ^13^C­{^1^H} NMR (100 MHz, CDCl_3_) δ 169.0, 134.5, 127.0, 46.0, 31.5, 31.5, 31.3, 29.4, 28.6, 27.1, 26.6, 22.6, 14.1, 14.0. HRMS (ESI) *m*/*z*: [M + H]^+^ calcd for C_15_H_29_N_2_S 269.2051; found 269.2077. IR (ATR, cm^–1^) 3188, 3098, 2954, 2926, 2855, 1692, 1578, 1554, 1453, 1367, 1342, 1304, 1163, 1124, 846, 726, 536
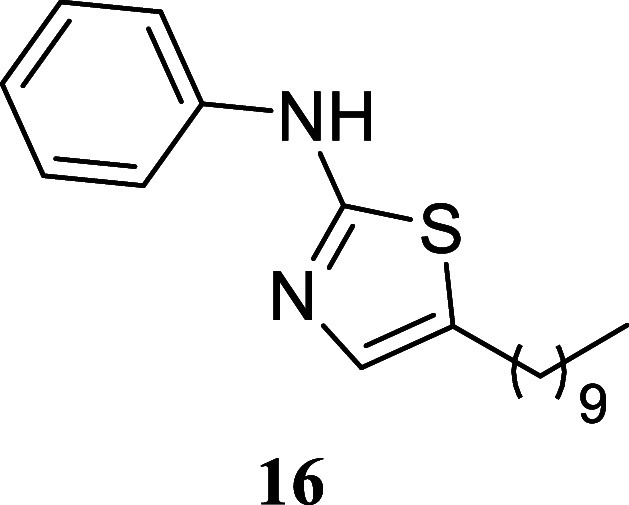
.

#### 5-Decyl-*N*-phenylthiazol-2-amine (**16**)

It was obtained as a yellow crystal (173.9 mg, 55%), mp 65.9–66.4 °C. Column chromatography eluent, EtOAc/hexane gradient from 1:9 to 3:2. *R*_f_ 0.62 (1:4 v/v EtOAc/hexanes). ^1^H NMR (400 MHz, CDCl_3_) δ 8.88 (br s, 1H), 7.38–7.30 (m, 4H), 7.07–7.00 (m, 1H), 6.95 (t, *J* = 1.0 Hz, 1H), 2.69 (td, *J* = 7.5, 1.0 Hz, 2H), 1.62 (tt, *J* = 7.5, 7.5 Hz, 2H), 1.40–1.21 (m, 14H), 0.88 (t, *J* = 6.7 Hz, 3H). ^13^C­{^1^H} NMR (100 MHz, CDCl_3_) δ 164.2, 140.9, 134.0, 129.4, 127.9, 122.4, 117.8, 31.9, 31.4, 29.6, 29.5, 29.3, 29.0, 27.0, 22.7, 14.1. HRMS (ESI) *m*/*z*: [M + H]^+^ calcd for C_19_H_29_N_2_S 317.2051; found 317.2071. IR (ATR, cm^–1^) 2924, 2851, 1603, 1570, 1498, 1463, 1428, 1369, 1321, 1301, 1159, 829, 779, 745, 670, 691, 609, 513, 495
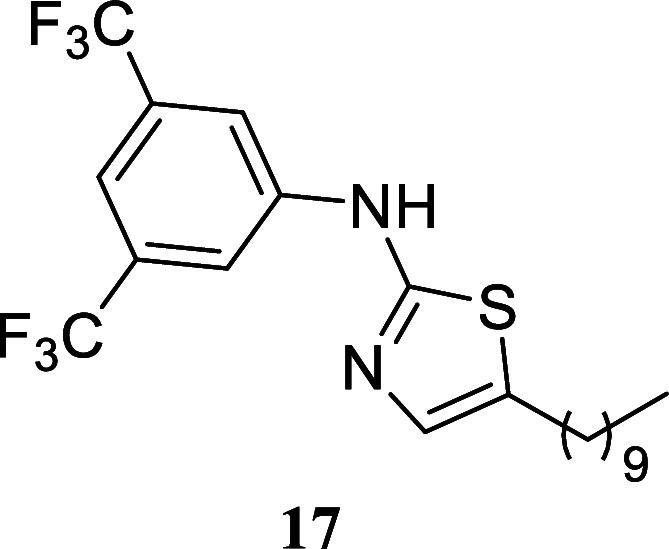
.

#### *N*-(3,5-Bis­(trifluoromethyl)­phenyl)-5-decylthiazol-2-amine (**17**)

It was obtained as a white crystal (144.7 mg, 32%), mp 71.8–73.7 °C. Column chromatography eluent, EtOAc/hexane gradient from 1:9 to 3:2. *R*_f_ 0.76 (1:4 v/v EtOAc/hexanes). ^1^H NMR (500 MHz, CDCl_3_) δ 7.89 (s, 2H), 7.46 (s, 1H), 7.01 (t, *J* = 1.0 Hz, 1H), 2.73 (td, *J* = 7.5, 1.0 Hz, 2H), 1.63 (tt, *J* = 7.3, 7.3 Hz, 2H), 1.41–1.15 (m, 14H), 0.87 (t, *J* = 6.9 Hz, 3H). ^13^C­{^1^H} NMR (125 MHz, CDCl_3_) δ 160.9, 142.0, 134.9, 132.6 (q, ^2^ *J*_C–F_ = 33.3 Hz), 130.9, 123.2 (q, ^1^ *J*_C–F_ = 272.7 Hz), 116.5–116.3 (m), 115.0–114.8 (m), 31.9, 31.3, 29.6, 29.5, 29.29, 29.26, 28.9, 26.9, 22.7, 14.1. ^19^F­{^1^H} NMR (376 MHz, CDCl_3_) δ – 63.08. HRMS (ESI) *m*/*z*: [M + H]^+^ calcd for C_21_H_27_N_2_F_6_S 453.1799; found 453.1821. IR (ATR, cm^–1^) 2924, 2854, 1627, 1602, 1482, 1468, 1427, 1395, 1324, 1275, 1179, 1167, 1120, 1112, 999, 968, 872, 687
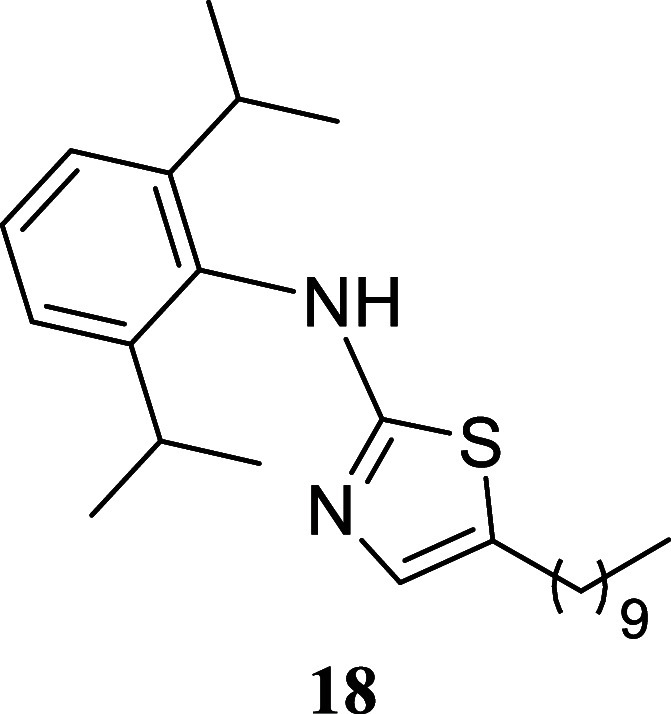
.

#### 5-Decyl-*N*-(2,6-diisopropylphenyl)­thiazol-2-amine (**18**)

It was obtained as a yellow oil (124.2 mg, 31%). Column chromatography eluent, EtOAc/hexane gradient from 1:9 to 3:2. *R*_f_ 0.54 (1:4 v/v EtOAc/hexanes). ^1^H NMR (500 MHz, CDCl_3_) δ 7.34 (t, *J* = 7.7 Hz, 1H), 7.22 (d, *J* = 7.7 Hz, 2H), 6.77 (t, *J* = 1.0 Hz, 1H), 3.29 (hept, *J* = 6.9 Hz, 2H), 2.54 (td, *J* = 7.6, 1.0 Hz, 2H), 1.49 (tt, *J* = 7.3, 7.3 Hz, 2H), 1.31–1.20 (m, 14H), 1.18 (d, *J* = 6.9 Hz, 12H), 0.87 (t, *J* = 7.0 Hz, 3H). ^13^C­{^1^H} NMR (125 MHz, CDCl_3_) δ 170.5, 147.7, 134.9, 134.5, 128.7, 127.8, 124.2, 31.9, 31.1, 29.53, 29.50, 29.27, 29.26, 28.9, 28.3, 27.1, 23.9, 22.7, 14.1. HRMS (ESI) *m*/*z*: [M + H]^+^ calcd for C_25_H_41_N_2_S 401.2990; found 401.3015. IR (ATR, cm^–1^) 3160, 2960, 2925, 2854, 1552, 1463, 1433, 1383, 1362, 1330, 1255, 1153, 812, 748
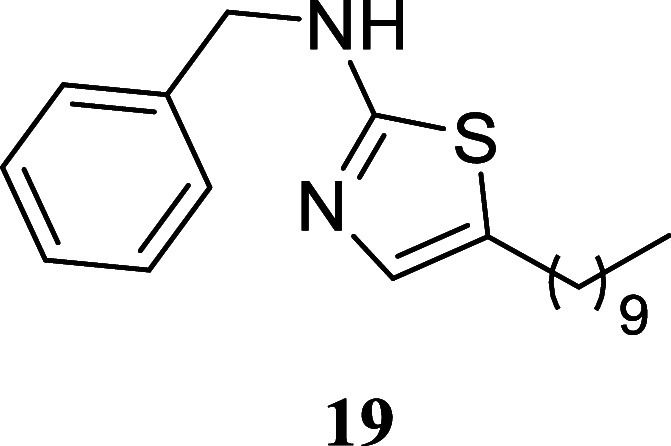
.

#### *N*-Benzyl-5-decylthiazol-2-amine (**19**)

It was obtained as a white crystal (148.6 mg, 45%), mp 79.6–80.2 °C. Column chromatography eluent, EtOAc/hexane gradient from 1:9 to 3:2. *R*_f_ 0.35 (1:4 v/v EtOAc/hexanes). ^1^H NMR (500 MHz, CDCl_3_) δ 7.39–7.33 (m, 4H), 7.31–7.27 (m, 1H), 6.74 (t, *J* = 1.0 Hz, 1H), 5.40 (br s, 1H), 4.45 (s, 2H), 2.61 (td, *J* = 7.5, 1.0 Hz, 2H), 1.56 (tt, *J* = 7.6, 7.6 Hz, 2H), 1.36–1.21 (m, 14H), 0.88 (t, *J* = 7.0 Hz, 3H). ^13^C­{^1^H} NMR (125 MHz, CDCl_3_) δ 168.4, 137.9, 134.6, 128.7, 127.7, 127.7, 127.6, 49.7, 31.9, 31.3, 29.6, 29.5, 29.3, 29.0, 27.1, 22.7, 14.1. HRMS (ESI) *m*/*z*: [M + H]^+^ calcd for C_20_H_31_N_2_S 331.2208; found 331.2227. IR (ATR, cm^–1^) 3176, 3063, 2963, 2952, 2916, 2849, 1589, 1576, 1561, 1461, 1452, 1349, 1165, 1100, 858, 837, 777, 755, 721, 701, 608, 506, 498
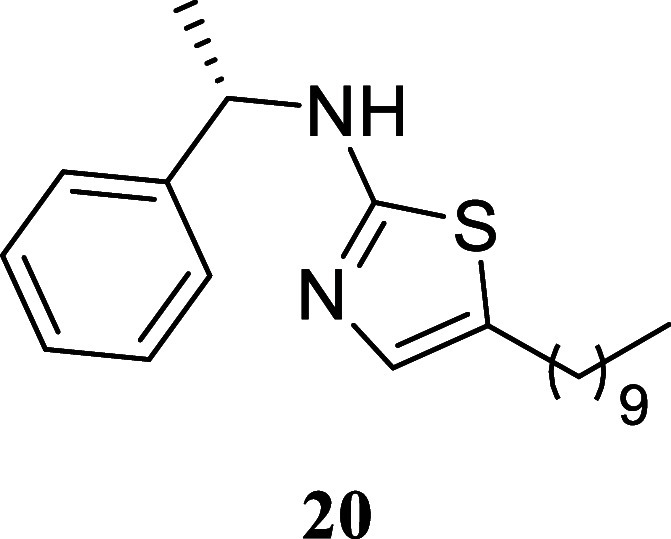
.

#### (*S*)-5-Decyl-*N*-(1-phenylethyl)­thiazol-2-amine (**20**)

It was obtained as a yellow oil (286.0 mg, 83%). α_[D]_^20^ – 47.8 (*c* 0.17, CHCl_3_). Column chromatography eluent, EtOAc/hexane gradient from 1:9 to 3:2. *R*_f_ 0.29 (1:4 v/v EtOAc/hexanes). ^1^H NMR (500 MHz, CDCl_3_) δ 7.39–7.31 (m, 4H), 7.29–7.24 (m, 1H), 6.72 (s, 1H), 6.04 (br s, 1H), 4.56 (br s, 1H), 2.55 (t, *J* = 7.5 Hz, 2H), 1.57 (d, *J* = 6.8 Hz, 3H), 1.50 (tt, *J* = 7.5, 7.5 Hz, 2H), 1.34–1.20 (m, 14H), 0.88 (t, *J* = 7.0 Hz, 3H). ^13^C­{^1^H} NMR (125 MHz, CDCl_3_) δ 168.0, 143.6, 134.2, 128.6, 127.6, 127.3, 126.1, 55.7, 31.9, 31.3, 29.6, 29.5, 29.28, 29.26, 29.0, 27.0, 24.0, 22.7, 14.1. HRMS (ESI) *m*/*z*: [M + H]^+^ calcd for C_21_H_33_N_2_S 345.2364; found 345.2372. IR (ATR, cm^–1^) 3195, 3064, 2924, 2853, 1574, 1554, 1449, 1374, 1351, 1319, 1164, 1129, 1084, 1760, 698
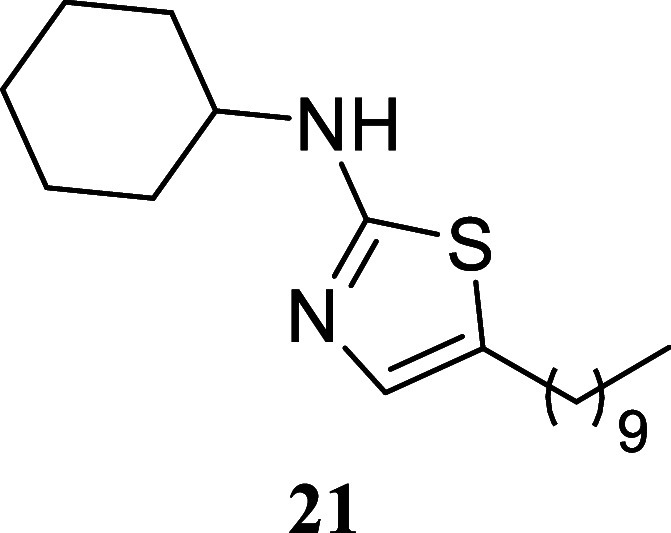
.

#### *N*-Cyclohexyl-5-decylthiazol-2-amine (**21**)

It was obtained as a yellow crystal (241.9 mg, 75%), mp 52.2–54.1 °C. Column chromatography eluent, EtOAc/hexane gradient from 1:9 to 3:2. *R*_f_ 0.43 (1:4 v/v EtOAc/hexanes). ^1^H NMR (400 MHz, CDCl_3_) δ 6.71 (s, 1H), 5.09 (br s, 1H), 3.38–3.23 (m, 1H), 2.60 (t, *J* = 7.4 Hz, 2H), 2.14–2.00 (m, 2H), 1.80–1.67 (m, 2H), 1.66–1.48 (m, 3H), 1.43–1.14 (m, 20H), 0.87 (t, *J* = 6.8 Hz, 3H). ^13^C­{^1^H} NMR (100 MHz, CDCl_3_) δ 168.0, 134.3, 126.5, 54.7, 33.2, 31.8, 31.3, 29.53, 29.50, 29.28, 29.26, 28.9, 27.1, 25.6, 24.8, 22.6, 14.1. HRMS (ESI) *m*/*z*: [M + H]^+^ calcd for C_19_H_35_N_2_S 323.2521; found 323.2548. IR (ATR, cm^–1^) 3186, 3081, 2918, 2849, 1580, 1557, 1460, 1370, 1322, 1156, 1136, 1111, 836, 722, 525
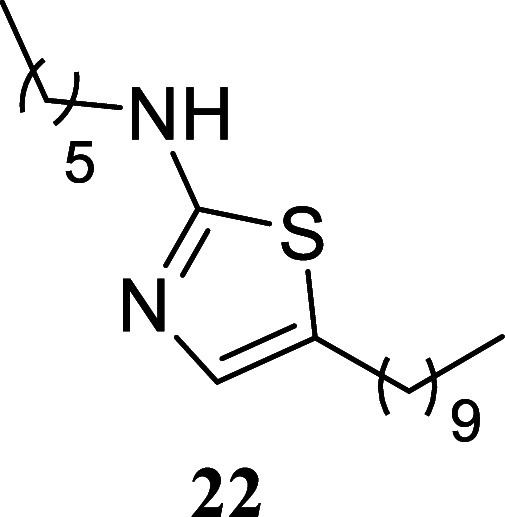
.

#### 5-Decyl-*N*-hexylthiazol-2-amine (**22**)

It was obtained as a white crystal (227.2 mg, 70%), mp 36.5–38.2 °C. Column chromatography eluent, EtOAc/hexane gradient from 1:9 to 3:2. *R*_f_ 0.45 (1:4 v/v EtOAc/hexanes). ^1^H NMR (500 MHz, CDCl_3_) δ 6.74 (t, *J* = 1.0 Hz, 1H), 5.18 (br s, 1H), 3.21 (t, *J* = 7.1, 2H), 2.61 (td, *J* = 7.5, 1.0 Hz, 2H), 1.62 (tt, *J* = 7.4, 7.4 Hz, 2H), 1.56 (tt, *J* = 7.4, 7.4 Hz, 2H), 1.41–1.21 (m, 20H), 0.89 (t, *J* = 7.1 Hz, 3H), 0.87 (t, *J* = 7.1 Hz, 3H). ^13^C­{^1^H} NMR (125 MHz, CDCl_3_) δ 169.0, 134.6, 127.0, 46.0, 31.9, 31.5, 31.3, 29.6, 29.5, 29.4, 29.31, 29.30, 28.9, 27.1, 26.6, 22.7, 22.6, 14.1, 14.0. HRMS (ESI) *m*/*z*: [M + H]^+^ calcd for C_19_H_37_N_2_S 325.2677; found 325.2700. IR (ATR, cm^–1^) 3193, 3102, 2952, 2916, 2850, 1684, 1586, 1558, 1457, 1370, 1340, 1317, 1171, 1126, 838, 718, 533
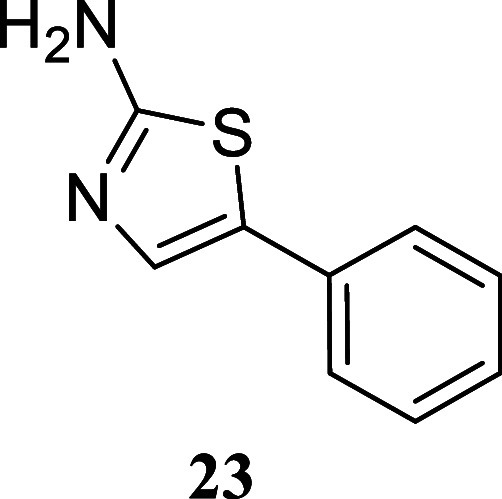
.

#### 5-Phenylthiazol-2-amine (**23**)^32^

It was obtained as a white crystal (167.2 mg, 95%), mp 145.5–147.0 °C. Column chromatography eluent, EtOAc/hexane gradient from 1:9 to 3:2. *R*_f_ 0.21 (2:3 v/v EtOAc/hexanes). ^1^H NMR (400 MHz, CDCl_3_) δ 7.44–7.40 (m, 2H), 7.37–7.32 (m, 2H), 7.31 (s, 1H), 7.26–7.21 (m, 1H), 4.98 (br s, 2H). ^13^C­{^1^H} NMR (100 MHz, CDCl_3_) δ 166.8, 134.3, 132.1, 129.3, 128.9, 127.0, 125.6. HRMS (ESI) *m*/*z*: [M + H]^+^ calcd for C_9_H_9_N_2_S 177.0486; found 177.0484. IR (ATR, cm^–1^) 3418, 3268, 3053, 1628, 1599, 1540, 1496, 1442, 1333, 1209, 1047, 853, 748, 686, 587, 488
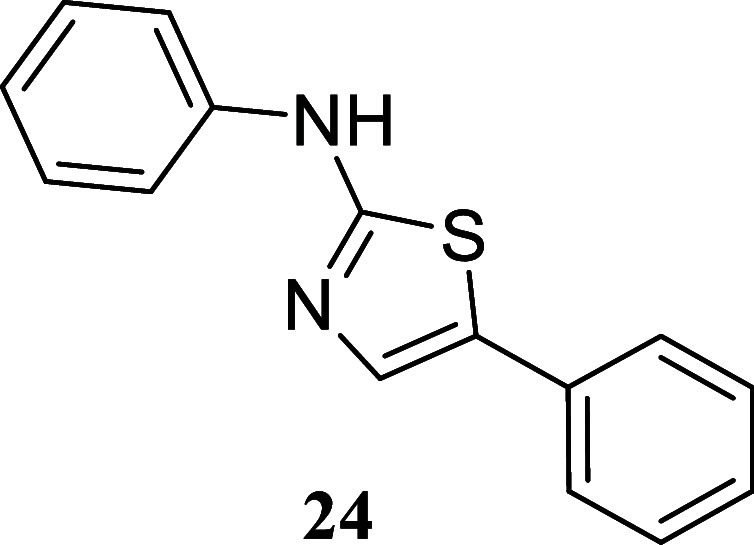
.

#### *N*,5-Diphenylthiazol-2-amine (**24**)^33^

It was obtained as a colorless crystal (131.1 mg, 52%), mp 152.9–154.5 °C. Column chromatography eluent, EtOAc/hexane gradient from 1:9 to 3:2. *R*_f_ 0.51 (1:4 v/v EtOAc/hexanes). ^1^H NMR (400 MHz, CDCl_3_) δ 8.83 (br s, 1H), 7.51 (s, 1H), 7.50–7.46 (m, 2H), 7.43–7.34 (m, 6H), 7.28–7.23 (m, 1H), 7.14–7.07 (m, 1H). ^13^C­{^1^H} NMR (100 MHz, CDCl_3_) δ 164.9, 140.4, 133.9, 133.0, 129.6, 128.9, 127.4, 127.1, 125.6, 123.2, 118.4. HRMS (ESI) *m*/*z*: [M + H]^+^ calcd for C_15_H_13_N_2_S 253.0799; found 253.0809. IR (ATR, cm^–1^) 2901, 1616, 1598, 1571, 1536, 1497, 1459, 1429, 1320, 1212, 1166, 841, 779, 743, 683, 696, 683, 643, 607, 588, 481, 501
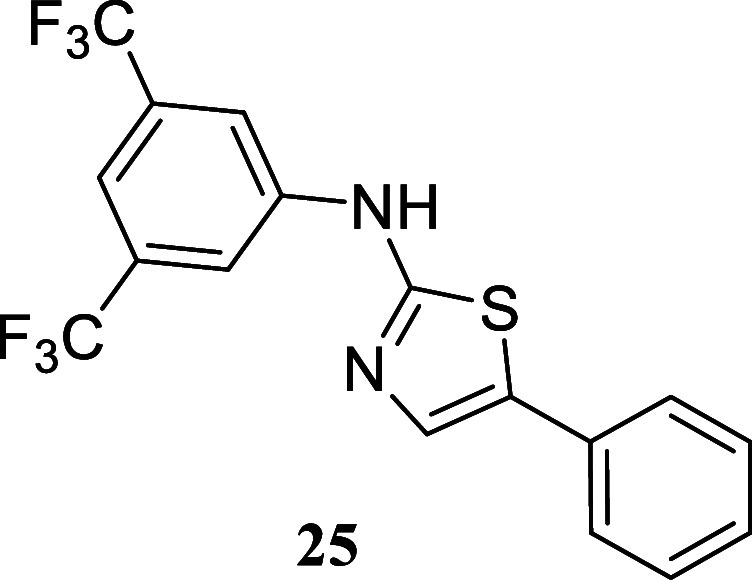
.

#### *N*-(3,5-Bis­(trifluoromethyl)­phenyl)-5-phenylthiazol-2-amine (**25**)

It was obtained as a white crystal (147.5 mg, 38%), mp 163.6–165.3 °C. Column chromatography eluent, EtOAc/hexane gradient from 1:9 to 3:2. *R*_f_ 0.57 (1:4 v/v EtOAc/hexanes). ^1^H NMR (500 MHz, CDCl_3_) δ 8.32 (br s, 1H), 7.96 (br s, 2H), 7.56 (s, 1H), 7.53 (br s, 1H), 7.51–7.48 (m, 2H), 7.42–7.37 (m, 2H), 7.33–7.29 (m, 1H). ^13^C­{^1^H} NMR (125 MHz, CDCl_3_) δ 161.8, 141.6, 134.1, 132.8 (q, ^2^ *J*_C–F_ = 33.4 Hz), 131.2, 130.1, 129.1, 127.9, 126.0, 123.2 (q, ^1^ *J*_C–F_ = 272.8 Hz), 117.0–116.8 (m), 115.6–115.4 (m). ^19^F­{^1^H} NMR (376 MHz, CDCl_3_) δ – 63.06. HRMS (ESI) *m*/*z*: [M + H]^+^ calcd for C_17_H_11_N_2_F_6_S 389.0547; found 389.0556. IR (ATR, cm^–1^) 3085, 2936, 1624, 1602, 1545, 1476, 1429, 1389, 1324, 1274, 1170, 1122, 1112, 999, 973, 961, 879, 752, 682, 663, 478
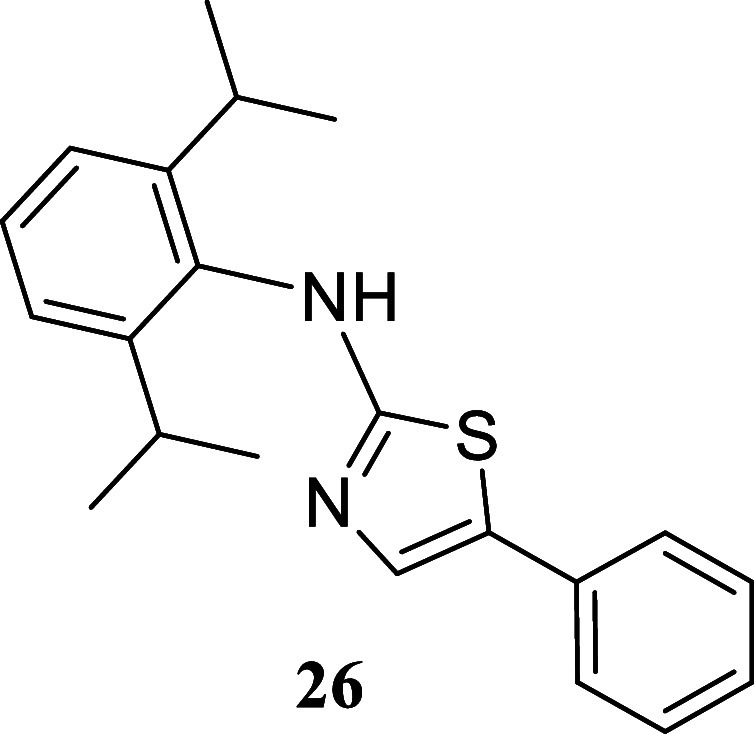
.

#### *N*-(2,6-Diisopropylphenyl)-5-phenylthiazol-2-amine (**26**)

It was obtained as a white crystal (238.7 mg, 71%), mp 178.0–178.4 °C. Column chromatography eluent, EtOAc/hexane gradient from 1:9 to 3:2. *R*_f_ 0.35 (1:4 v/v EtOAc/hexanes). ^1^H NMR (500 MHz, CDCl_3_) δ 7.40 (br t, *J* = 7.7 Hz, 1H), 7.37–7.31 (m, 3H), 7.29–7.26 (m, 3H), 7.18–7.14 (m, 1H), 3.34 (hept, *J* = 6.8 Hz, 2H), 1.22 (d, *J* = 6.9 Hz, 12H). ^13^C­{^1^H} NMR (125 MHz, CDCl_3_) δ 171.5, 147.8, 134.6, 134.4, 132.4, 129.1, 128.8, 127.0, 126.6, 125.1, 124.3, 28.4, 24.0. HRMS (ESI) *m*/*z*: [M + H]^+^ calcd for C_21_H_25_N_2_S 337.1738; found 337.1735. IR (ATR, cm^–1^) 3146, 2961, 2865, 1566, 1533, 1425, 1329, 1156, 810, 784, 757, 748, 689, 626, 484
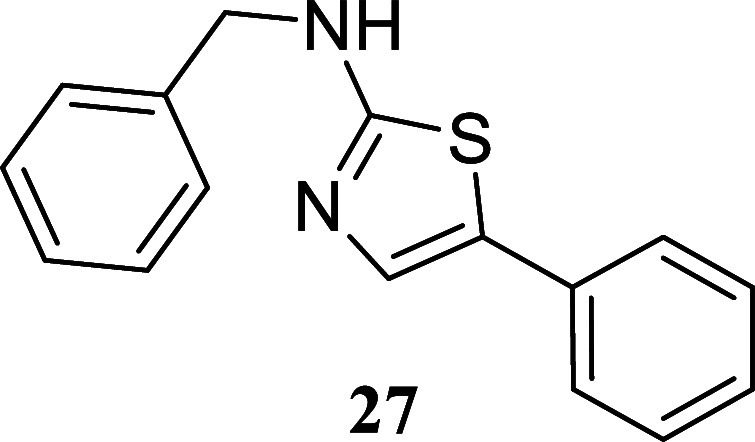
.

#### *N*-Benzyl-5-phenylthiazol-2-amine (**27**)

It was obtained as a white crystal (106.4 mg, 40%), mp 147.8–149.6 °C. Column chromatography eluent, EtOAc/hexane gradient from 1:9 to 3:2. *R*_f_ 0.30 (1:4 v/v EtOAc/hexanes). ^1^H NMR (400 MHz, CDCl_3_) δ 7.36–7.30 (m, 5H), 7.30–7.23 (m, 3H), 7.20 (d, *J* = 7.1 Hz, 2H), 7.17–7.12 (m, 1H), 5.84 (br s, 1H), 4.45 (s, 2H). ^13^C­{^1^H} NMR (100 MHz, CDCl_3_) δ 169.0, 137.4, 134.6, 132.4, 128.84, 128.78, 127.8, 127.7, 127.0, 126.7, 125.3, 49.8. HRMS (ESI) *m*/*z*: [M + H] + calcd for C_16_H_15_N_2_S 267.0956; found 267.0973. IR (ATR, cm^–1^) 3169, 3064, 2845, 1575, 1535, 1453, 1347, 1189, 1169, 1098, 1028, 860, 836, 776, 745, 701, 681, 626, 508, 480
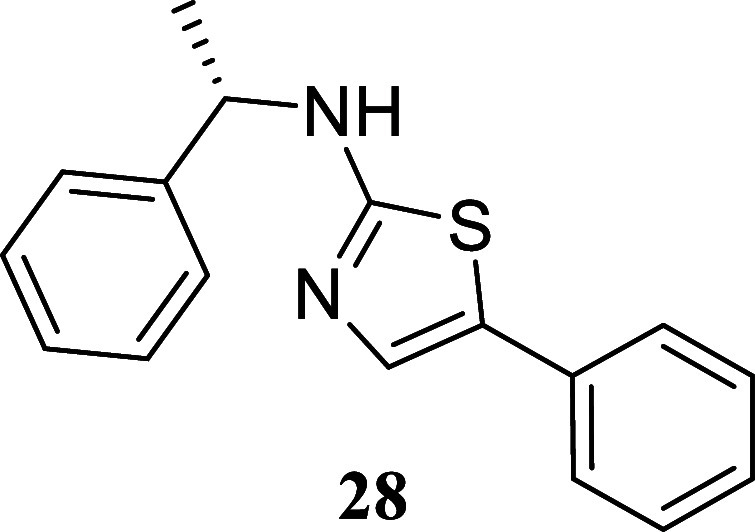
.

#### (*S*)-5-Phenyl-*N*-(1-phenylethyl)­thiazol-2-amine (**28**)

It was obtained as a white crystal (224.1 mg, 80%), mp 122.5–124.2 °C. α_[D]_^20^ – 206.2 (*c* 0.13, CHCl_3_). Column chromatography eluent, EtOAc/hexane gradient from 1:9 to 3:2. *R*_f_ 0.24 (1:4 v/v EtOAc/hexanes). ^1^H NMR (400 MHz, CDCl_3_) δ 7.46–7.40 (m, 2H), 7.40–7.33 (m, 4H), 7.32–7.27 (m, 4H), 7.21–7.14 (m, 1H), 4.66–4.56 (m, 1H), 1.65 (d, *J* = 7.1 Hz, 3H). ^13^C­{^1^H} NMR (100 MHz, CDCl_3_) δ 169.3, 143.2, 134.0, 132.5, 128.74, 128.72, 127.49, 126.48, 126.22, 126.18, 125.2, 56.1, 24.0. HRMS (ESI) *m*/*z*: [M + H]^+^ calcd for C_17_H_17_N_2_S 281.1112; found 281.1128. IR (ATR, cm^–1^) 3179, 3028, 2962, 2870, 1571, 1532, 1443, 1172, 1127, 1088, 839, 751, 697, 686, 629, 615, 554, 484
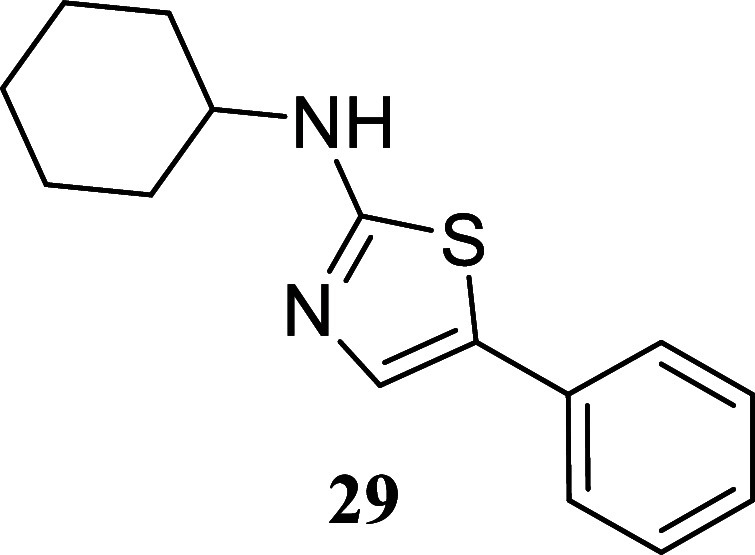
.

#### *N*-Cyclohexyl-5-phenylthiazol-2-amine (**29**)

It was obtained as a white crystal (227.1 mg, 88%), mp 142.8–143.9 °C. Column chromatography eluent, EtOAc/hexane gradient from 1:9 to 3:2. *R*_f_ 0.32 (1:4 v/v EtOAc/hexanes). ^1^H NMR (400 MHz, CDCl_3_) δ 7.44–7.37 (m, 2H), 7.35–7.28 (m, 3H), 7.23–7.16 (m, 1H), 5.49 (br s, 1H), 3.47–3.29 (m, 1H), 2.18–2.07 (m, 2H), 1.88–1.72 (m, 2H), 1.71–1.59 (m, 1H), 1.50–1.17 (m, 5H). ^13^C­{^1^H} NMR (100 MHz, CDCl_3_) δ 168.5, 134.5, 132.6, 128.8, 126.5, 126.1, 125.2, 55.0, 33.1, 25.5, 24.8. HRMS (ESI) *m*/*z*: [M + H]^+^ calcd for C_15_H_19_N_2_S 259.1269; found 259.1279. IR (ATR, cm^–1^) 3182, 3063, 2934, 2853, 1571, 1535, 1446, 1368, 1200, 1160, 1139, 1106, 850, 747, 684, 599, 572, 482, 462
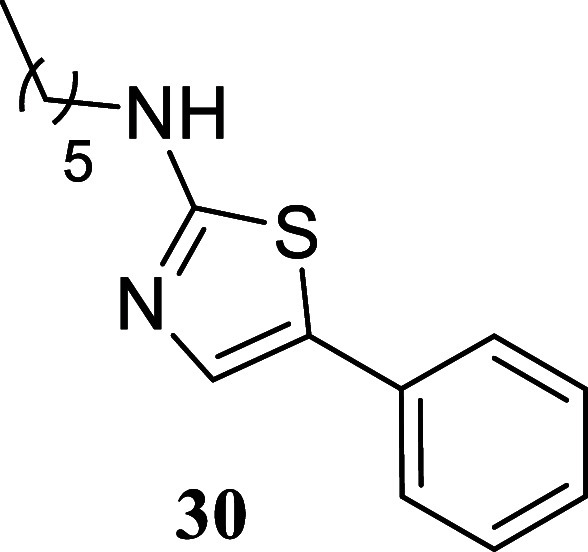
.

#### *N*-Hexyl-5-phenylthiazol-2-amine (**30**)

It was obtained as a white crystal (106.7 mg, 41%), mp 87.8–89.0 °C. Column chromatography eluent, EtOAc/hexane gradient from 1:9 to 3:2. *R*_f_ 0.81 (1:4 v/v EtOAc/hexanes). ^1^H NMR (400 MHz, CDCl_3_) δ 7.44–7.39 (m, 2H), 7.36–7.30 (m, 2H), 7.34 (s, 1H), 7.23–7.18 (m, 1H), 5.62 (br s, 1H), 3.29 (t, *J* = 7.1, 2H), 1.68 (tt, *J* = 7.1, 7.1 Hz, 2H), 1.47–1.27 (m, 6H), 0.91 (t, *J* = 6.9 Hz, 3H). ^13^C­{^1^H} NMR (100 MHz, CDCl_3_) δ 169.7, 134.7, 132.6, 128.8, 126.5, 126.3, 125.2, 46.2, 31.5, 29.3, 26.6, 22.6, 14.0. HRMS (ESI) *m*/*z*: [M + H]^+^ calcd for C_15_H_21_N_2_S 261.1425; found 261.1436. IR (ATR, cm^–1^) 3186, 3087, 2952, 2928, 2856, 1580, 1538, 1450, 1436, 1372, 1338, 1177, 1128, 1089, 845, 747, 725, 683, 619, 605, 515, 478
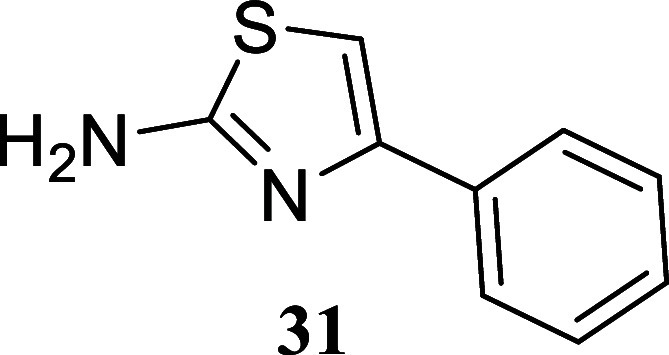
.

#### 4-Phenylthiazol-2-amine (**31**)^34^

It was obtained as a colorless crystal (156.7 mg, 89%), mp 145.9–147.4 °C. Column chromatography eluent, EtOAc/hexane gradient from 1:9 to 3:2. *R*_f_ 0.48 (2:3 v/v EtOAc/hexanes). ^1^H NMR (400 MHz, CDCl_3_) δ 7.82–7.74 (m, 2H), 7.42–7.34 (m, 2H), 7.32–7.26 (m, 1H), 6.72 (s, 1H), 5.22 (br s, 2H). ^13^C­{^1^H} NMR (125 MHz, CDCl_3_) δ 167.4, 151.3, 134.7, 128.6, 127.7, 126.0, 102.8. HRMS (ESI) *m*/*z*: [M + H]^+^ calcd for C_9_H_9_N_2_S 177.0486; found 177.0488. IR (ATR, cm^–1^) 3418, 3268, 3053, 1628, 1599, 1496, 1442, 1333, 1209, 1047, 853, 748, 686, 587, 488
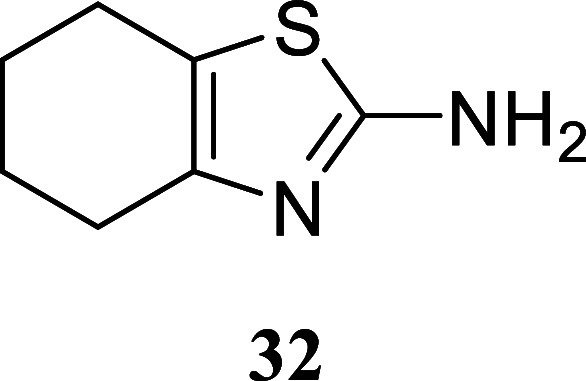
.

#### 4,5,6,7-Tetrahydrobenzo­[*d*]­thiazol-2-amine (**32**)^35^

It was obtained as a yellow oil (103.2 mg, 67%). Column chromatography eluent, EtOAc/hexane gradient from 1:9 to 3:2. *R*_f_ 0.13 (2:3 v/v EtOAc/hexanes). ^1^H NMR (400 MHz, CDCl_3_) δ 4.93 (br s, 2H), 2.60–2.46 (m, 4H), 1.85–1.73 (m, 4H). ^13^C­{^1^H} NMR (100 MHz, CDCl_3_) δ 165.0, 145.3, 118.1, 26.5, 23.5, 23.1, 22.9. HRMS (ESI) *m*/*z*: [M + H]^+^ calcd for C_7_H_11_N_2_S 155.0643; found 155.0629. IR (ATR, cm^–1^) 3291, 3170, 2926, 2841, 1613, 1585, 1519, 1442, 1366, 1306, 1276, 1239, 1098, 894, 680, 539
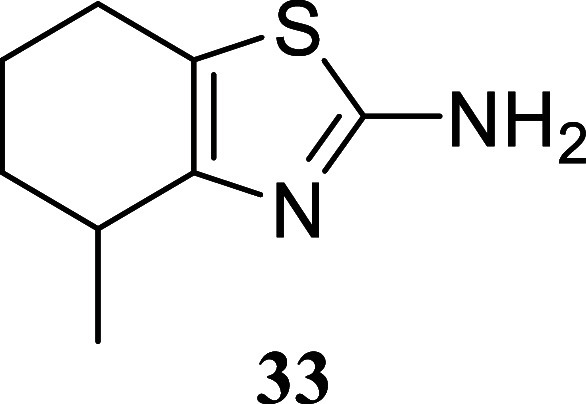
.

#### 4-Methyl-4,5,6,7-tetrahydrobenzo­[*d*]­thiazol-2-amine (**33**)

It was obtained as a yellow oil (63.9 mg, 38%). Column chromatography eluent, EtOAc/hexane gradient from 1:9 to 3:2. *R*_f_ 0.24 (2:3 v/v EtOAc/hexanes). ^1^H NMR (400 MHz, CDCl_3_) δ 4.99 (br s, 2H), 2.76–2.64 (m, 1H), 2.58–2.48 (m, 2H), 1.95–1.81 (m, 2H), 1.78–1.65 (m, 1H), 1.50–1.38 (m, 1H), 1.21 (d, *J* = 7.0 Hz, 3H). ^13^C­{^1^H} NMR (100 MHz, CDCl_3_) δ 164.9, 150.1, 118.1, 31.5, 31.4, 23.5, 21.4, 20.4. HRMS (ESI) *m*/*z*: [M + H]^+^ calcd for C_8_H_13_N_2_S 169.0799; found 169.0792. IR (ATR, cm^–1^) 3289, 3128, 2927, 2847, 1613, 1520, 1455, 1372, 1347, 1333, 1302, 1240, 1086
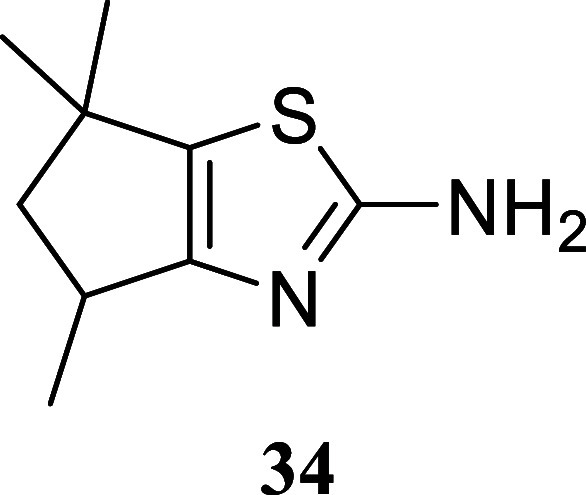
.

#### 4,6,6-Trimethyl-5,6-dihydro-4*H*-cyclopenta­[*d*]­thiazol-2-amine (**34**)

It was obtained as an amorphous solid (127.5 mg, 70%). Column chromatography eluent, EtOAc/hexane gradient from 1:9 to 3:2. *R*_f_ 0.56 (2:3 v/v EtOAc/hexanes). ^1^H NMR (500 MHz, CDCl_3_) δ 4.87 (br s, 2H), 3.07 (qt, *J* = 6.7, 6.7 Hz, 1H), 2.39 (dd, *J* = 12.5, 7.8 Hz, 1H), 1.74 (dd, *J* = 12.5, 6.7 Hz, 1H), 1.30 (s, 3H), 1.24 (d, *J* = 6.9 Hz, 3H), 1.21 (s, 3H). ^13^C­{^1^H} NMR (125 MHz, CDCl_3_) δ 170.8, 157.0, 132.3, 52.5, 40.9, 34.8, 31.0, 29.6, 20.2. HRMS (ESI) *m*/*z*: [M + H]^+^ calcd for C_9_H_15_N_2_S 183.0956; found 183.0959. IR (ATR, cm^–1^) 2959, 1696, 1523, 1454, 1363, 1089, 613
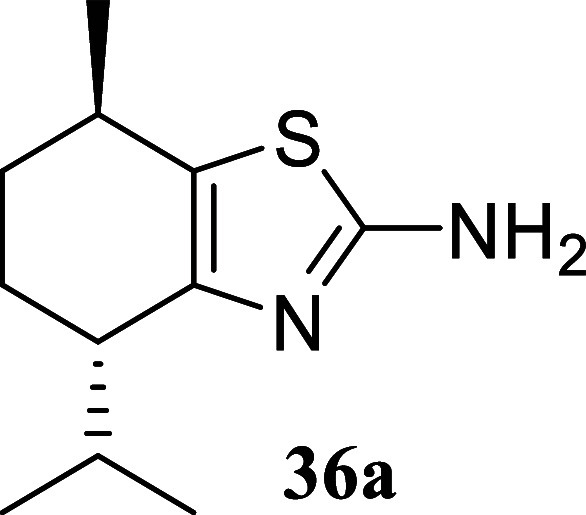
.

#### (4*S*,7*R*)-4-Isopropyl-7-methyl-4,5,6,7-tetrahydrobenzo­[*d*]­thiazol-2-amine (**36a**)

It was obtained as a yellow oil (23.1 mg, 11%). α_[D]_^20^ – 86.3 (*c* 0.12, 13:1 *d.r.*, CHCl_3_). Column chromatography eluent, EtOAc/hexane gradient from 1:9 to 3:2. *R*_f_ 0.79 (2:3 v/v EtOAc/hexanes). ^1^H NMR (500 MHz, CDCl_3_) δ 4.74 (br s, 2H), 2.80–2.73 (m, 1H), 2.62–2.55 (m, 1H), 2.44 (qqd, *J* = 7.0, 7.0, 3.8 Hz, 1H), 1.99 (dddd, *J* = 12.7, 5.0, 5.0, 2.8 Hz, 1H), 1.80 (dddd, *J* = 13.4, 5.5, 5.5, 2.7 Hz, 1H), 1.45 (dddd, *J* = 13.0, 13.0, 10.3, 2.8 Hz, 1H), 1.29 (dddd, *J* = 12.7, 12.7, 10.0, 2.7 Hz, 1H), 1.16 (d, *J* = 6.8 Hz, 3H), 0.97 (d, *J* = 7.0 Hz, 3H), 0.69 (d, *J* = 6.9 Hz, 3H). ^13^C­{^1^H} NMR (125 MHz, CDCl_3_) δ 164.5, 148.7, 126.9, 42.5, 32.6, 30.4, 29.8, 23.4, 21.9, 20.3, 16.7. HRMS (ESI) *m*/*z*: [M + H]^+^ calcd for C_11_H_19_N_2_S 211.1269; found 211.1277. IR (ATR, cm^–1^) 3293, 3176, 2956, 2929, 2868, 1618, 1524, 1454, 1366, 1323, 1096
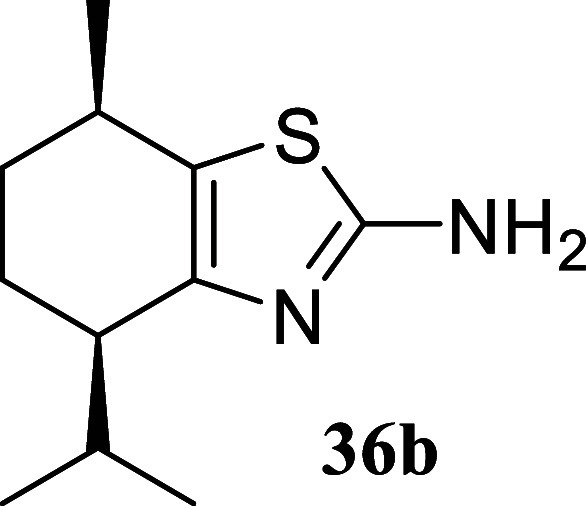
.

#### (4*R*,7*R*)-4-Isopropyl-7-methyl-4,5,6,7-tetrahydrobenzo­[*d*]­thiazol-2-amine (**36b**)

It was obtained as a yellow oil (44.2 mg, 21%). α_[D]_^20^ + 9.2 (*c* 0.13, 19:1 *d.r.*, CHCl_3_). Column chromatography eluent, EtOAc/hexane gradient from 1:9 to 3:2. *R*_f_ 0.49 (2:3 v/v EtOAc/hexanes). ^1^H NMR (500 MHz, CDCl_3_) δ 4.75 (br s, 2H), 2.86–2.78 (m, 1H), 2.54–2.47 (m, 1H), 2.30 (qqd, *J* = 6.9, 6.9, 4.5 Hz, 1H), 1.83 (dddd, *J* = 12.6, 5.9, 5.9, 3.0 Hz, 1H), 1.74 (dddd, *J* = 13.2, 9.2, 6.6, 2.7 Hz, 1H), 1.63 (dddd, *J* = 13.2, 8.8, 6.1, 2.9 Hz, 1H), 1.56 (dddd, *J* = 12.6, 8.4, 5.6, 2.7 Hz, 1H), 1.19 (d, *J* = 6.9 Hz, 3H), 1.00 (d, *J* = 7.0 Hz, 3H), 0.77 (d, *J* = 6.9 Hz, 3H). ^13^C­{^1^H} NMR (125 MHz, CDCl_3_) δ 164.2, 148.4, 126.4, 42.1, 30.5, 30.0, 29.2, 23.2, 20.9, 20.2, 17.9. HRMS (ESI) *m*/*z*: [M + H]^+^ calcd for C_11_H_19_N_2_S 211.1269; found 211.1281. IR (ATR, cm^–1^) 3288, 3136, 2955, 2867, 1609, 1521, 1452, 1384, 1366, 1347, 1310, 1207, 1177, 1101, 990, 902, 877, 618, 528
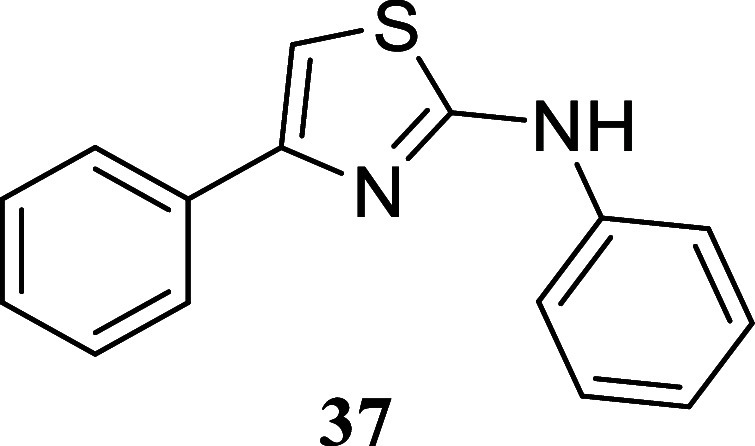
.

#### *N*,4-Diphenylthiazol-2-amine (**37**)^36^

It was obtained as a yellow crystal (121.0 mg, 48%), mp 131.5–133.1 °C. Column chromatography eluent, EtOAc/hexane gradient from 1:9 to 3:2. *R*_f_ 0.82 (2:3 v/v EtOAc/hexanes). ^1^H NMR (400 MHz, CDCl_3_) δ 7.89–7.83 (m, 2H), 7.44–7.34 (m, 6H), 7.33–7.28 (m, 1H), 7.12–7.04 (m, 1H), 6.84 (s, 1H). ^13^C­{^1^H} NMR (125 MHz, CDCl_3_) δ 164.4, 151.4, 140.3, 134.6, 129.5, 128.6, 127.9, 126.1, 123.0, 118.1, 101.9. HRMS (ESI) *m*/*z*: [M + H]^+^ calcd for C_15_H_13_N_2_S 253.0799; found 253.0817. IR (ATR, cm^–1^) 3187, 3051, 2943, 1600, 1566, 1483, 1460, 1443, 1424, 1343, 1315, 1279, 1213, 1073, 1062, 1028, 846, 771, 751, 699, 688, 661, 501
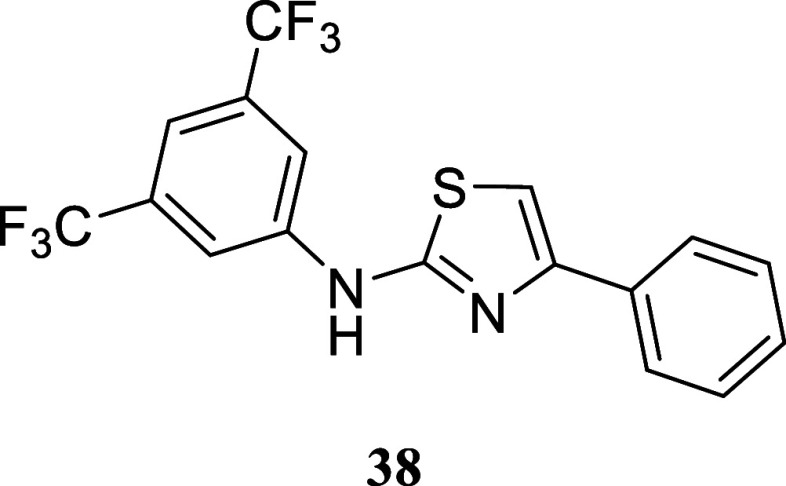
.

#### *N*-(3,5-Bis­(trifluoromethyl)­phenyl)-4-phenylthiazol-2-amine (**38**)

It was obtained as a white crystal (124.2 mg, 32%), mp 160.6–162.0 °C. Column chromatography eluent, EtOAc/hexane gradient from 1:9 to 3:2. *R*_f_ 0.76 (2:3 v/v EtOAc/hexanes). ^1^H NMR (400 MHz, CDCl_3_) δ 8.08 (br s, 2H), 7.90–7.84 (m, 2H), 7.51 (br s, 1H), 7.47–7.40 (m, 2H), 7.38–7.31 (m, 1H), 6.96 (s, 1H). ^13^C­{^1^H} NMR (100 MHz, CDCl_3_) δ 161.9, 151.8, 141.5, 134.0, 132.6 (q, ^2^ *J*_C–F_ = 33.3 Hz), 128.8, 128.3, 127.3, 126.0, 123.2 (q, ^1^ *J*_C–F_ = 273.0 Hz), 117.0–116.8 (m), 115.4–115.3 (m), 103.2. ^19^F­{^1^H} NMR (376 MHz, CDCl_3_) δ – 63.12. HRMS (ESI) *m*/*z*: [M + H]^+^ calcd for C_17_H_11_N_2_F_6_S 389.0547; found 389.0543. IR (ATR, cm^–1^) 3388, 1561, 1540, 1471, 1380, 1275, 1163, 1119, 941, 877, 724, 699, 683
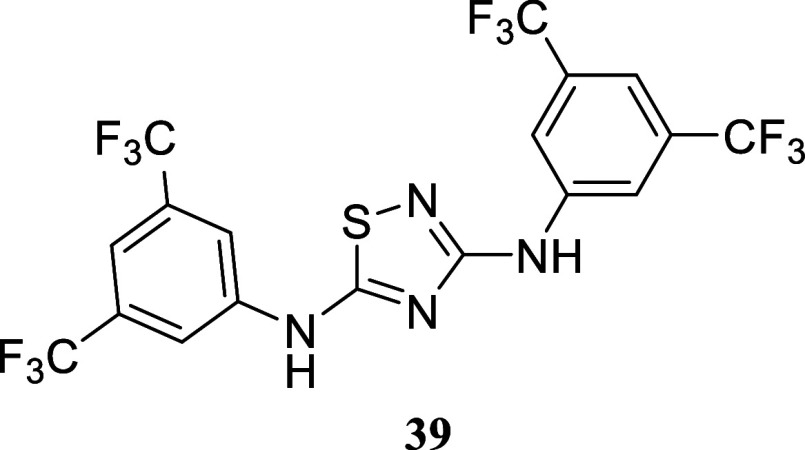
.

#### *N*3,*N*5-Bis­(3,5-bis­(trifluoromethyl)­phenyl)-1,2,4-thiadiazole-3,5-diamine (**39**)

It was obtained as a yellow crystal (200.0 mg, 57%), mp 162.7–164.2 °C. Column chromatography eluent, EtOAc/hexane gradient from 1:9 to 3:2. *R*_f_ 0.49 (1:4 v/v EtOAc/hexanes). ^1^H NMR (400 MHz, DMSO-*d*_6_) δ 7.39 (s, 2H), 7.38 (s, 3H), 6.73 (s, 1H). ^13^C­{^1^H} NMR (100 MHz, DMSO-*d*_6_) δ 160.8, 133.1, 127.4, 125.3 (q, ^2^ *J*_C–F_ = 34.1 Hz), 123.6 (q, ^2^ *J*_C–F_ = 33.2 Hz), 122.9–122.5 (m), 115.9–115.5 (m), 115.2 (q, ^1^ *J*_C–F_ = 271.8 Hz), 114.9 (q, ^1^ *J*_C–F_ = 272.3 Hz), 110.8–110.5 (m), 107.1–106.8 (m). ^19^F­{^1^H} NMR (376 MHz, DMSO-*d*_6_) δ – 64.24, – 64.64. HRMS (ESI) *m*/*z*: [M + H]^+^ calcd for C_18_H_9_N_4_F_12_S 541.0356; found 541.0359. IR (ATR, cm^–1^) 3328, 3100, 1586, 1552, 1474, 1448, 1380, 1278, 1228, 1190, 1148, 1123, 1109, 1075, 941, 885, 743, 701, 684
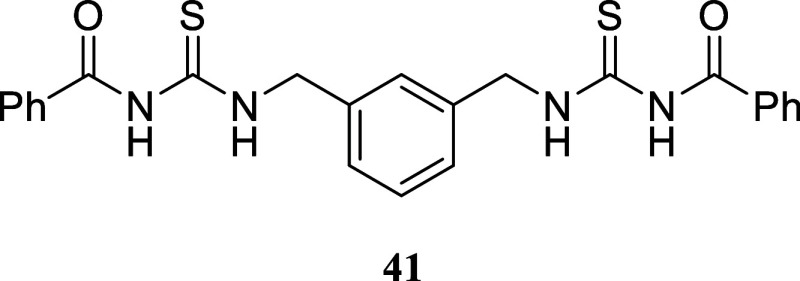
.

#### *N*,*N*′-(((1,3-Phenylenebis­(methylene))­bis­(azanediyl))­bis­(carbonothioyl))­dibenzamide (**41**)

It was obtained as a white crystal (2.155 g, 51%), mp 148.9–150.4 °C. The solid was collected by filtration and washed with cold acetonitrile. ^1^H NMR (500 MHz, DMSO-*d*_6_) δ 11.40 (s, 2H), 11.24 (t, *J* = 5.7 Hz, 2H), 7.95–7.89 (m, 4H), 7.65–7.60 (m, 2H), 7.53–7.47 (m, 4H), 7.41 (br s, 1H), 7.39–7.34 (m, 1H), 7.34–7.30 (m, 2H), 4.89 (*J* = 5.7 Hz, 4H). ^13^C­{^1^H} NMR (125 MHz, DMSO-*d*_6_) δ 180.6, 168.0, 137.7, 132.9, 132.2, 128.7, 128.6, 128.4, 126.8, 126.6, 48.0. HRMS (ESI) *m*/*z*: [M + H]^+^ calcd for C_24_H_23_N_4_O_2_S_2_ 463.1262; found 463.1275. IR (ATR, cm^–1^) 3239, 3057, 1665, 1514, 1491, 1423, 1321, 1255, 1171, 1101, 1065, 1027, 979, 771, 721, 692, 666, 606
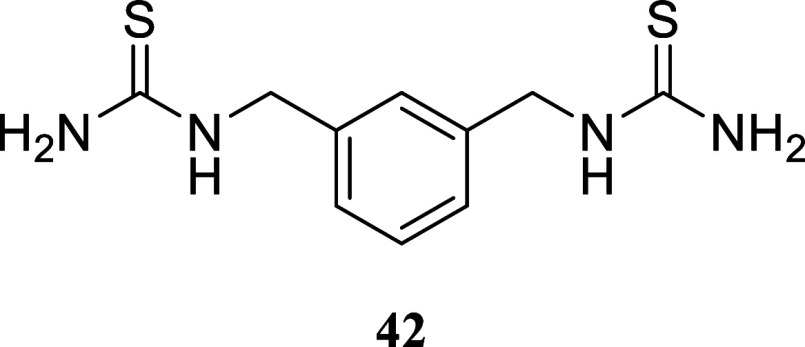
.

#### 1,1′-(1,3-Phenylenebis­(methylene))­bis­(thiourea) (**42**)

It was obtained as an amorphous solid (1.126 g, 95%). Column chromatography eluent, EtOAc/hexanes 3:2 to pure EtOAc and then EtOAc/MeOH gradient from 9:1 to 1:1. ^1^H NMR (500 MHz, DMSO-*d*_6_) δ 8.02 (br s, 2H), 7.29 (t, *J* = 7.6 Hz, 1H), 7.22–7.15 (m, 3H), 7.08 (br s, 2H), 4.61 (br s, 4H). ^13^C­{^1^H} NMR (125 MHz, DMSO-*d*_6_) δ 183.4, 139.3, 128.3, 126.3, 126.0, 47.5. HRMS (ESI) *m*/*z*: [M + H]^+^ calcd for C_10_H_15_N_4_S_2_ 255.0738; found 255.0735. IR (ATR, cm^–1^) 3379, 3258, 3176, 3073, 1603, 1544, 1440, 1355, 1296, 1165, 1148, 807, 735, 697, 606, 544, 505
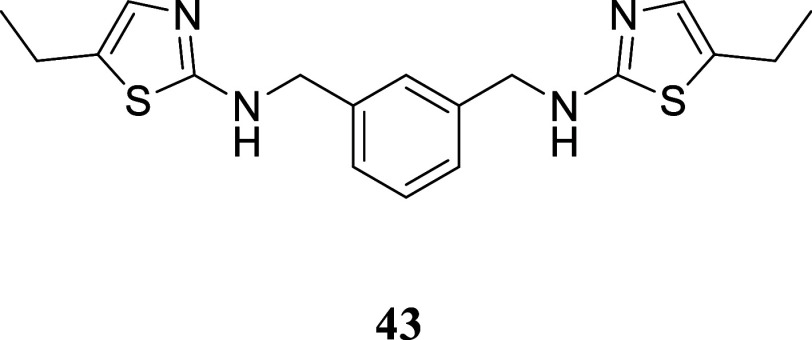
.

#### *N*,*N*′-(1,3-Phenylenebis­(methylene))­bis­(5-ethylthiazol-2-amine) (**43**)

It was obtained as a yellow oil (96.7 mg, 27%). Column chromatography eluent, EtOAc/hexane gradient from 1:9 to 9:1. *R*_f_ 0.39 (3:2 v/v EtOAc/hexanes). ^1^H NMR (500 MHz, CDCl_3_) δ 7.36 (br s, 1H), 7.35–7.27 (m, 3H), 6.77 (br s, 2H), 5.30 (br s, 2H), 4.45 (s, 4H), 2.66 (qd, *J* = 7.5, 1.2 Hz, 4H), 1.23 (t, *J* = 7.5 Hz, 6H). ^13^C­{^1^H} NMR (125 MHz, CDCl_3_) δ 168.2, 138.6, 134.0, 129.4, 129.1, 126.9, 126.8, 49.5, 20.6, 15.8. HRMS (ESI) *m*/*z*: [M + H]^+^ calcd for C_18_H_23_N_4_S_2_ 359.1364; found 359.1362. IR (ATR, cm^–1^) 3240, 2929, 1607, 1539, 1489, 1447, 1342, 1237, 1147, 1076, 699
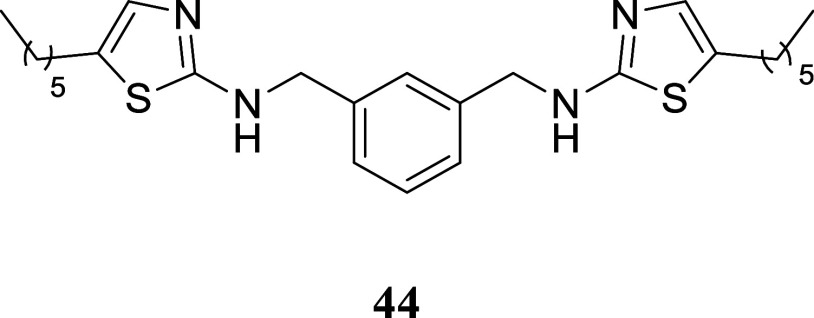
.

#### *N*,*N*′-(1,3-Phenylenebis­(methylene))­bis­(5-hexylthiazol-2-amine) (**44**)

It was obtained as a white crystal (244.5 mg, 52%), mp 78.2–76.9 °C. Column chromatography eluent, EtOAc/hexane gradient from 1:9 to 9:1. *R*_f_ 0.61 (3:2 v/v EtOAc/hexanes). ^1^H NMR (500 MHz, CDCl_3_) δ 7.36 (br s, 1H), 7.34–7.28 (m, 3H), 6.76 (t, *J* = 1.1 Hz, 2H), 5.31 (br s, 2H), 4.45 (s, 4H), 2.62 (td, *J* = 7.6, 1.1 Hz, 4H), 1.56 (tt, *J* = 7.4, 7.4 Hz, 4H), 1.37–1.24 (m, 12H), 0.87 (t, *J* = 6.9 Hz, 6H). ^13^C­{^1^H} NMR (125 MHz, CDCl_3_) δ 168.2, 138.5, 134.6, 129.1, 127.8, 126.9, 126.8, 49.5, 31.5, 31.3, 28.6, 27.1, 22.6, 14.0. HRMS (ESI) *m*/*z*: [M + H]^+^ calcd for C_26_H_39_N_4_S_2_ 471.2616; found 471.2645. IR (ATR, cm^–1^) 3181, 3088, 2952, 2924, 2852, 1594, 1573, 1552, 1456, 1336, 1163, 1153, 1110, 1091, 847, 710, 633, 512, 474
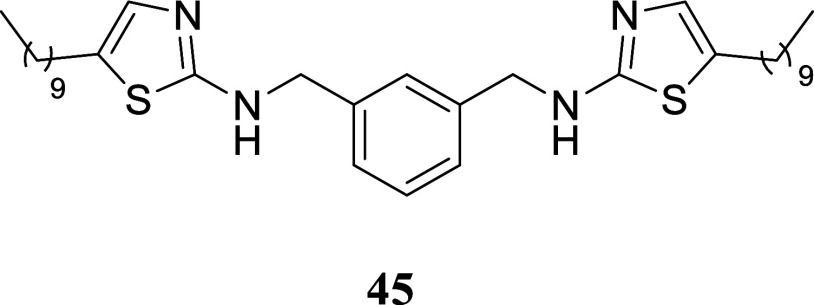
.

#### *N*,*N*′-(1,3-Phenylenebis­(methylene))­bis­(5-hexylthiazol-2-amine) (**45**)

It was obtained as an amorphous solid (116.5 mg, 20%). Column chromatography eluent, EtOAc/hexane gradient from 1:9 to 9:1. *R*_f_ 0.72 (3:2 v/v EtOAc/hexanes). ^1^H NMR (500 MHz, CDCl_3_) δ 7.38 (br s, 1H), 7.35–7.27 (m, 3H), 6.77 (t, *J* = 1.0 Hz, 2H), 5.57 (br s, 2H), 4.43 (s, 4H), 2.61 (td, *J* = 7.5, 1.0 Hz, 4H), 1.56 (tt, *J* = 7.6, 7.6 Hz, 4H), 1.37–1.19 (m, 28H), 0.88 (t, *J* = 7.0 Hz, 6H). ^13^C­{^1^H} NMR (125 MHz, CDCl_3_) δ 168.4, 138.4, 134.3, 129.1, 127.7, 127.0, 126.8, 49.6, 31.9, 31.3, 29.58, 29.55, 29.3, 29.0, 27.1, 22.7, 14.1. HRMS (ESI) *m*/*z*: [M + H]^+^ calcd for C_34_H_55_N_4_S_2_ 583.3868; found 583.3886. IR (ATR, cm^–1^) 3188, 2922, 2852, 1693, 1642, 1551, 1456, 1341, 1310, 1153, 711
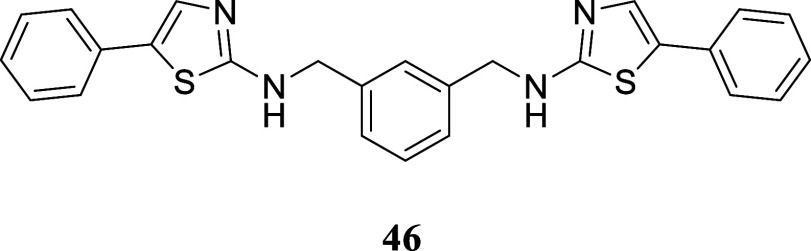
.

#### *N*,*N*′-(1,3-Phenylenebis­(methylene))­bis­(5-phenylthiazol-2-amine) (**46**)

It was obtained as a yellow crystal (163.5 mg, 36%), mp 183.8–185.6 °C. Column chromatography eluent, EtOAc/hexane gradient from 1:9 to 9:1. *R*_f_ 0.60 (3:2 v/v EtOAc/hexanes). ^1^H NMR (400 MHz, CDCl_3_) δ 7.43 (br s, 1H), 7.40–7.27 (m, 13H), 7.23–7.18 (m, 2H), 5.71 (br s, 2H), 4.53 (s, 4H). ^13^C­{^1^H} NMR (100 MHz, CDCl_3_) δ 169.0, 138.2, 134.4, 132.3, 129.2, 128.8, 128.6, 127.1, 126.8, 126.7, 125.3, 49.6. HRMS (ESI) *m*/*z*: [M + H]^+^ calcd for C_26_H_23_N_4_S_2_ 455.1364; found 455.1387. IR (ATR, cm^–1^) 3199, 2923, 1573, 1533, 1445, 1418, 1348, 1153, 1103, 970, 846, 788, 750, 686, 623, 478
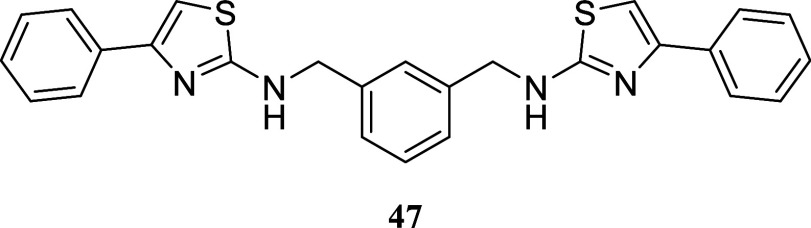
.

#### *N*,*N*′-(1,3-Phenylenebis­(methylene))­bis­(4-phenylthiazol-2-amine) (**47**)

It was obtained as a yellow oil (81.7 mg, 18%). Column chromatography eluent, EtOAc/hexane gradient from 1:9 to 9:1. *R*_f_ 0.89 (3:2 v/v EtOAc/hexanes). ^1^H NMR (500 MHz, CDCl_3_) δ 7.81–7.76 (m, 4H), 7.42 (br s, 1H), 7.38–7.32 (m, 7H), 7.29–7.25 (m, 2H), 6.68 (s, 2H), 5.72 (br s, 2H), 4.51 (d, *J* = 4.6 Hz, 4H). ^13^C­{^1^H} NMR (125 MHz, CDCl_3_) δ 169.2, 151.4, 138.3, 134.8, 129.1, 128.5, 127.6, 127.0, 126.8, 126.0, 101.1, 49.6. HRMS (ESI) *m*/*z*: [M + H]^+^ calcd for C_26_H_23_N_4_S_2_ 455.1364; found 455.1389. IR (ATR, cm^–1^) 3206, 2971, 1575, 1548, 1483, 1443, 1423, 1331, 1216, 771, 754, 702
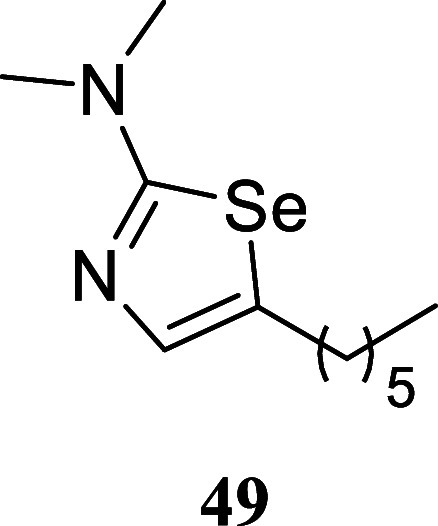
.

#### 5-Hexyl-*N*,*N*-dimethyl-1,3-selenazol-2-amine (**49**)

It was obtained as a yellow oil (135.2 mg, 52%). Column chromatography eluent, EtOAc/hexane gradient from 1:9 to 3:2. *R*_f_ 0.34 (1:4 v/v EtOAc/hexanes). ^1^H NMR (400 MHz, CDCl_3_) δ 6.77 (t, *J* = 1.1 Hz, 1H), 3.05 (s, 6H), 2.69 (td, *J* = 7.6, 1.1 Hz, 2H), 1.57 (tt, *J* = 7.4, 7.4 Hz, 2H), 1.39–1.21 (m, 6H), 0.87 (t, *J* = 7.1 Hz, 3H). ^13^C­{^1^H} NMR (100 MHz, CDCl_3_) δ 172.9, 136.4, 133.9, 41.0, 32.4, 31.6, 29.5, 28.6, 22.6, 14.1. HRMS (ESI) *m*/*z*: [M + H]^+^ calcd for C_11_H_21_N_2_Se 261.0870; found 261.0873. IR (ATR, cm^–1^) 2955, 2925, 2854, 1552, 1456, 1423, 1407, 1345, 1263, 1106, 1062, 920, 844, 591
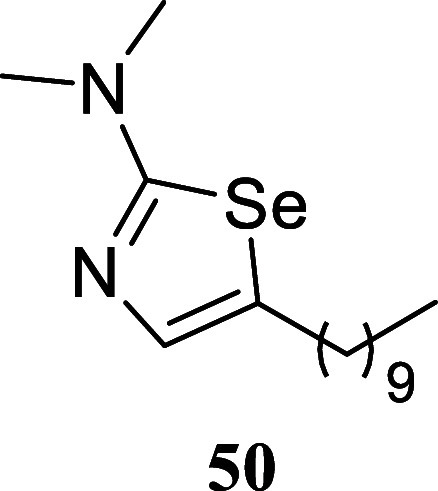
.

#### 5-Decyl-*N*,*N*-dimethyl-1,3-selenazol-2-amine (**50**)

It was obtained as a yellow oil (230.8 mg, 73%). Column chromatography eluent, EtOAc/hexane gradient from 1:9 to 3:2. *R*_f_ 0.43 (1:4 v/v EtOAc/hexanes). ^1^H NMR (500 MHz, CDCl_3_) δ 6.77 (t, *J* = 1.1 Hz, 1H), 3.05 (s, 6H), 2.69 (td, *J* = 7.4, 1.1 Hz, 2H), 1.57 (tt, *J* = 7.4, 7.4 Hz, 2H), 1.39–1.19 (m, 14H), 0.87 (t, *J* = 7.0 Hz, 3H). ^13^C­{^1^H} NMR (125 MHz, CDCl_3_) δ 172.9, 136.5, 133.9, 41.0, 32.5, 31.9, 29.58, 29.55, 29.5, 29.34, 29.30, 28.9, 22.7, 14.1. HRMS (ESI) *m*/*z*: [M + H]^+^ calcd for C_15_H_29_N_2_Se 317.1496; found 317.1481. IR (ATR, cm^–1^) 2923, 2853, 1555, 1457, 1423, 1346, 1263, 1107, 1062, 920, 845, 722, 591
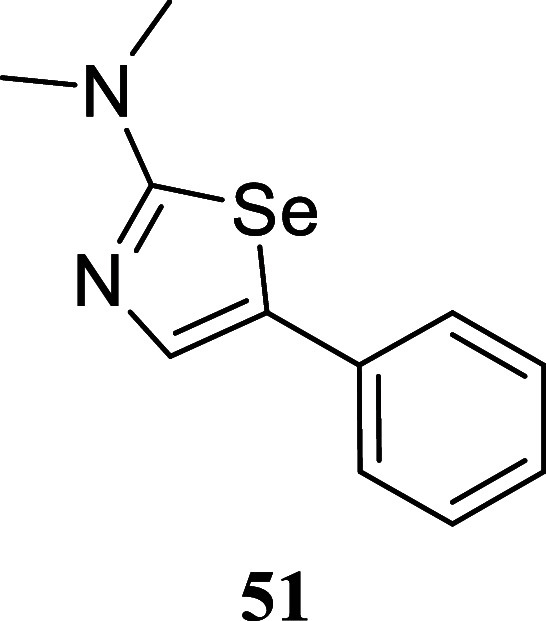
.

#### *N*,*N*-Dimethyl-5-phenyl-1,3-selenazol-2-amine (**51**)

It was obtained as an orange crystal (161.3 mg, 64%), mp 91.5–92.8 °C. Column chromatography eluent, EtOAc/hexane gradient from 1:9 to 3:2. *R*_f_ 0.25 (1:4 v/v EtOAc/hexanes). ^1^H NMR (400 MHz, CDCl_3_) δ 7.39–7.36 (m, 2H), 7.36 (s, 1H), 7.34–7.28 (m, 2H), 7.21–7.16 (m, 1H), 3.13 (s, 6H). ^13^C­{^1^H} NMR (100 MHz, CDCl_3_) δ 173.0, 136.5, 134.8, 132.1, 128.8, 126.4, 125.9, 41.0. HRMS (ESI) *m*/*z*: [M + H]^+^ calcd for C_11_H_13_N_2_Se 253.0244; found 253.0243. IR (ATR, cm^–1^) 2869, 2796, 1554, 1534, 1443, 1409, 1344, 1265, 1186, 1109, 915, 859, 756, 690, 652, 594, 473
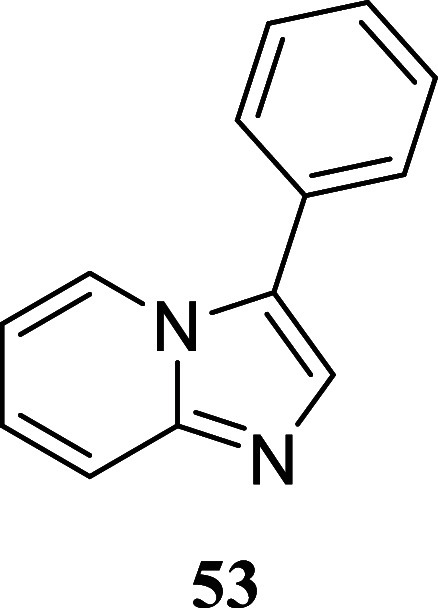
.

#### 3-Phenylimidazo­[1,2-*a*]­pyridine (**53**)^37^

It was obtained as a yellow oil (172.7 mg, 89%). Column chromatography eluent, EtOAc/hexane 3:2. *R*_f_ 0.25 (3:2 v/v EtOAc/hexanes). ^1^H NMR (500 MHz, CDCl_3_) δ 8.35 (ddd, *J* = 7.0, 1.2, 1.2 Hz, 1H), 7.70 (s, 1H), 7.68 (ddd, *J* = 9.1, 1.1, 1.1 Hz, 1H), 7.58–7.54 (m, 2H), 7.54–7.49 (m, 2H), 7.45–7.39 (m, 1H), 7.20 (ddd, *J* = 9.1, 6.7, 1.3 Hz, 1H), 6.82 (ddd, *J* = 6.8, 6.8, 1.2 Hz, 1H). ^13^C­{^1^H} NMR (125 MHz, CDCl_3_) δ 146.1, 132.5, 129.3, 129.2, 128.2, 128.1, 125.7, 124.2, 123.3, 118.3, 112.5. HRMS (ESI) *m*/*z*: [M + H]^+^ calcd for C_13_H_11_N_2_ 195.0922; found 195.0928. IR (ATR, cm^–1^) 3368, 3056, 1635, 1604, 1499, 1481, 1442, 1353, 1297, 1265, 1149, 1010, 964, 857, 750, 736, 696, 528
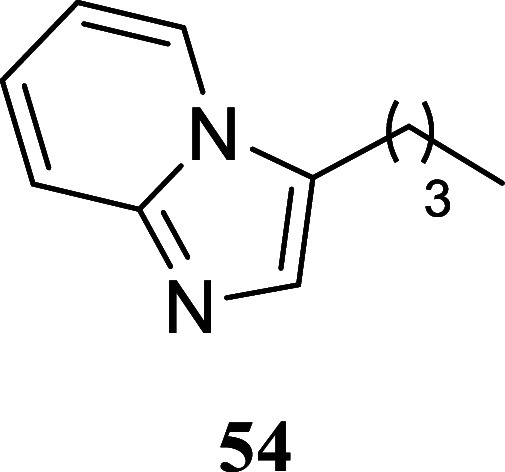
.

#### 3-Butylimidazo­[1,2-*a*]­pyridine (**54**)

It was obtained as a yellow oil (101.0 mg, 58%). Column chromatography eluent, EtOAc/hexanes 3:2. *R*_f_ 0.10 (3:2 v/v EtOAc/hexanes). ^1^H NMR (500 MHz, CDCl_3_) δ 7.91 (ddd, *J* = 6.9, 1.2, 1.2 Hz, 1H), 7.60 (ddd, *J* = 9.1, 1.2, 1.2 Hz, 1H), 7.41 (s, 1H), 7.14 (ddd, *J* = 9.1, 6.7, 1.3 Hz, 1H), 6.81 (ddd, *J* = 6.8, 6.8, 1.2 Hz, 1H), 2.83 (t, *J* = 7.5 Hz, 2H), 1.76 (tt, *J* = 7.5, 7.5 Hz, 2H), 1.47 (tq, *J* = 7.5, 7.5 Hz, 2H), 0.98 (t, *J* = 7.5 Hz, 3H). ^13^C­{^1^H} NMR (125 MHz, CDCl_3_) δ 145.3, 130.7, 124.5, 123.0, 122.9, 118.0, 111.8, 29.1, 23.6, 22.5, 13.8. HRMS (ESI) *m*/*z*: [M + H]^+^ calcd for C_11_H_14_N_2_ 175.1235; found 175.1236. IR (ATR, cm^–1^) 3339, 2956, 2930, 2851, 1635, 1502, 1442, 1359, 1313, 1144, 1131, 848, 750, 734
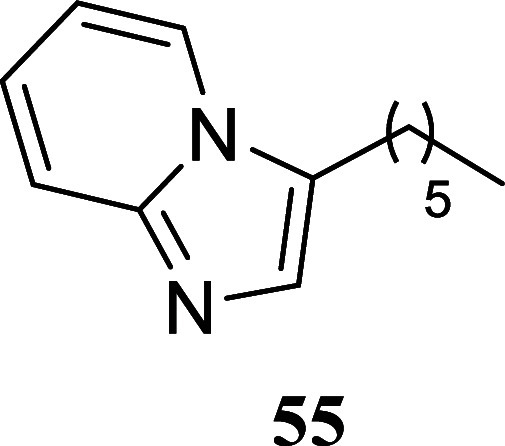
.

#### 3-Hexylimidazo­[1,2-*a*]­pyridine (**55**)^38^

It was obtained as a yellow oil (103.1 mg, 51%). Column chromatography eluent, EtOAc/hexanes 3:2. *R*_f_ 0.18 (3:2 v/v EtOAc/hexanes). ^1^H NMR (500 MHz, CDCl_3_) δ 7.87 (ddd, *J* = 6.9, 1.1, 1.1 Hz, 1H), 7.56 (ddd, *J* = 9.1, 1.1, 1.1 Hz, 1H), 7.38 (s, 1H), 7.09 (ddd, *J* = 9.1, 6.7, 1.2 Hz, 1H), 6.76 (ddd, *J* = 6.8, 6.8, 1.1 Hz, 1H), 2.78 (t, *J* = 7.5 Hz, 2H), 1.73 (tt, *J* = 7.5, 7.5 Hz, 2H), 1.45–1.36 (m, 2H), 1.35–1.25 (m, 4H), 0.88 (t, *J* = 7.1 Hz, 3H). ^13^C­{^1^H} NMR (125 MHz, CDCl_3_) δ 145.2, 130.6, 124.4, 122.9, 122.8, 117.8, 111.7, 31.5, 29.0, 26.8, 23.8, 22.5, 14.0. HRMS (ESI) *m*/*z*: [M + H]^+^ calcd for C_13_H_19_N_2_ 203.1548; found 203.1554. IR (ATR, cm^–1^) 3322, 2927, 2857, 1635, 1502, 1441, 1358, 1308, 1143, 1130, 848, 750, 734
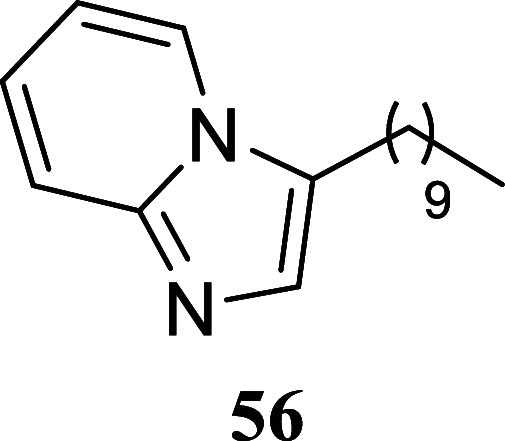
.

#### 3-Decylimidazo­[1,2-*a*]­pyridine (**56**)

It was obtained as a yellow oil (154.9 mg, 60%). Column chromatography eluent, EtOAc/hexanes 3:2. *R*_f_ 0.20 (3:2 v/v EtOAc/hexanes). ^1^H NMR (500 MHz, CDCl_3_) δ 7.87 (ddd, *J* = 6.9, 1.1, 1.1 Hz, 1H), 7.57 (ddd, *J* = 9.1, 1.1, 1.1 Hz, 1H), 7.38 (s, 1H), 7.10 (ddd, *J* = 9.1, 6.7, 1.3 Hz, 1H), 6.77 (ddd, *J* = 6.8, 6.8, 1.2 Hz, 1H), 2.78 (td, *J* = 7.7, 0.4 Hz, 2H), 1.74 (tt, *J* = 7.5, 7.5 Hz, 2H), 1.45–1.36 (m, 2H), 1.36–1.18 (m, 12H), 0.85 (t, *J* = 7.0 Hz, 3H). ^13^C­{^1^H} NMR (125 MHz, CDCl_3_) δ 145.2, 130.6, 124.4, 122.9, 122.8, 117.8, 111.7, 31.8, 29.48, 29.46, 29.4, 29.3, 29.2, 26.9, 23.8, 22.6, 14.0. HRMS (ESI) *m*/*z*: [M + H]^+^ calcd for C_17_H_27_N_2_ 259.2174; found 259.2191. IR (ATR, cm^–1^) 2923, 2853, 1635, 1502, 1466, 1358, 1310, 1143, 1130, 849, 749, 733
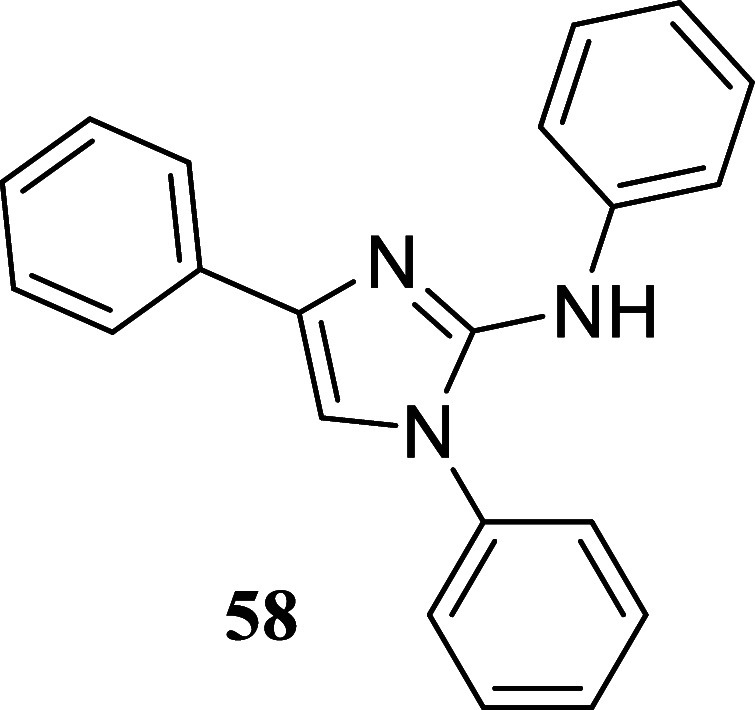
.

#### *N*,1,4-Triphenyl-1*H*-imidazol-2-amine (**58**)

It was obtained as an orange crystal (155.6 mg, 50%), mp 165.7–167.5 °C. Column chromatography eluent, EtOAc/hexane gradient from 1:9 to 3:2. *R*_f_ 0.86 (3:2 v/v EtOAc/hexanes). ^1^H NMR (500 MHz, CDCl_3_) δ 7.53–7.44 (m, 3H), 7.43–7.38 (m, 2H), 7.30–7.26 (m, 4H), 7.21–7.12 (m, 3H), 7.10 (s, 1H), 7.08–7.04 (m, 2H), 6.96–6.91 (m, 1H), 5.89 (s, 1H). ^13^C­{^1^H} NMR (125 MHz, CDCl_3_) δ 145.5, 140.6, 135.4, 130.2, 130.0, 129.4, 129.1, 129.0, 128.3, 128.1, 127.0, 126.6, 124.5, 121.2, 116.9. HRMS (ESI) *m*/*z*: [M + H]^+^ calcd for C_21_H_18_N_3_ 312.1501; found 312.1514. IR (ATR, cm^–1^) 3412, 3061, 1640, 1612, 1587, 1571, 1511, 1484, 1443, 1312, 1249, 690, 751, 555, 505
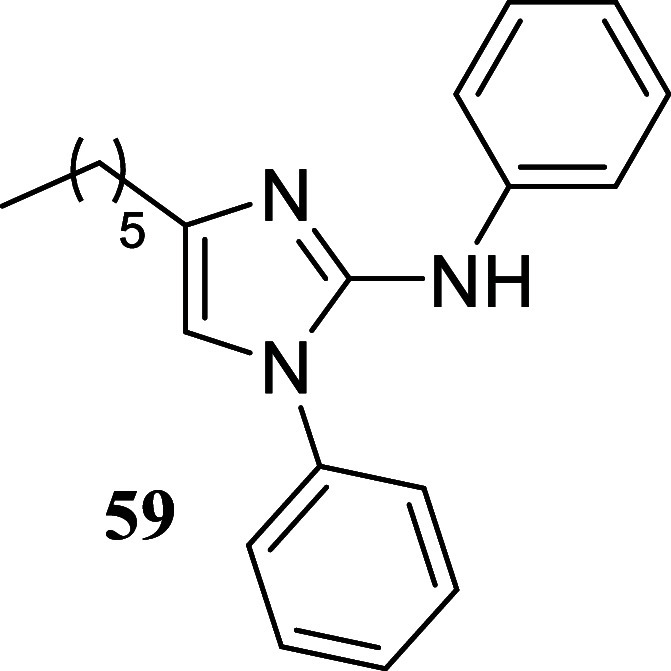
.

#### 4-Hexyl-*N*,1-diphenyl-1*H*-imidazol-2-amine (**59**)

It was obtained as a yellow oil (115.0 mg, 36%). Column chromatography eluent, EtOAc/hexane gradient from 1:9 to 3:2. *R*_f_ 0.69 (3:2 v/v EtOAc/hexanes). ^1^H NMR (400 MHz, CDCl_3_) δ 7.58–7.47 (m, 3H), 7.33–7.28 (m, 4H), 7.25–7.19 (m, 2H), 6.91–6.85 (m, 1H), 6.67 (t, *J* = 1.0 Hz, 1H), 5.61 (s, 1H), 2.32 (td, *J* = 7.7, 1.0 Hz, 2H), 1.42 (tt, *J* = 7.3, 7.3 Hz, 2H), 1.29–1.15 (m, 6H), 0.84 (t, *J* = 7.0 Hz, 3H). ^13^C­{^1^H} NMR (125 MHz, CDCl_3_) δ 143.7, 141.3, 135.1, 130.1, 129.2, 129.04, 128.99, 128.1, 121.7, 120.7, 116.4, 31.4, 28.8, 28.0, 24.7, 22.5, 14.0. HRMS (ESI) *m*/*z*: [M + H]^+^ calcd for C_21_H_26_N_3_ 320.2127; found 320.2149. IR (ATR, cm^–1^) 3061, 2928, 2858, 1655, 1590, 1552, 1497, 1451, 1406, 750, 694
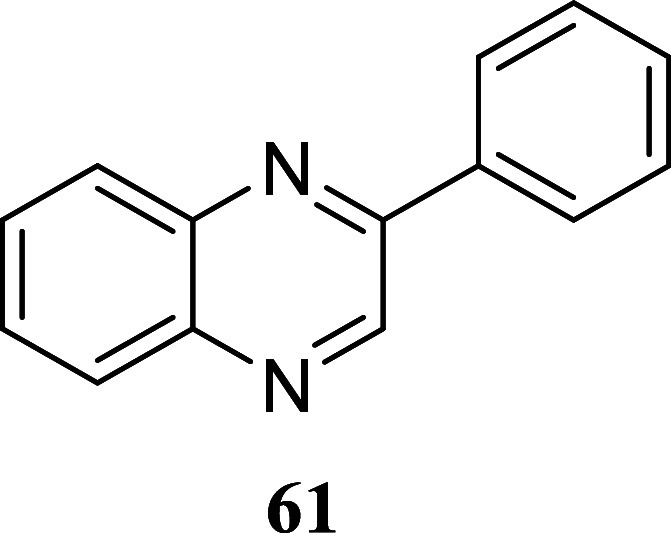
.

#### 2-Phenylquinoxaline (**61**)^39^

It was obtained as a brown crystal (127.9 mg, 62%), mp 63.0–64.7 °C. Column chromatography eluent, EtOAc/hexane gradient from 1:9 to 3:2. *R*_f_ 0.91 (3:2 v/v EtOAc/hexanes). ^1^H NMR (400 MHz, CDCl_3_) δ 9.32 (s, 1H), 8.23–8.09 (m, 4H), 7.81–7.71 (m, 2H), 7.60–7.49 (m, 3H). ^13^C­{^1^H} NMR (100 MHz, CDCl_3_) δ 151.8, 143.3, 142.3, 141.5, 136.7, 130.2, 130.1, 129.6, 129.5, 129.10, 129.07, 127.5. HRMS (ESI) *m*/*z*: [M + H]^+^ calcd for C_14_H_11_N_2_ 207.0922; found 207.0927. IR (ATR, cm^–1^) 3060, 1549, 1538, 1489, 1445, 1376, 1316, 1307, 1126, 1048, 1028, 1020, 957, 798, 771, 761, 751, 688, 671, 608, 558, 487
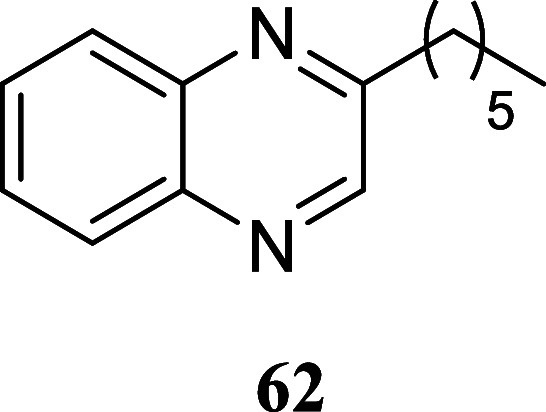
.

#### 2-Hexylquinoxaline (**62**)^40^

It was obtained as a yellow oil (66.4 mg, 31%). Column chromatography eluent, EtOAc/hexane gradient from 1:9 to 3:2. *R*_f_ 0.76 (1:4 v/v EtOAc/hexanes). ^1^H NMR (500 MHz, CDCl_3_) δ 8.74 (s, 1H), 8.10–8.02 (m, 2H), 7.77–7.67 (m, 2H), 3.02 (t, *J* = 7.5 Hz, 2H), 1.89–1.80 (m, 2H), 1.44 (tt, *J* = 7.5, 7.5 Hz, 2H), 1.38–1.28 (m, 4H), 0.89 (t, *J* = 7.1 Hz, 3H). ^13^C­{^1^H} NMR (125 MHz, CDCl_3_) δ 157.7, 145.9, 142.2, 141.2, 129.9, 129.2, 128.88, 128.86, 36.6, 31.6, 29.5, 29.1, 22.5, 14.0. HRMS (ESI) *m*/*z*: [M + H]^+^ calcd for C_14_H_19_N_2_ 215.1548; found 215.1555. IR (ATR, cm^–1^) 2954, 2926, 2856, 1559, 1491, 1466, 1410, 1366, 1283, 1176, 1123, 1013, 972, 921, 759, 610
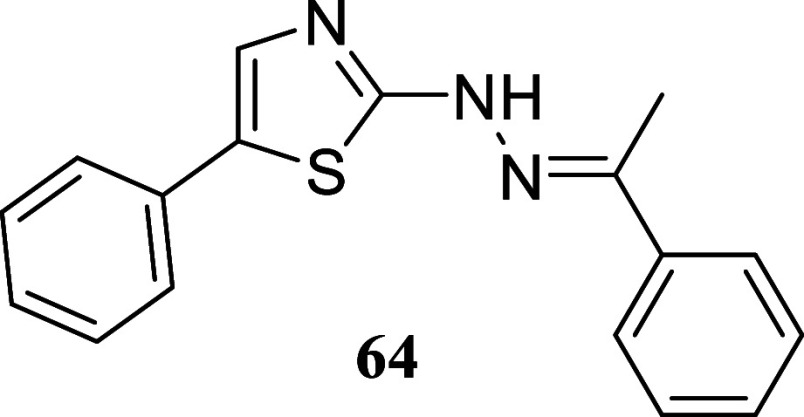
.

#### (*E*)-5-Phenyl-2-(2-(1-phenylethylidene)­hydrazineyl)­thiazole (**64**)

It was obtained as an amorphous solid (14.7 mg, 5%). Column chromatography eluent, EtOAc/hexane gradient from 1:9 to 3:2. *R*_f_ 0.43 (1:4 v/v EtOAc/hexanes). ^1^H NMR (500 MHz, CDCl_3_) δ 7.83–7.79 (m, 2H), 7.53–7.50 (m, 2H), 7.50 (s, 1H), 7.44–7.35 (m, 5H), 7.28–7.23 (m, 1H), 2.30 (s, 3H). ^13^C­{^1^H} NMR (125 MHz, CDCl_3_) δ 168.9, 146.0, 137.7, 134.3, 132.4, 129.6, 129.0, 128.9, 128.5, 127.0, 125.9, 125.5, 13.1. HRMS (ESI) *m*/*z*: [M + H]^+^ calcd for C_17_H_16_N_3_S 294.1065; found 294.1089. IR (ATR, cm^–1^) 3288, 3195, 3070, 1643, 1514, 1477, 1444, 1280, 1203, 1096, 927, 854, 753, 685, 547
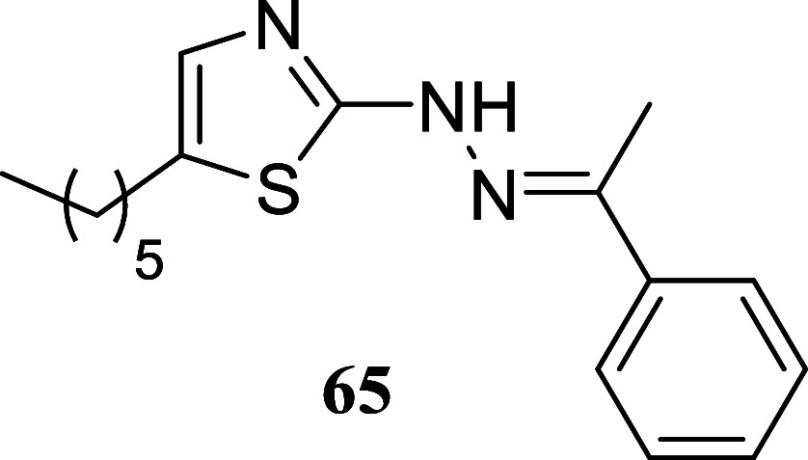
.

#### (*E*)-5-Hexyl-2-(2-(1-phenylethylidene)­hydrazineyl)­thiazole (**65**)

It was obtained as an amorphous solid (60.2 mg, 20%). Column chromatography eluent, EtOAc/hexane gradient from 1:9 to 3:2. *R*_f_ 0.14 (1:4 v/v EtOAc/hexanes). ^1^H NMR (500 MHz, CDCl_3_) δ 9.09 (br s, 1H), 7.81–7.76 (m, 2H), 7.43–7.31 (m, 3H), 6.91 (t, *J* = 1.1 Hz, 1H), 2.69 (td, *J* = 7.5, 1.1 Hz, 2H), 2.26 (s, 3H), 1.63 (tt, *J* = 7.5, 7.5 Hz, 2H), 1.41–1.25 (m, 6H), 0.89 (t, *J* = 7.0 Hz, 3H). ^13^C­{^1^H} NMR (125 MHz, CDCl_3_) δ 168.4, 145.1, 137.9, 134.3, 130.0, 128.7, 128.4, 125.8, 31.5, 31.3, 28.6, 27.2, 22.6, 14.1, 13.0. HRMS (ESI) *m*/*z*: [M + H]^+^ calcd for C_17_H_24_N_3_S 302.1691; found 302.1706. IR (ATR, cm^–1^) 3150, 3062, 2952, 2927, 2855, 1556, 1494, 1465, 1431, 1369, 1345, 1294, 1257, 1157, 1118, 1100, 1067, 1027, 815, 756, 715, 686, 634, 617, 552, 519.

## Supplementary Material



## Data Availability

The data underlying this study are available in the published article and its Supporting Information.

## Notes

Mw methods and copies of NMR spectra for all compounds (PDF)
