# Supplementary material for: Microwave-Assisted One-Pot Telescoped Synthesis of 2‑Amino-1,3-thiazoles, Selenazoles, Imidazo[1,2‑a]pyridines, and Other Heterocycles from Alcohols
Source: J Org Chem. 2024 Mar 18;89(7):4628–46. doi: 10.1021/acs.joc.3c02903 (PMC12863238; doi:10.1021/acs.joc.3c02903)
Supplement: Supplementary file 1 [file jo3c02903_si_001.pdf]

Supporting Information

Microwave-Assisted One-Pot Telescoped Synthesis of 2-Amino-1,3-Thiazoles, Selenazoles, Imidazo[1,2-a]pyridines and Other Heterocycles from Alcohols.

Pablo Macías-Benítez, Alfonso Sierra-Padilla, Francisco M. Guerra,* and F. Javier Moreno-Dorado

Department of Organic Chemistry and Instituto de Biomoléculas, University of Cádiz, 11510 Puerto Real, Cádiz, Spain.

*E-mail: francisco.guerra@uca.es

Table of Contents

1. MW Methods………………………………………………………………….....S2

2. NMR Spectra………………………………………………………………....S3

1. MW Methods.

Table S1. **Method A (Optimal Conditions – Step 1)**

Nº	t	T_1 – vessel (ºC)	T_2 – system (ºC)	P (bar)	E (W)
1	00:00:10	60	50	35.0	1500
2	00:01:00	60	50	45.0	1500
3	00:09:00	60	50	45.0	1500

Table S2. **Method B (Optimal Conditions – Step 2)**

Nº	t	T_1 – vessel (ºC)	T_2 – system (ºC)	P (bar)	E (W)
1	00:00:10	100	50	35.0	1500
2	00:01:00	100	50	45.0	1500
3	00:14:00	100	50	45.0	1500

Table S3. **Method C**

Nº	t	T_1 – vessel (ºC)	T_2 – system (ºC)	P (bar)	E (W)
1	00:00:10	60	50	35.0	1500
2	00:01:00	60	50	45.0	1500
3	00:09:00	60	50	45.0	1500
4	00:00:01	100	50	45.0	1500
5	00:15:00	100	50	45.0	1500

Table S4. **Method D**

Nº	t	T_1 – vessel (ºC)	T_2 – system (ºC)	P (bar)	E (W)
1	00:00:10	70	50	35.0	1500
2	00:01:00	70	50	45.0	1500
3	00:14:00	70	50	45.0	1500
4	00:00:01	100	50	45.0	1500
5	00:15:00	100	50	45.0	1500

Table S5. **Method E**

Nº	t	T_1 – vessel (ºC)	T_2 – system (ºC)	P (bar)	E (W)
1	00:00:10	70	50	35.0	1500
2	00:01:00	70	50	45.0	1500
3	00:14:00	70	50	45.0	1500

Table S6. **Method F**

Nº	t	T_1 – vessel (ºC)	T_2 – system (ºC)	P (bar)	E (W)
1	00:00:10	100	50	35.0	1500
2	00:01:00	100	50	45.0	1500
3	00:29:00	100	50	45.0	1500

2. NMR Spectra

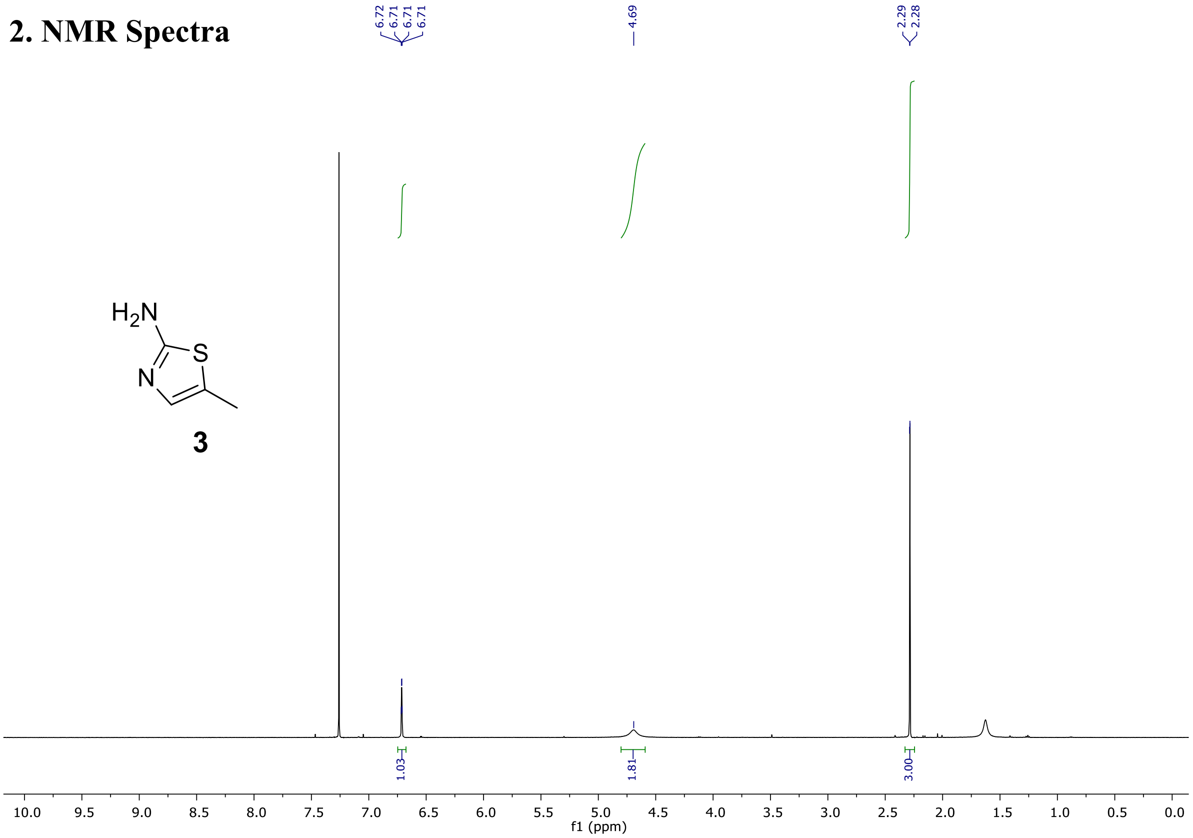

Figure S1. ^{1}H Spectrum of **3** in $CDCl_3$ (500 MHz)

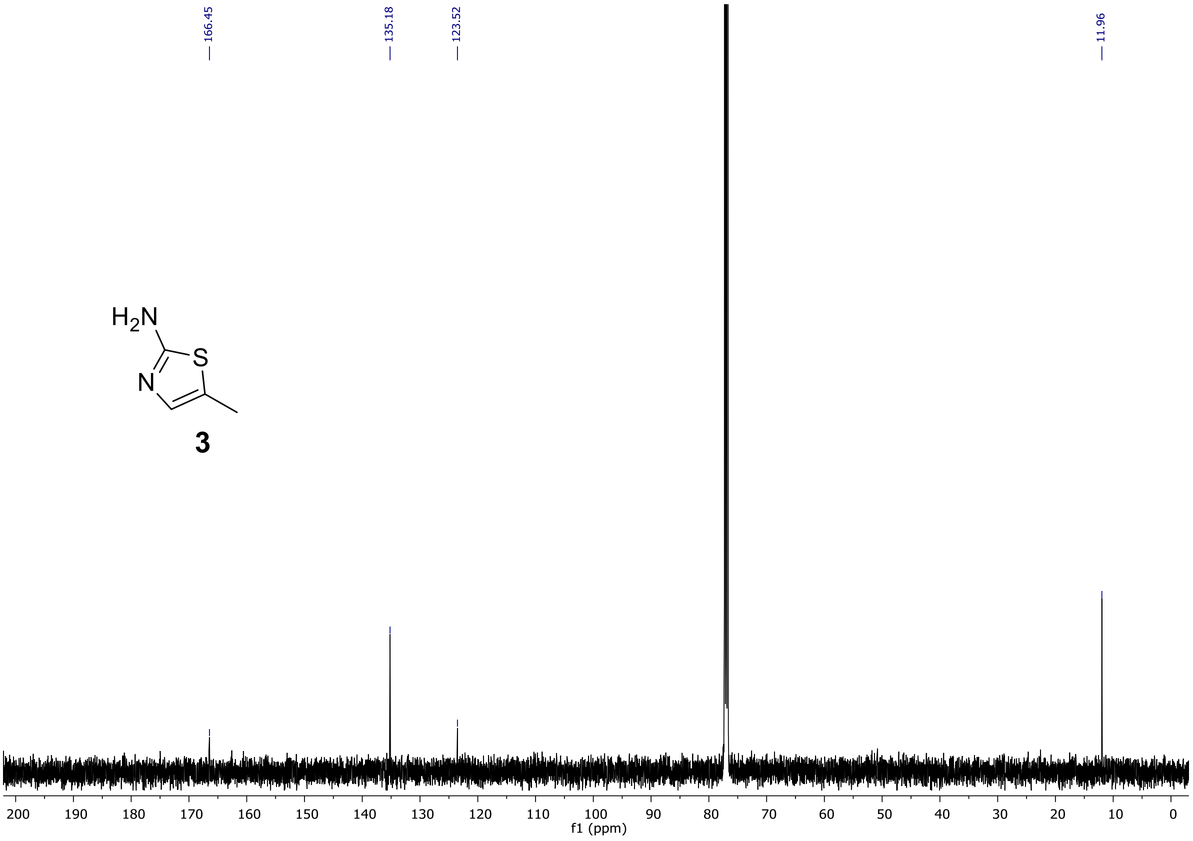

Figure S2. $^{13}C\{^{1}H\}$ Spectrum of **3** in $CDCl_3$ (125 MHz)

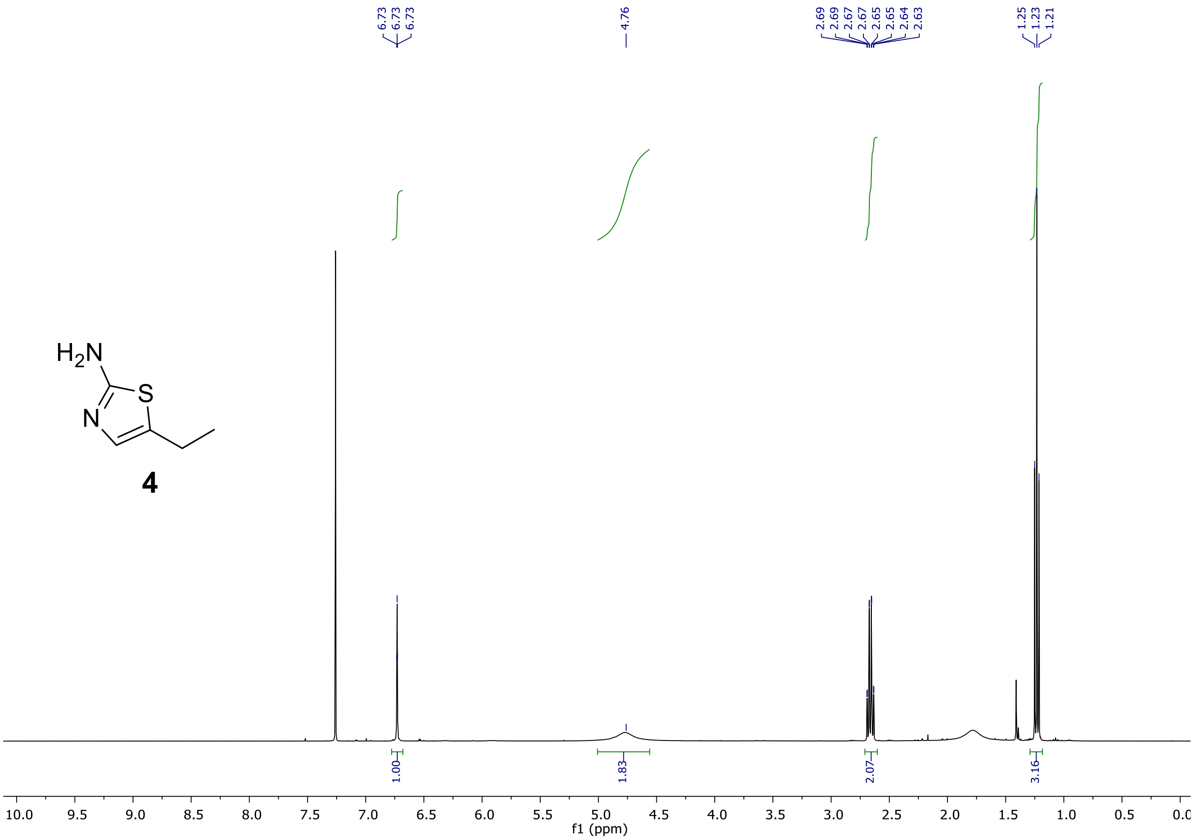

Figure S3. ^{1}H Spectrum of **4** in $CDCl_3$ (400 MHz)

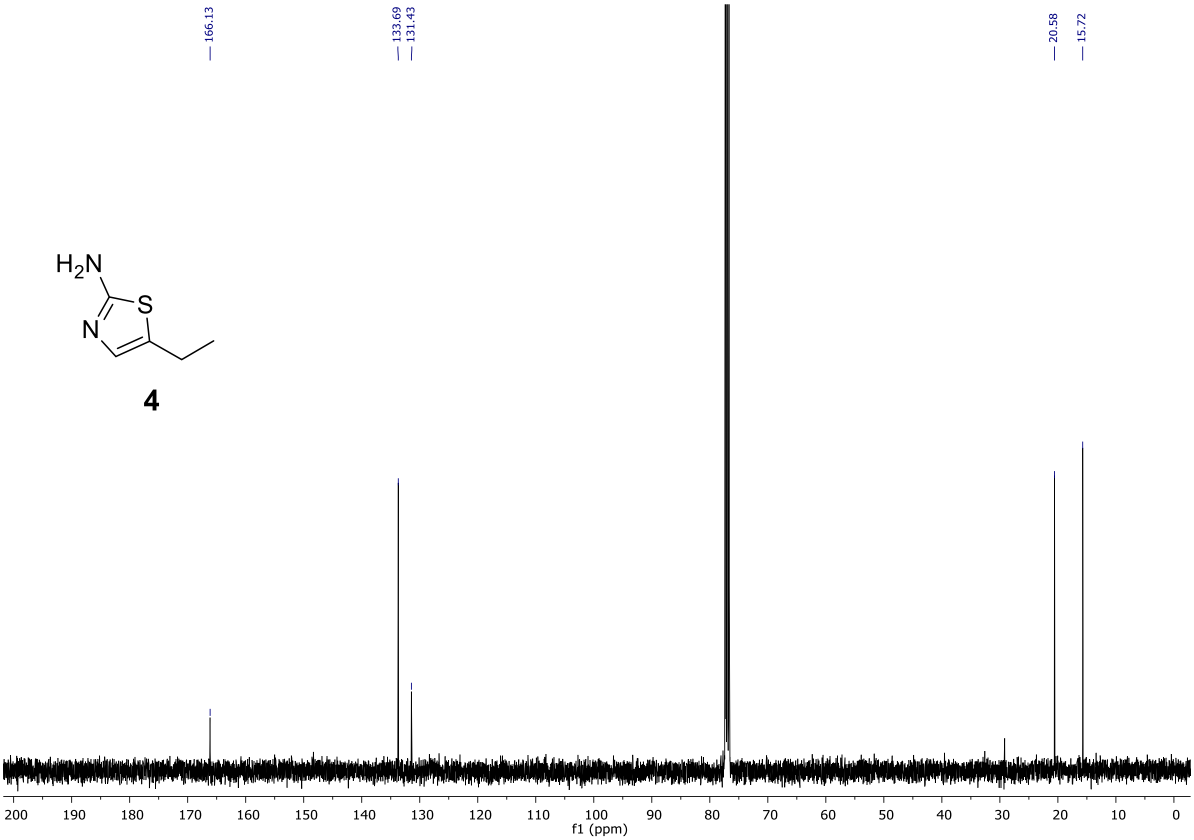

Figure S4. $^{13}C\{^{1}H\}$ Spectrum of **4** in $CDCl_3$ (100 MHz)

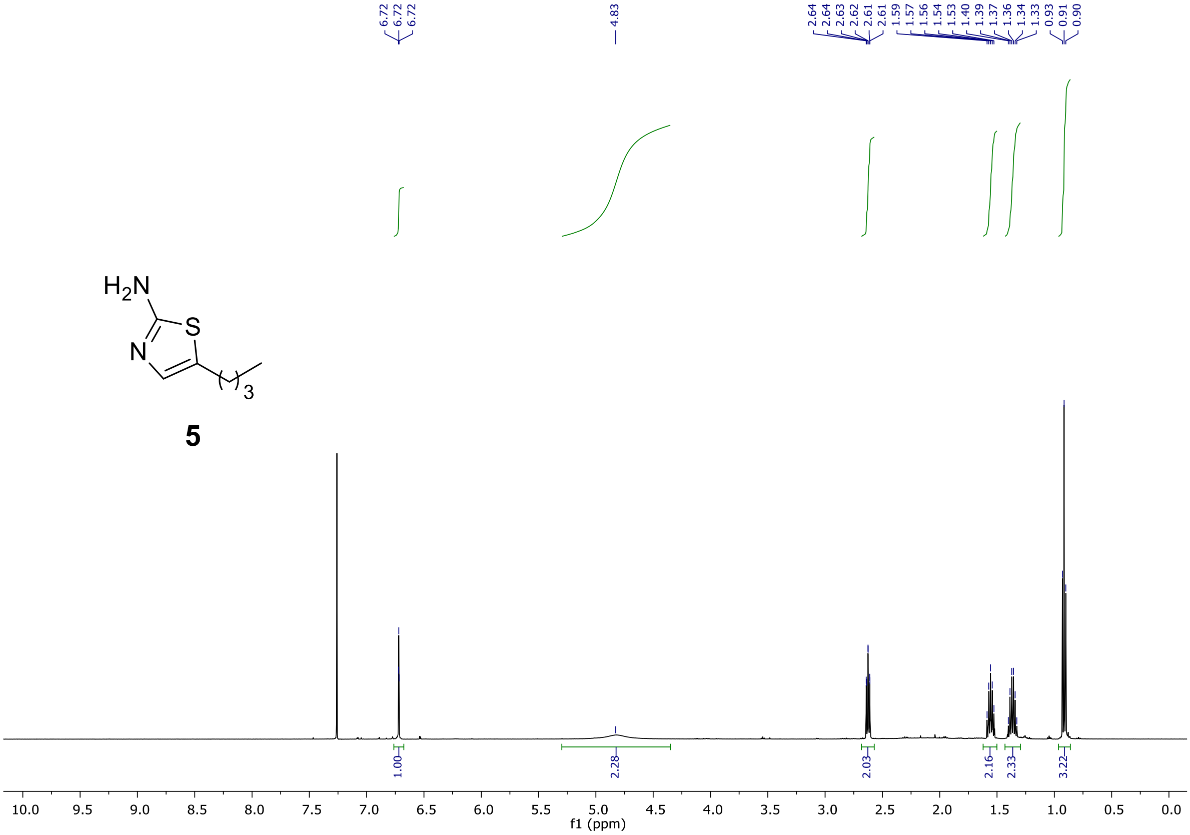

Figure S5. ^{1}H Spectrum of **5** in $CDCl_3$ (500 MHz)

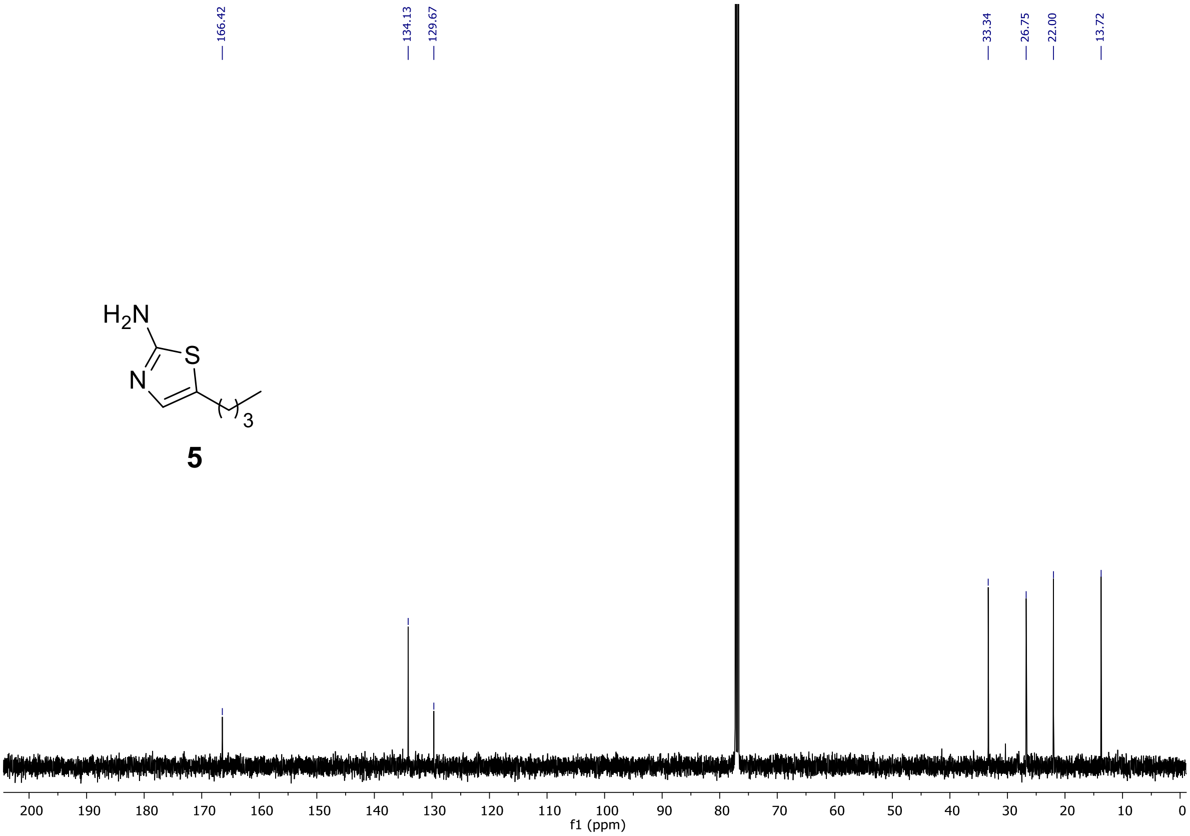

Figure S6. $^{13}C\{^{1}H\}$ Spectrum of **5** in $CDCl_3$ (125 MHz)

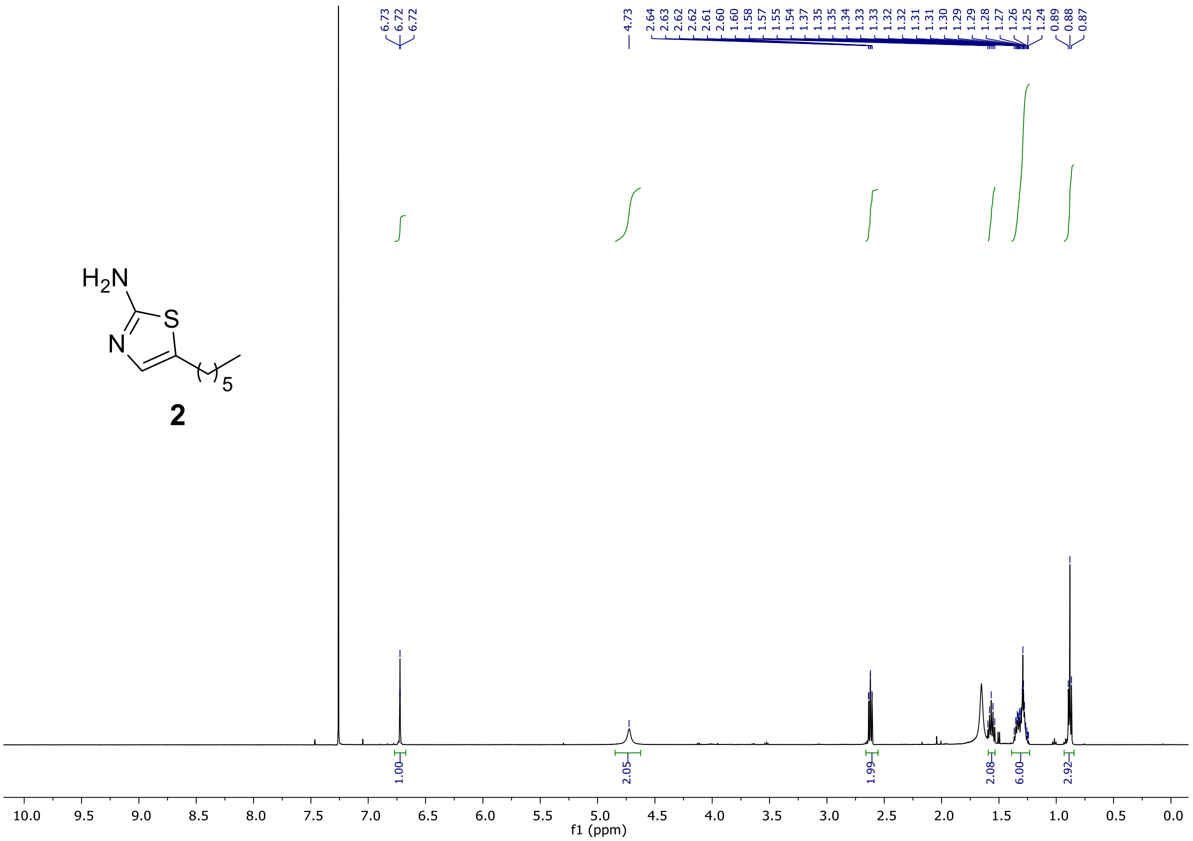

Figure S7. ^{1}H Spectrum of **2** in $CDCl_3$ (500 MHz)

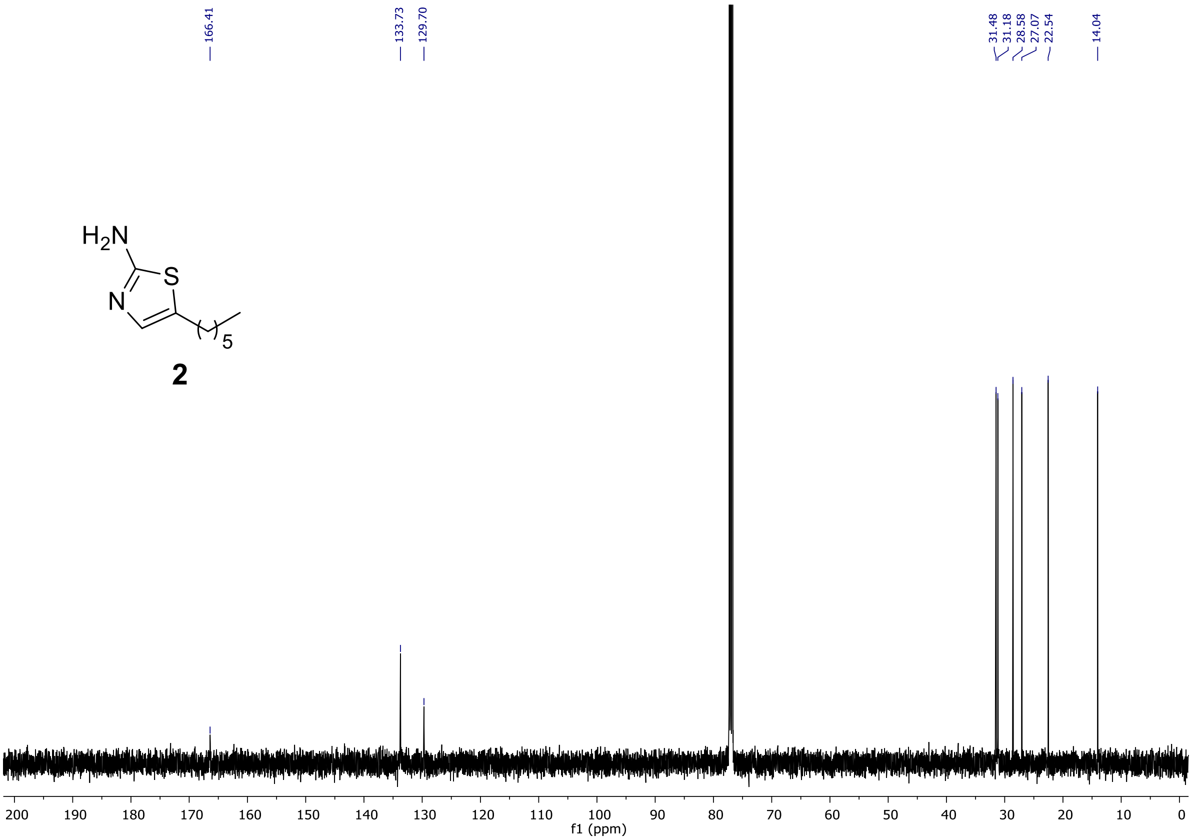

Figure S8. $^{13}C\{^{1}H\}$ Spectrum of **2** in $CDCl_3$ (125 MHz)

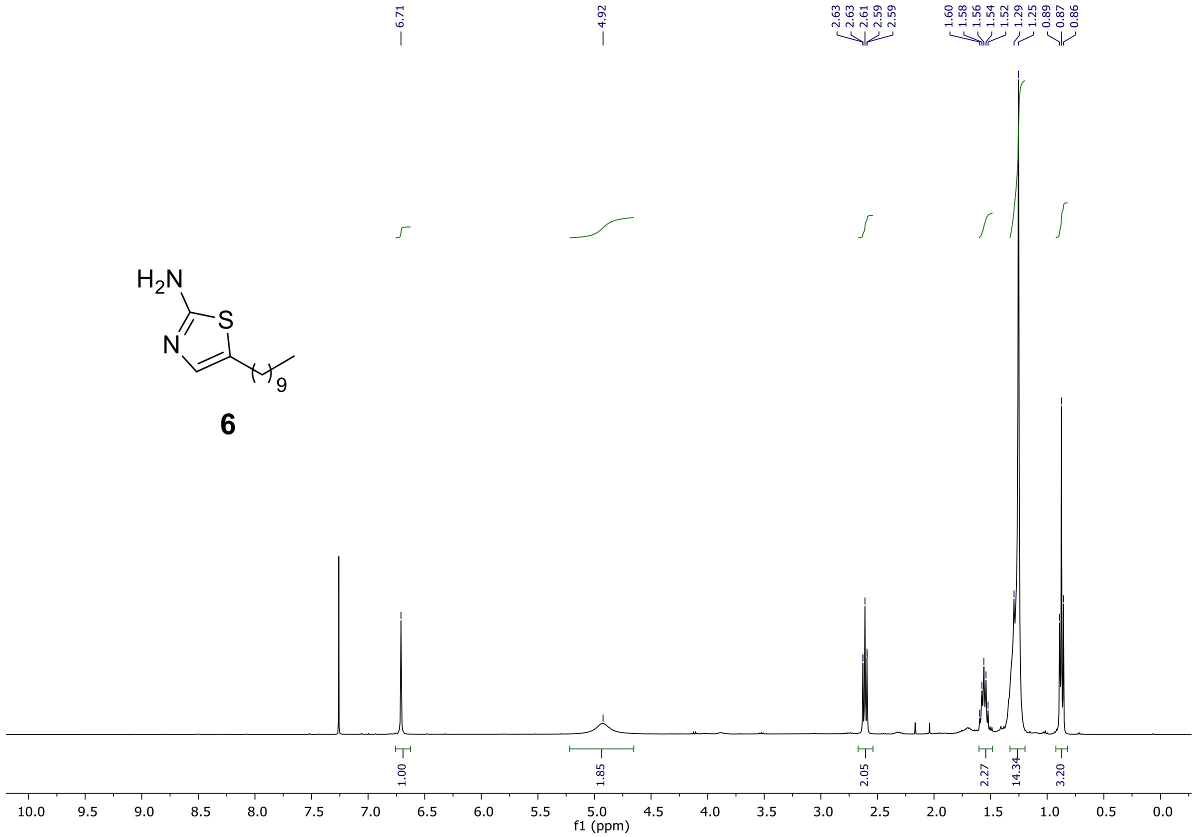

Figure S9. ^{1}H Spectrum of **6** in $CDCl_3$ (400 MHz)

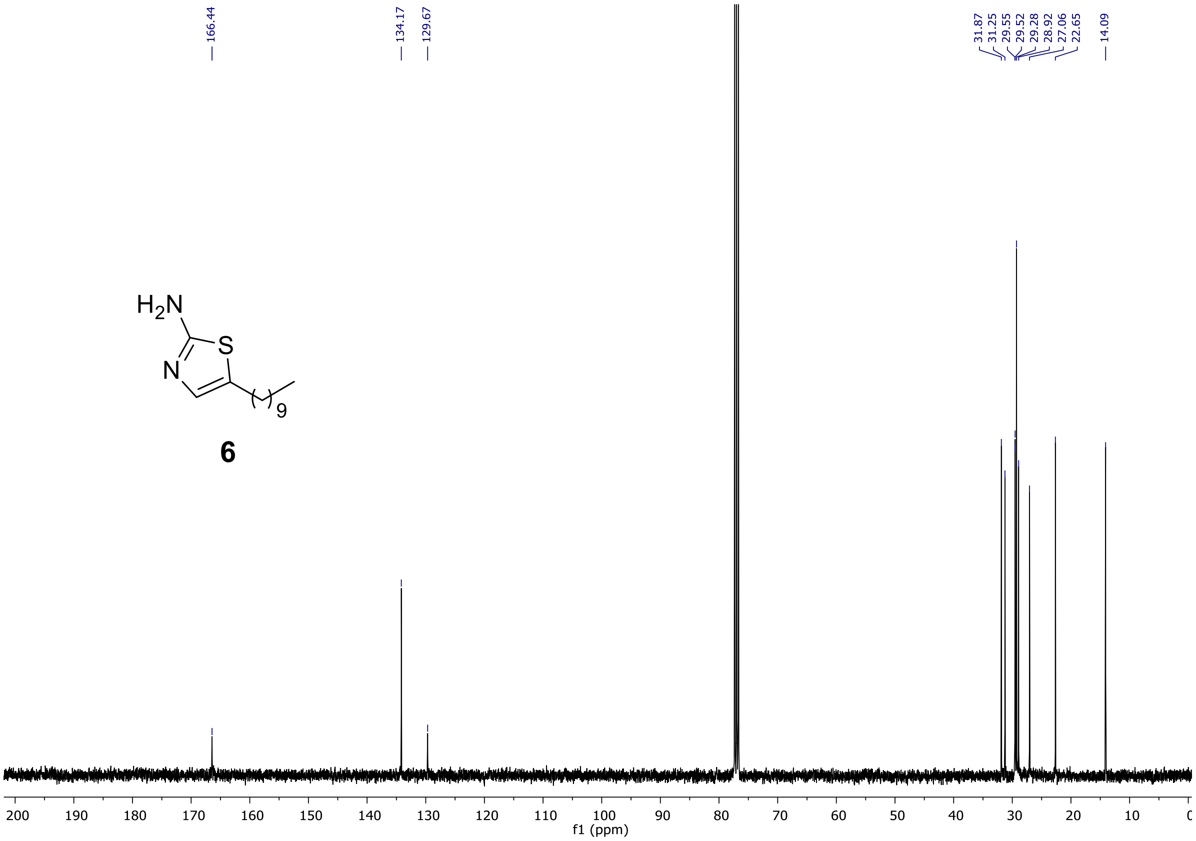

Figure S10. $^{13}C\{^{1}H\}$ Spectrum of **6** in $CDCl_3$ (100 MHz)

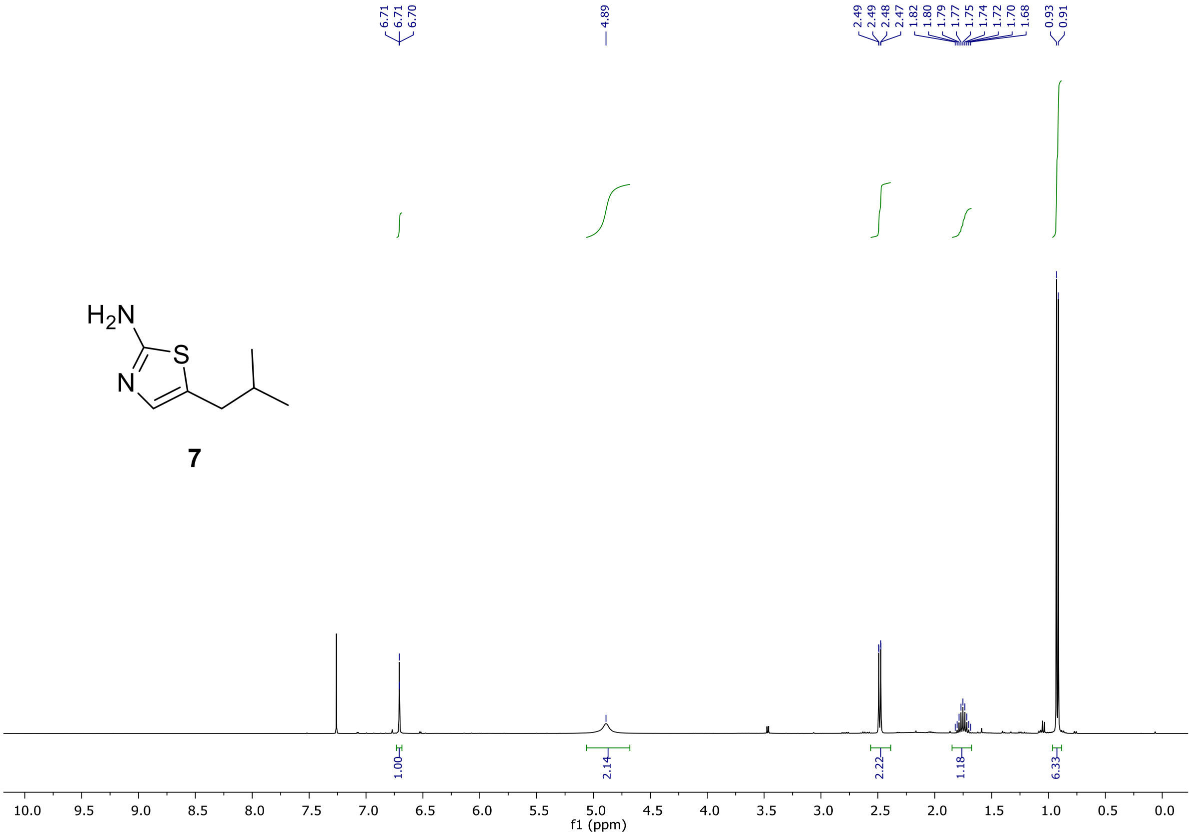

Figure S11. ^{1}H Spectrum of **7** in $CDCl_3$ (400 MHz)

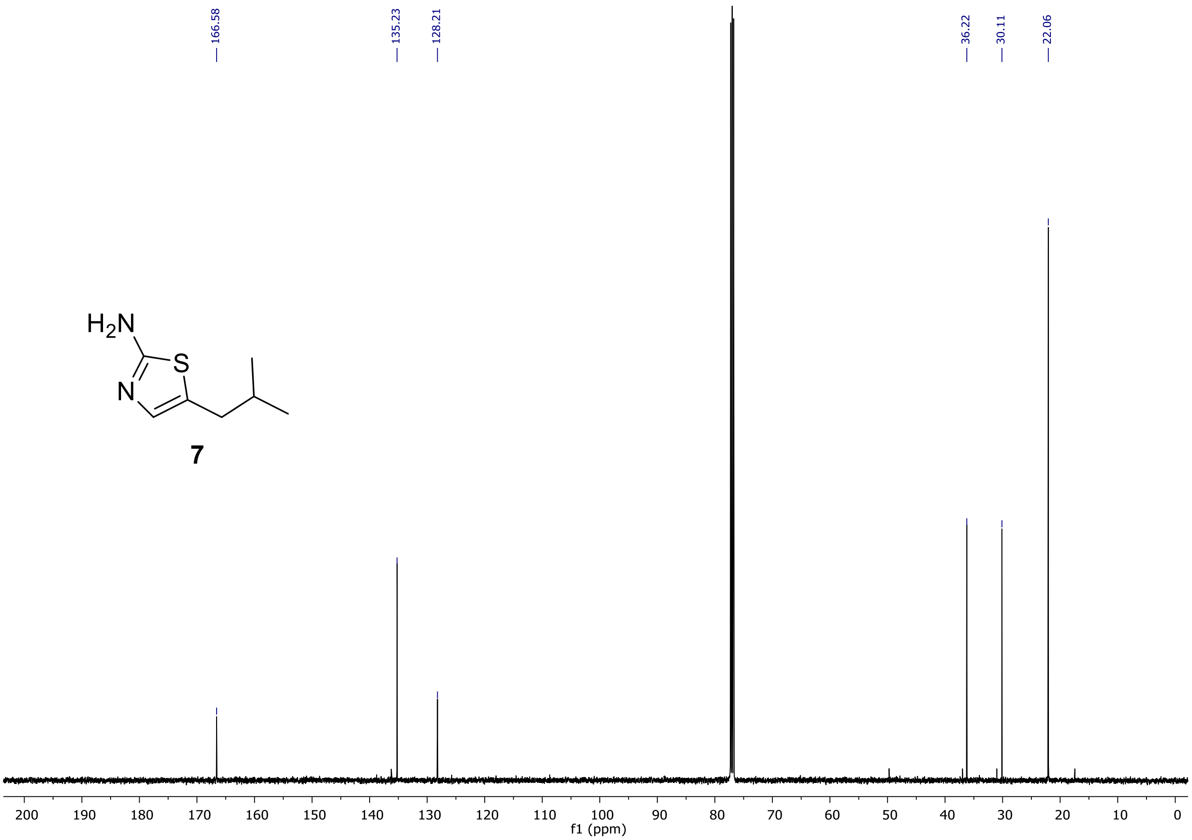

Figure S12. $^{13}C\{^{1}H\}$ Spectrum of **7** in $CDCl_3$ (125 MHz)

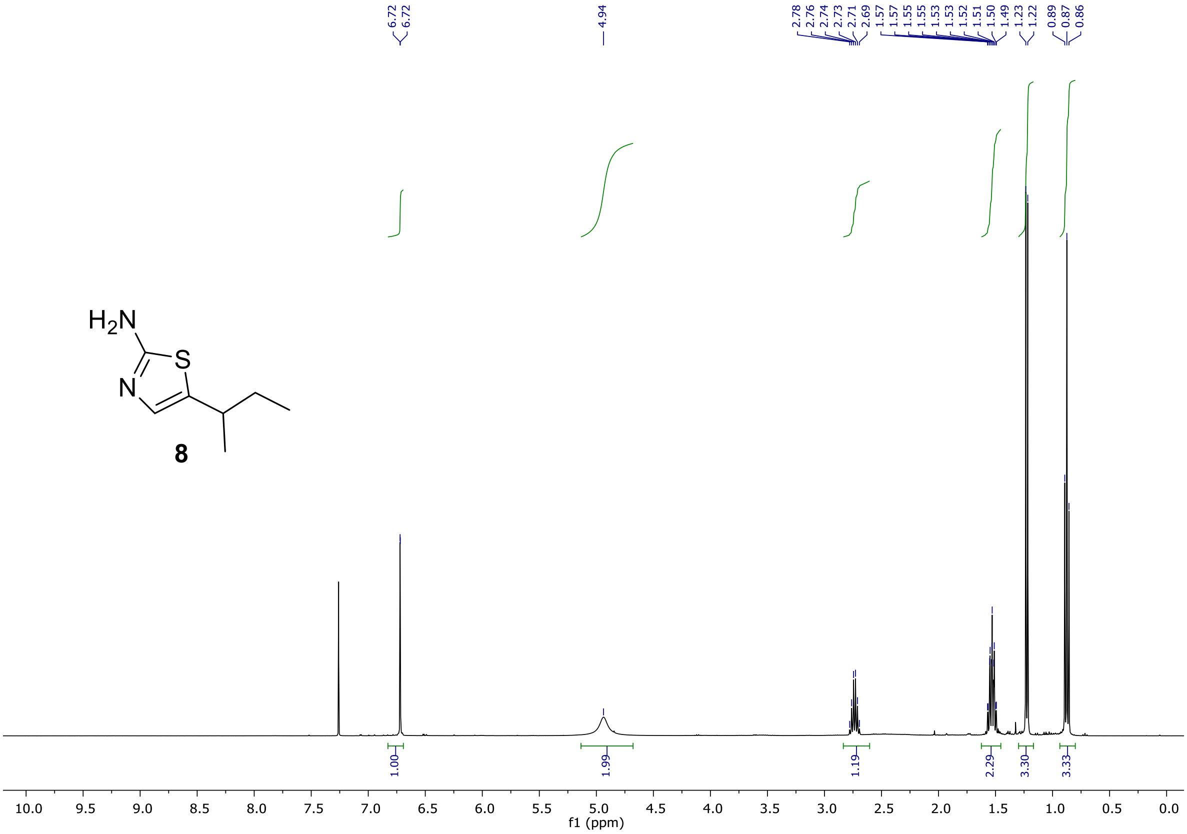

Figure S13. ^{1}H Spectrum of **8** in $CDCl_3$ (400 MHz)

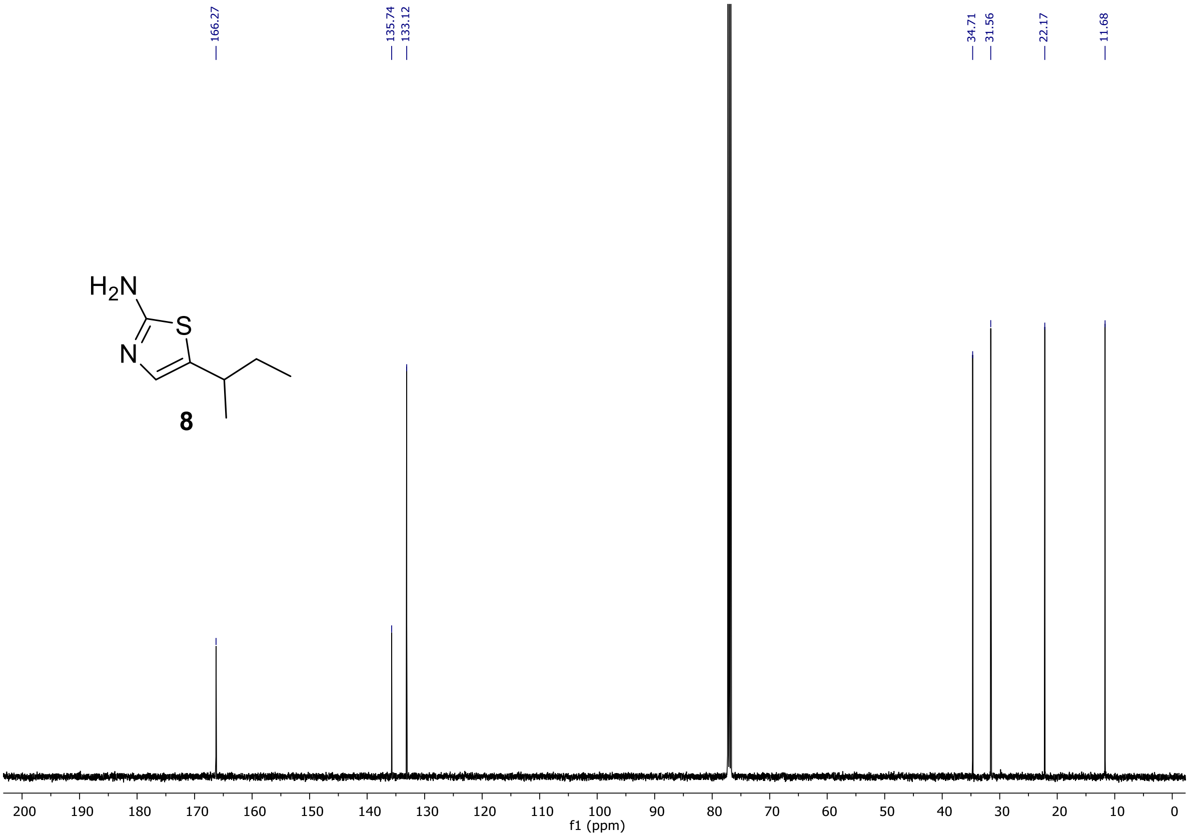

Figure S14. $^{13}C\{^{1}H\}$ Spectrum of **8** in $CDCl_3$ (125 MHz)

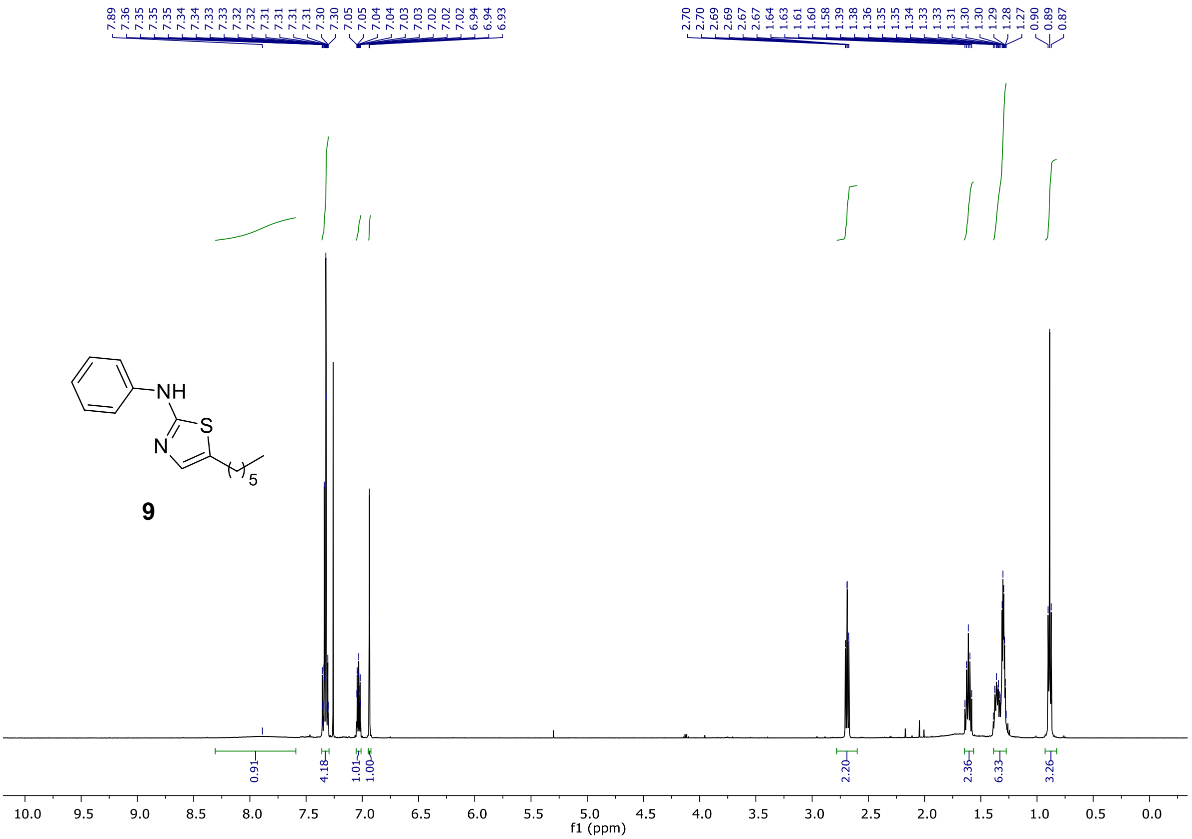

Figure S15. ^{1}H Spectrum of **9** in $CDCl_3$ (500 MHz)

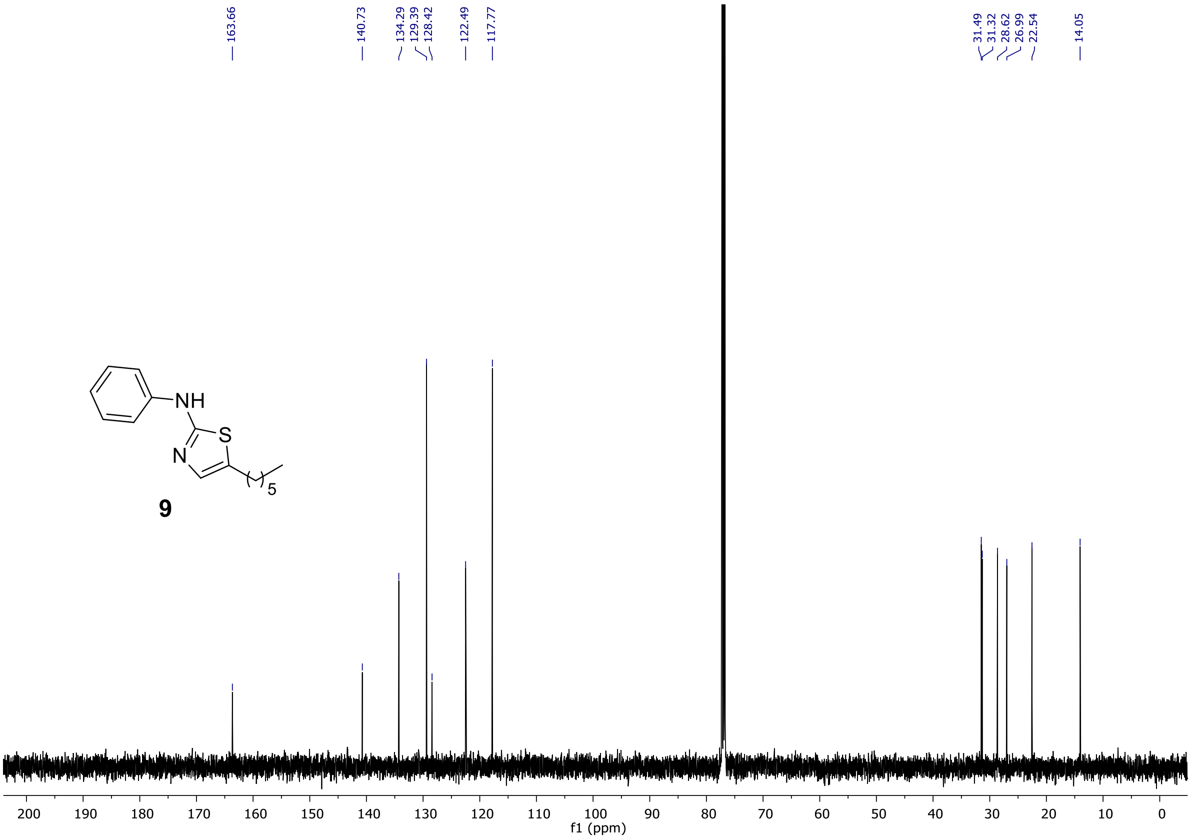

Figure S16. $^{13}C\{^{1}H\}$ Spectrum of **9** in $CDCl_3$ (125 MHz)

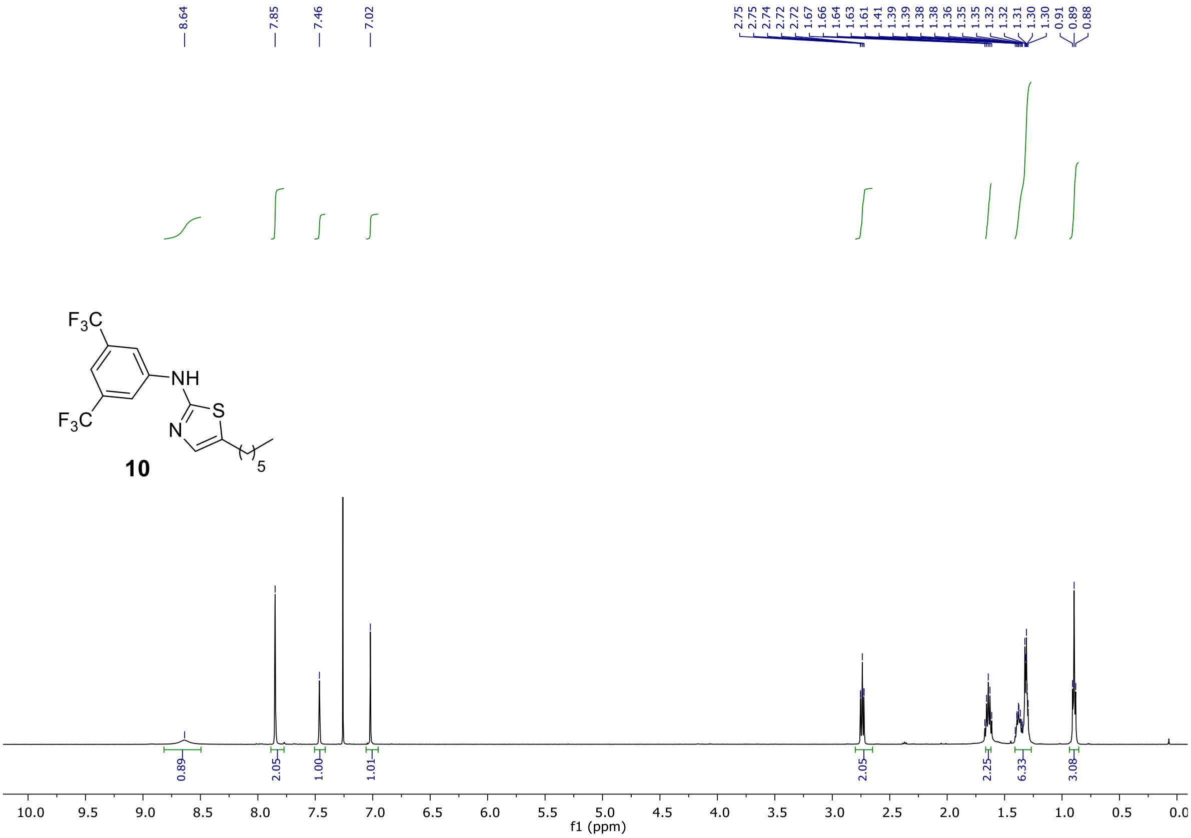

Figure S17. ^{1}H Spectrum of **10** in $CDCl_3$ (500 MHz)

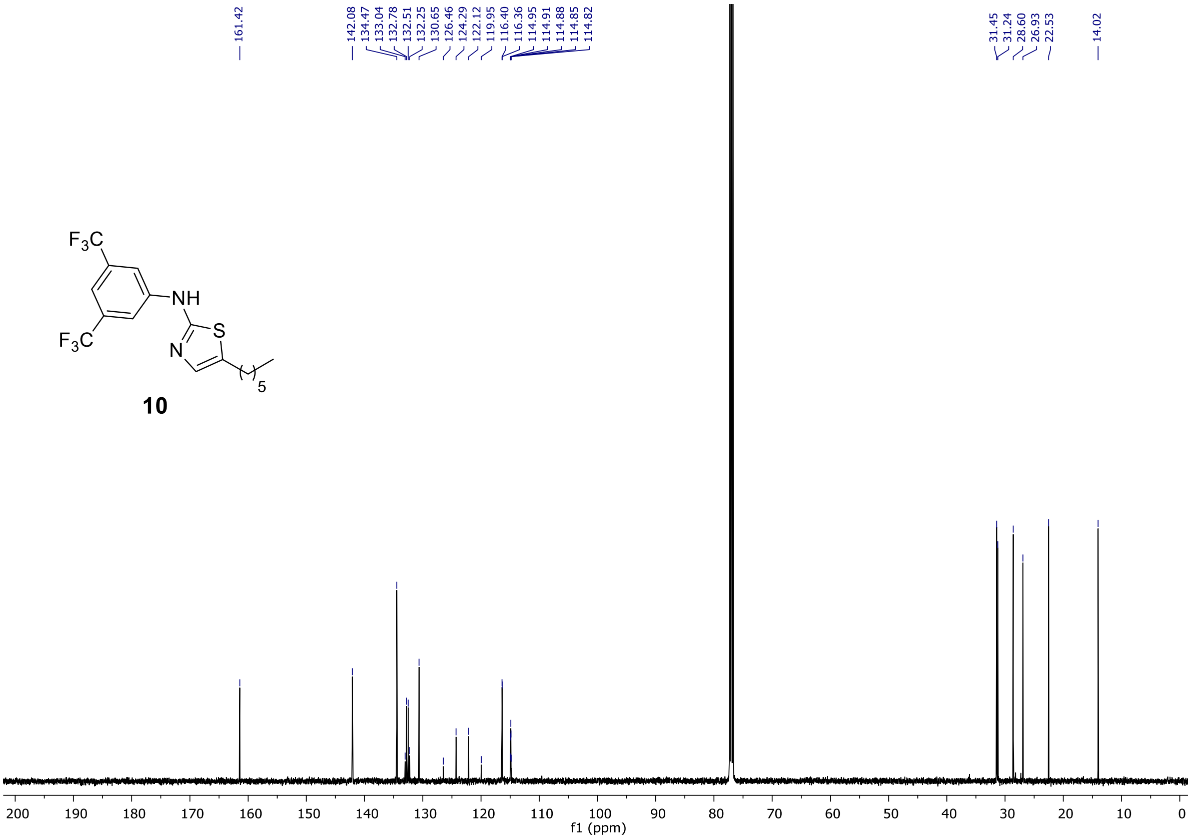

Figure S18. $^{13}C\{^{1}H\}$ Spectrum of **10** in $CDCl_3$ (125 MHz)

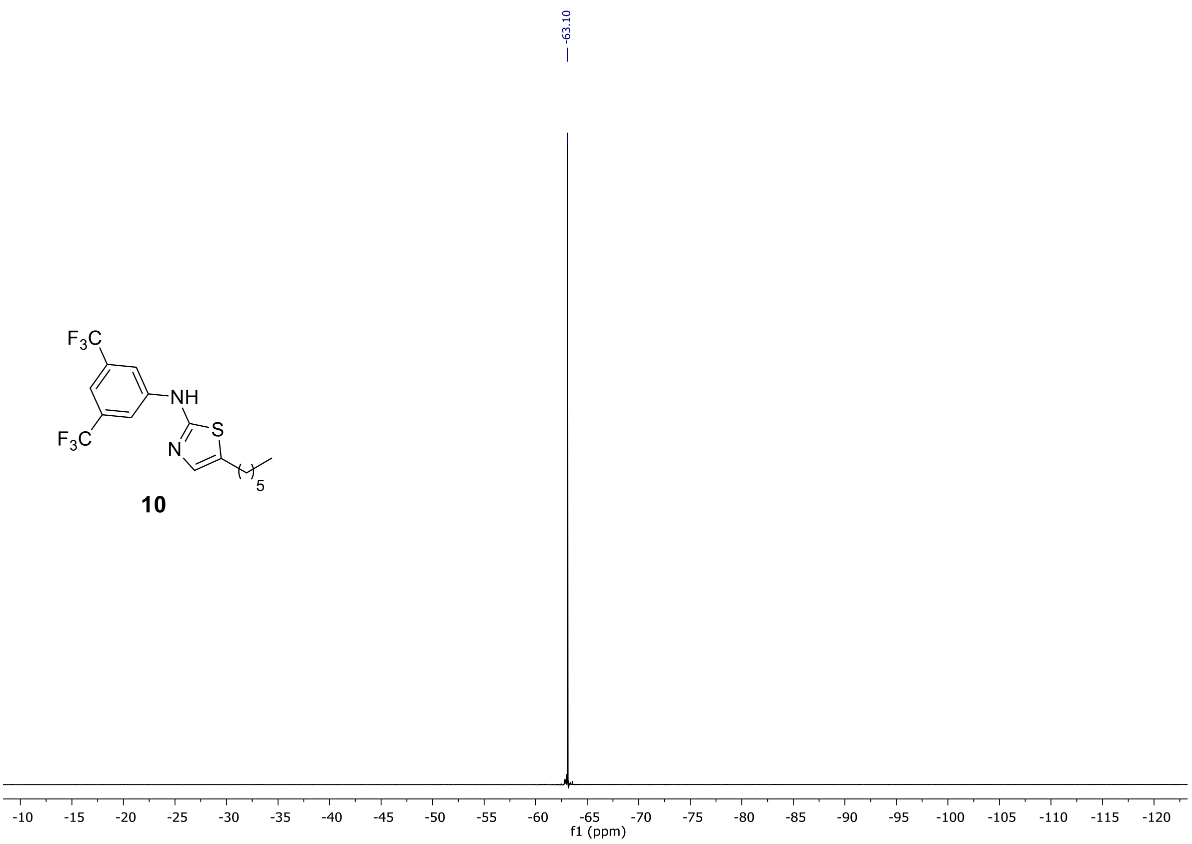

Figure S19. ^{19}F Spectrum of **10** in $CDCl_3$ (376 MHz)

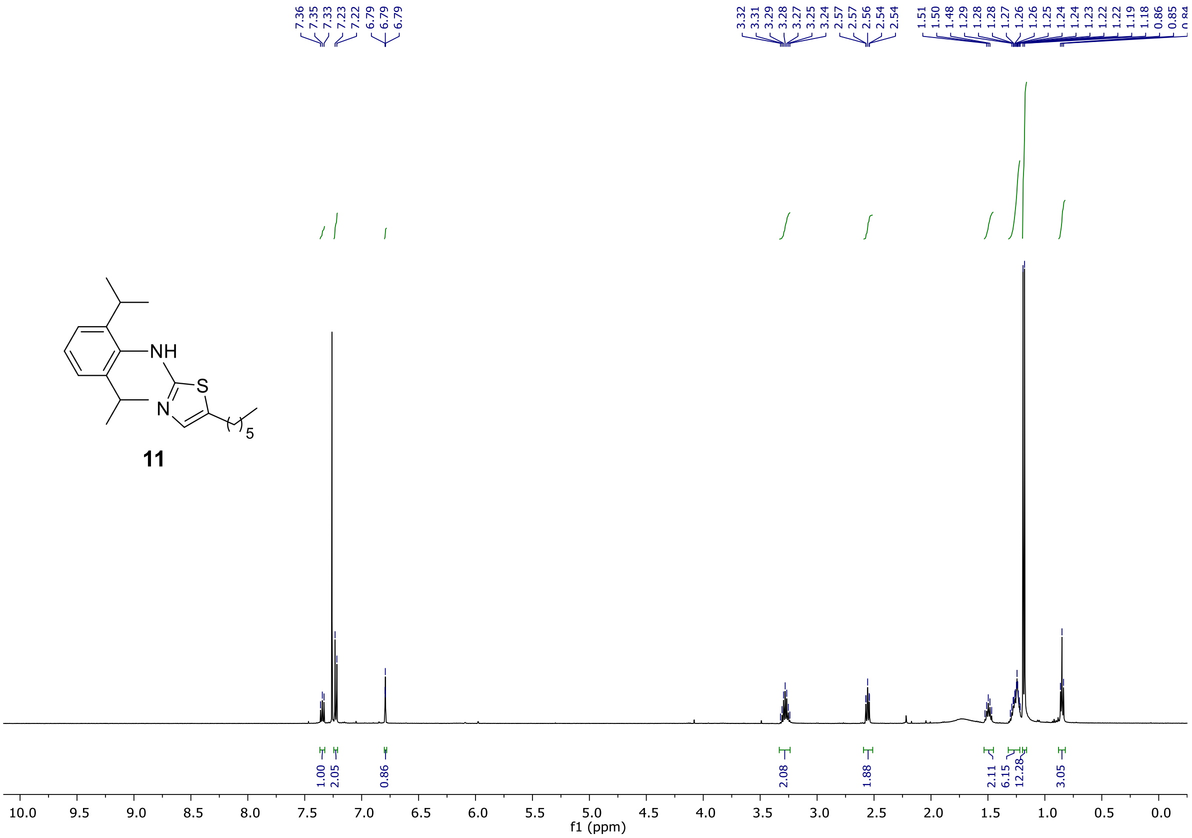

Figure S20. ^{1}H Spectrum of **11** in $CDCl_3$ (500 MHz)

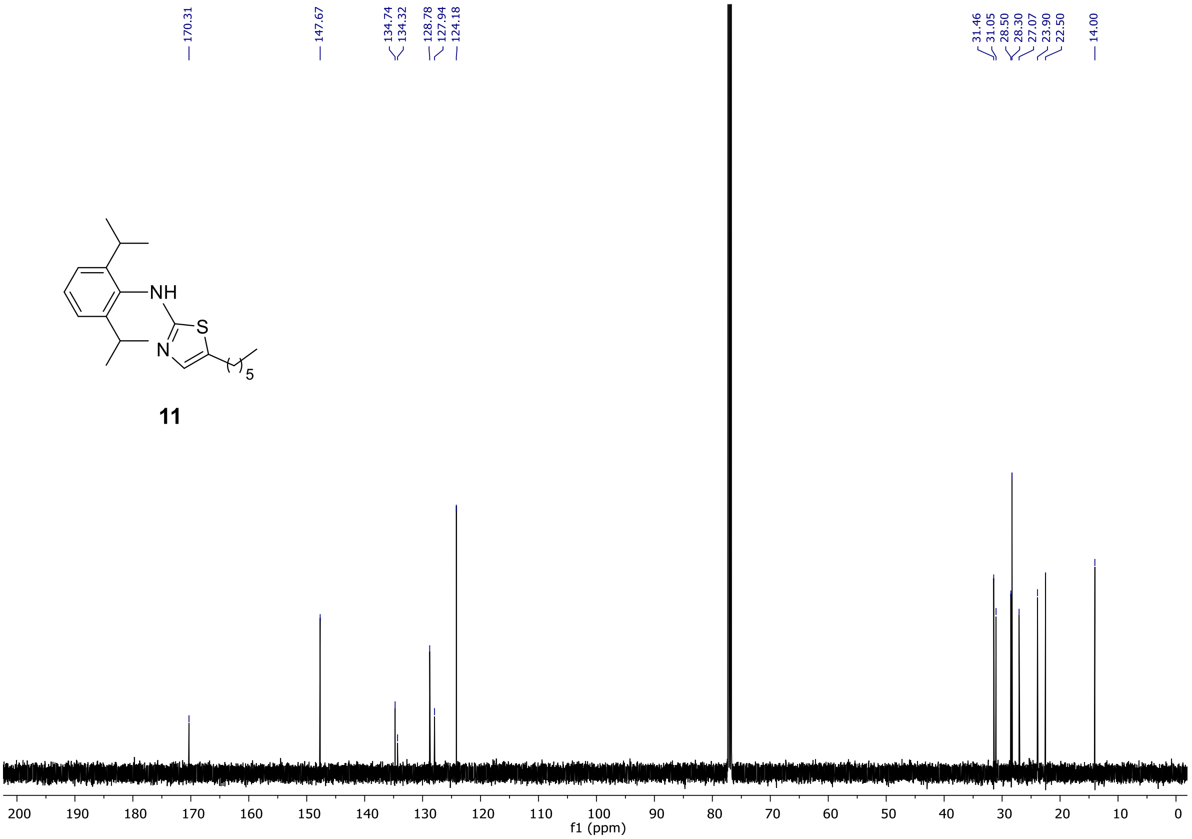

Figure S21. $^{13}C\{^{1}H\}$ Spectrum of **11** in $CDCl_3$ (125 MHz)

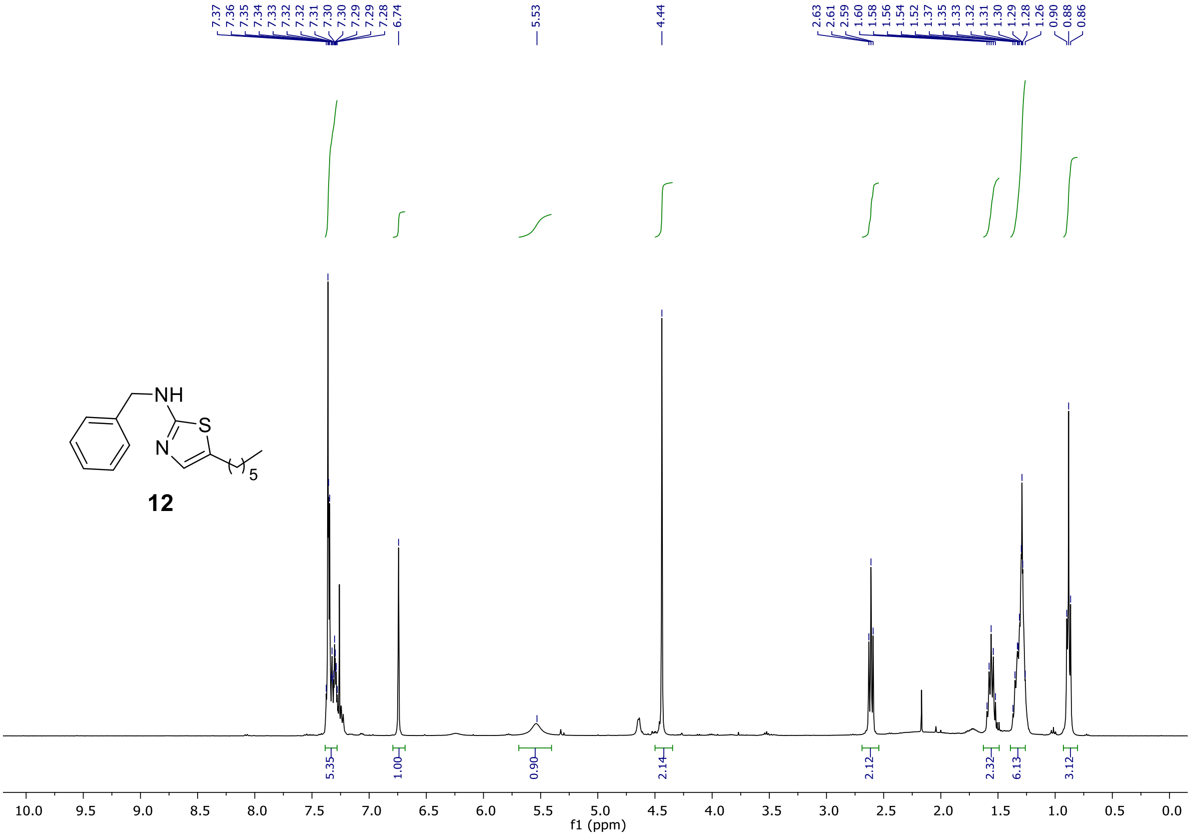

Figure S22. ^{1}H Spectrum of **12** in $CDCl_3$ (400 MHz)

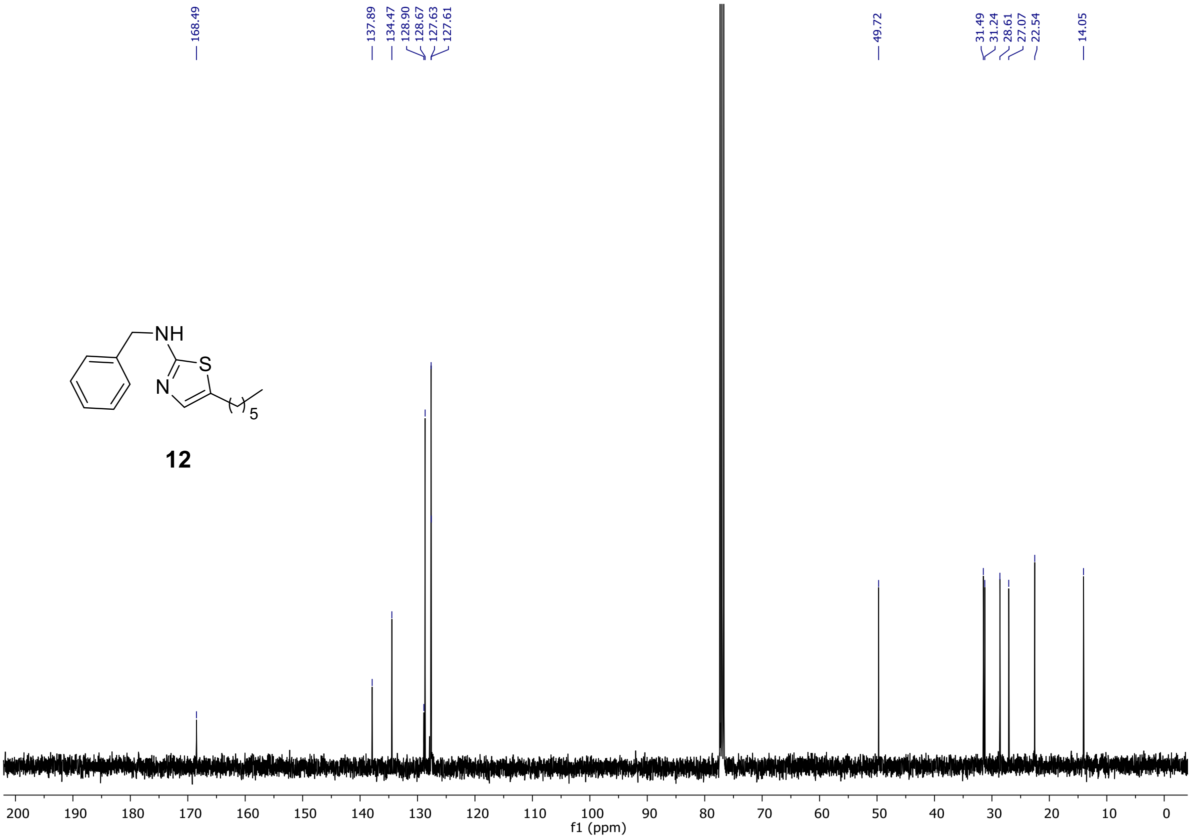

Figure S23. $^{13}C\{^{1}H\}$ Spectrum of **12** in $CDCl_3$ (100 MHz)

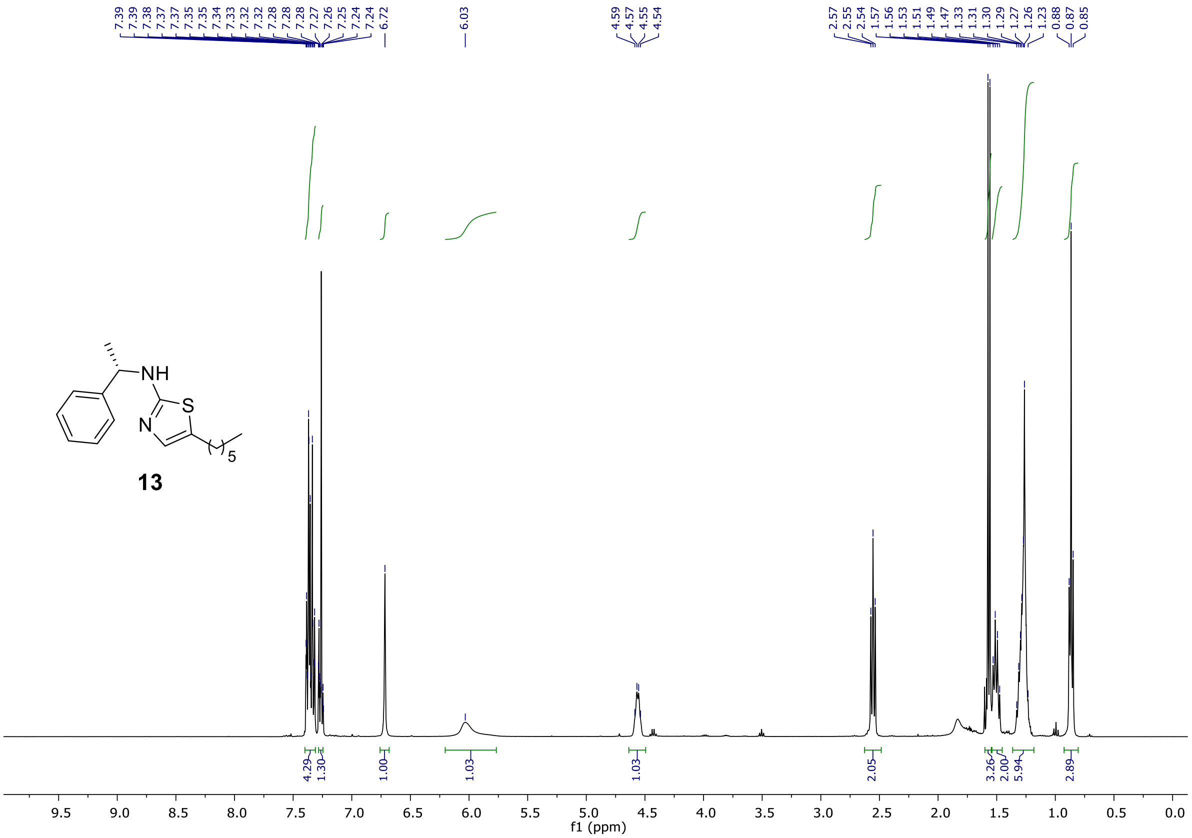

Figure S24. ^{1}H Spectrum of **13** in $CDCl_3$ (400 MHz)

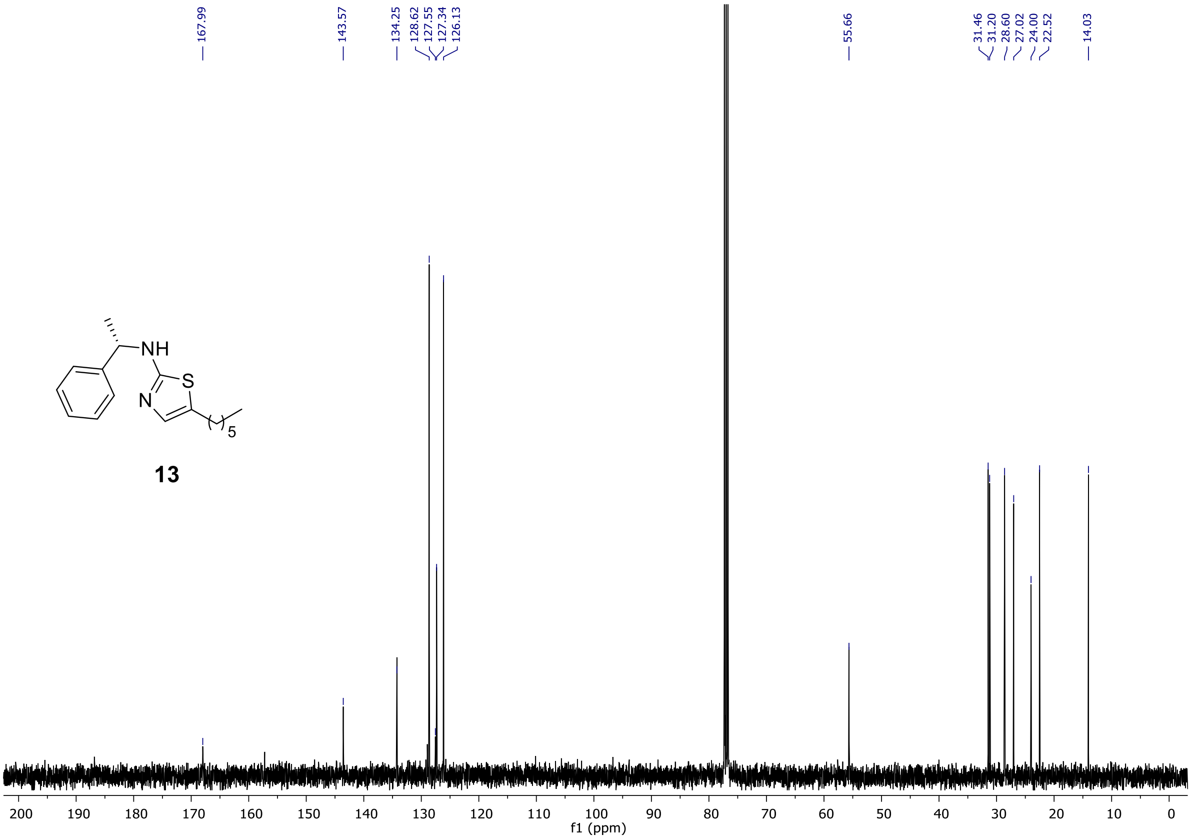

Figure S25. $^{13}C\{^{1}H\}$ Spectrum of **13** in $CDCl_3$ (100 MHz)

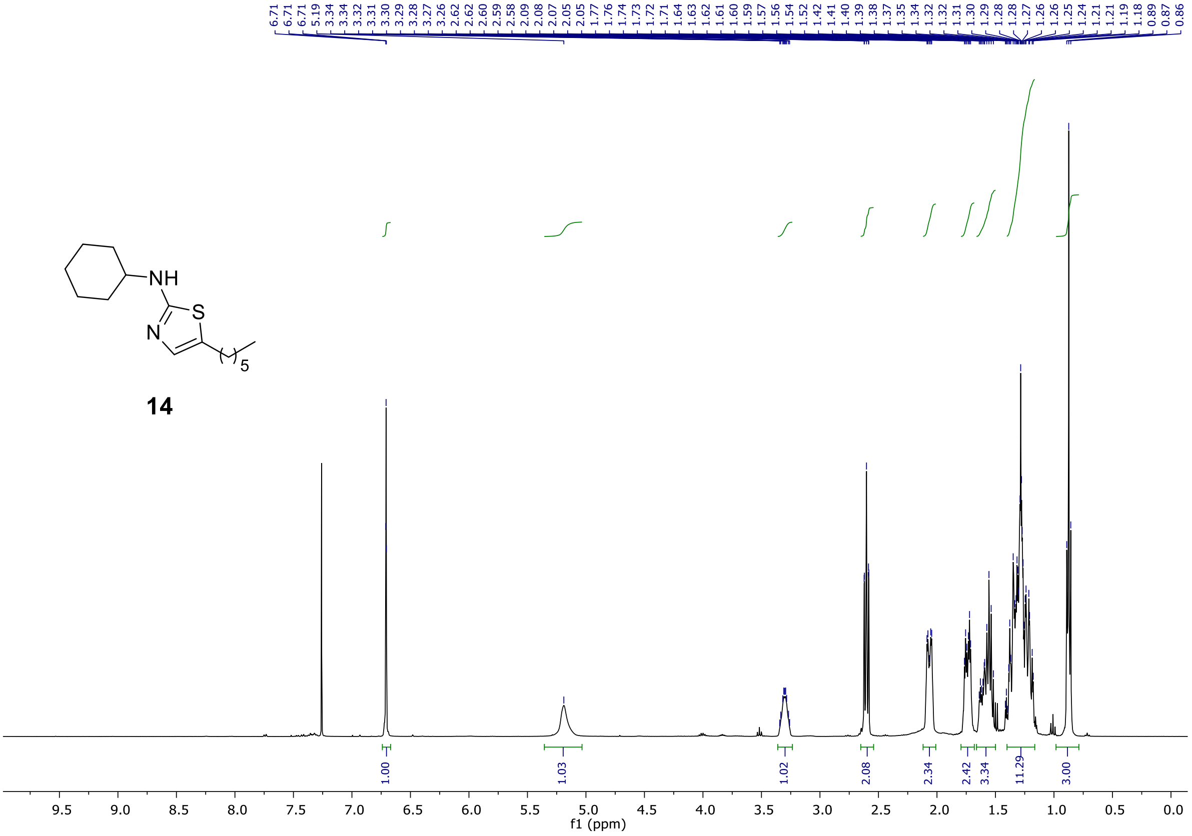

Figure S26. ^{1}H Spectrum of **14** in $CDCl_3$ (400 MHz)

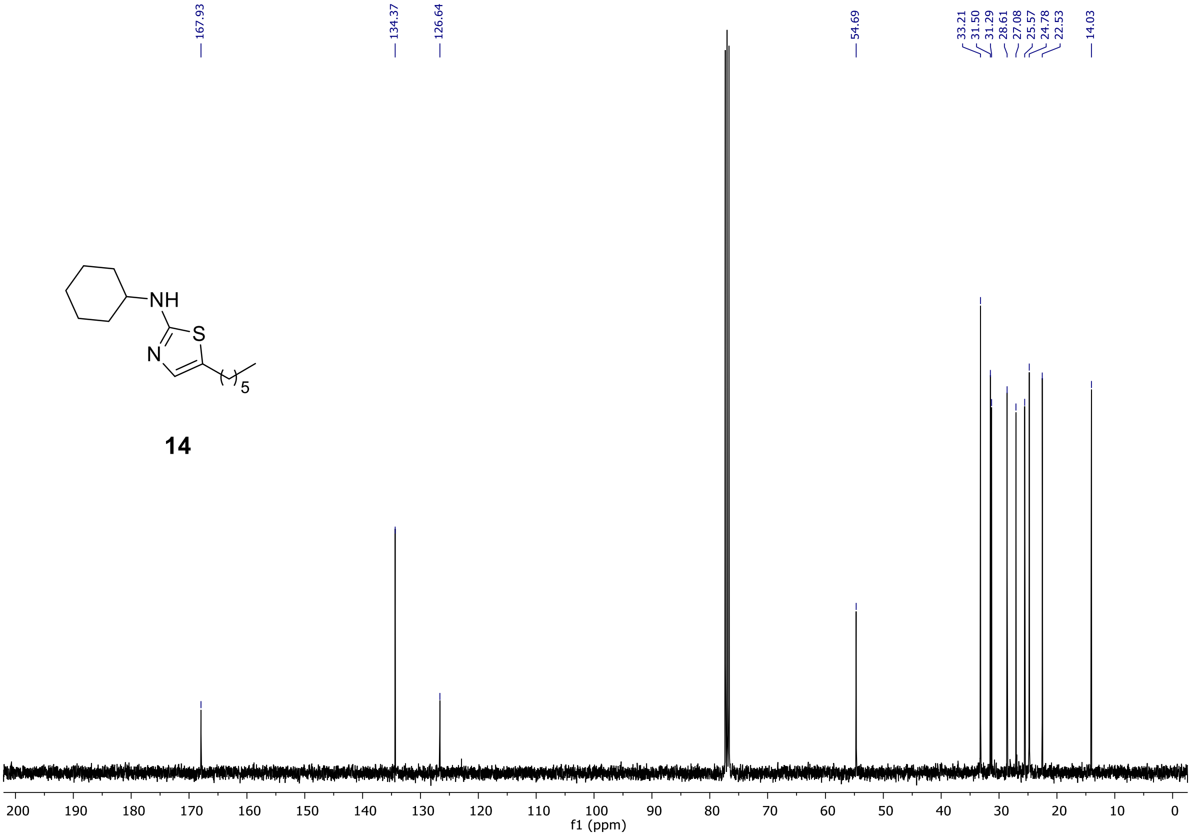

Figure S27. $^{13}C\{^{1}H\}$ Spectrum of **14** in $CDCl_3$ (100 MHz)

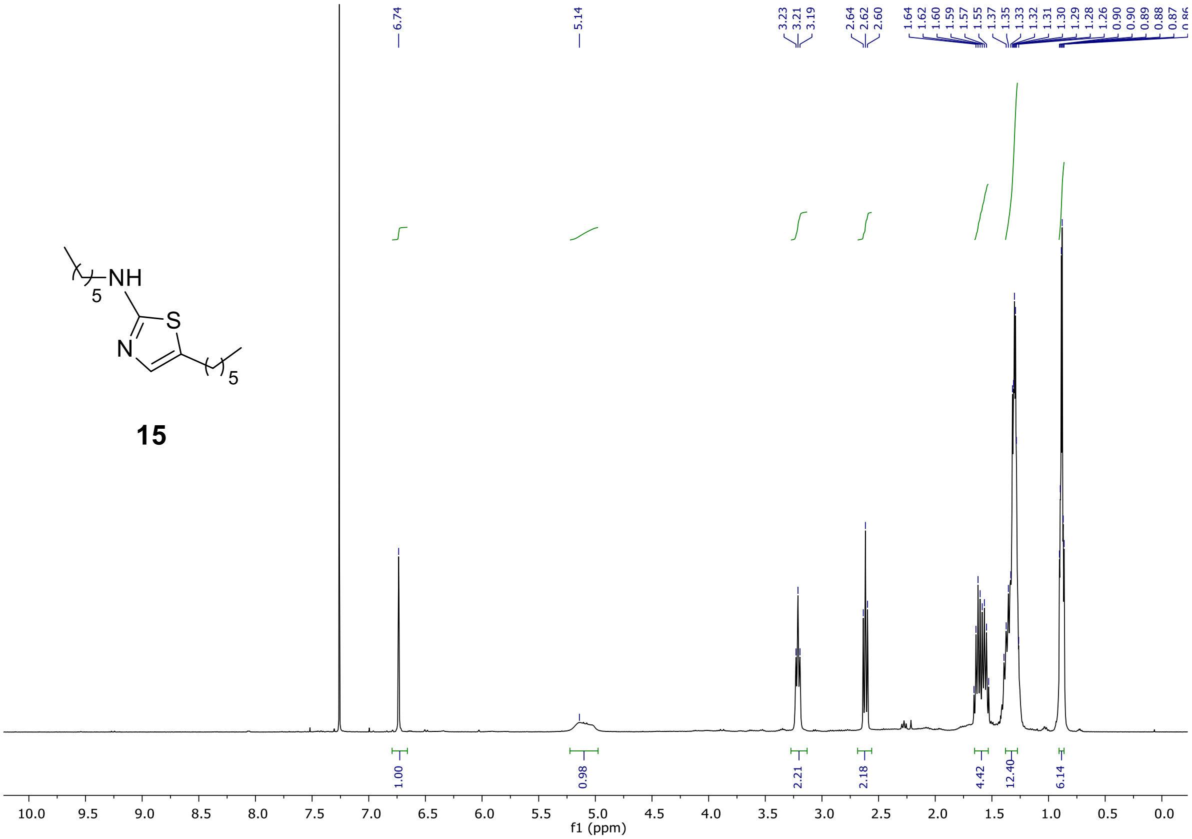

Figure S28. ^{1}H Spectrum of **15** in $CDCl_3$ (400 MHz)

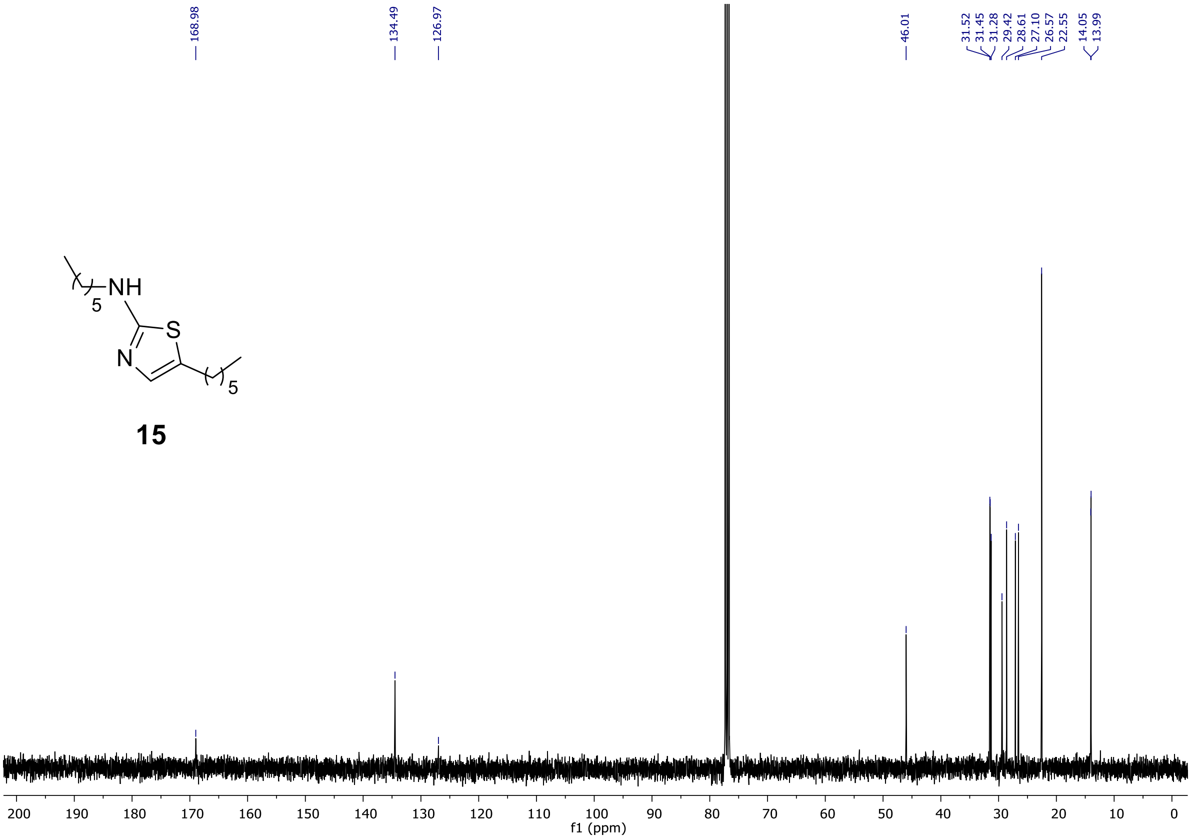

Figure S29. $^{13}C\{^{1}H\}$ Spectrum of **15** in $CDCl_3$ (100 MHz)

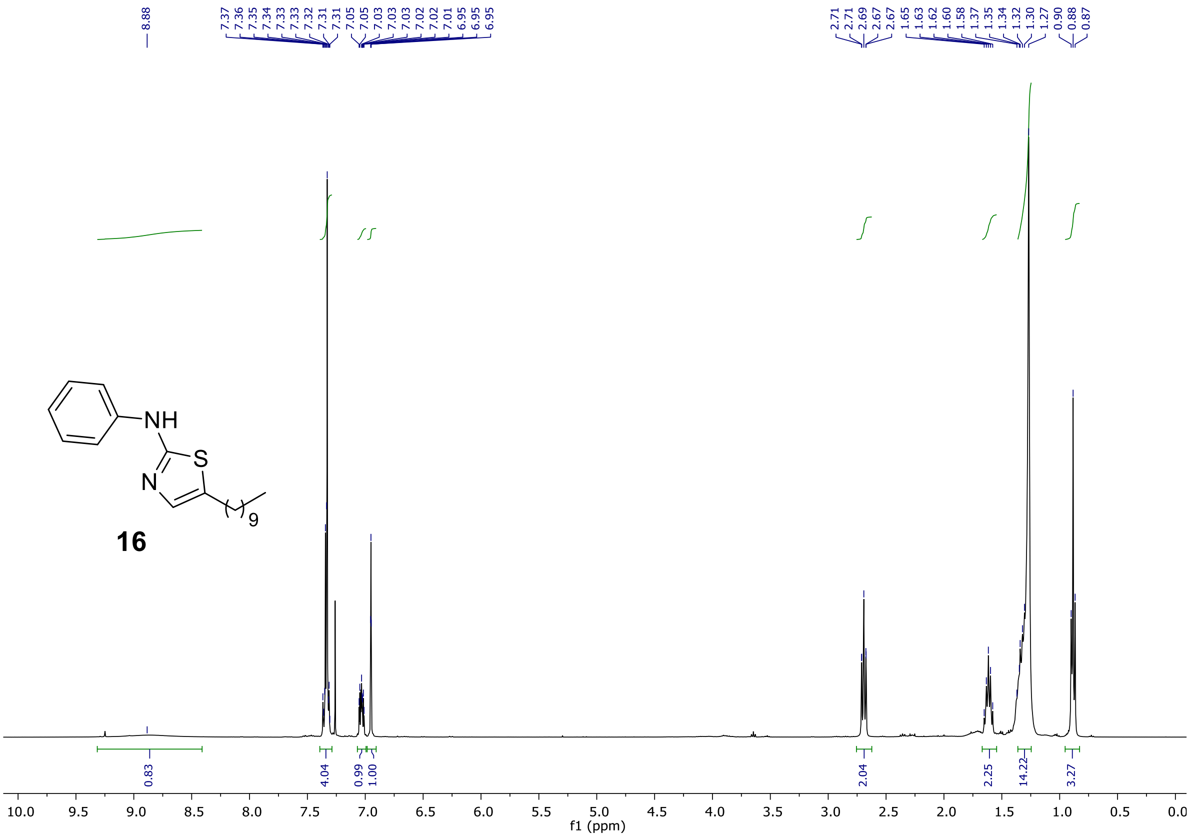

Figure S30. ^{1}H Spectrum of **16** in $CDCl_3$ (400 MHz)

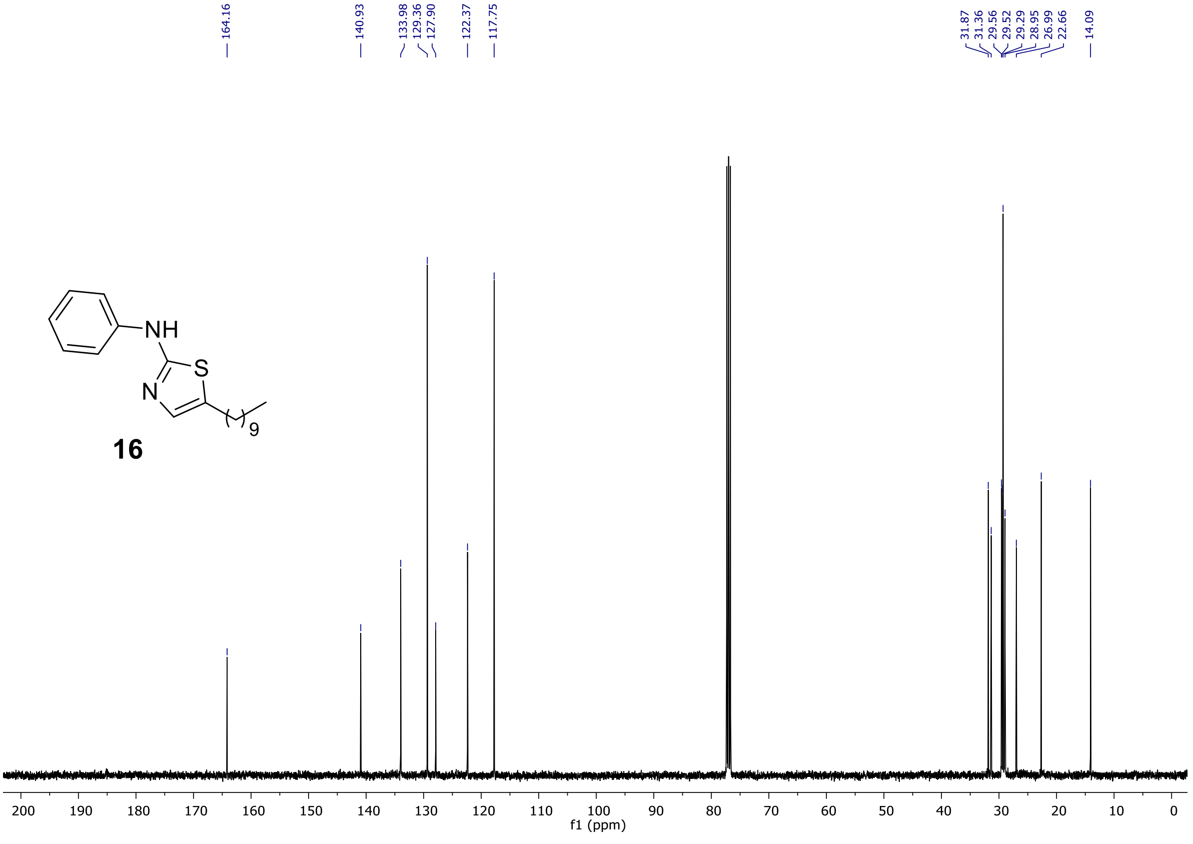

Figure S31. $^{13}C\{^{1}H\}$ Spectrum of **16** in $CDCl_3$ (100 MHz)

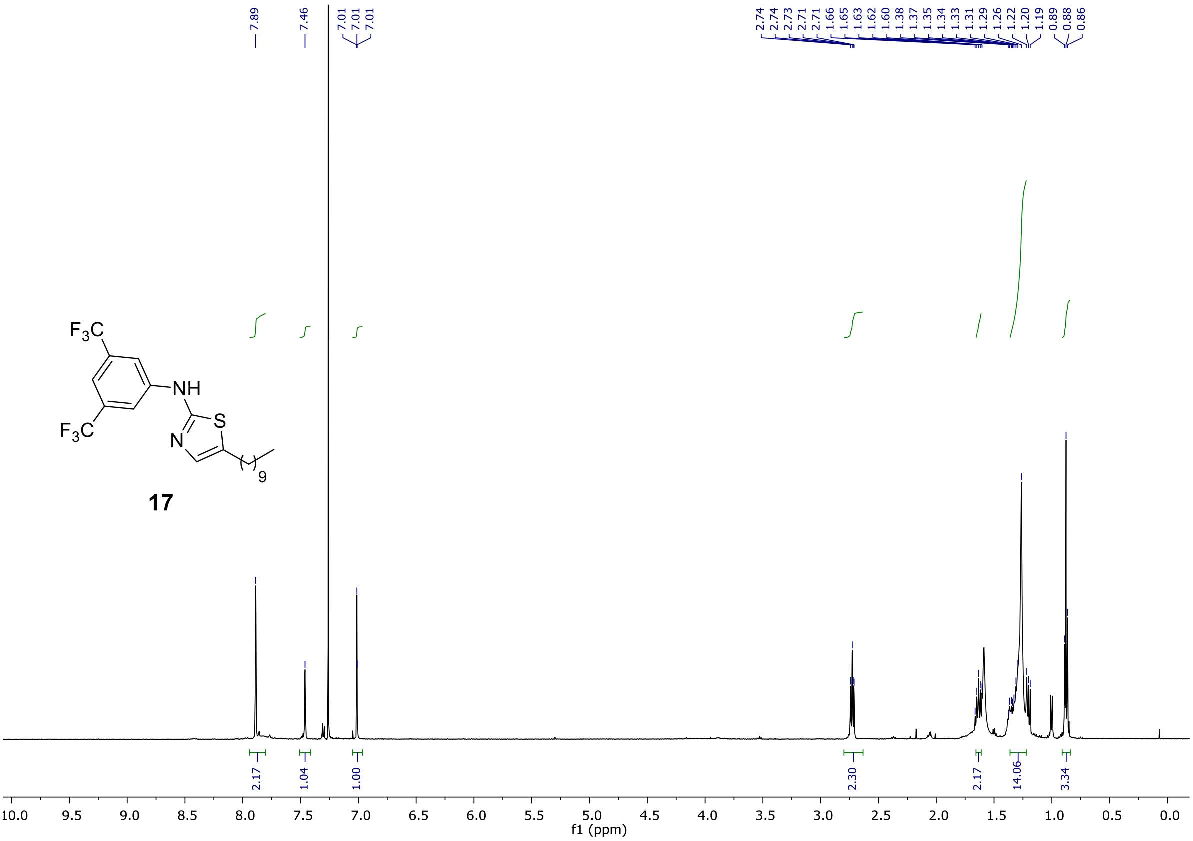

Figure S32. ^{1}H Spectrum of **17** in $CDCl_3$ (500 MHz)

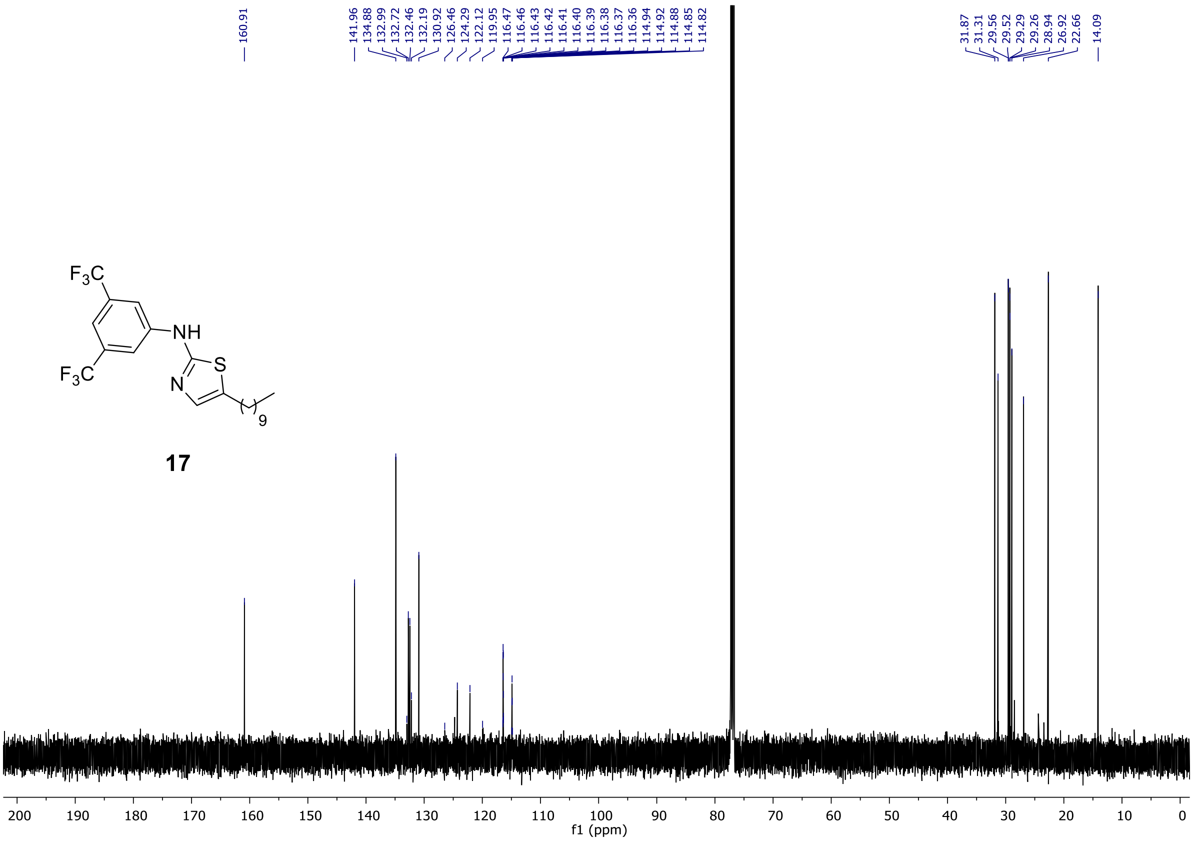

Figure S33. $^{13}C\{^{1}H\}$ Spectrum of **17** in $CDCl_3$ (125 MHz)

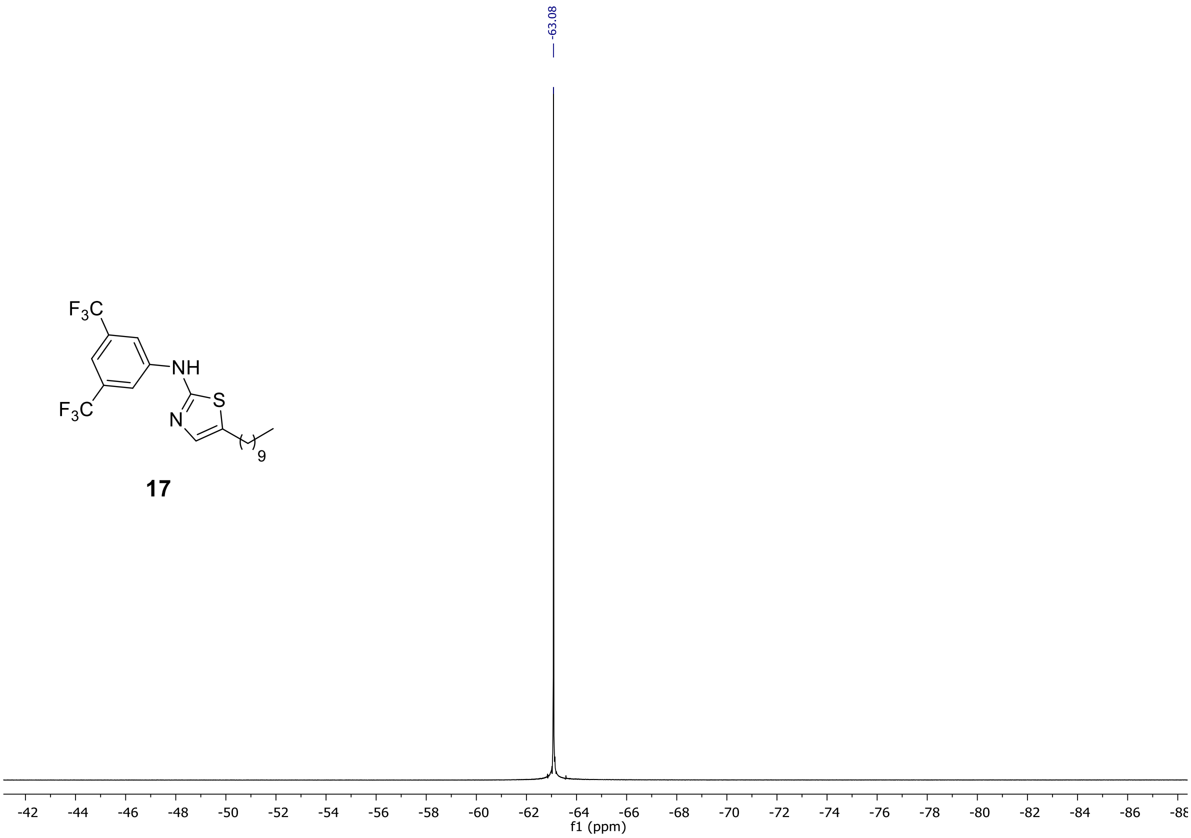

Figure S34. ^{19}F Spectrum of **17** in $CDCl_3$ (376 MHz)

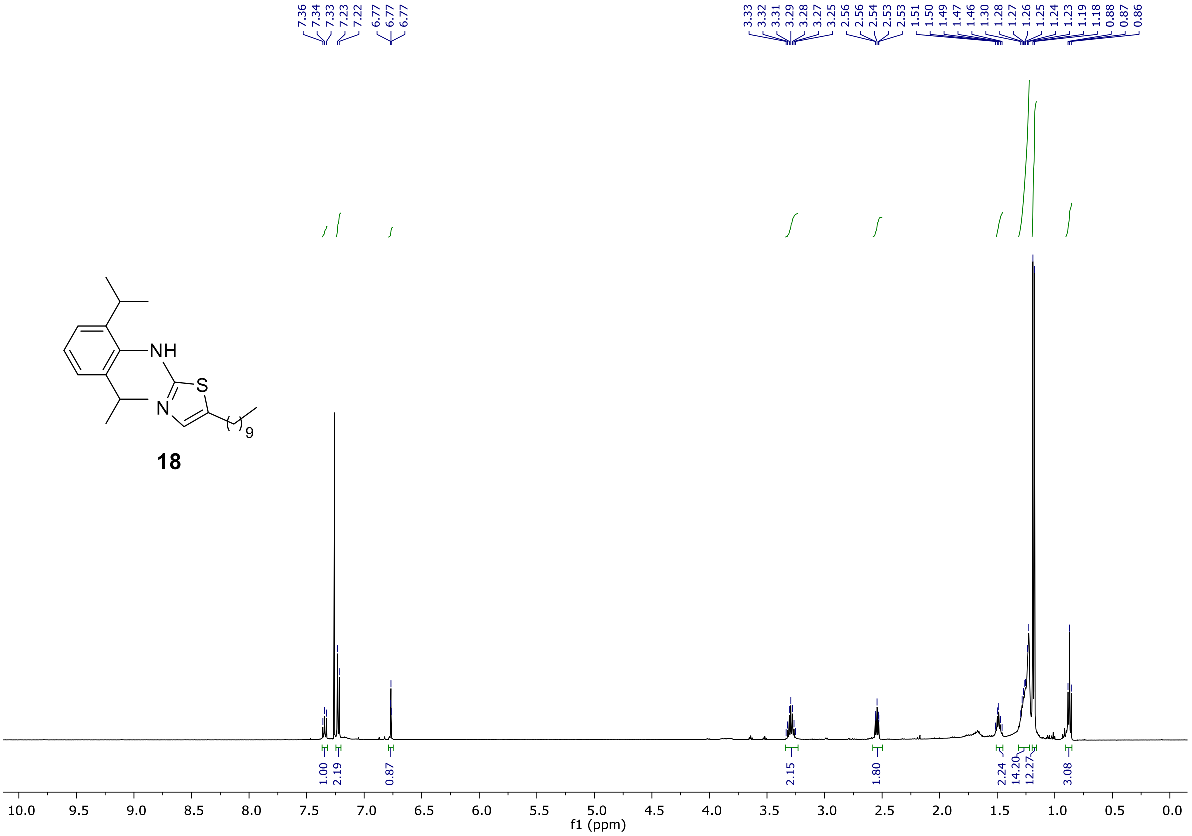

Figure S35. ^{1}H Spectrum of **18** in $CDCl_3$ (500 MHz)

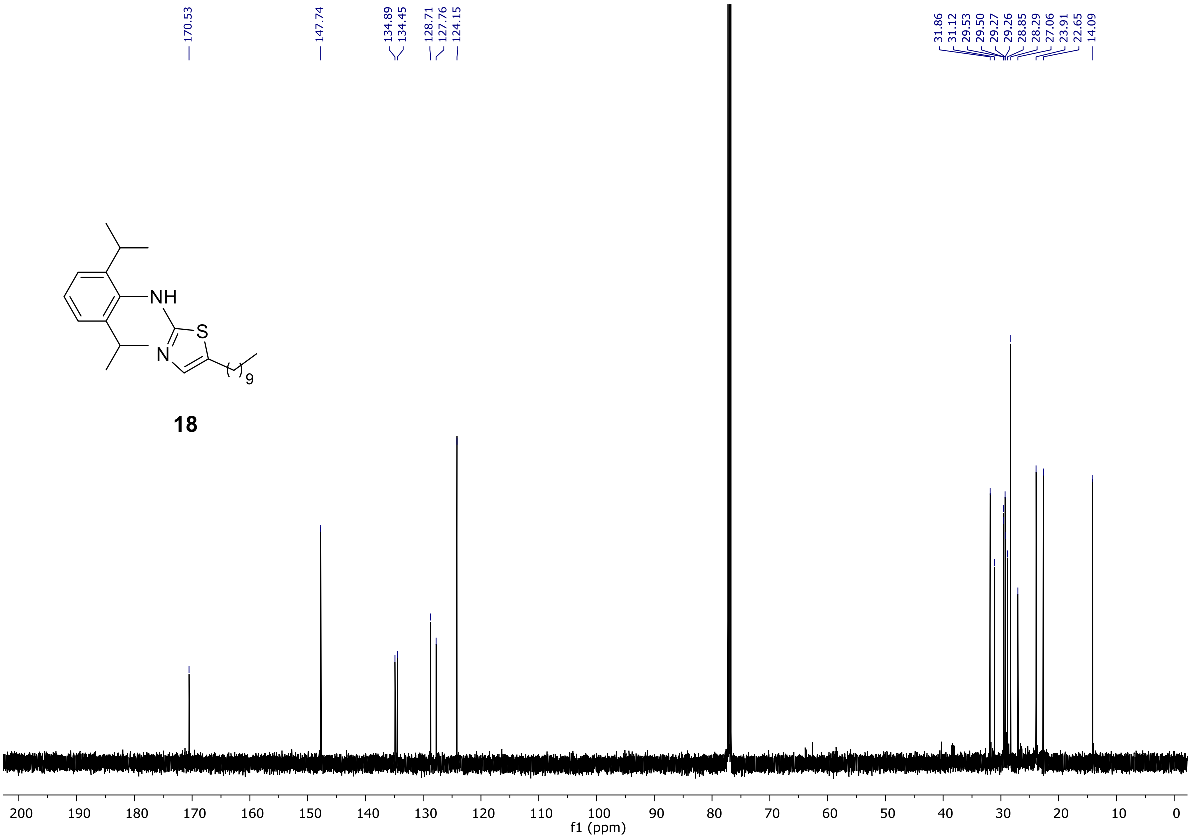

Figure S36. $^{13}C\{^{1}H\}$ Spectrum of **18** in $CDCl_3$ (125 MHz)

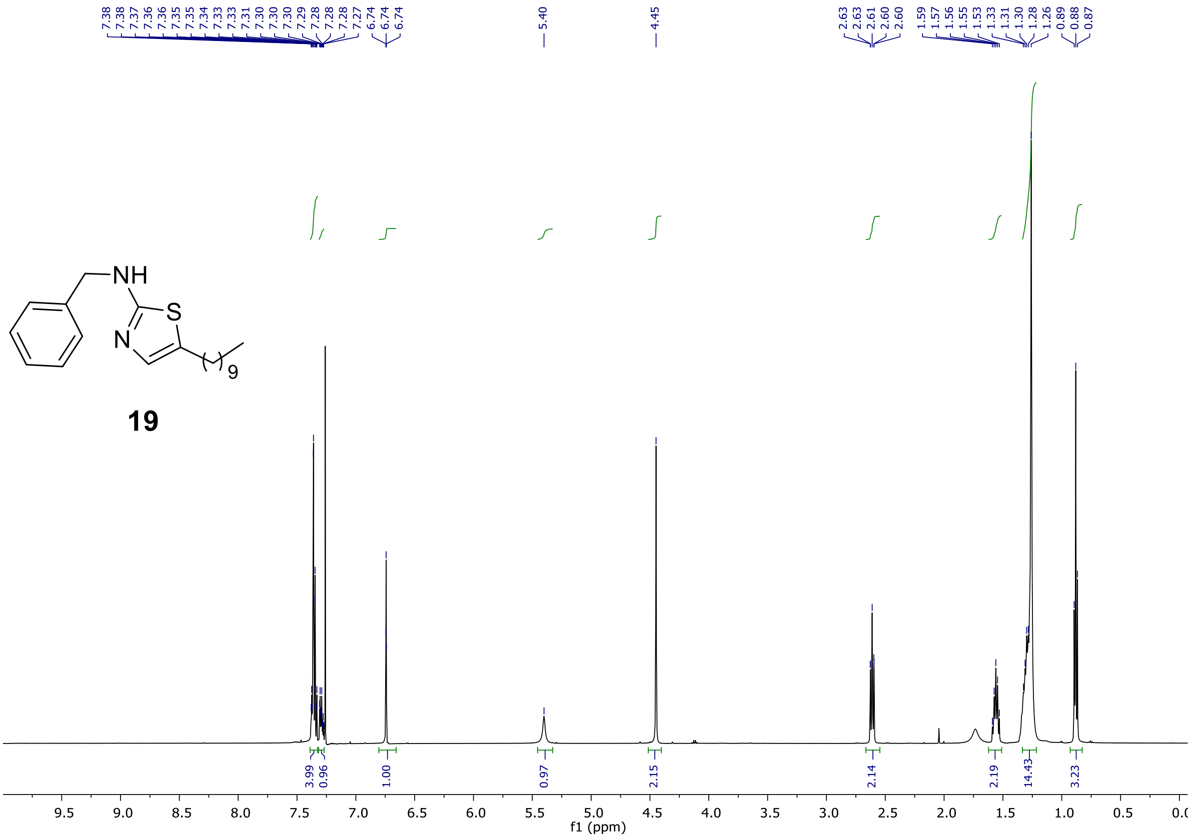

Figure S37. ^{1}H Spectrum of **19** in $CDCl_3$ (500 MHz)

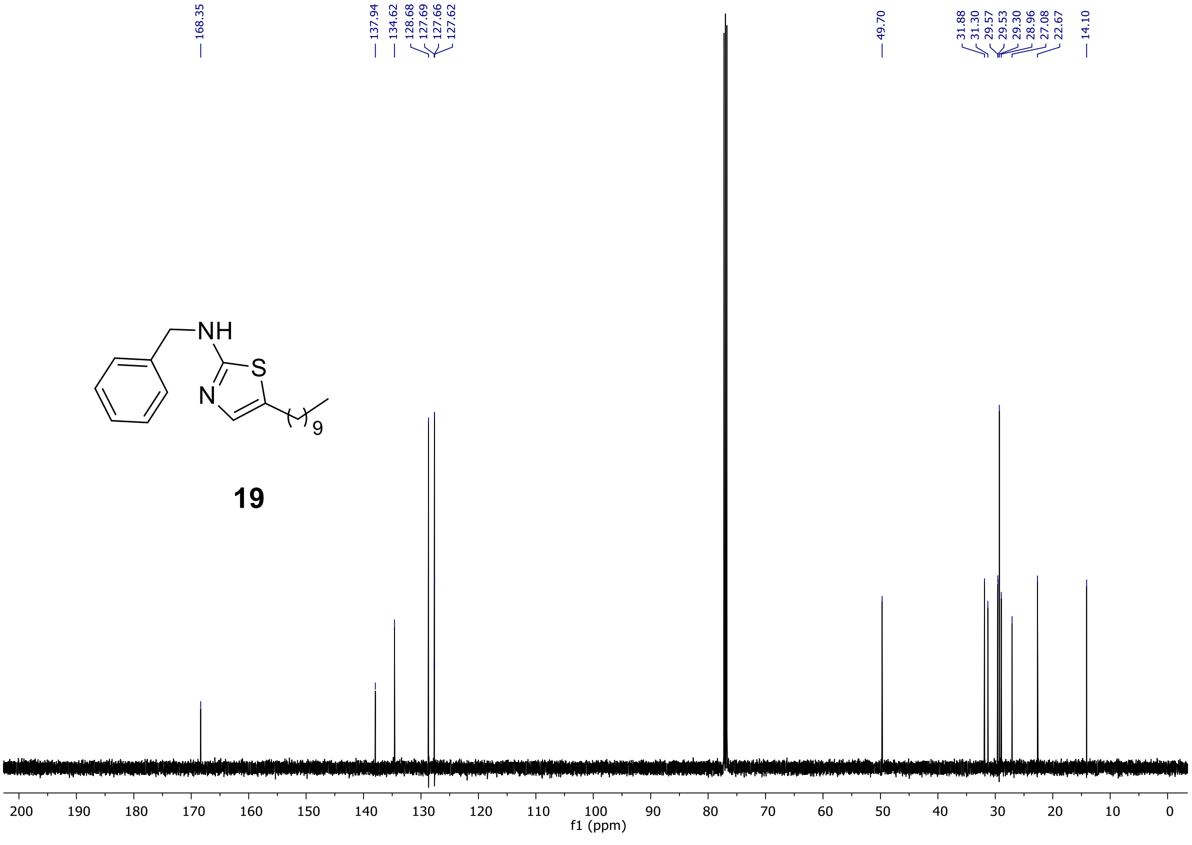

Figure S38. $^{13}C\{^{1}H\}$ Spectrum of **19** in $CDCl_3$ (125 MHz)

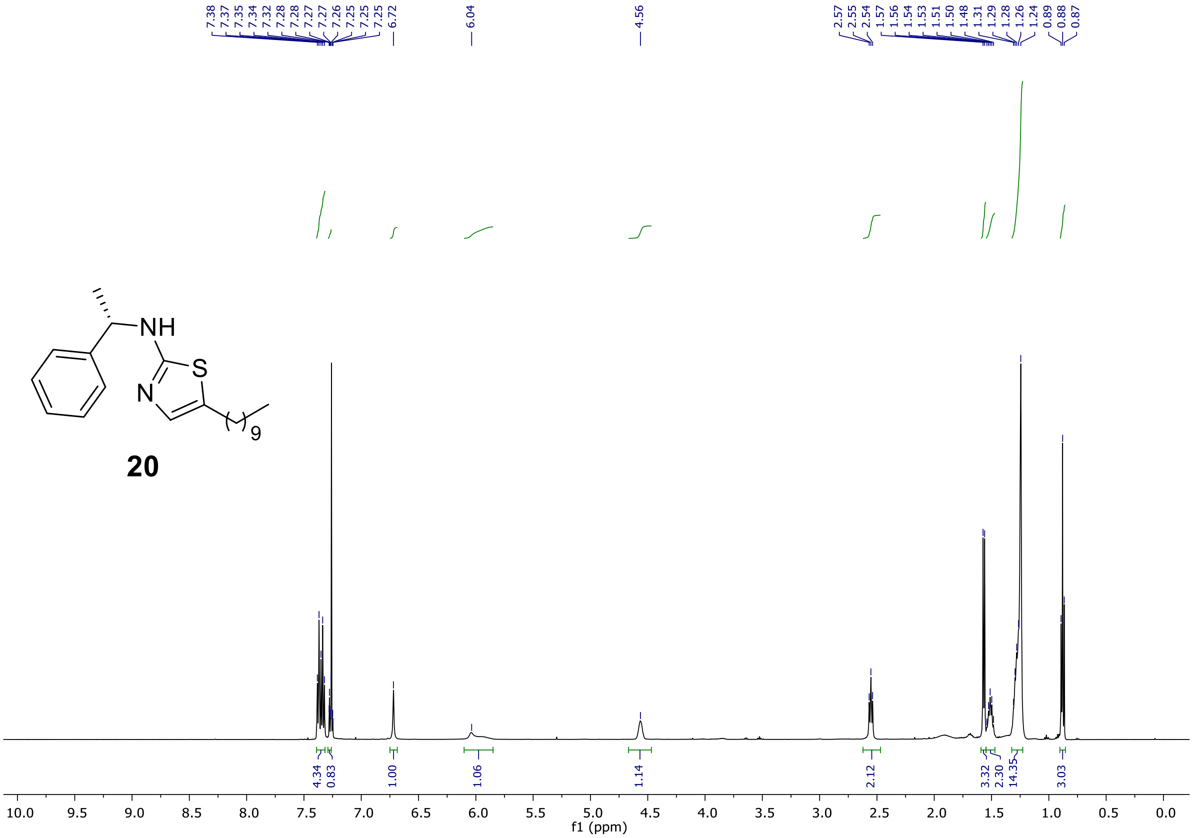

Figure S39. ^{1}H Spectrum of **20** in $CDCl_3$ (500 MHz)

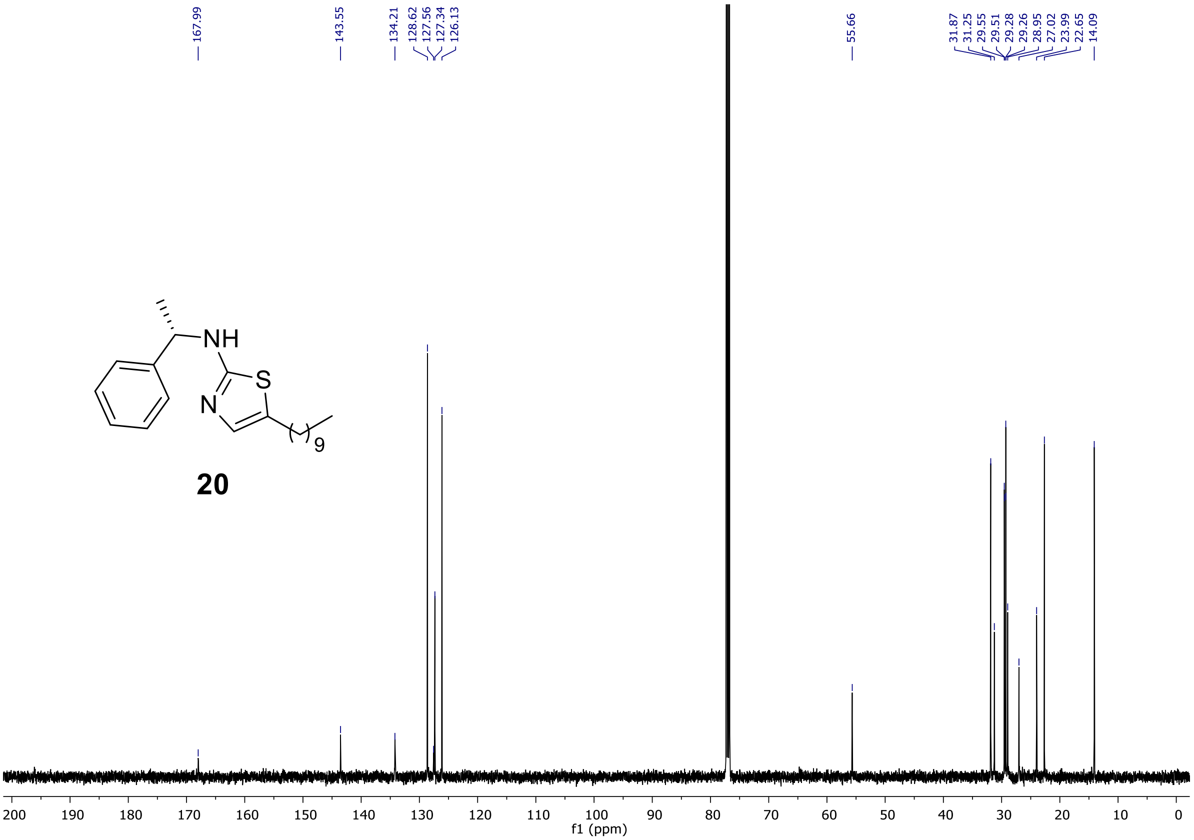

Figure S40. $^{13}C\{^{1}H\}$ Spectrum of **20** in $CDCl_3$ (125 MHz)

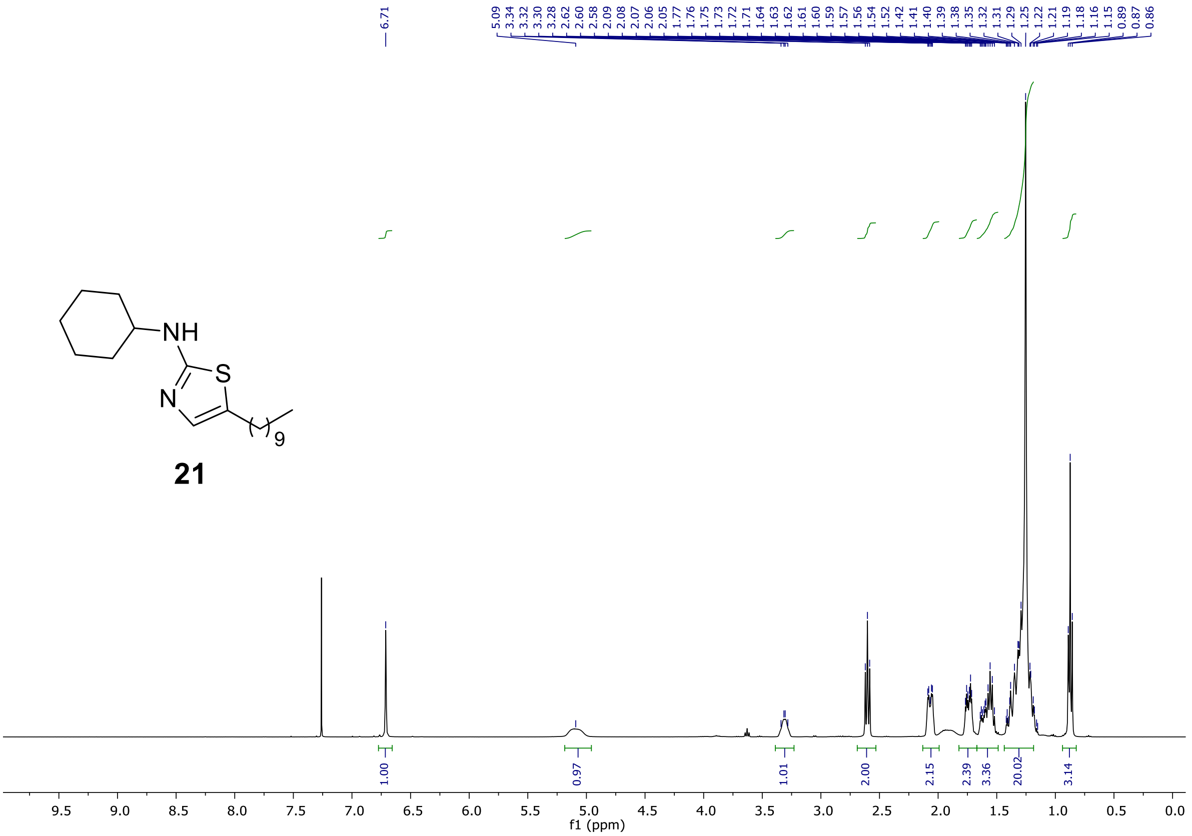

Figure S41. ^{1}H Spectrum of **21** in $CDCl_3$ (400 MHz)

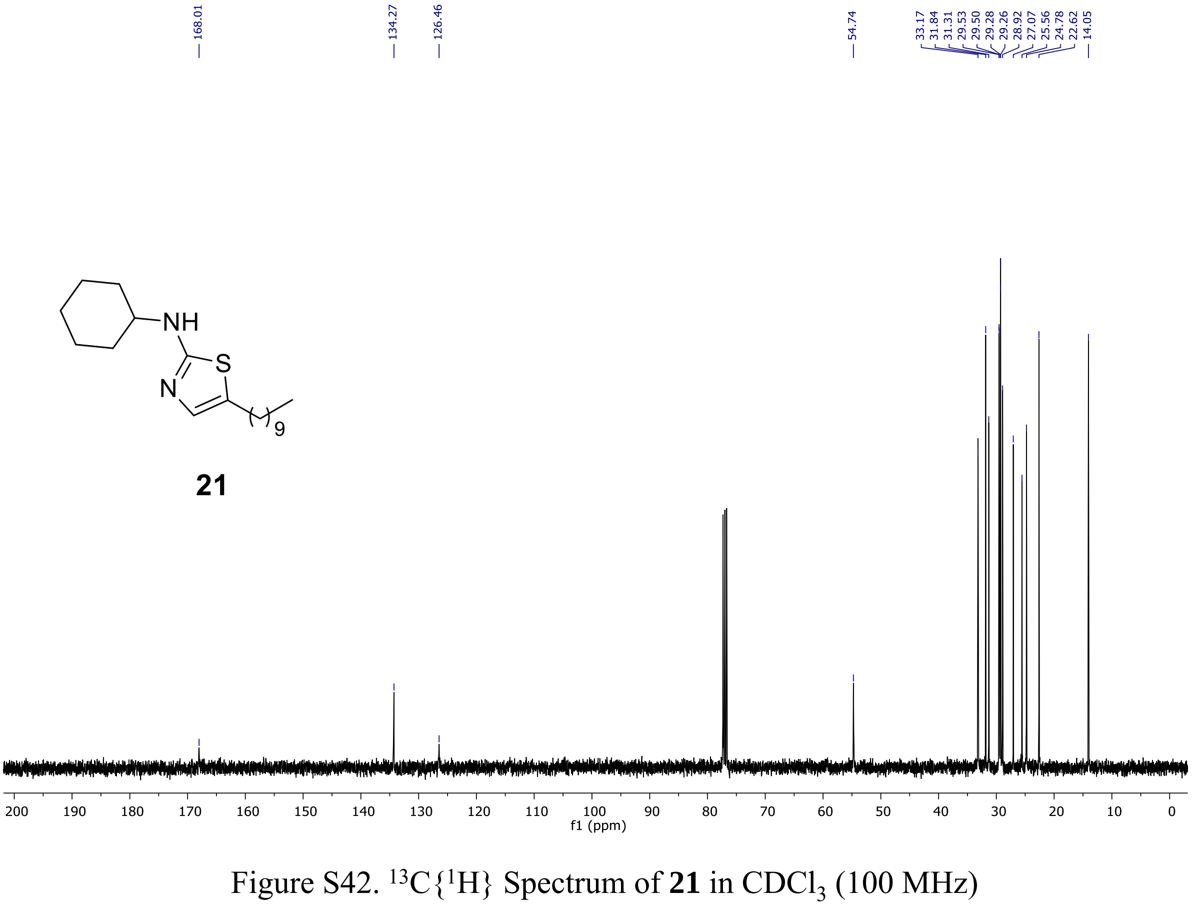

Figure S42. $^{13}C\{^{1}H\}$ Spectrum of **21** in $CDCl_3$ (100 MHz)

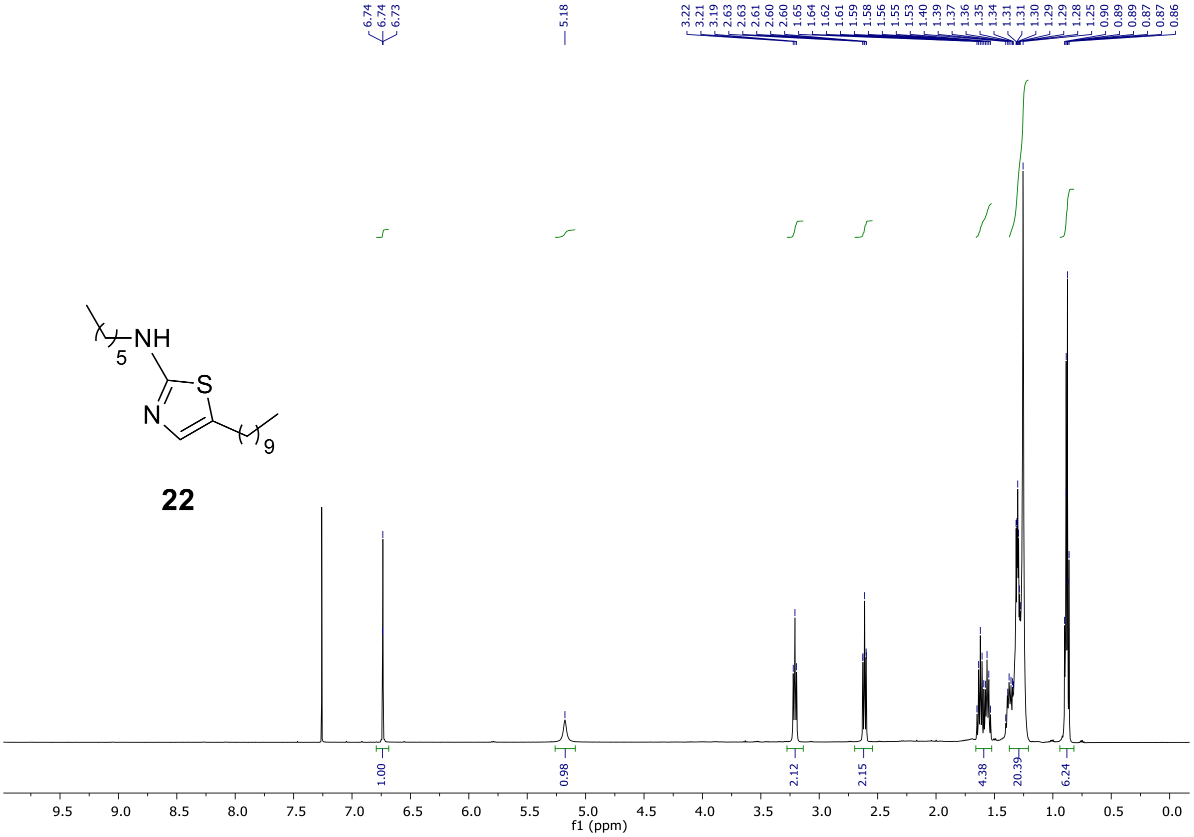

Figure S43. ^{1}H Spectrum of **22** in $CDCl_3$ (500 MHz)

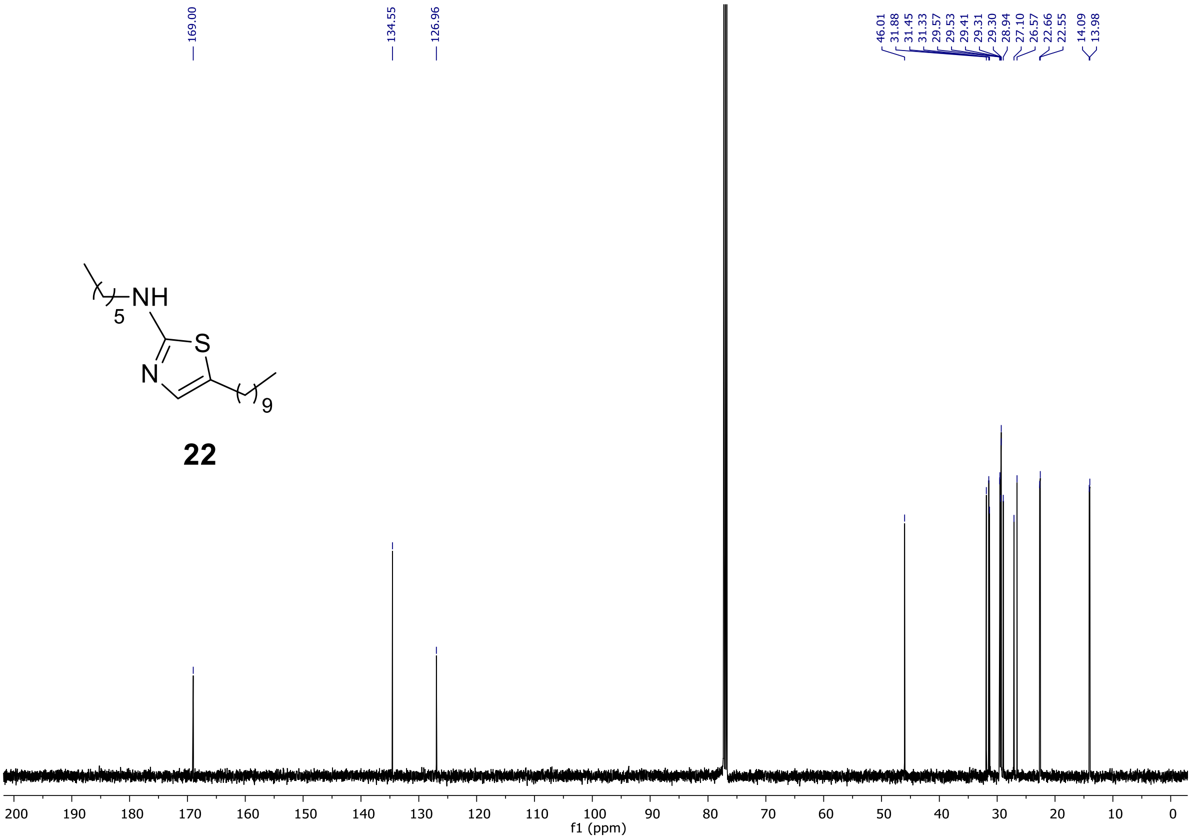

Figure S44. $^{13}C\{^{1}H\}$ Spectrum of **22** in $CDCl_3$ (125 MHz)

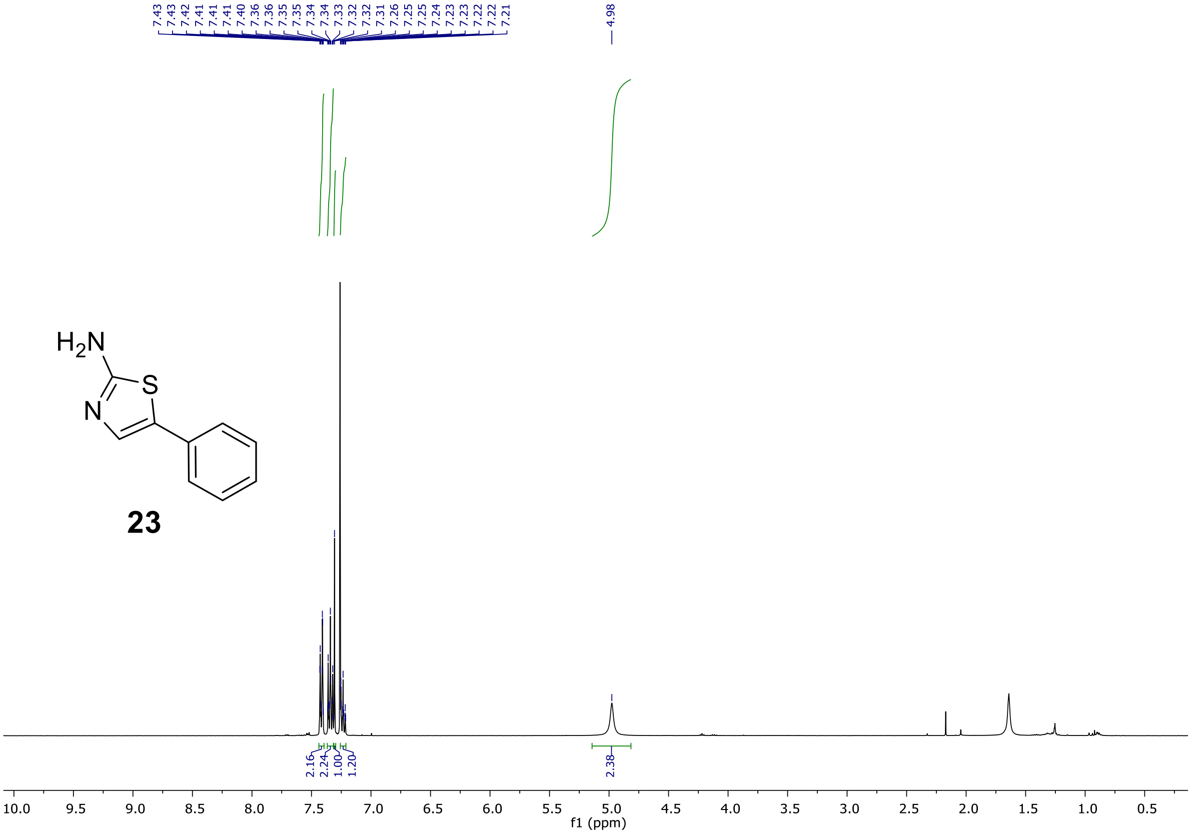

Figure S45. ^{1}H Spectrum of **23** in $CDCl_3$ (400 MHz)

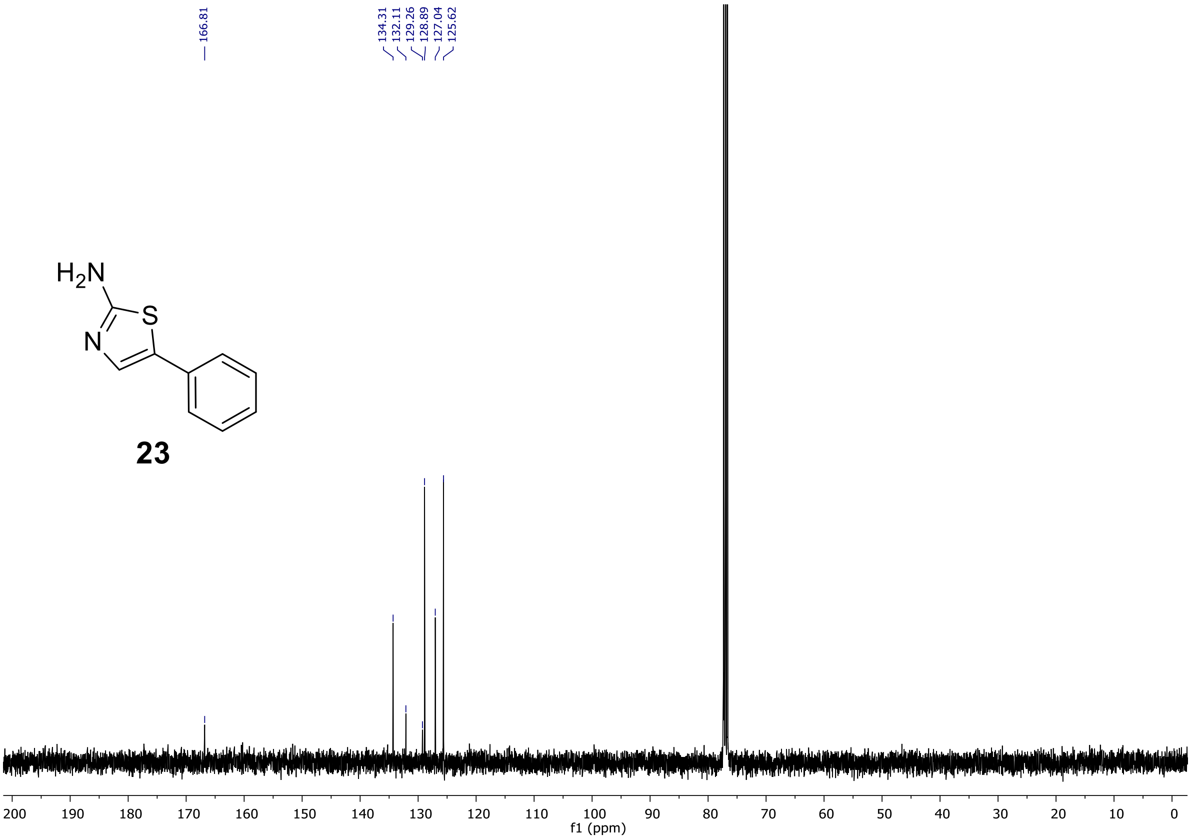

Figure S46. $^{13}C\{^{1}H\}$ Spectrum of **23** in $CDCl_3$ (100 MHz)

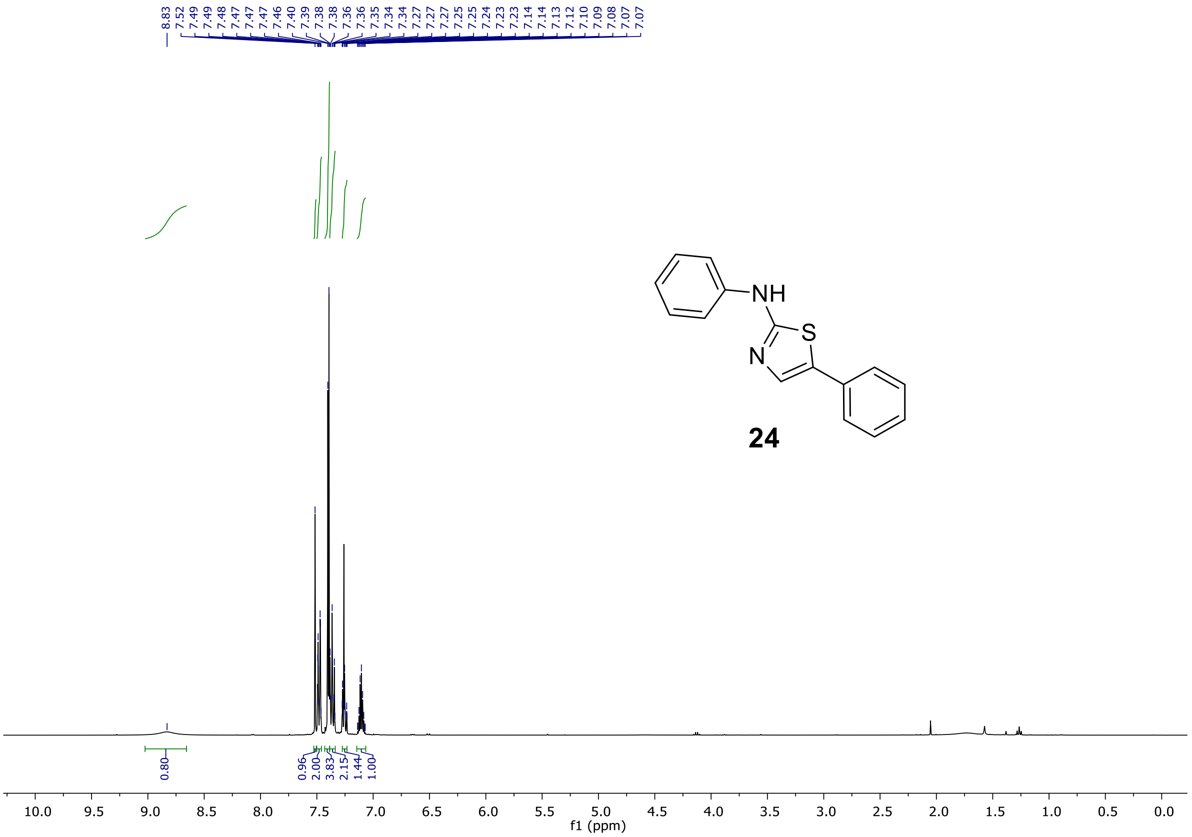

Figure S47. ^{1}H Spectrum of **24** in $CDCl_3$ (400 MHz)

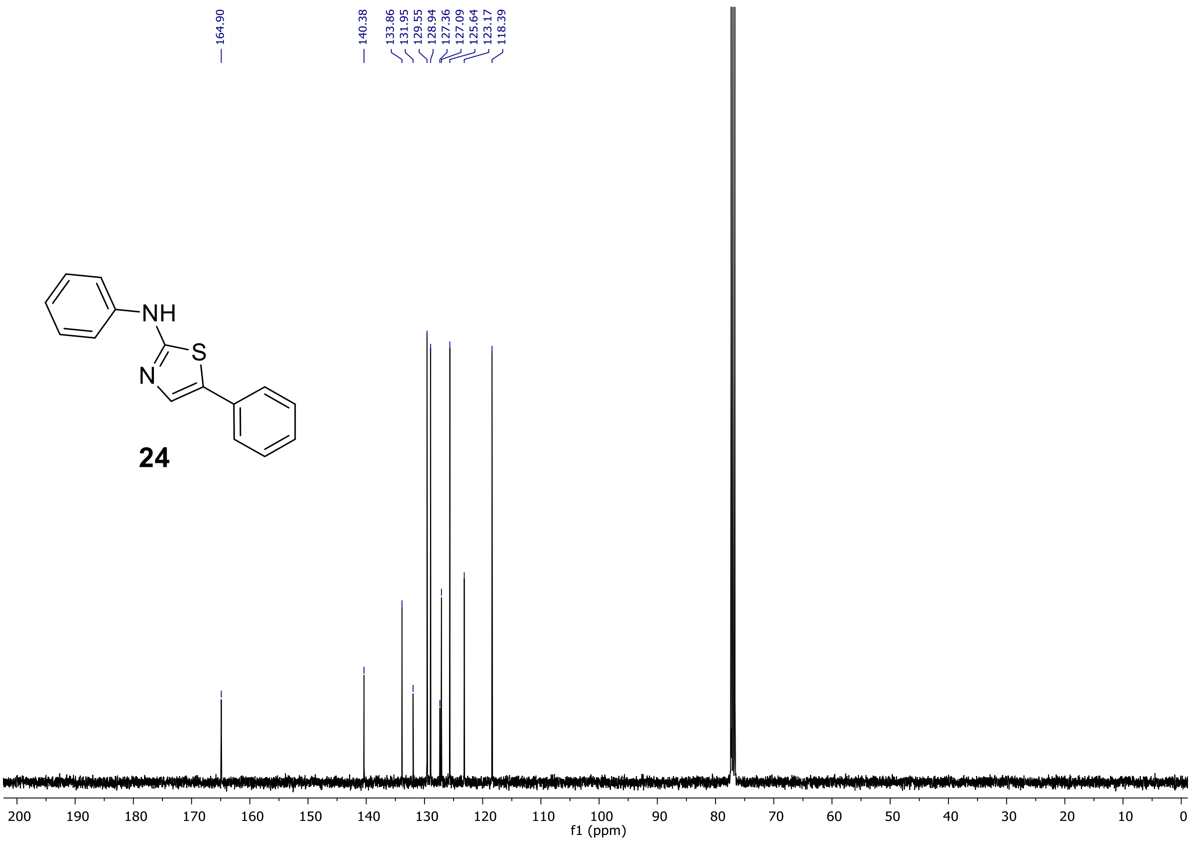

Figure S48. $^{13}C\{^{1}H\}$ Spectrum of **24** in $CDCl_3$ (100 MHz)

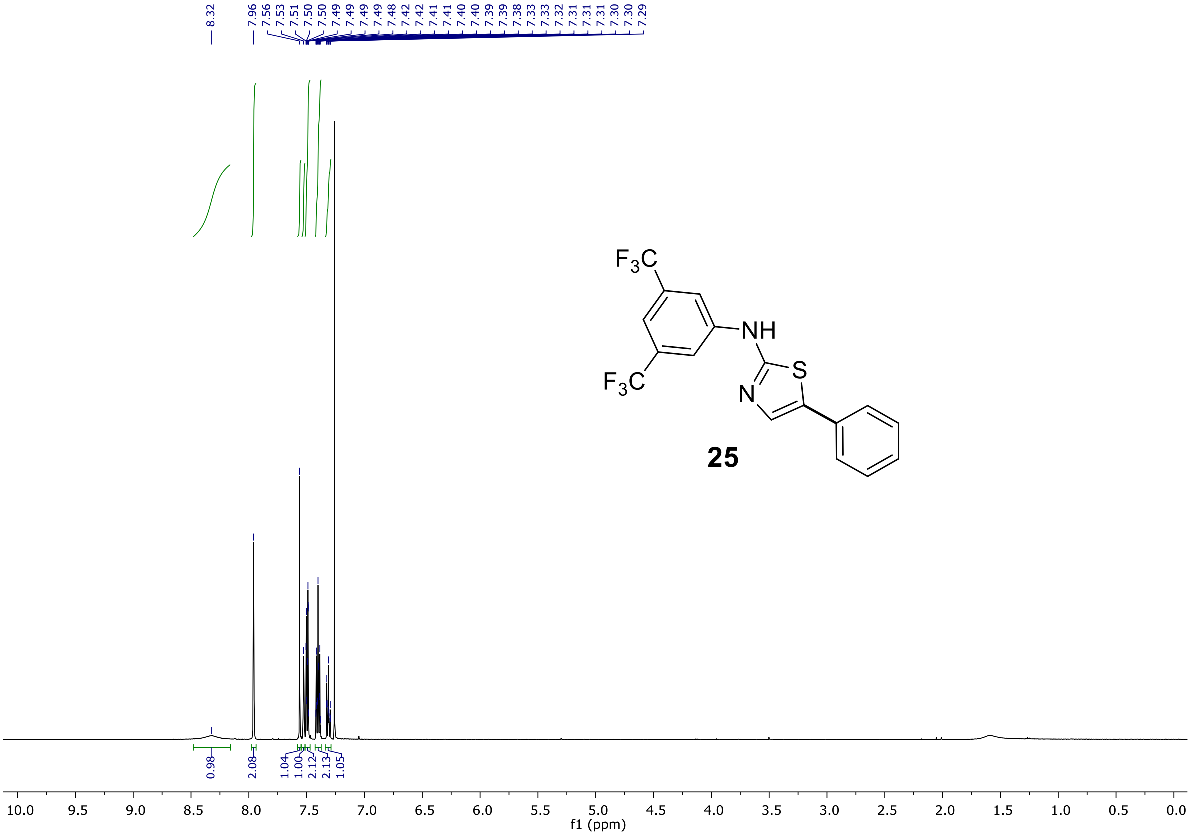

Figure S49. ^{1}H Spectrum of **25** in $CDCl_3$ (500 MHz)

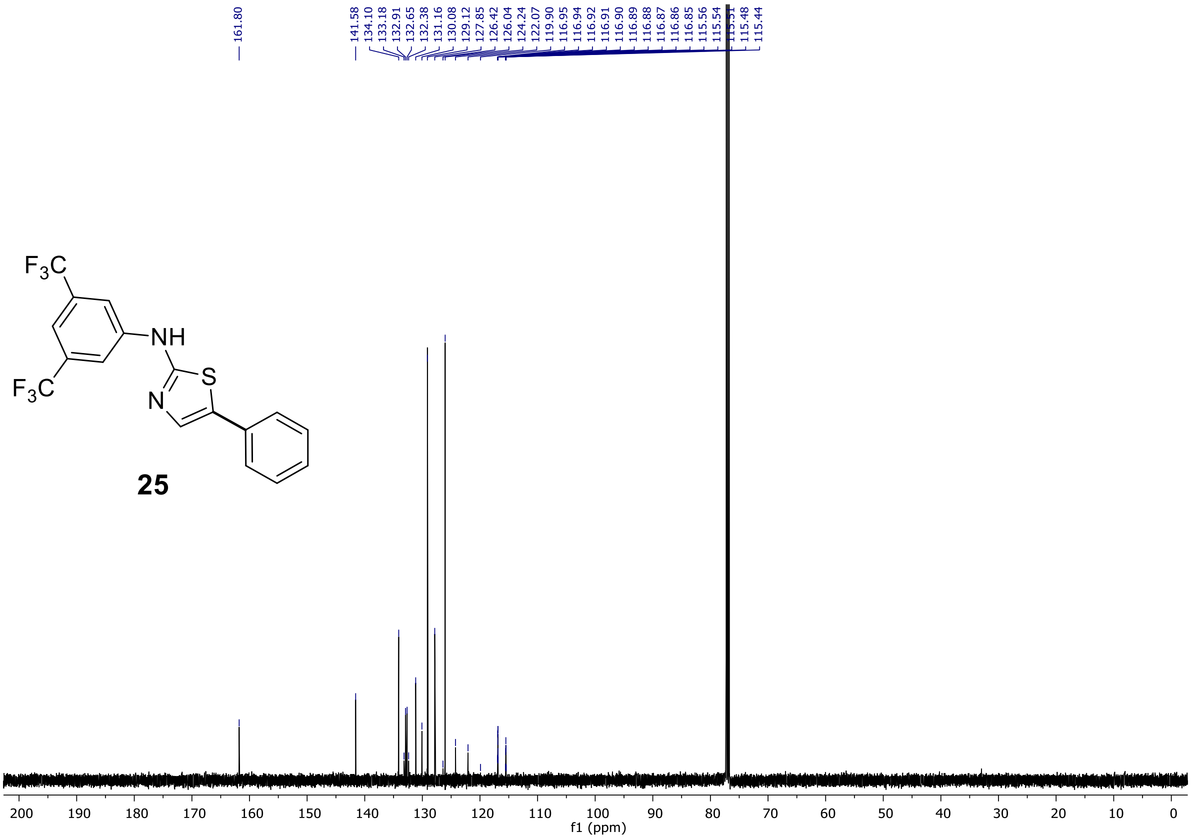

Figure S50. $^{13}C\{^{1}H\}$ Spectrum of **25** in $CDCl_3$ (125 MHz)

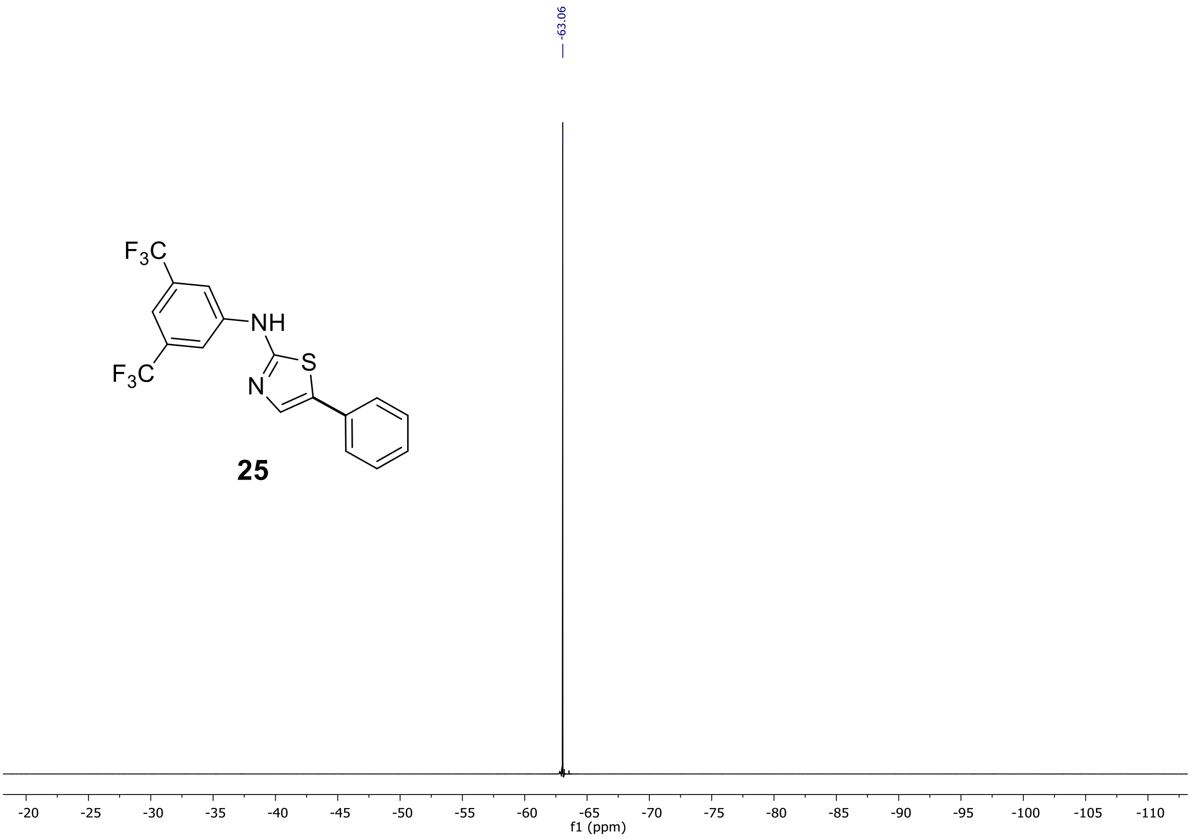

Figure S51. ^{19}F Spectrum of **25** in $CDCl_3$ (376 MHz)

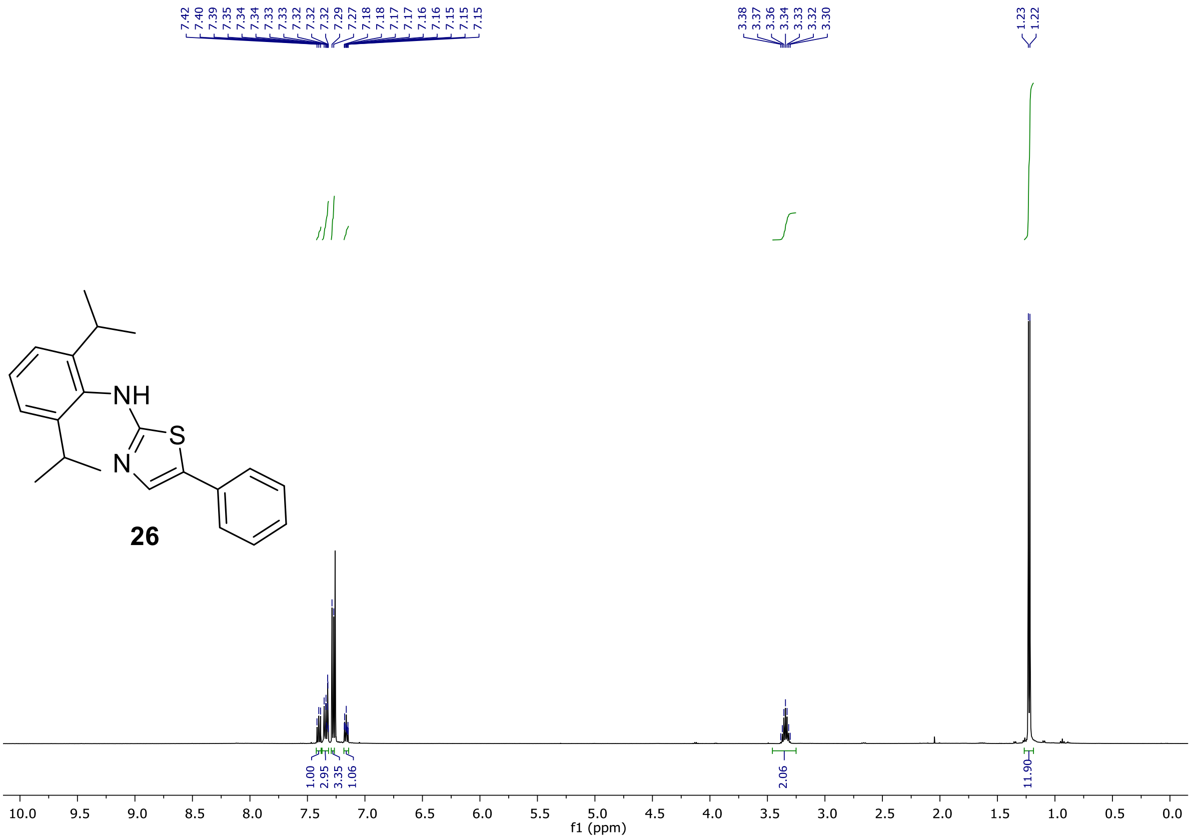

Figure S52. ^{1}H Spectrum of **26** in $CDCl_3$ (400 MHz)

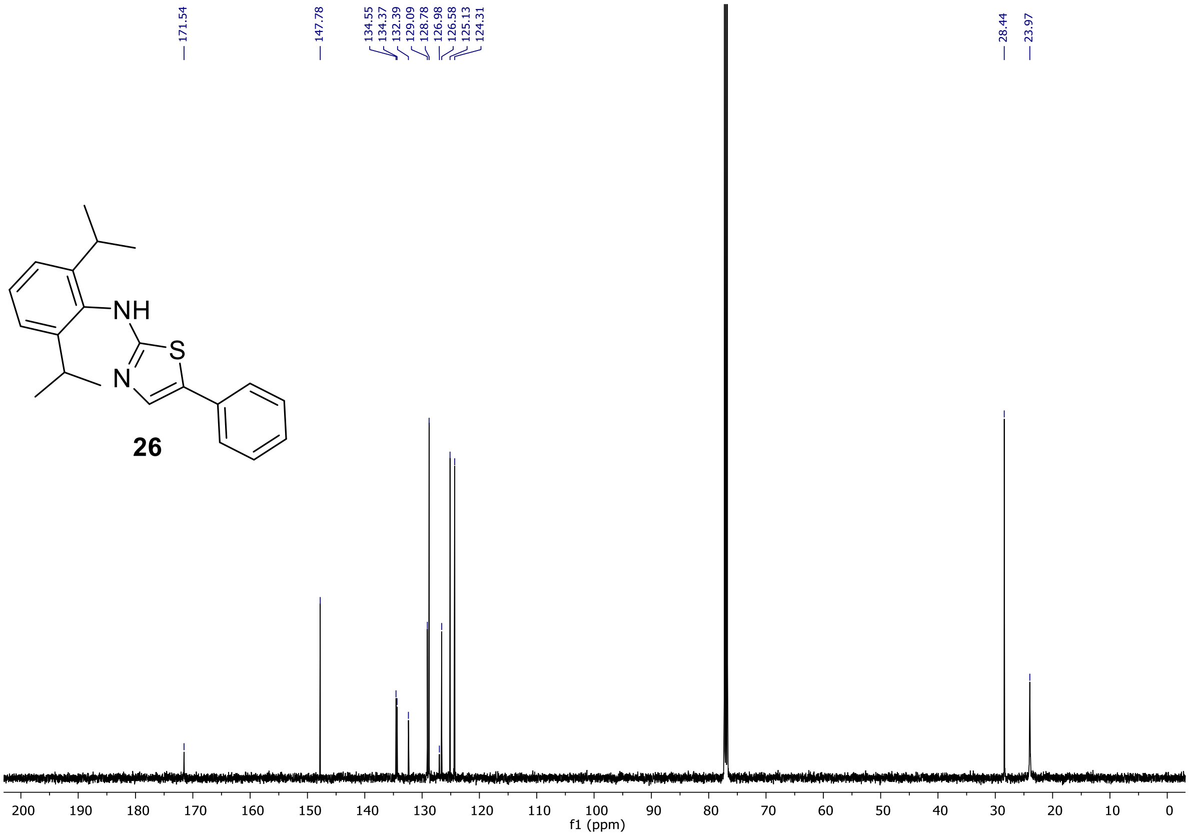

Figure S53. $^{13}C\{^{1}H\}$ Spectrum of **26** in $CDCl_3$ (100 MHz)

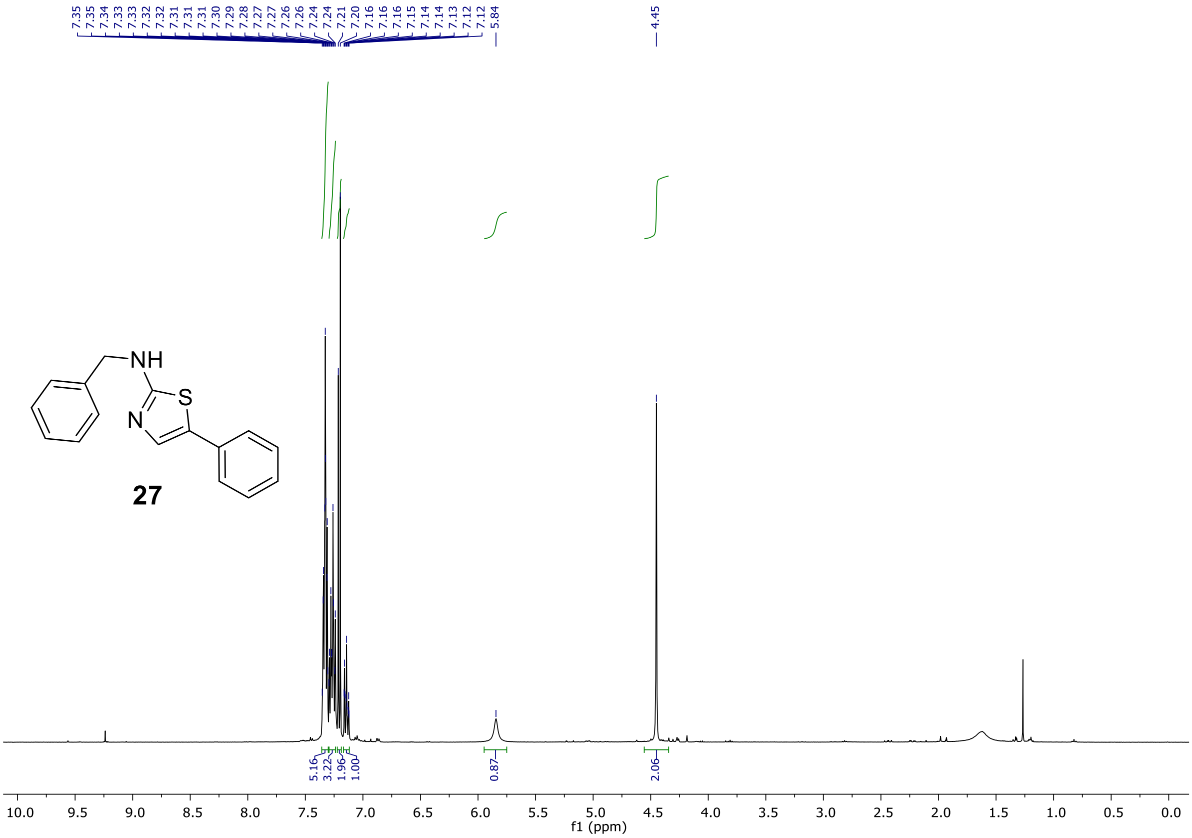

Figure S54. ^{1}H Spectrum of **27** in $CDCl_3$ (400 MHz)

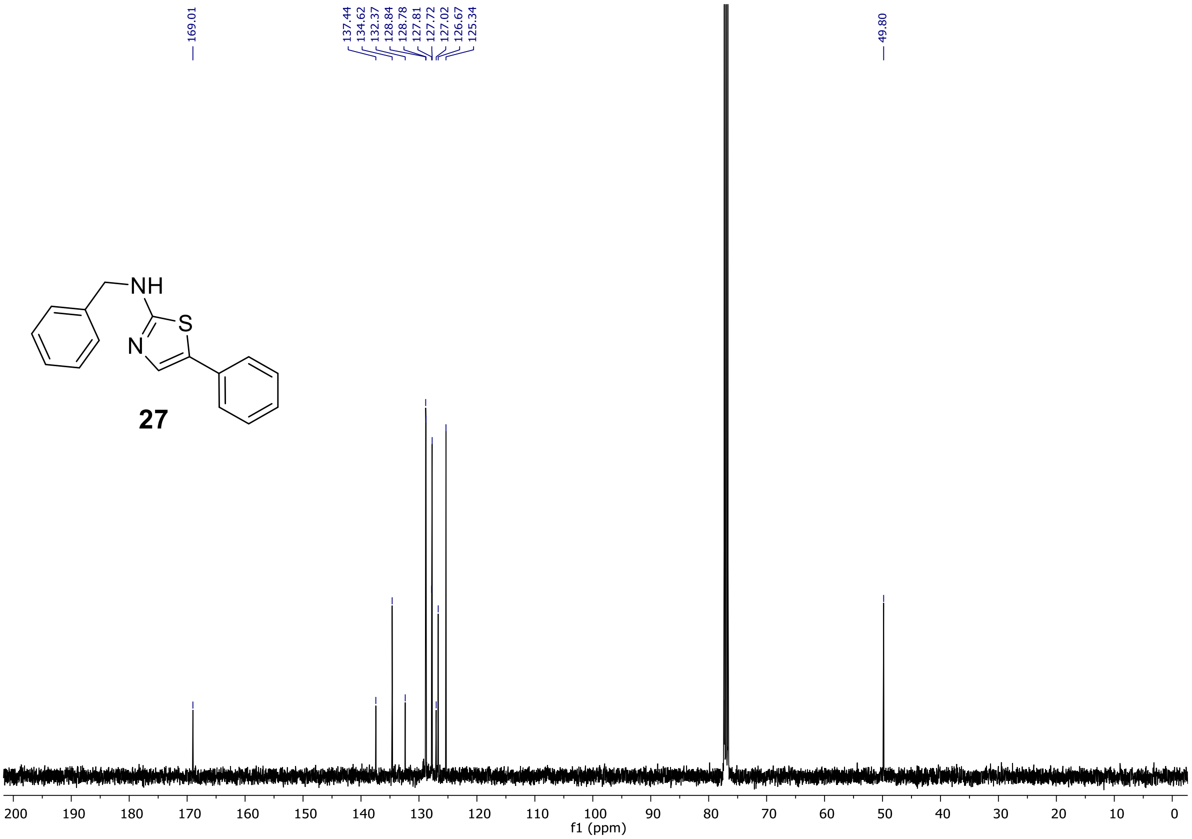

Figure S55. $^{13}C\{^{1}H\}$ Spectrum of **27** in $CDCl_3$ (100 MHz)

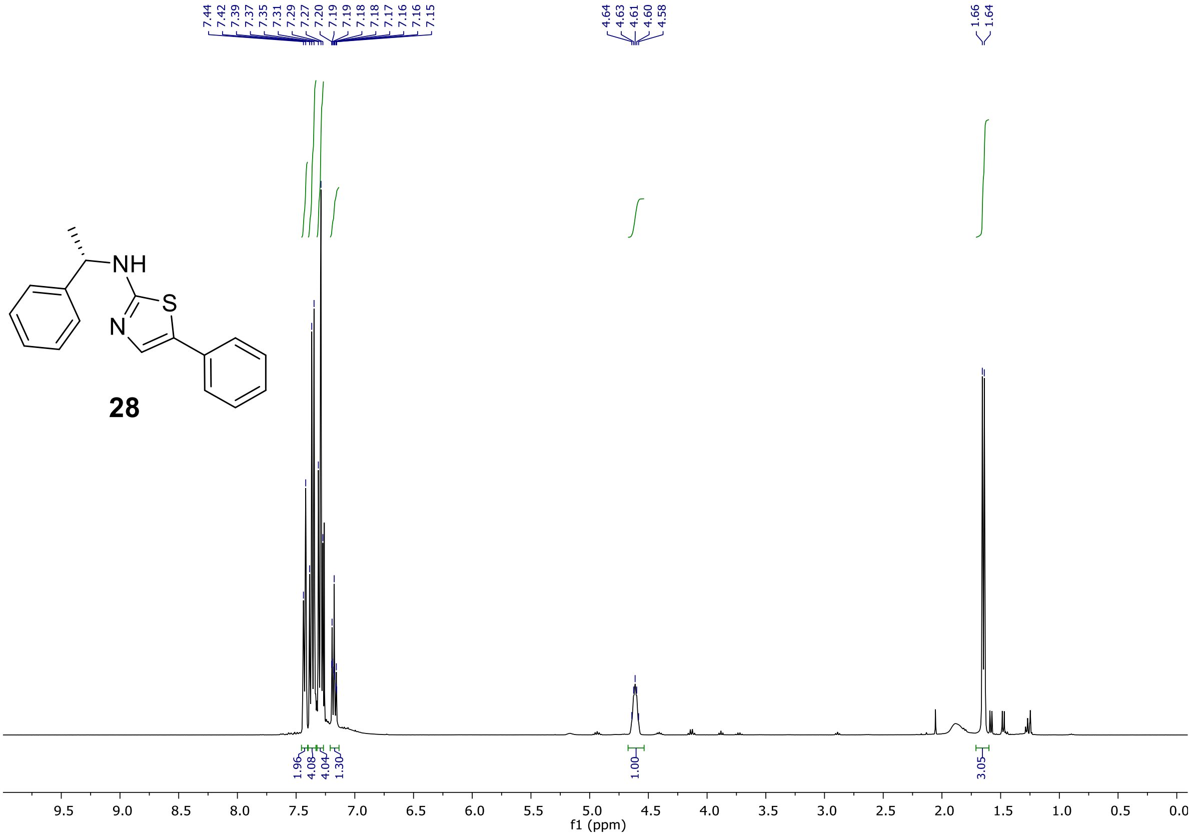

Figure S56. ^{1}H Spectrum of **28** in $CDCl_3$ (400 MHz)

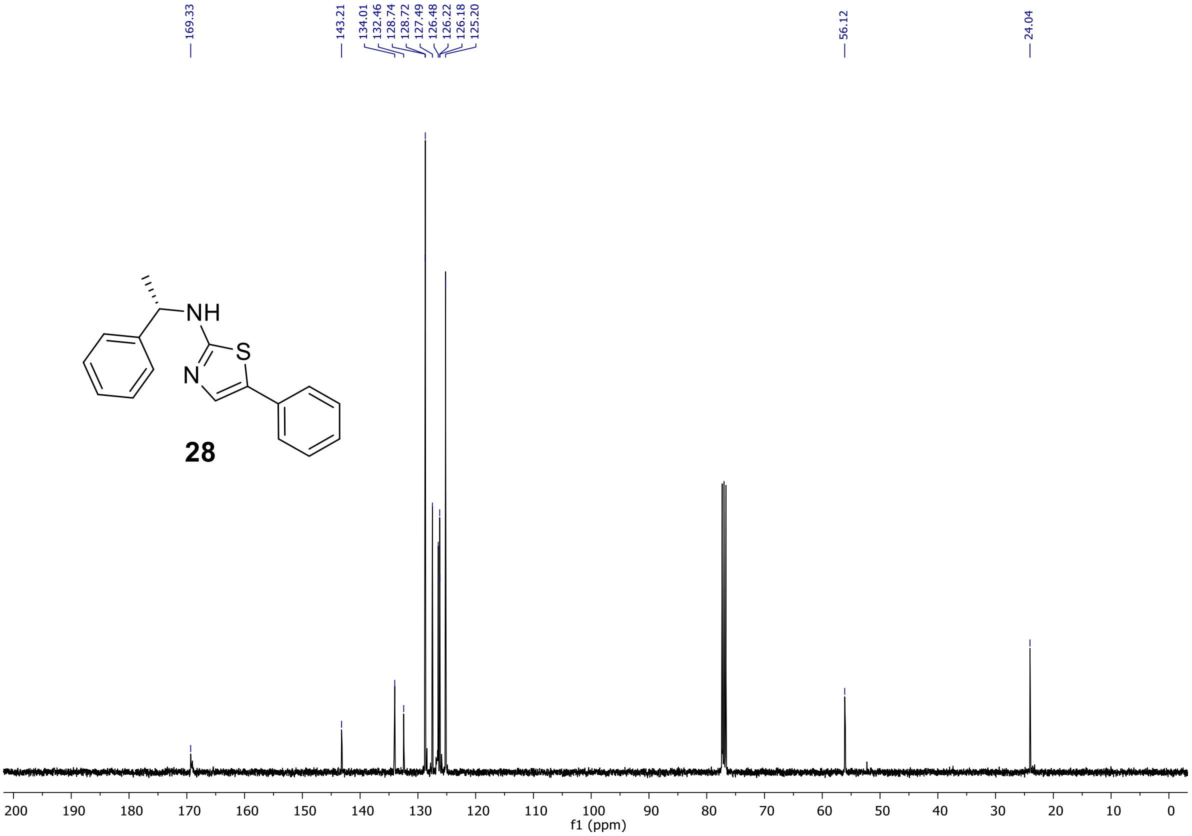

Figure S57. $^{13}C\{^{1}H\}$ Spectrum of **28** in $CDCl_3$ (100 MHz)

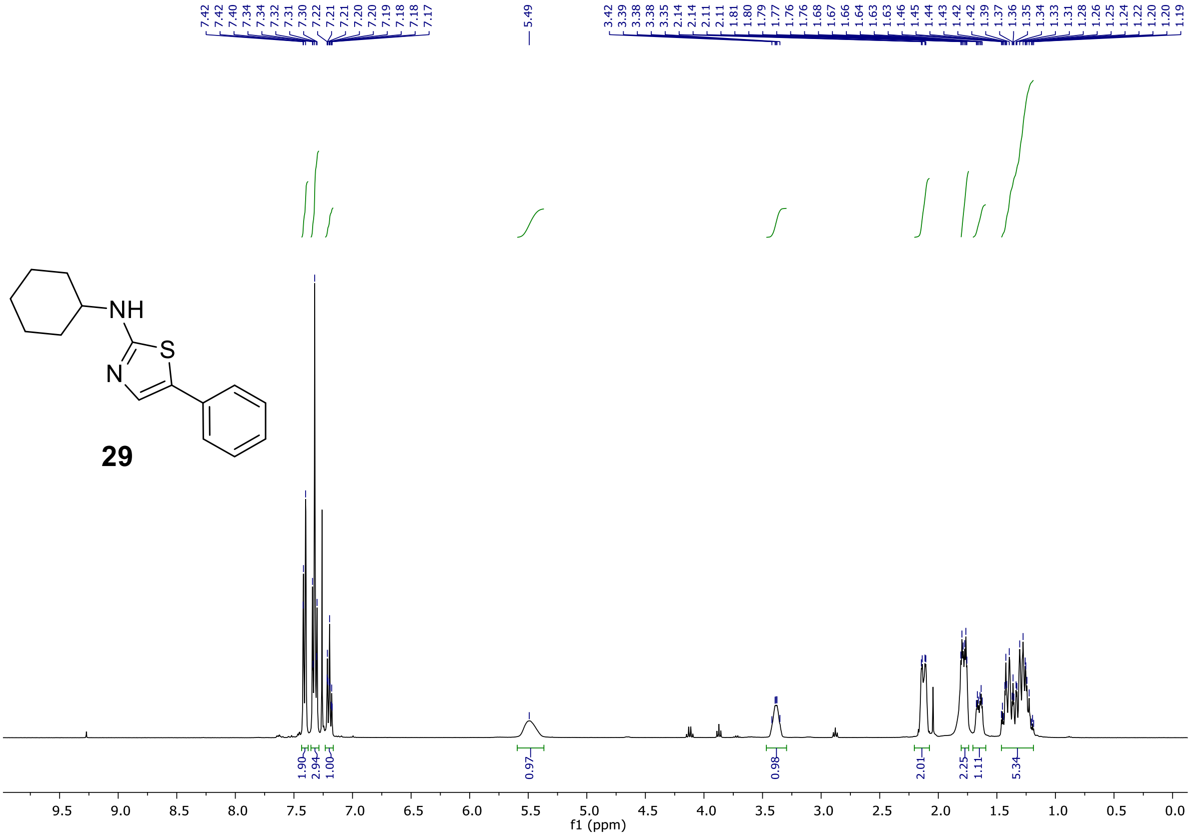

Figure S58. ^{1}H Spectrum of **29** in $CDCl_3$ (400 MHz)

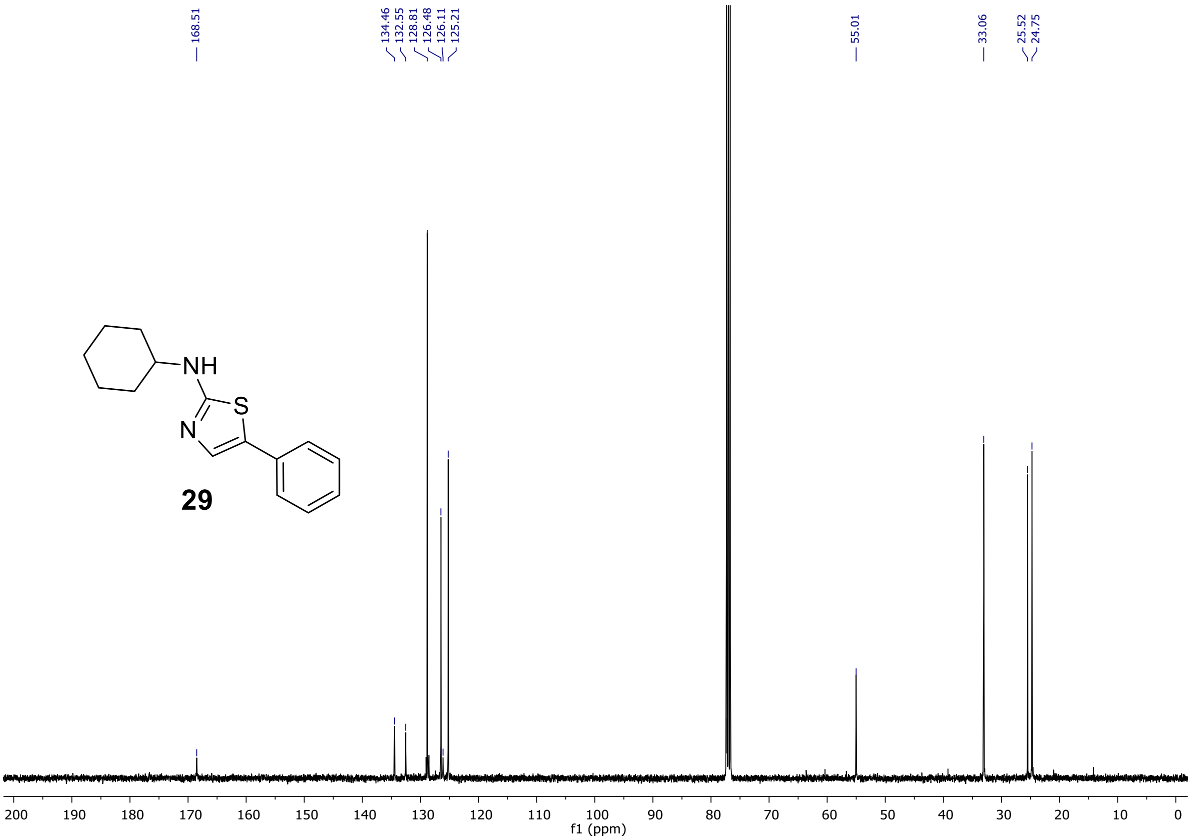

Figure S59. $^{13}C\{^{1}H\}$ Spectrum of **29** in $CDCl_3$ (100 MHz)

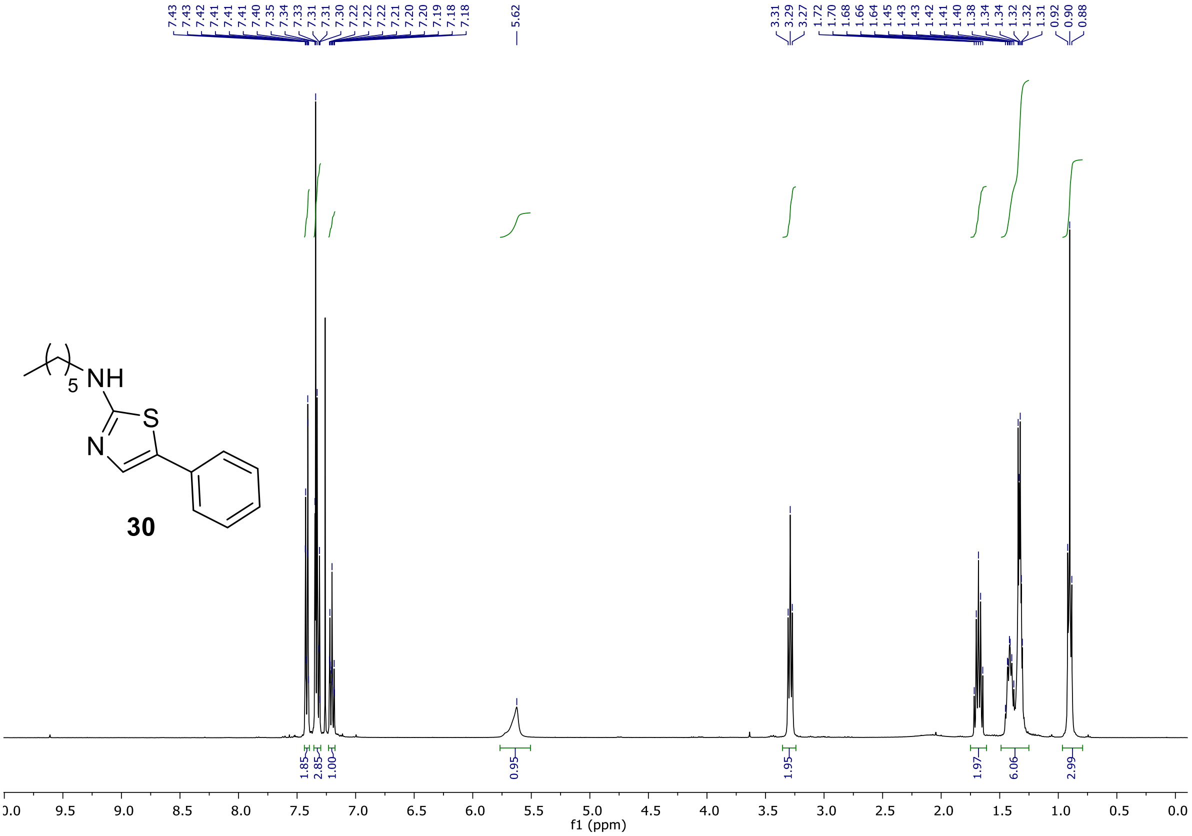

Figure S60. [1]H Spectrum of **30** in $CDCl_3$ (400 MHz)

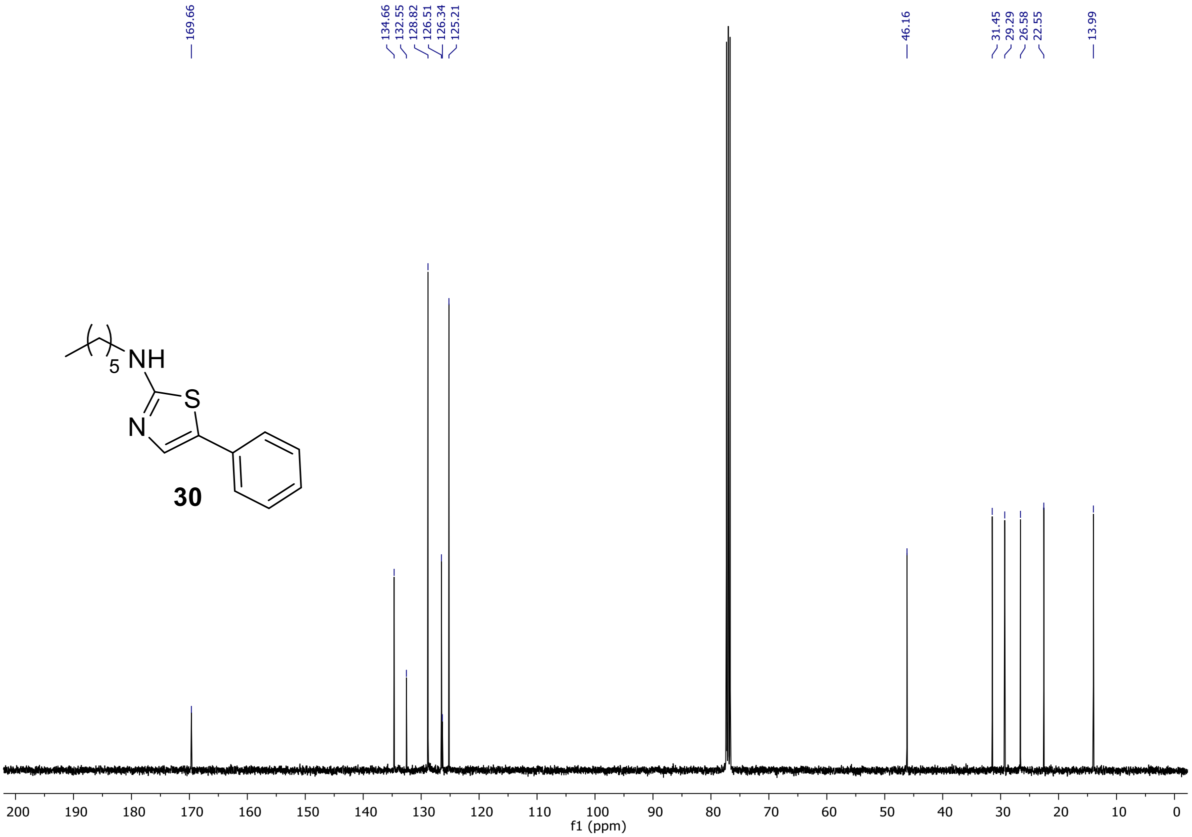

Figure S61. ^{13}C{^{1}H} Spectrum of **30** in $CDCl_3$ (100 MHz)

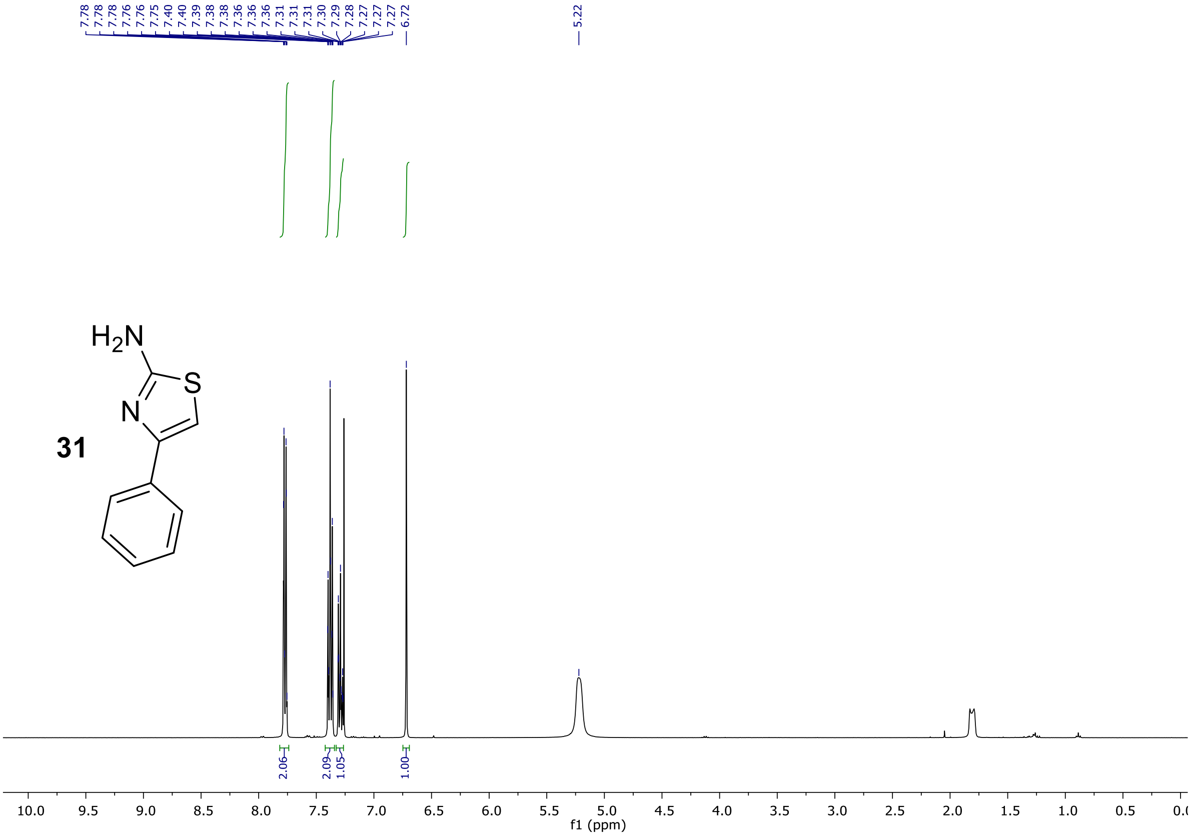

Figure S62. [1]H Spectrum of **31** in $CDCl_3$ (400 MHz)

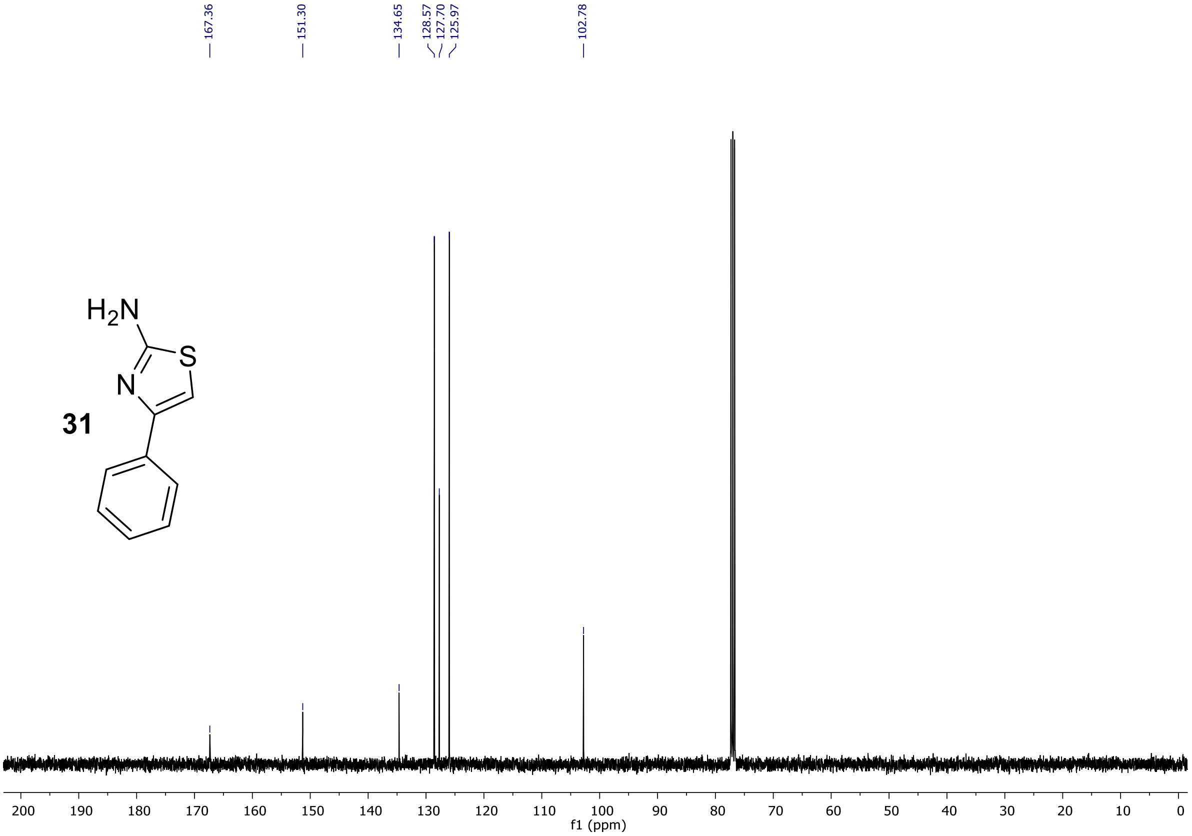

Figure S63. ^{13}C{^{1}H} Spectrum of **31** in $CDCl_3$ (125 MHz)

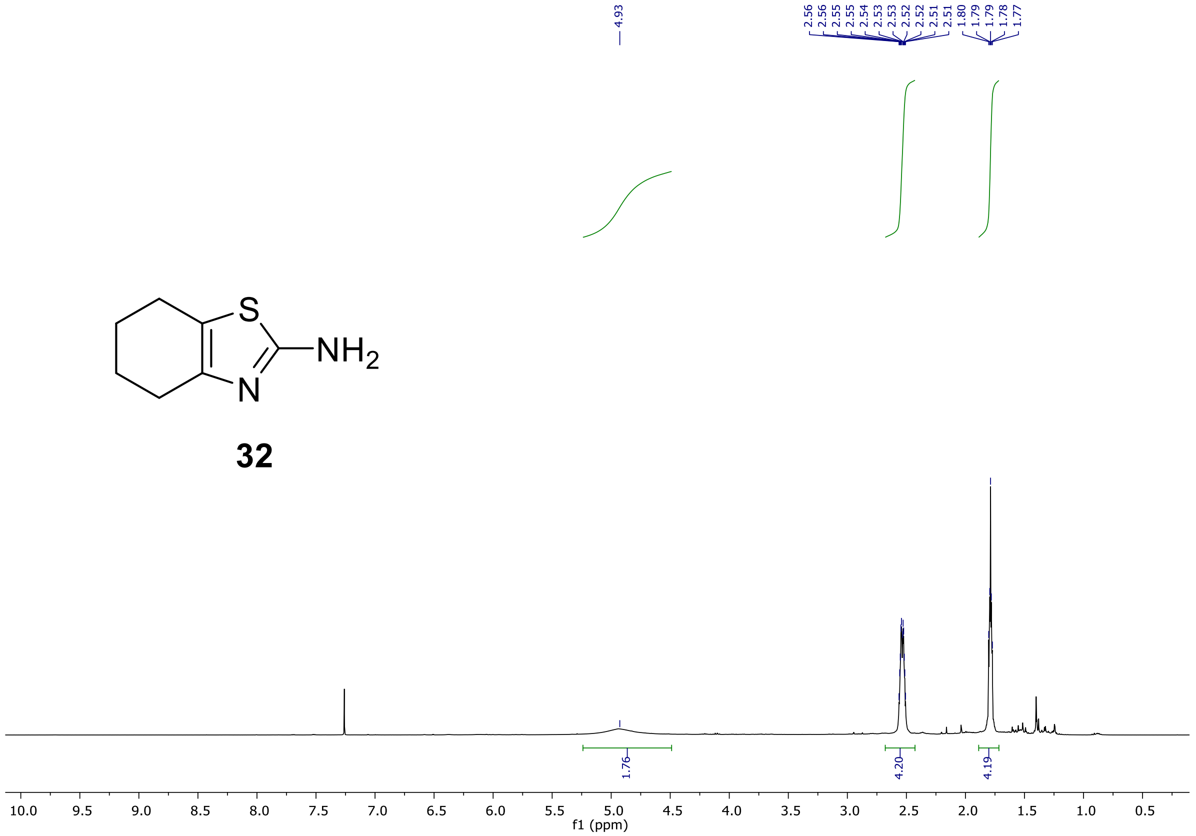

Figure S64. ^{1}H Spectrum of **32** in $CDCl_3$ (400 MHz)

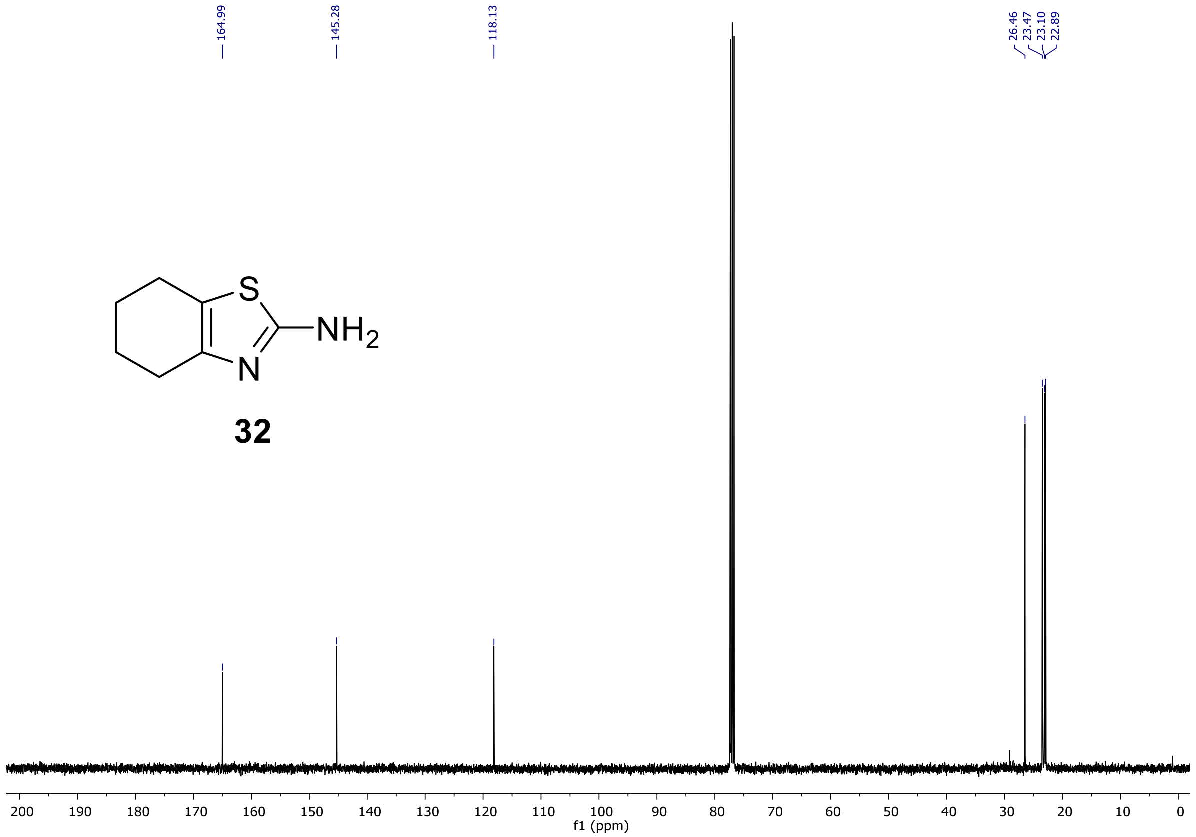

Figure S65. $^{13}C\{^{1}H\}$ Spectrum of **32** in $CDCl_3$ (100 MHz)

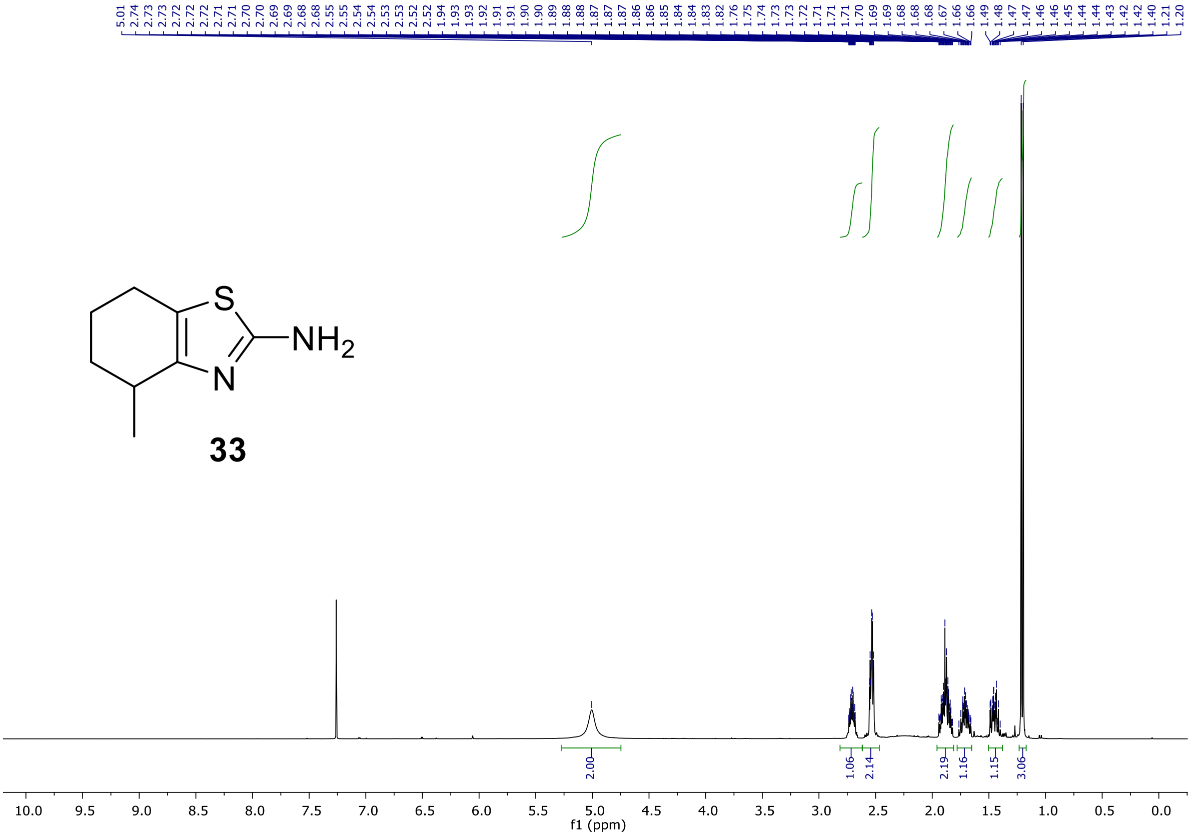

Figure S66. ^{1}H Spectrum of **33** in $CDCl_3$ (400 MHz)

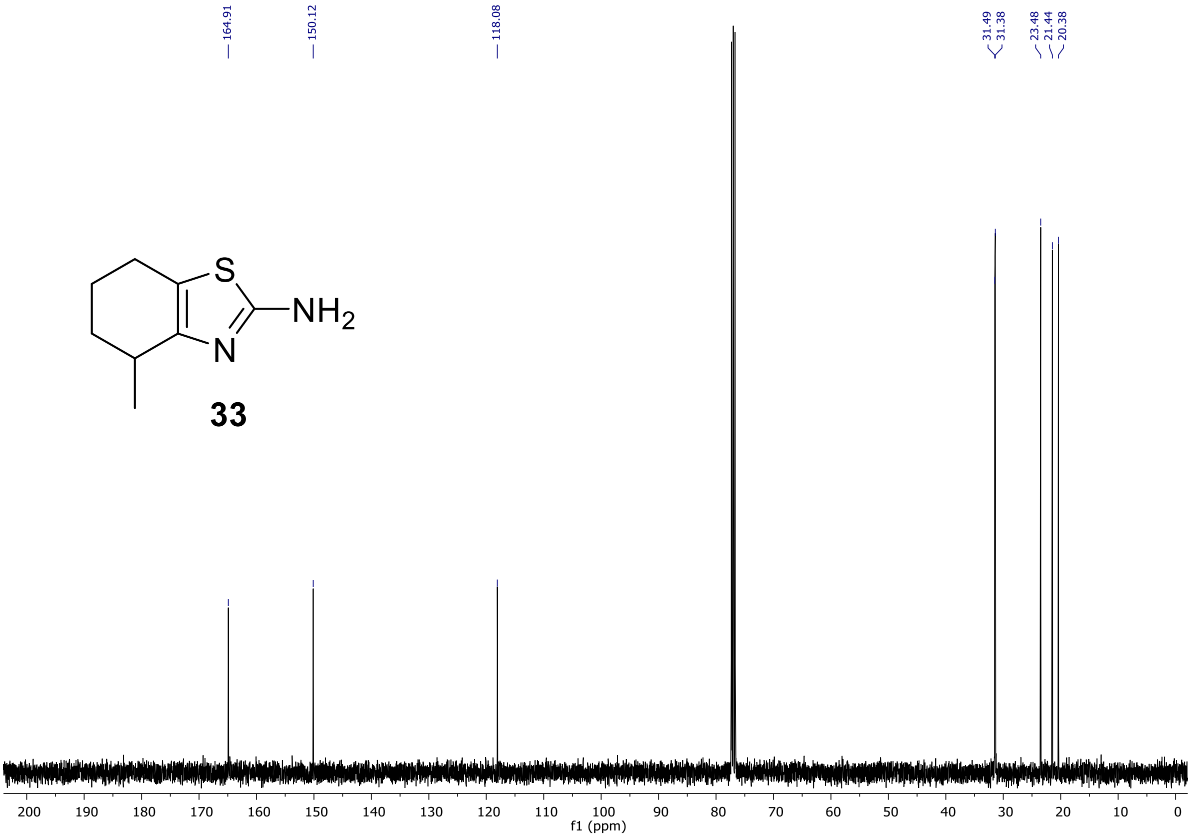

Figure S67. $^{13}C\{^{1}H\}$ Spectrum of **33** in $CDCl_3$ (100 MHz)

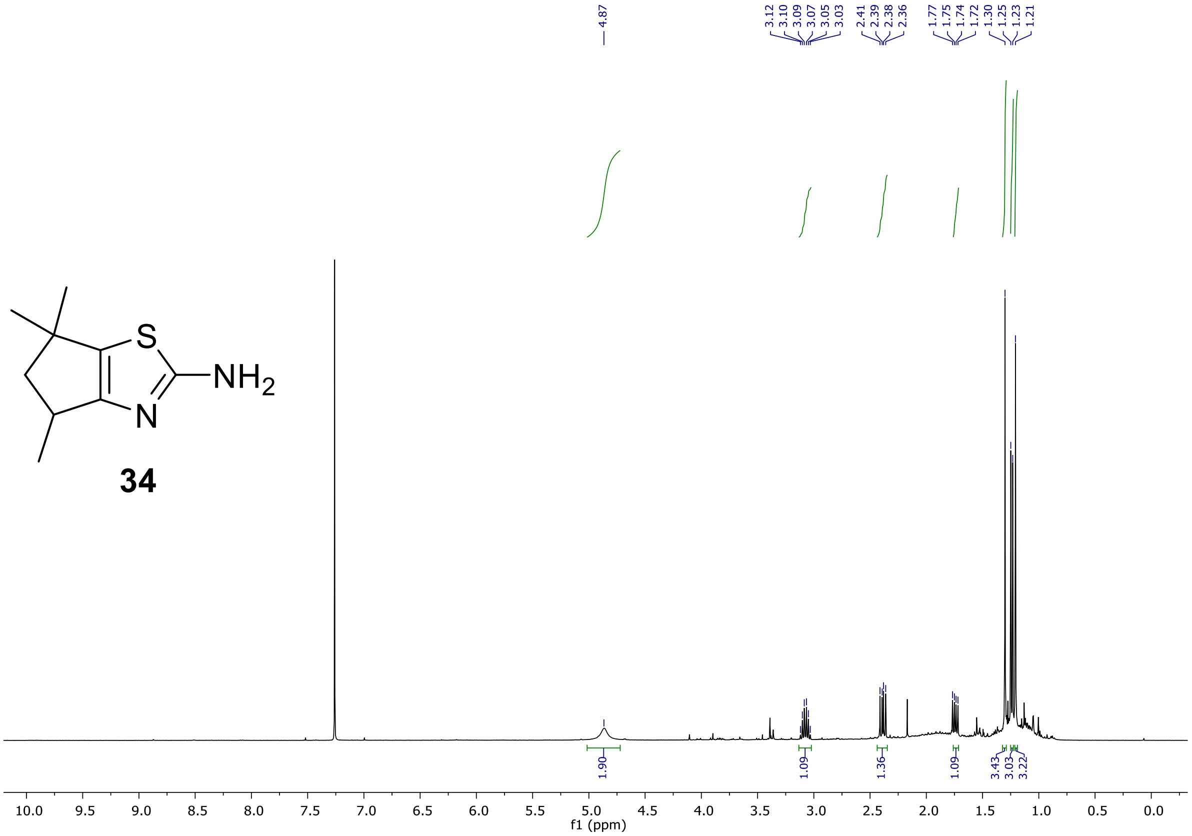

Figure S68. ^{1}H Spectrum of **34** in $CDCl_3$ (500 MHz)

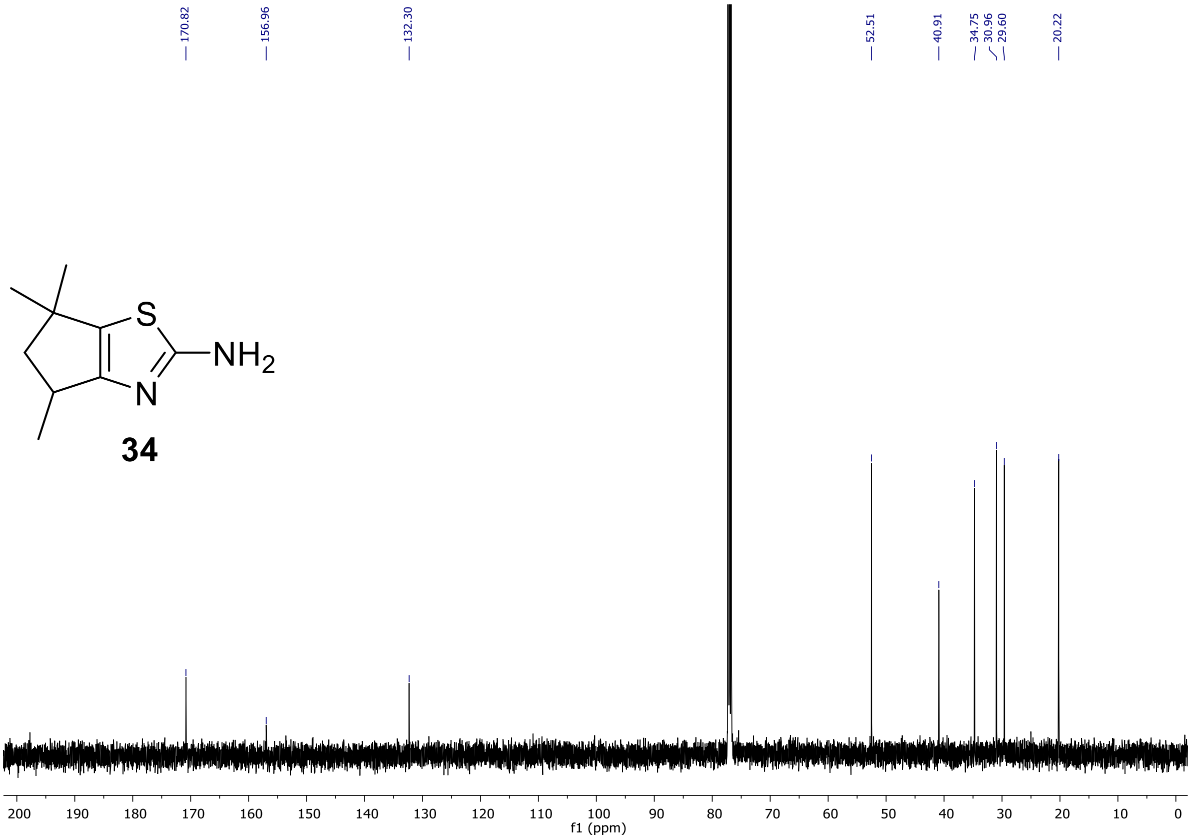

Figure S69. $^{13}C\{^{1}H\}$ Spectrum of **34** in $CDCl_3$ (125 MHz)

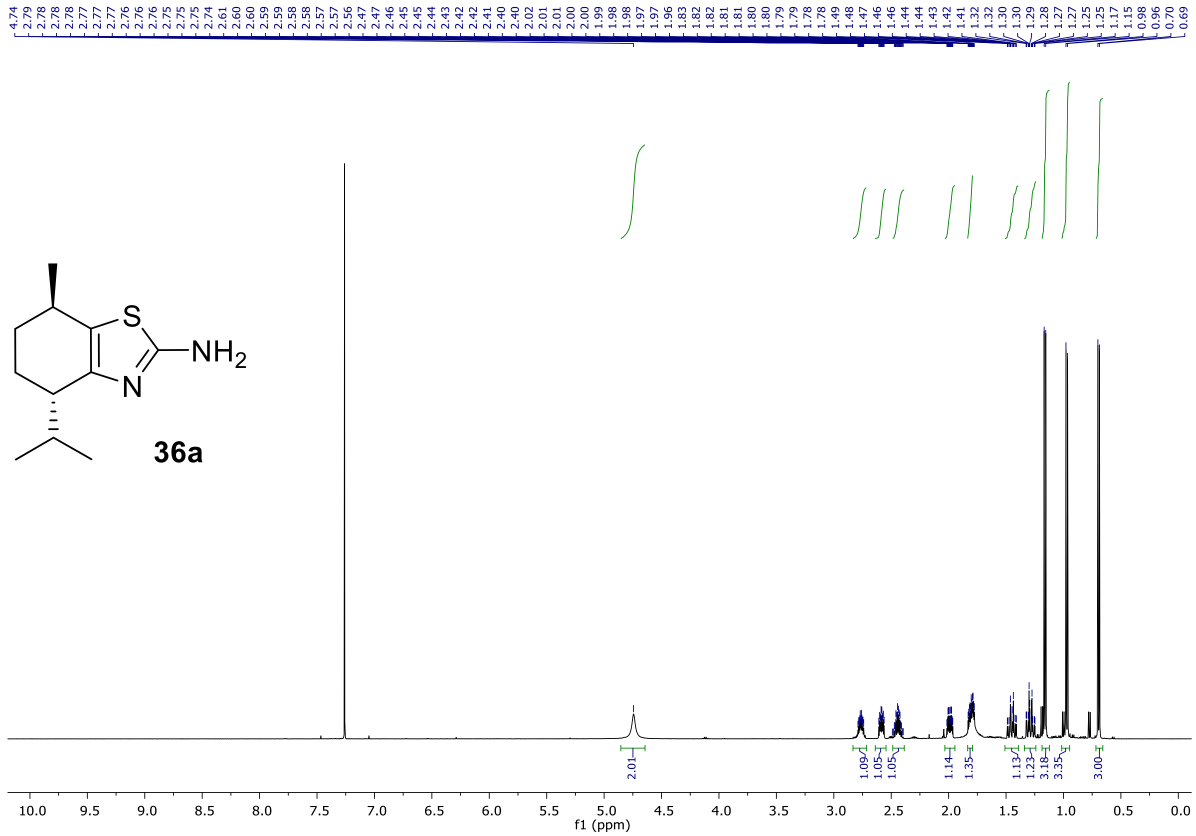

Figure S70. ^{1}H Spectrum of **36a** in $CDCl_3$ (500 MHz)

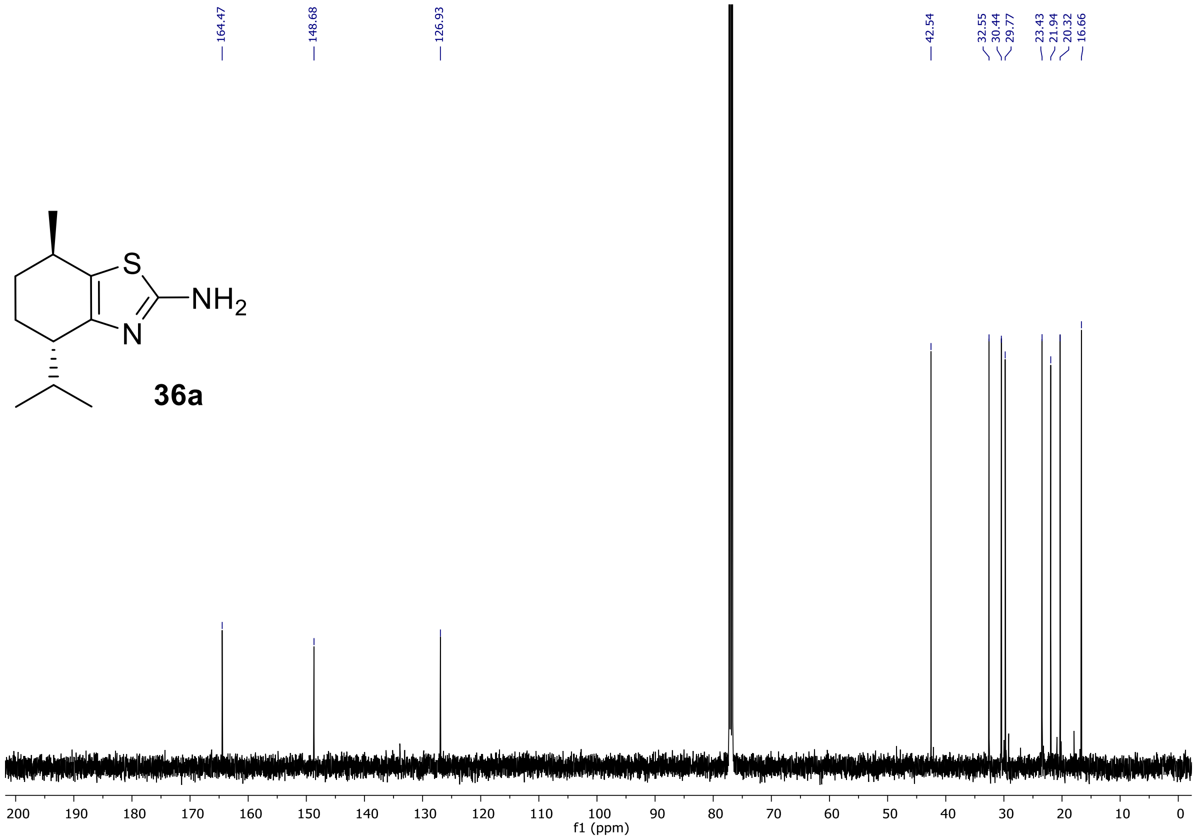

Figure S71. $^{13}C\{^{1}H\}$ Spectrum of **36a** in $CDCl_3$ (125 MHz)

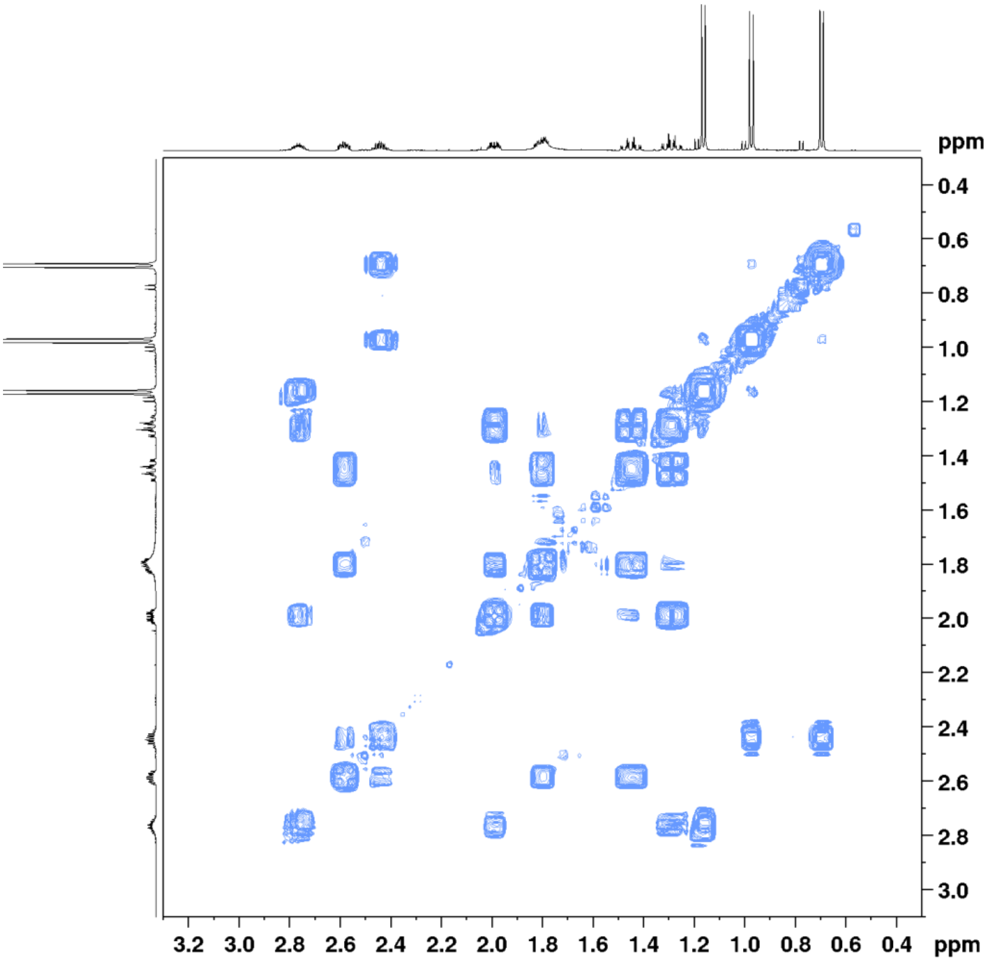

Figure S72. ^{1}H-^{1}H COSY Spectrum of **36a.**

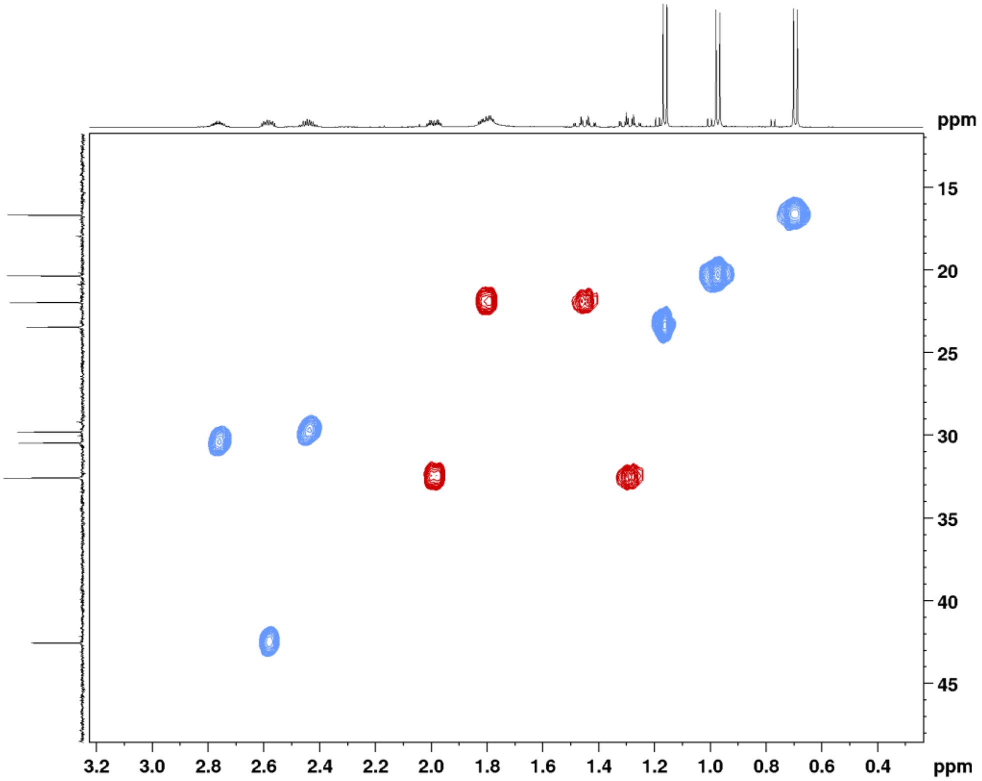

Figure S73. HSQC Spectrum of **36a**.

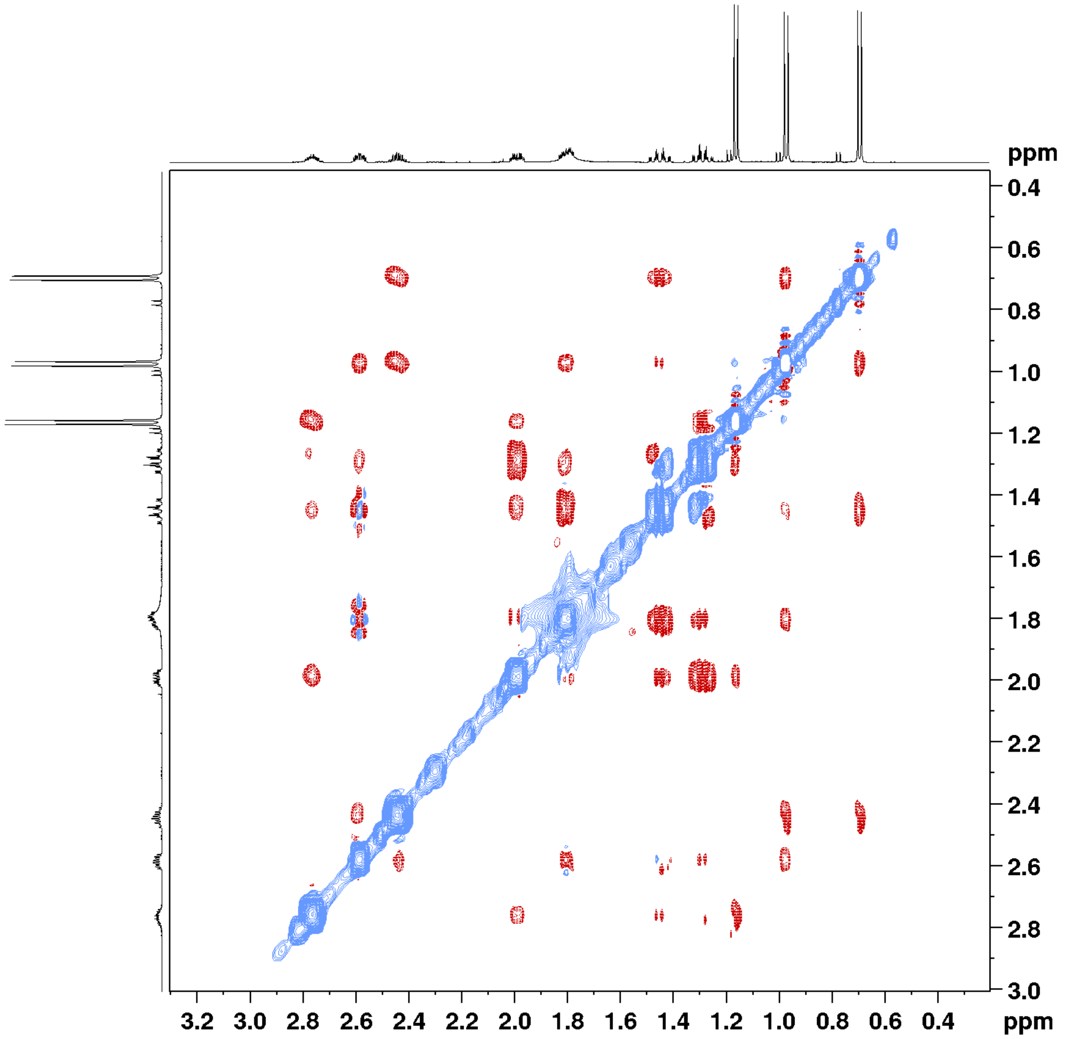

Figure S74. 2D ^{1}H-^{1}H NOESY Spectrum of **36a.**

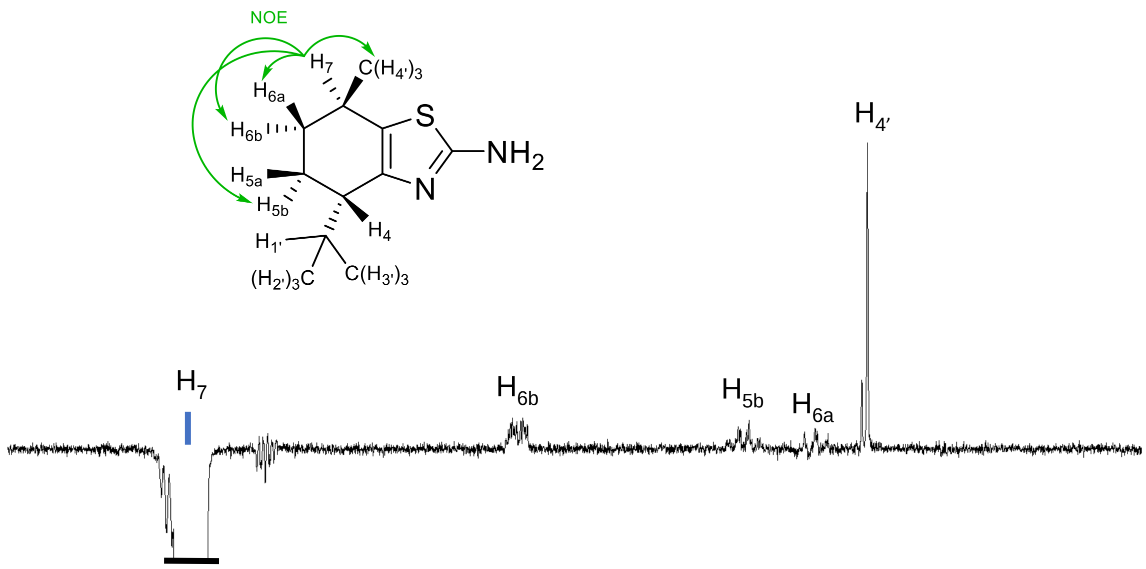

Figure S75. 1D Selective ¹H NOE NMR Spectrum of H_7 (2.76 ppm) in diastereomer **36a** in $CDCl_3$ (500 MHz).

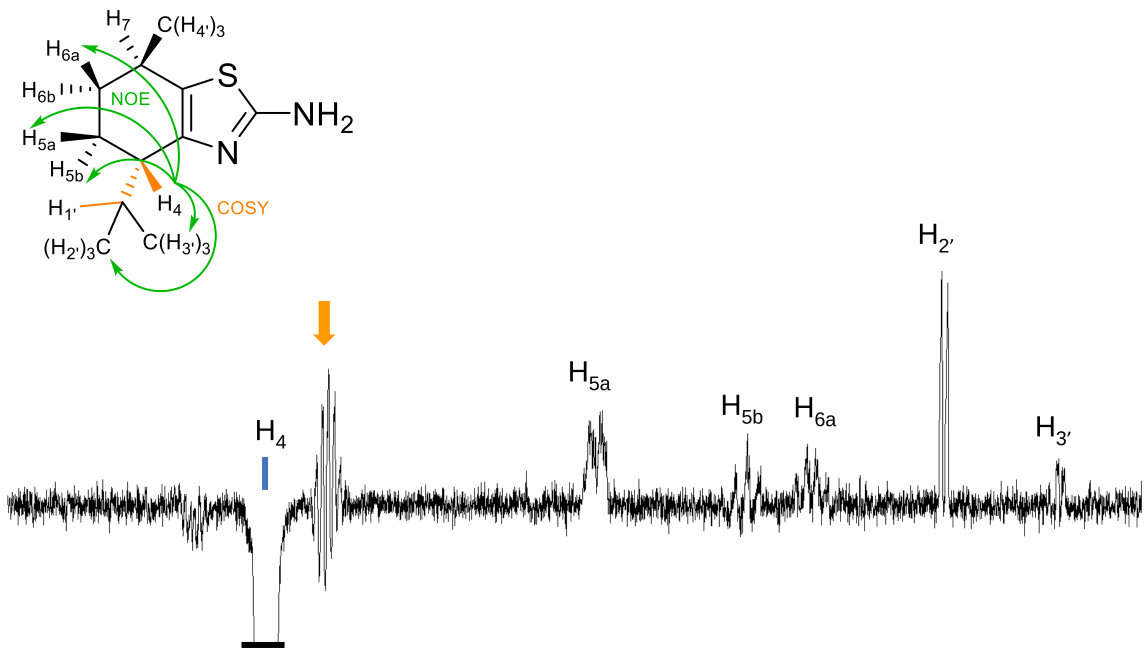

Figure S76. 1D Selective ¹H NOE NMR Spectrum of H_4 (2.59 ppm) in diastereomer **36a** in $CDCl_3$ (500 MHz).

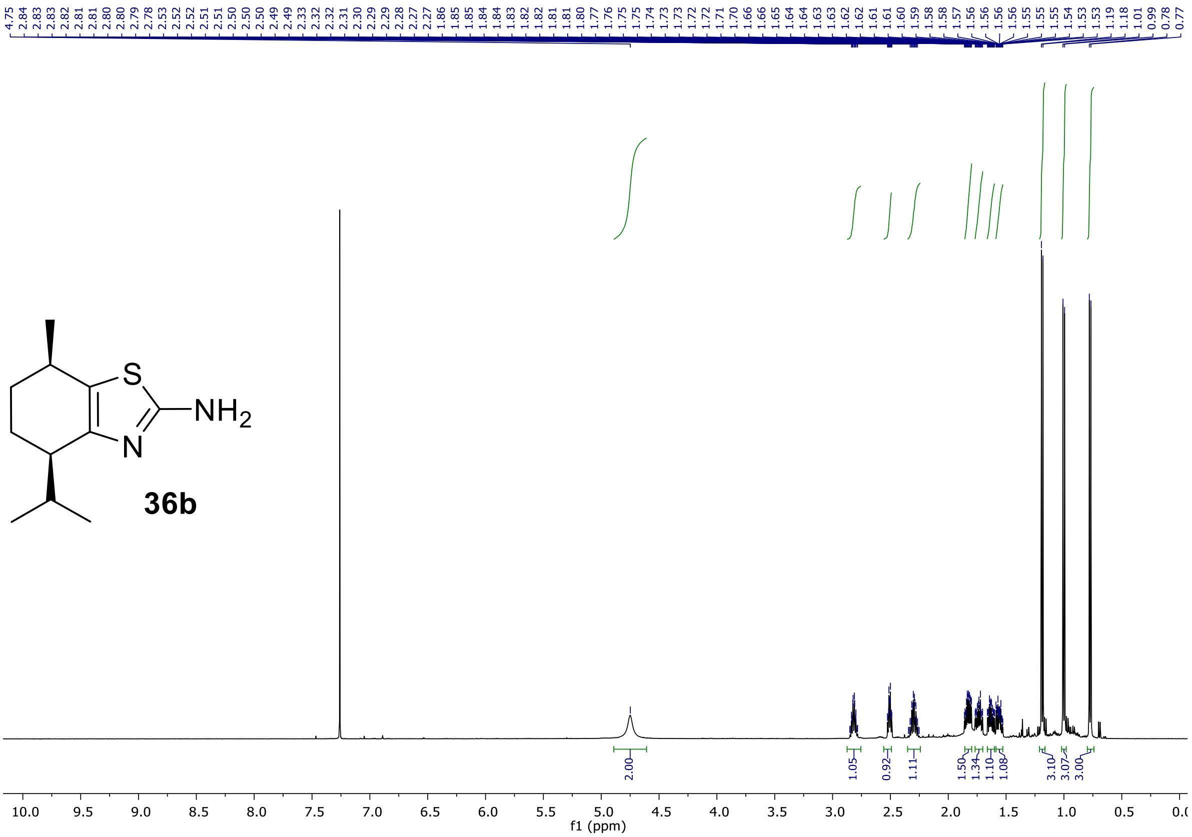

Figure S77. ^{1}H Spectrum of **36b** in $CDCl_3$ (500 MHz)

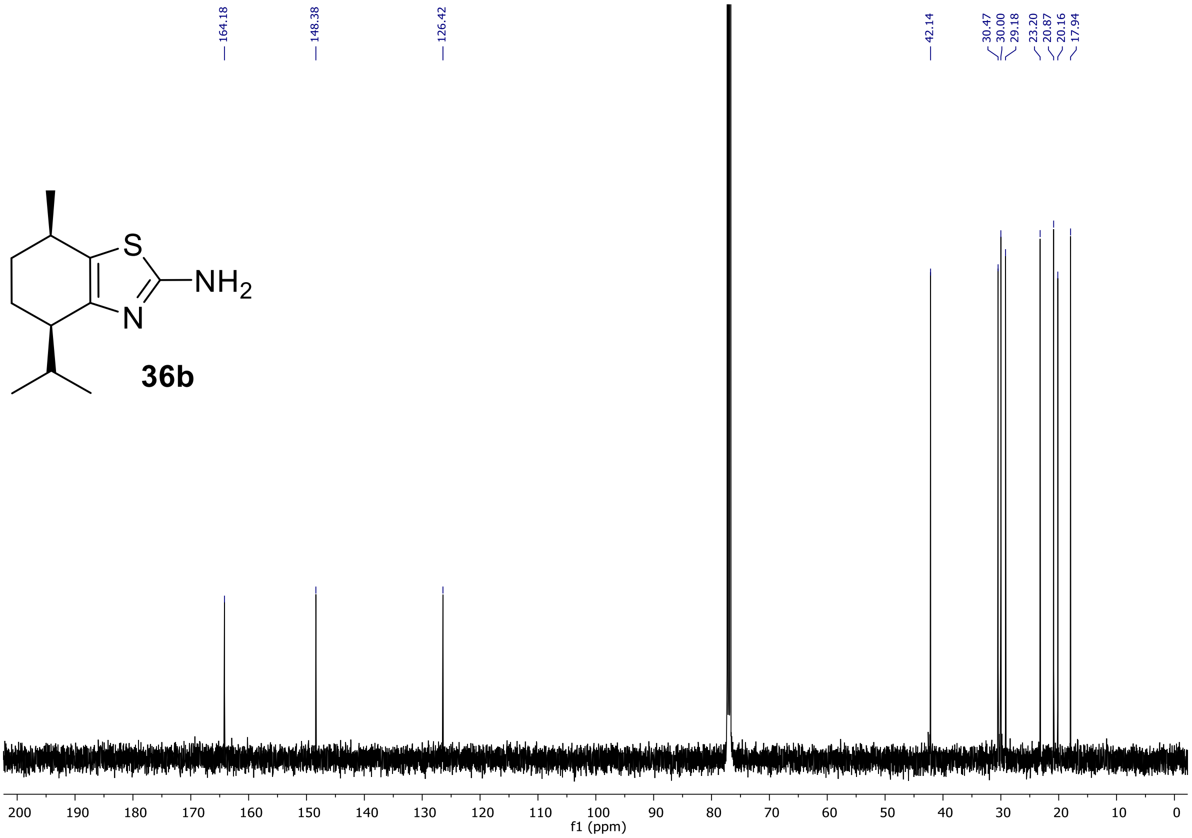

Figure S78. $^{13}C\{^{1}H\}$ Spectrum of **36b** in $CDCl_3$ (125 MHz)

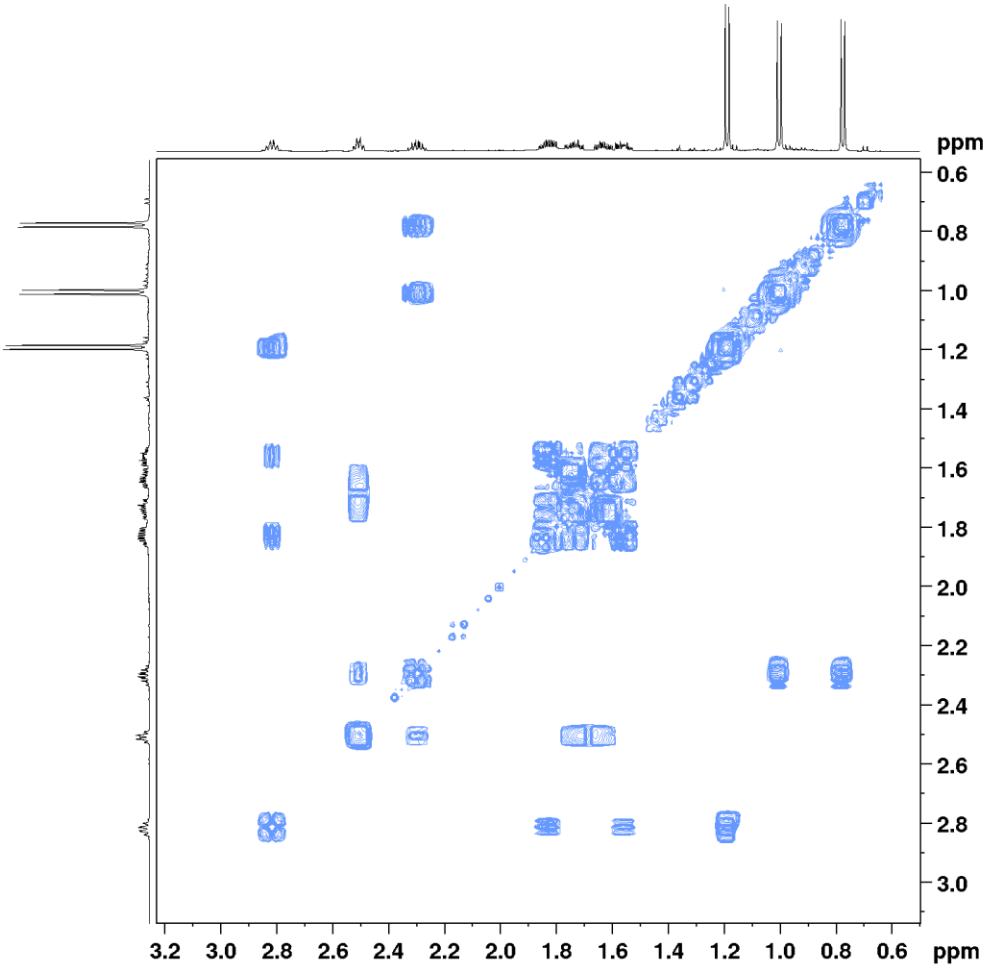

Figure S79. ^{1}H-^{1}H COSY Spectrum of **36b.**

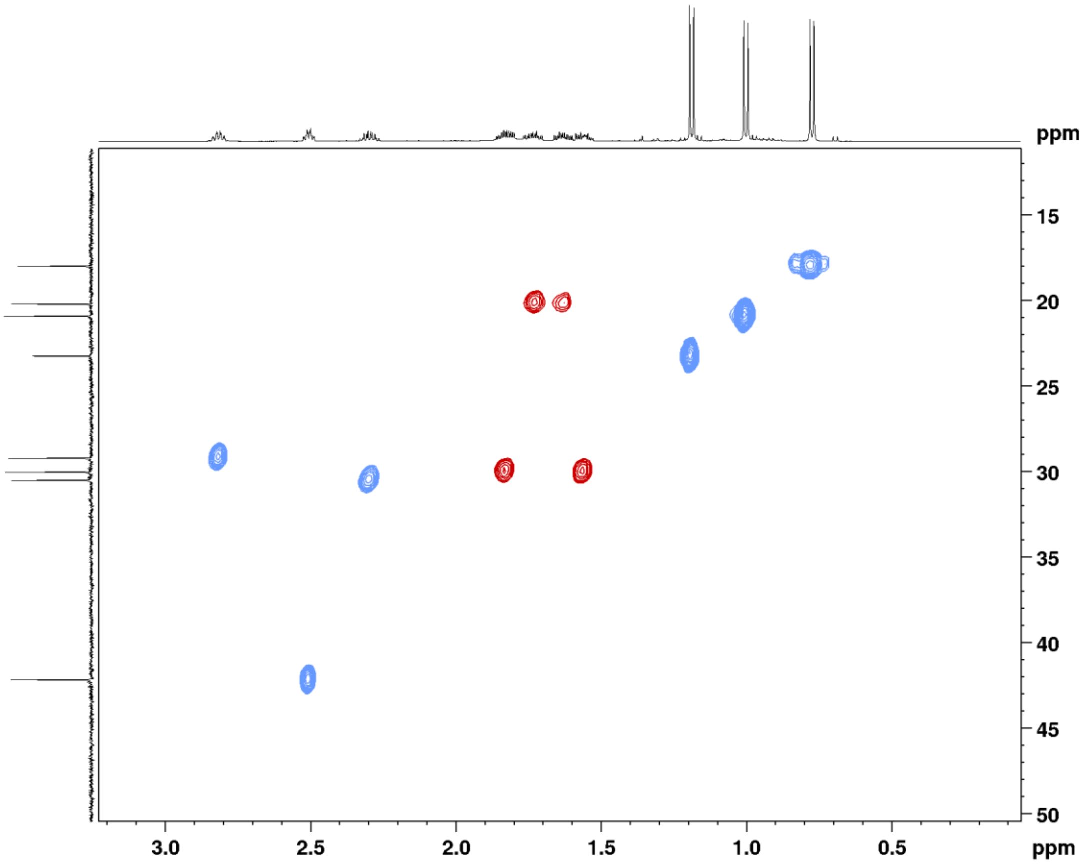

Figure S80. HSQC Spectrum of **36b**.

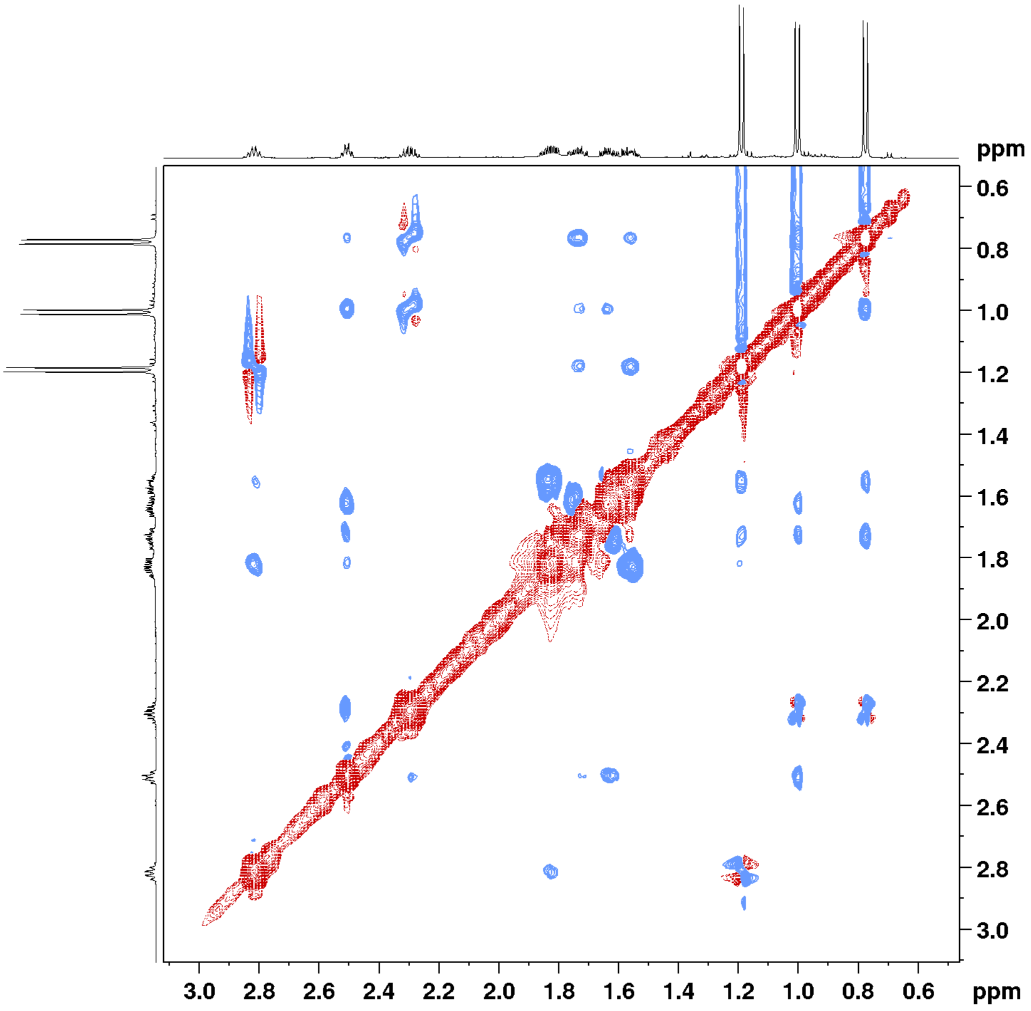

Figure S81. 2D ^{1}H-^{1}H NOESY Spectrum of **36b.**

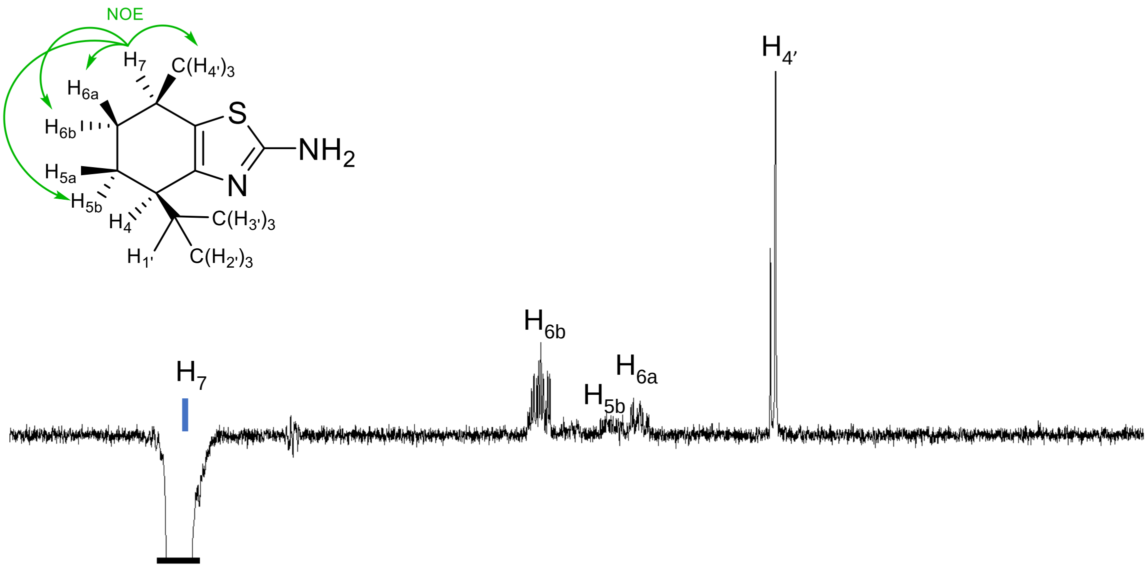

Figure S82. 1D Selective ^{1}H NOE NMR Spectrum of H_7 (2.82 ppm) in diastereomer **36b** in $CDCl_3$ (500 MHz).

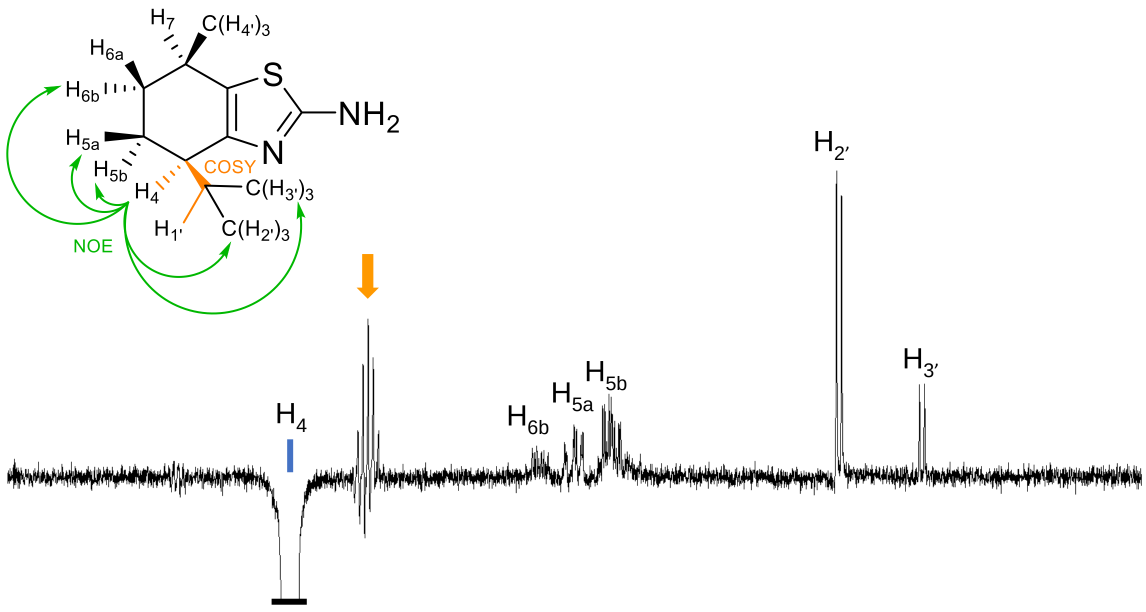

Figure S83. 1D Selective ^{1}H NOE NMR Spectrum of H_4 (2.51 ppm) in diastereomer **36b** in $CDCl_3$ (500 MHz).

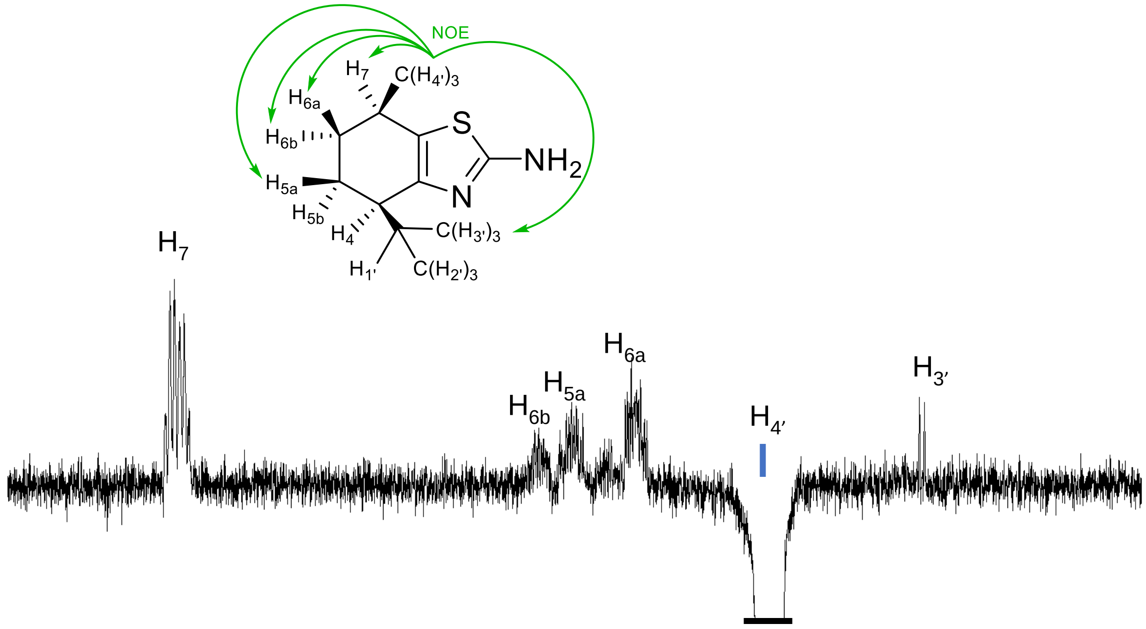

Figure S84. 1D Selective ^{1}H NOE NMR Spectrum of $H_{4'}$ (1.19 ppm) in diastereomer **36b** in $CDCl_3$ (500 MHz).

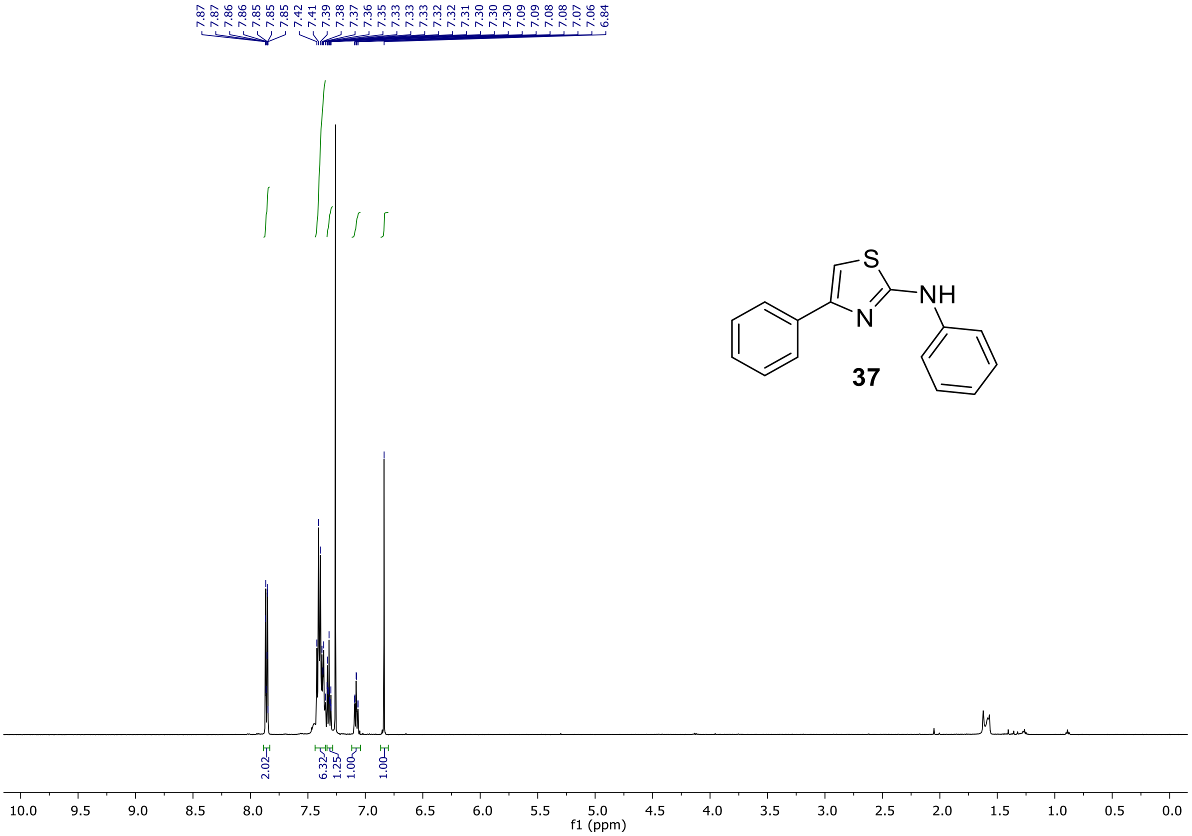

Figure S85. ^{1}H Spectrum of **37** in $CDCl_3$ (500 MHz)

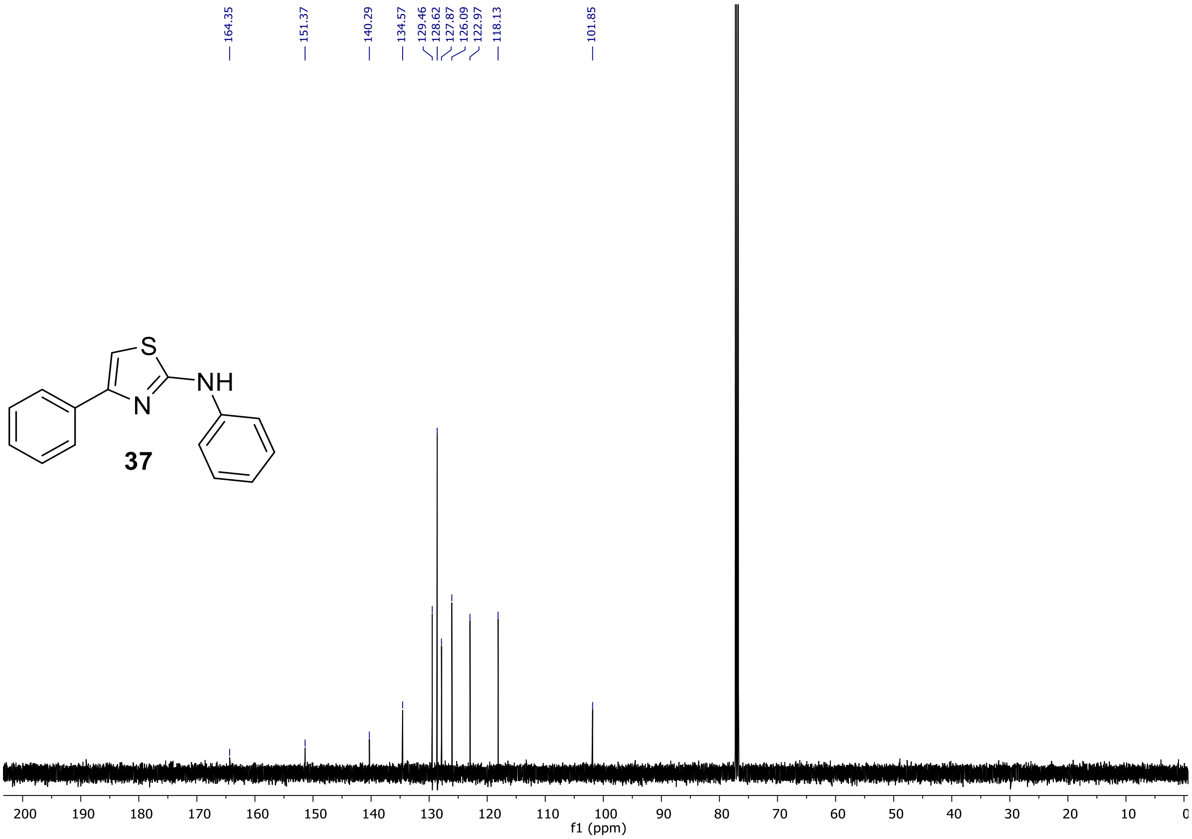

Figure S86. $^{13}C\{^{1}H\}$ Spectrum of **37** in $CDCl_3$ (125 MHz)

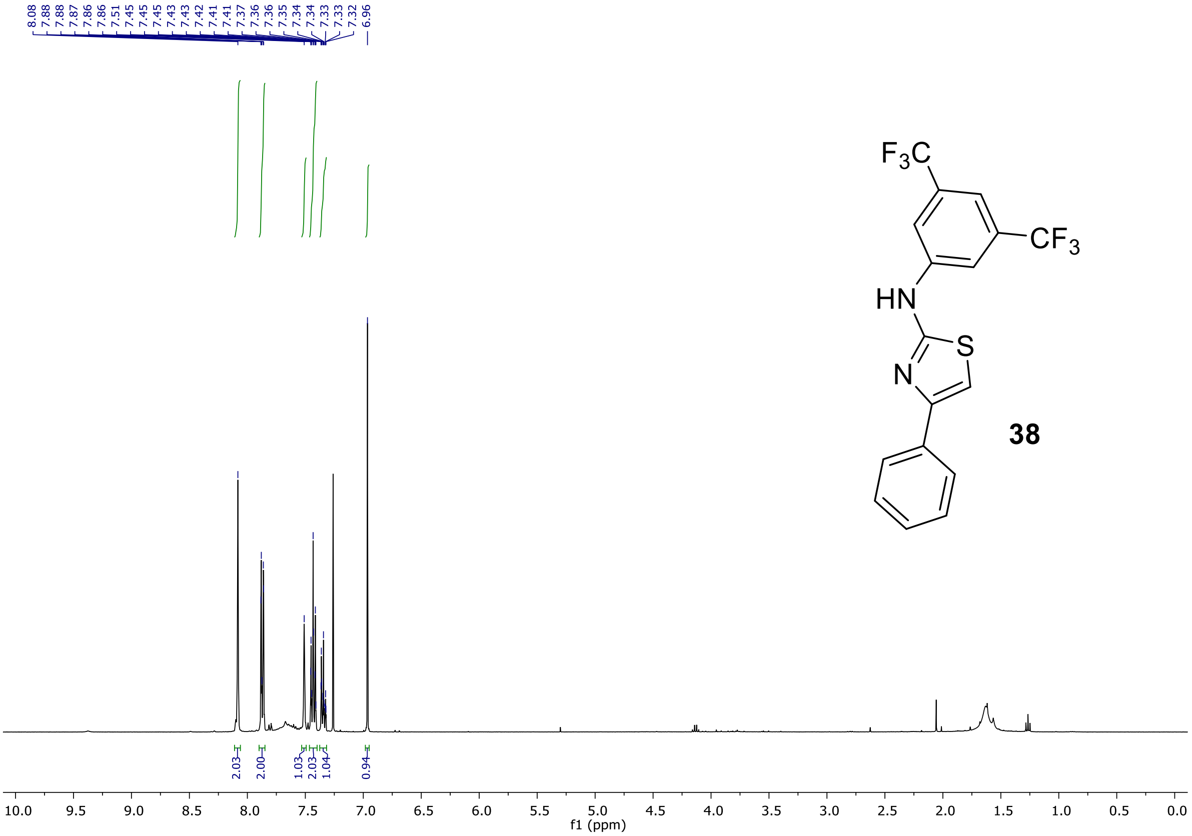

Figure S87. ^{1}H Spectrum of **38** in $CDCl_3$ (400 MHz)

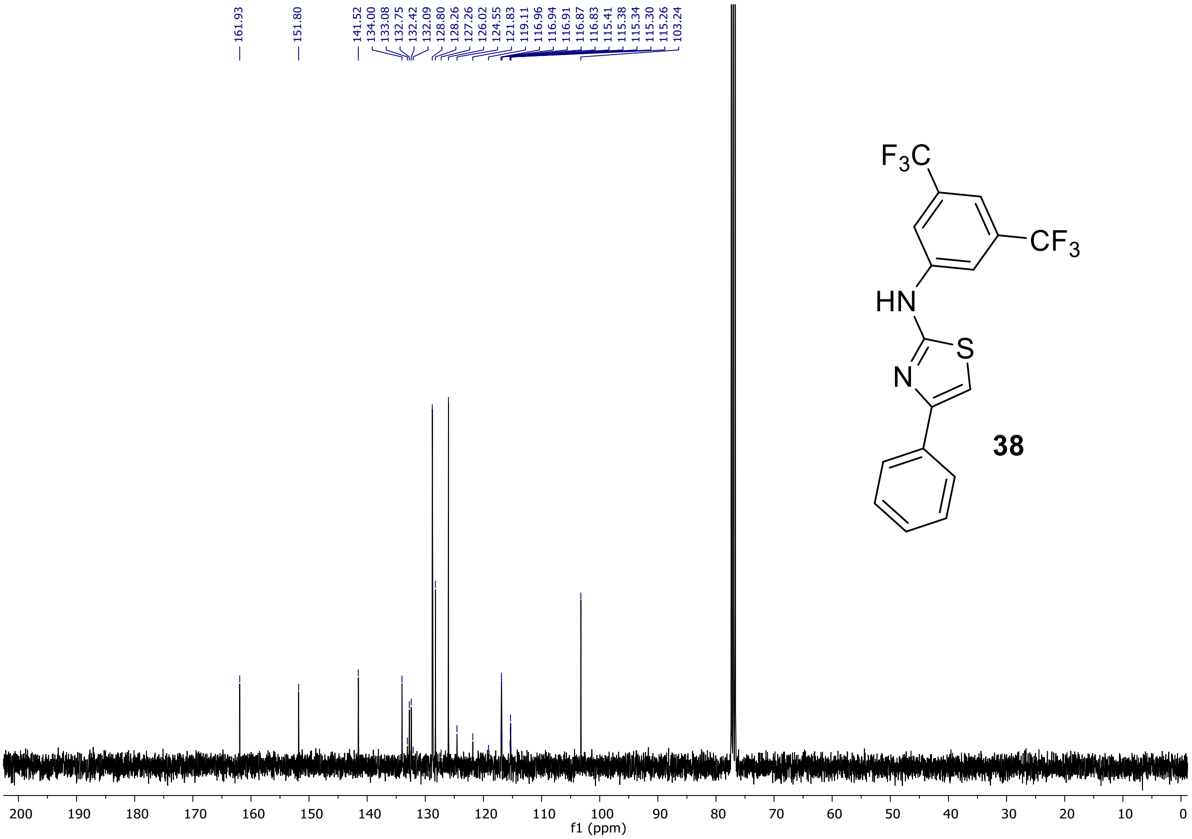

Figure S88. $^{13}C\{^{1}H\}$ Spectrum of **38** in $CDCl_3$ (100 MHz)

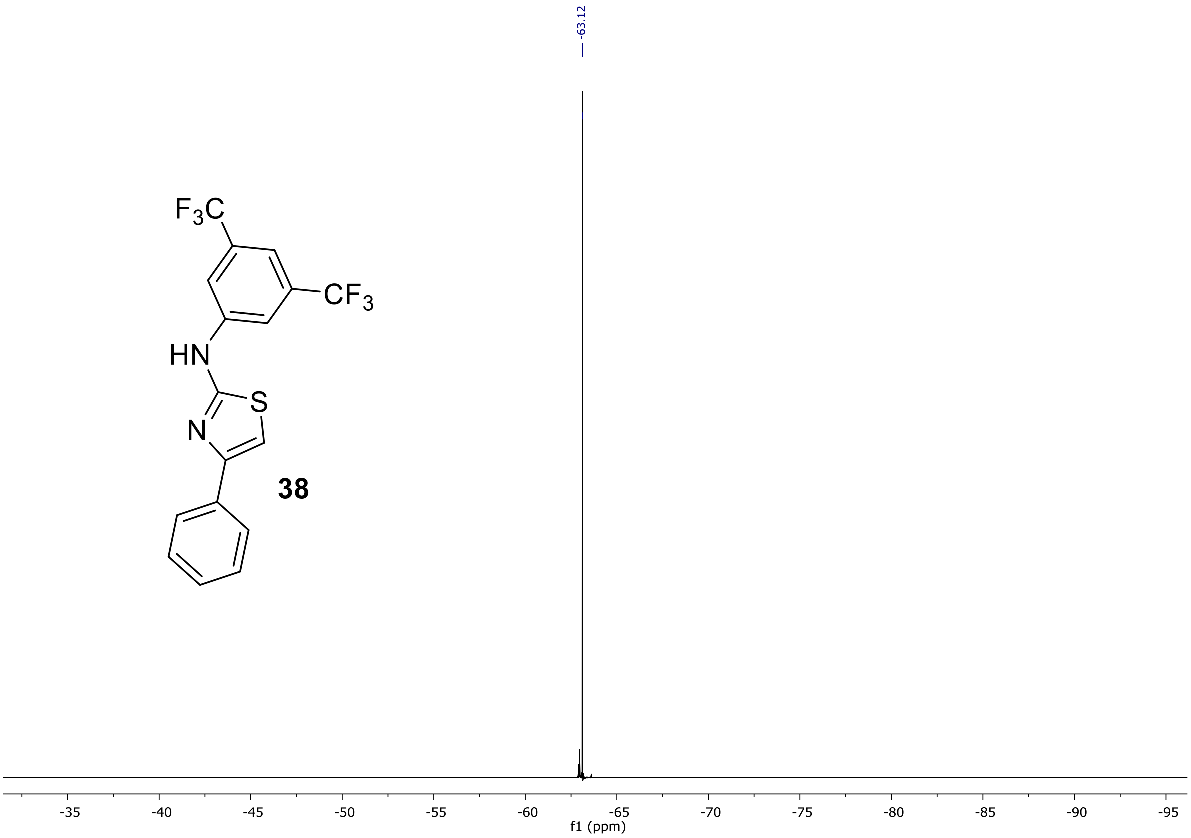

Figure S89. ^{19}F Spectrum of **38** in $CDCl_3$ (376 MHz)

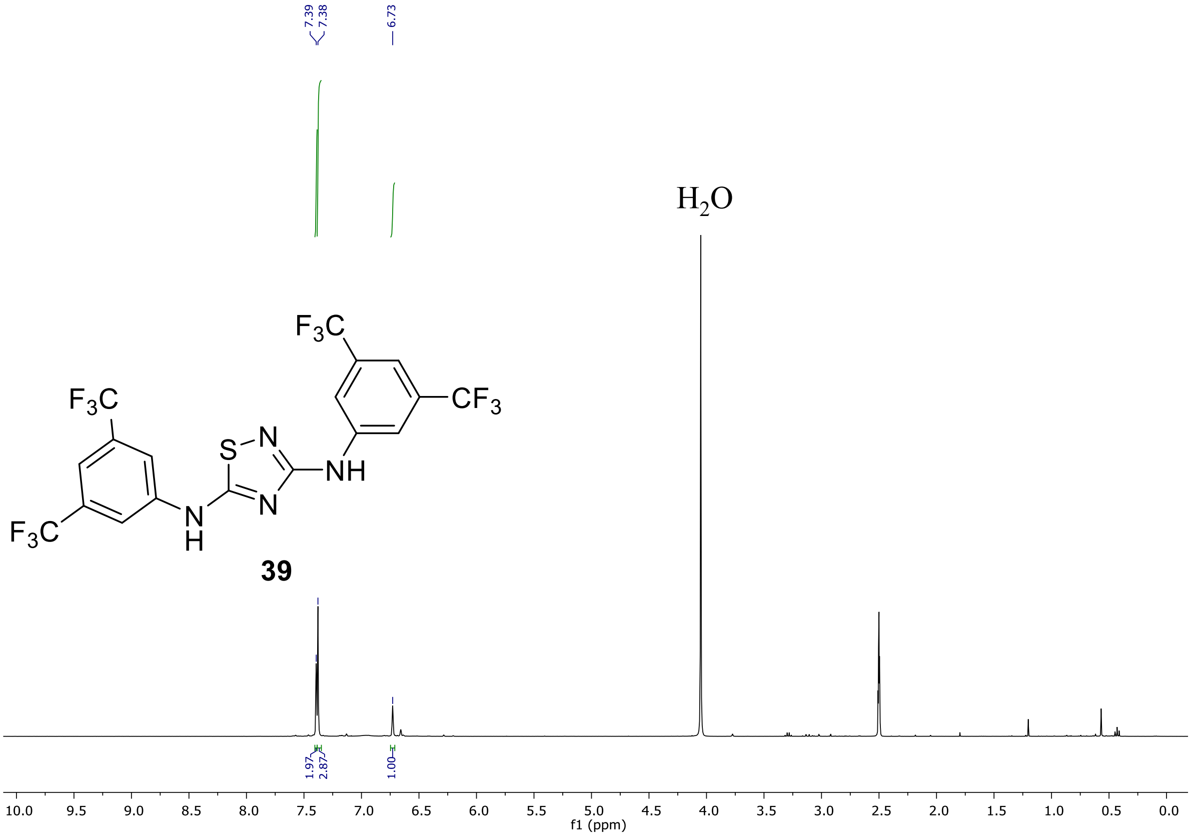

Figure S90. ^{1}H Spectrum of **39** in DMSO-d_6 (400 MHz)

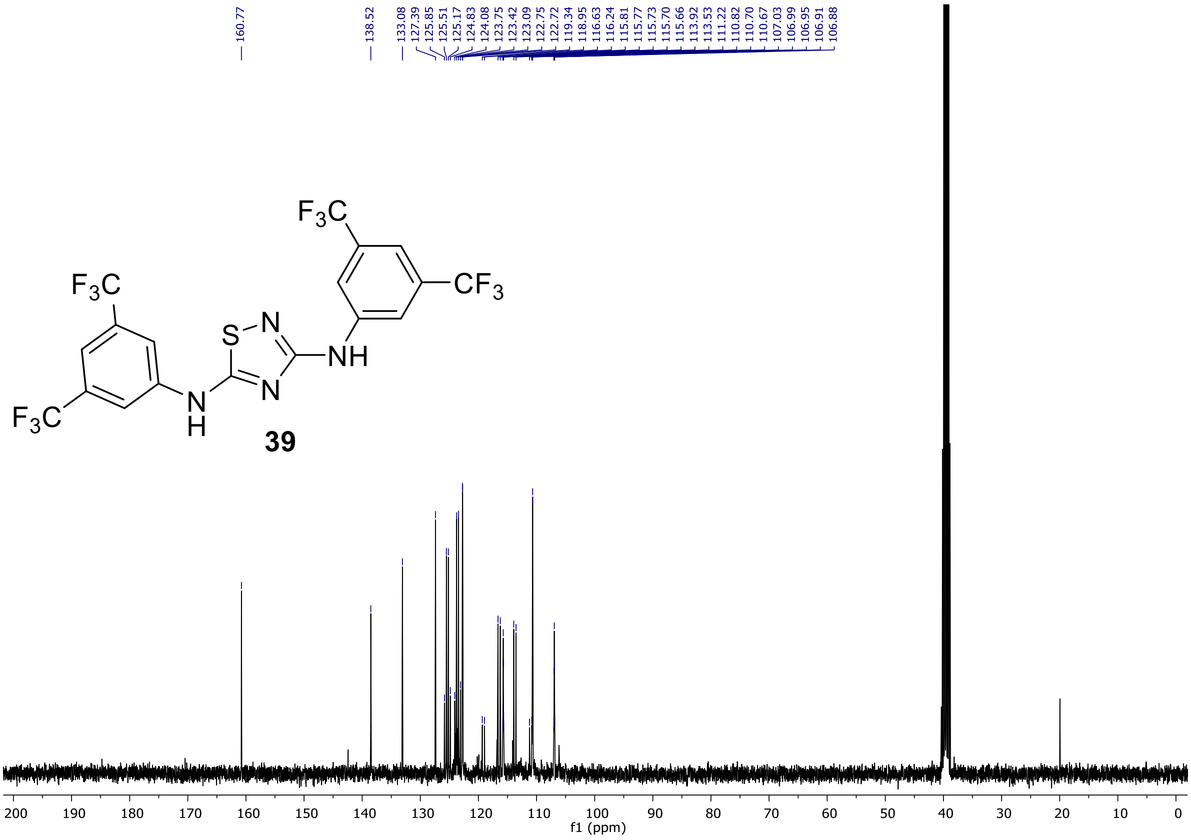

Figure S91. $^{13}C\{^{1}H\}$ Spectrum of **39** in DMSO-d_6 (100 MHz)

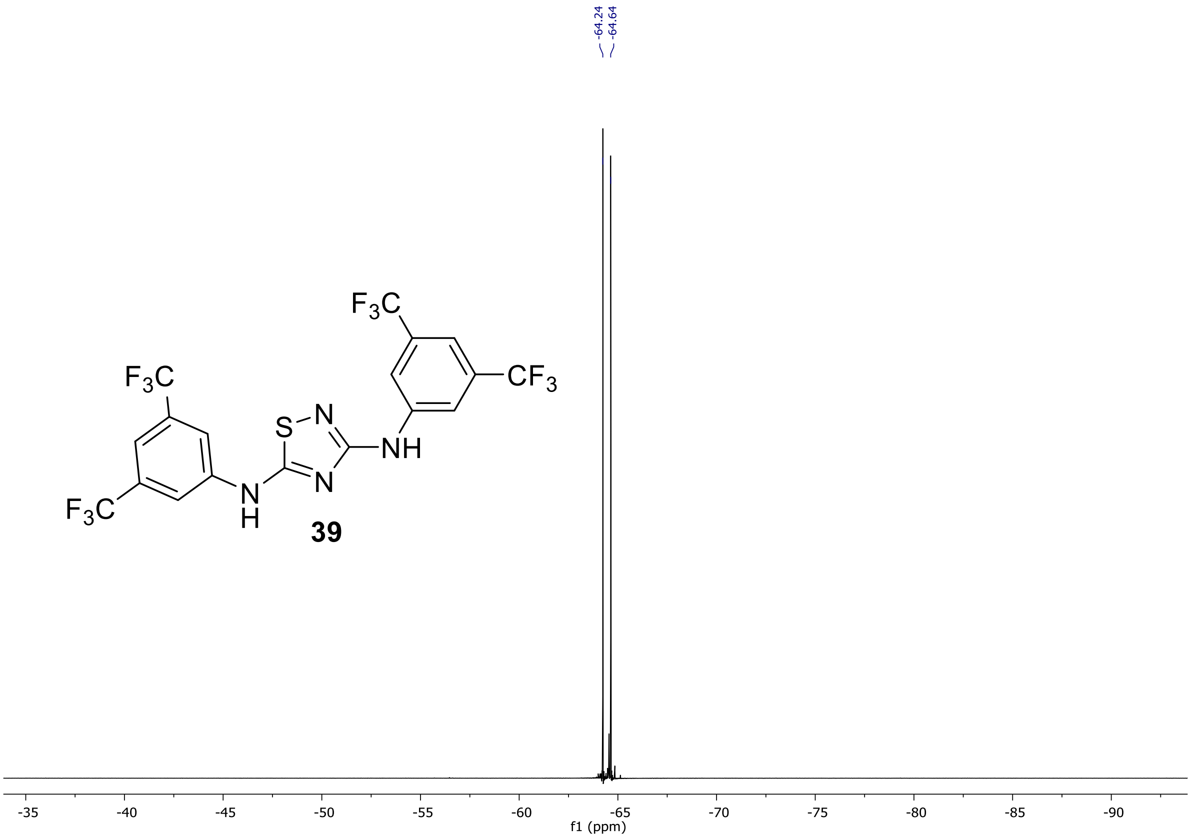

Figure S92. ^{19}F Spectrum of **39** in $CDCl_3$ (376 MHz)

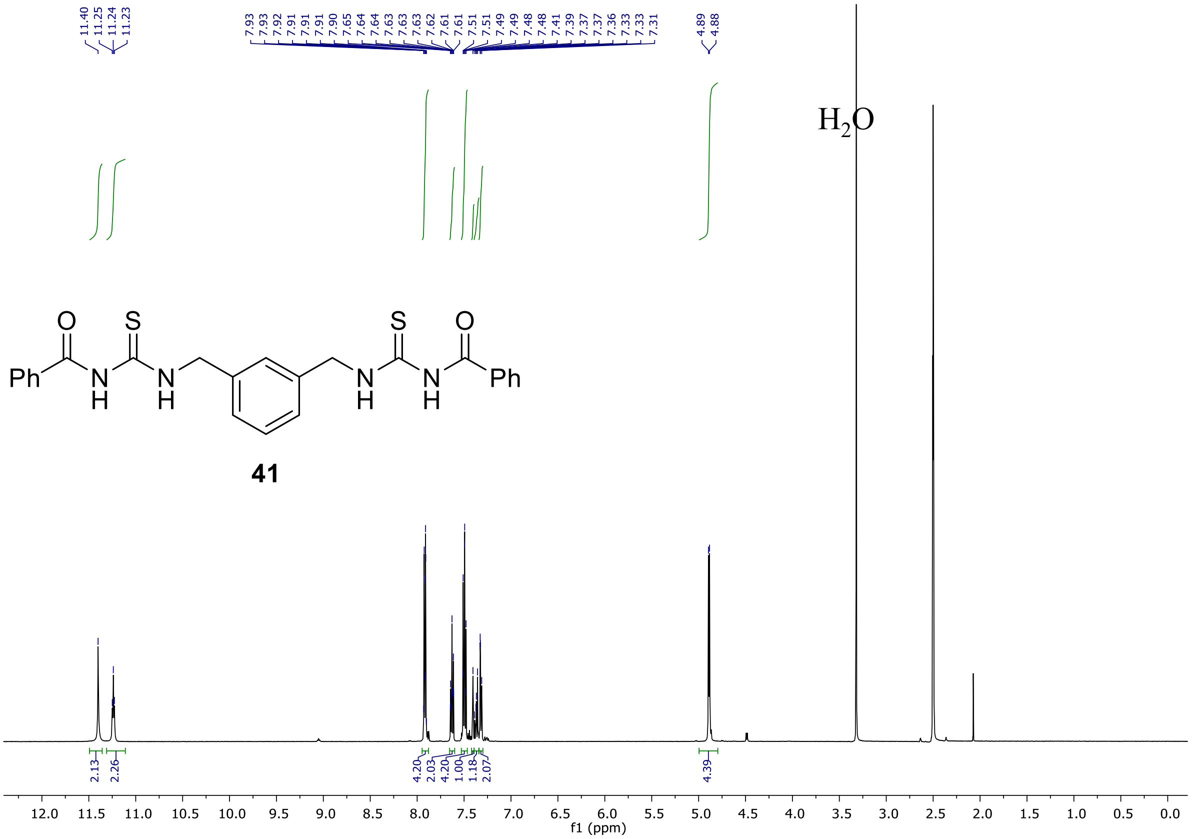

Figure S93. ^{1}H Spectrum of **41** in DMSO-d_6 (500 MHz)

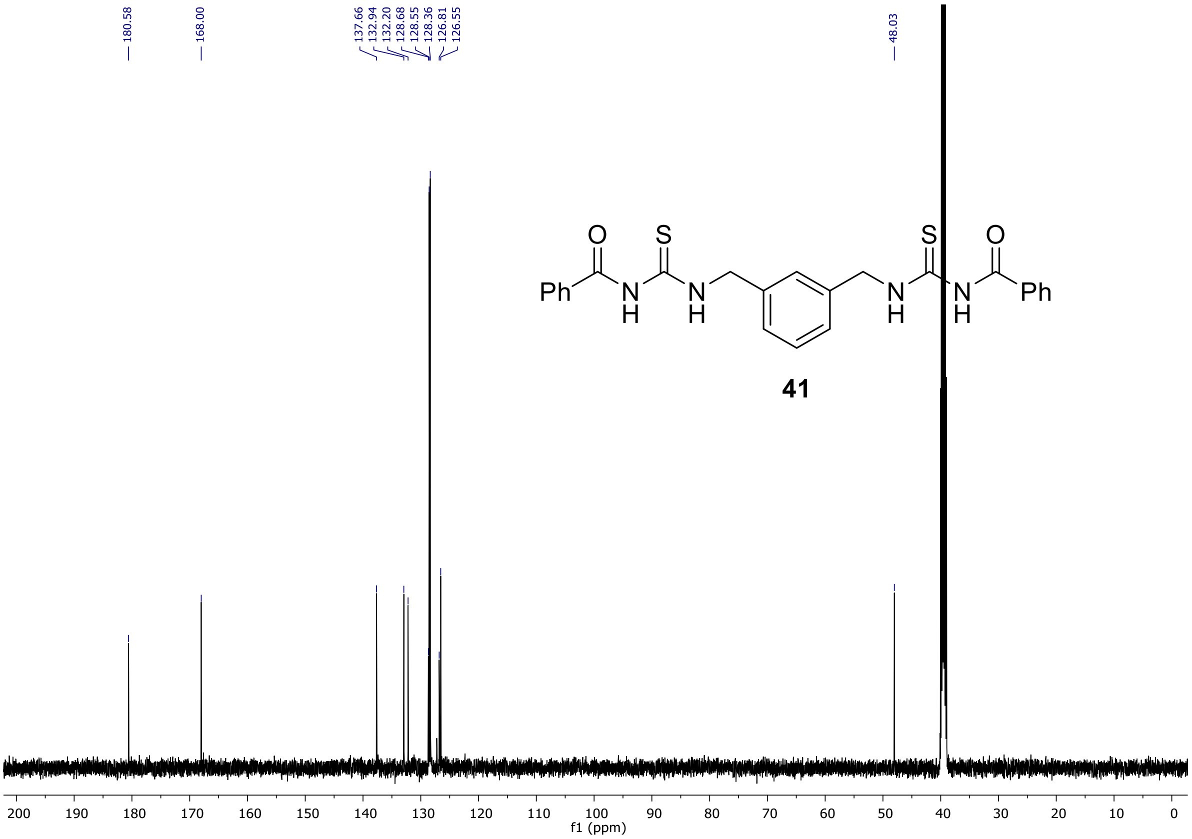

Figure S94. ^{13}C{^{1}H} Spectrum of **41** in DMSO-d_6 (125 MHz)

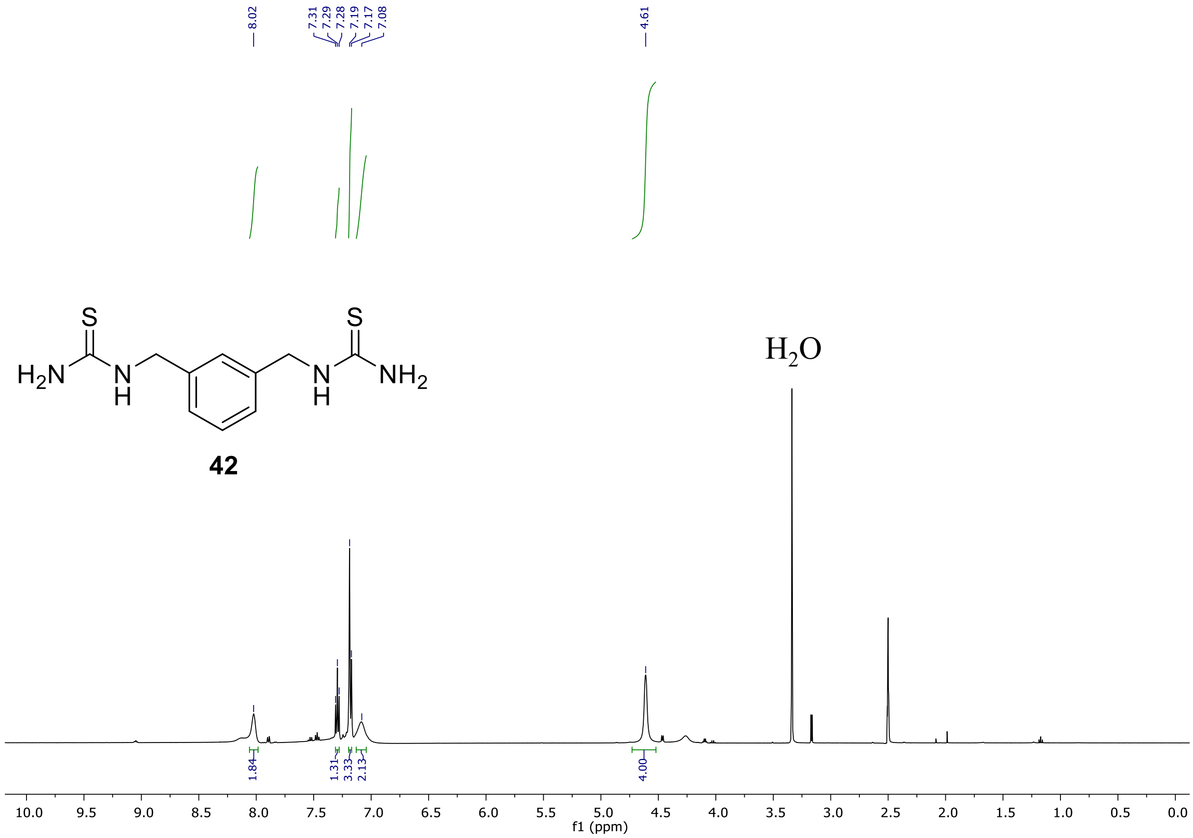

Figure S95. [1]H Spectrum of **42** in DMSO-d_6 (500 MHz)

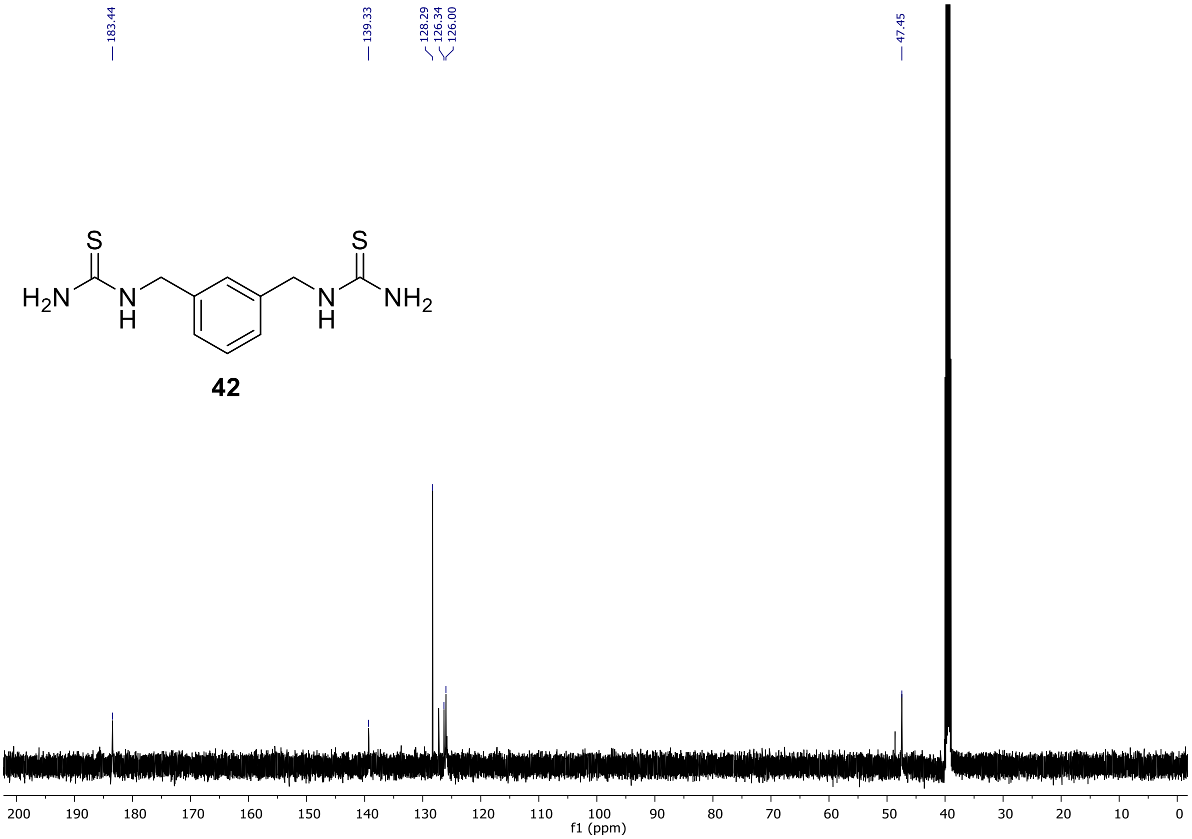

Figure S96. ^{13}C{^{1}H} Spectrum of **42** in DMSO-d_6 (125 MHz)

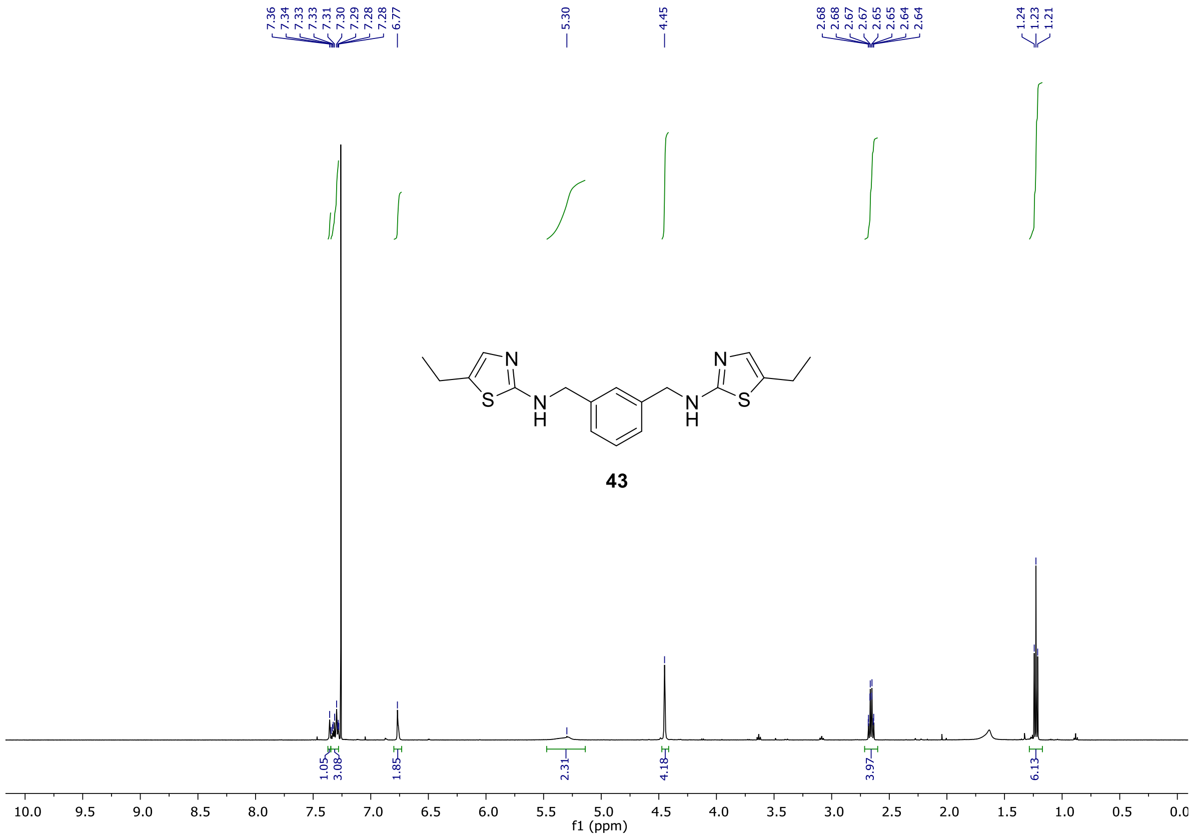

Figure S97. ^{1}H Spectrum of **43** in $CDCl_3$ (500 MHz)

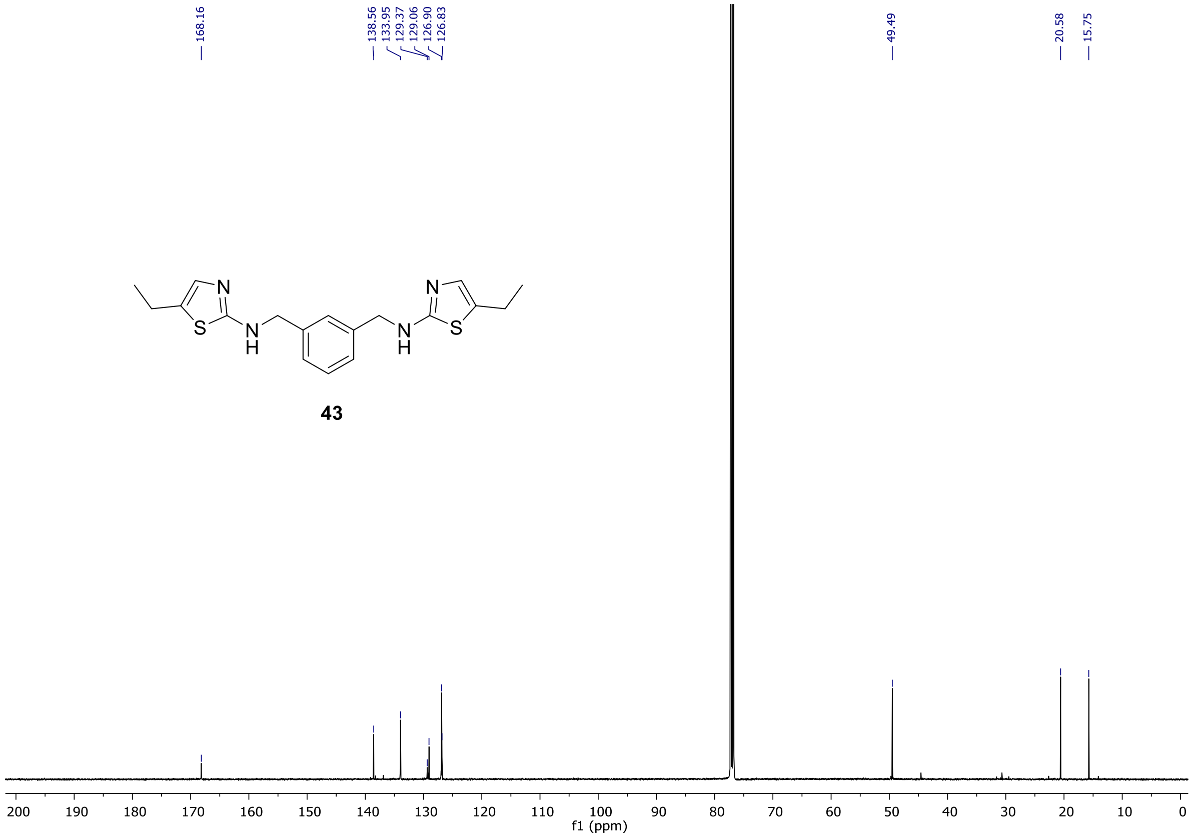

Figure S98. $^{13}C\{^{1}H\}$ Spectrum of **43** in $CDCl_3$ (125 MHz)

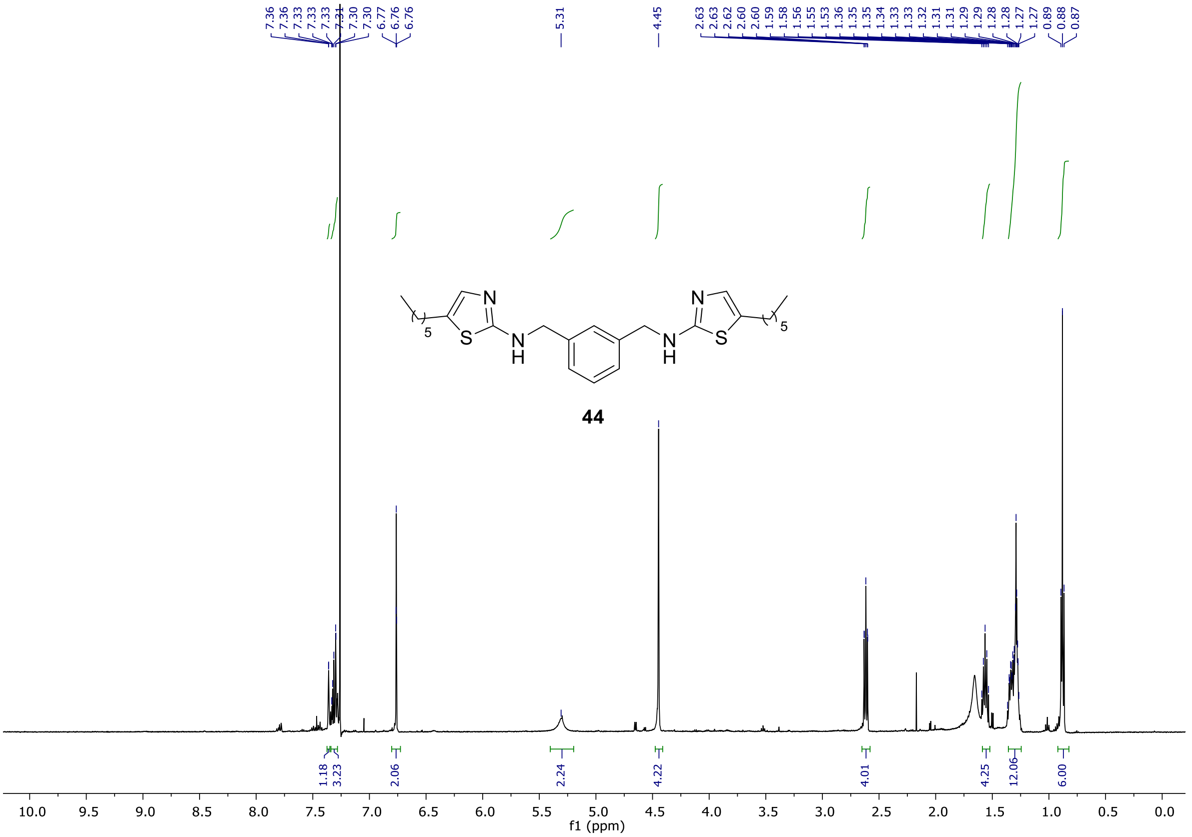

Figure S99. ^{1}H Spectrum of **44** in $CDCl_3$ (500 MHz)

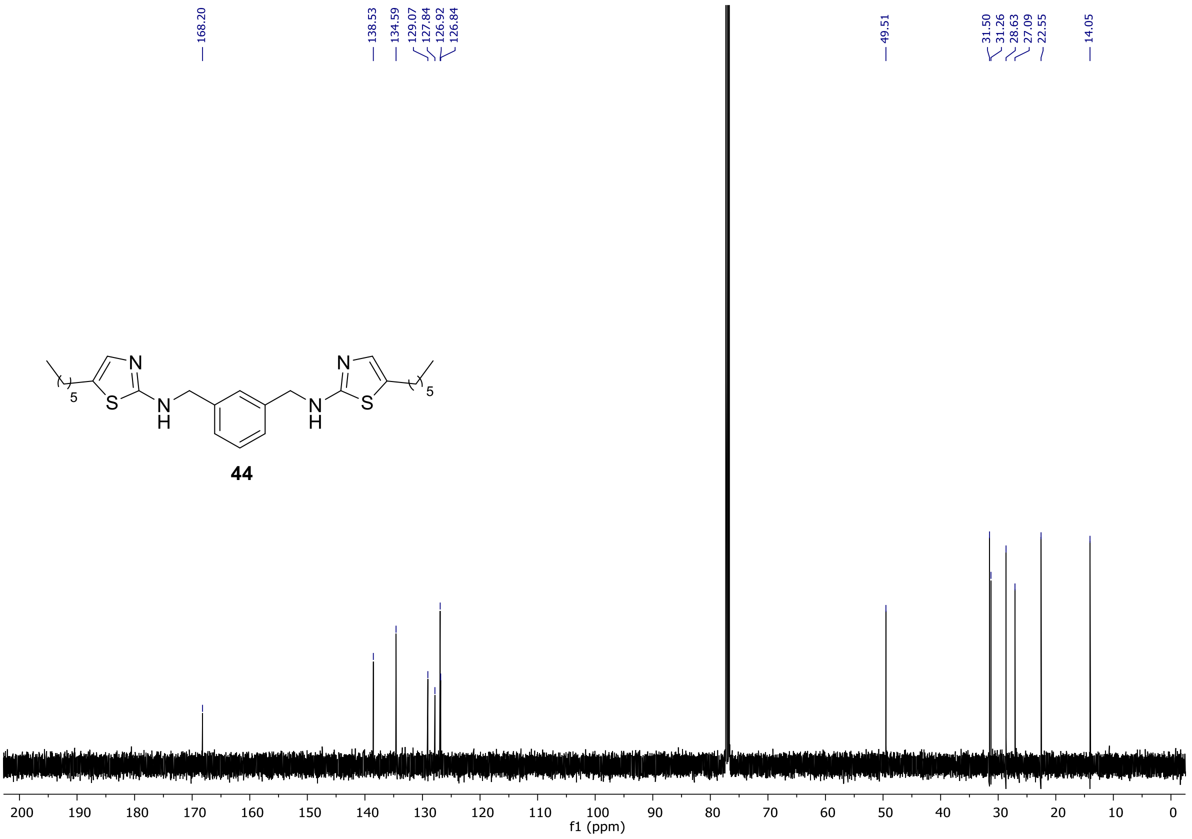

Figure S100. $^{13}C\{^{1}H\}$ Spectrum of **44** in $CDCl_3$ (125 MHz)

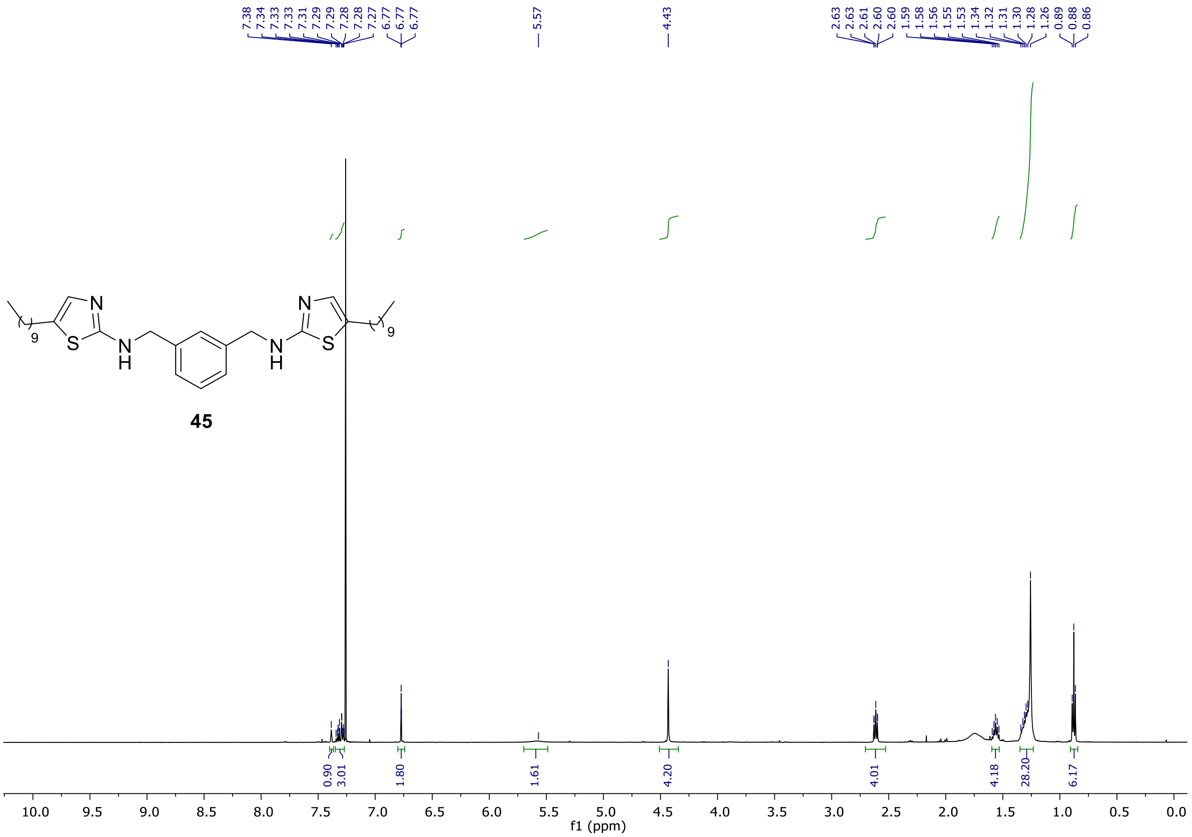

Figure S101. ^{1}H Spectrum of **45** in $CDCl_3$ (500 MHz)

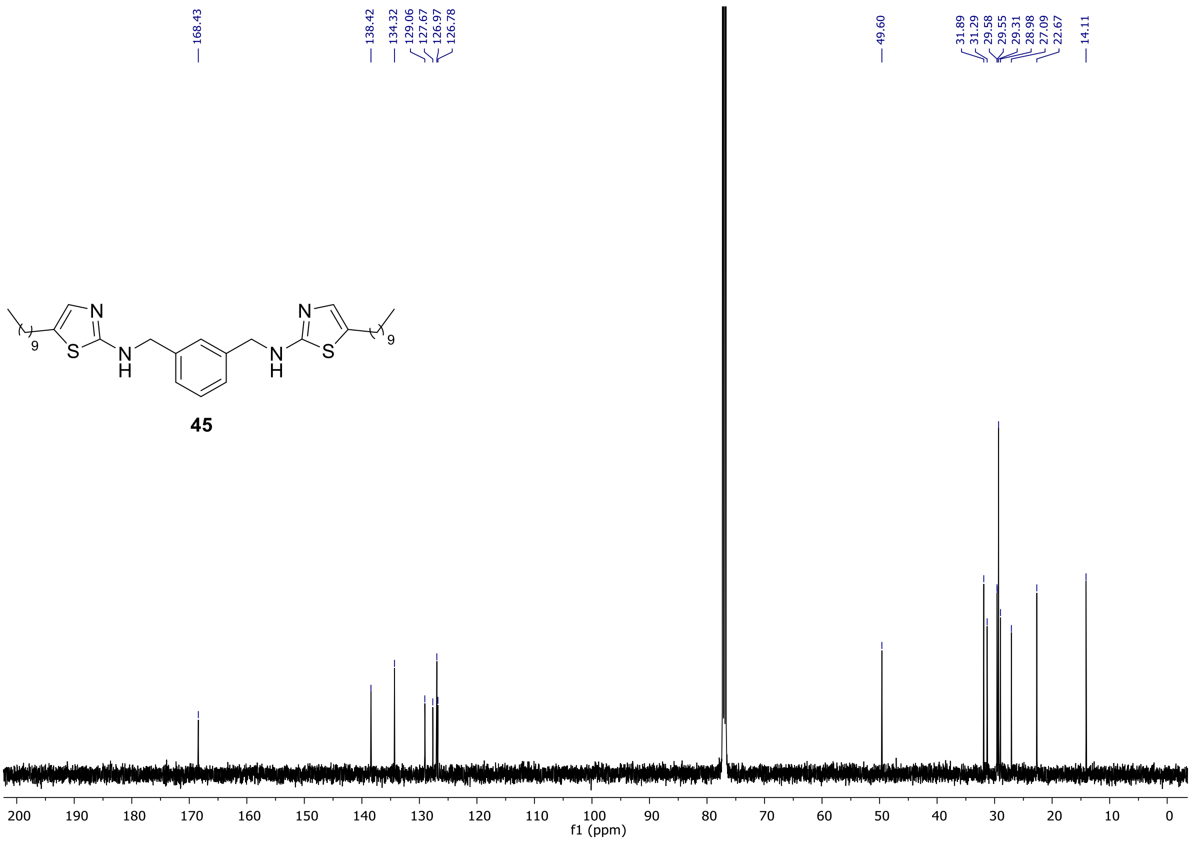

Figure S102. $^{13}C\{^{1}H\}$ Spectrum of **45** in $CDCl_3$ (125 MHz)

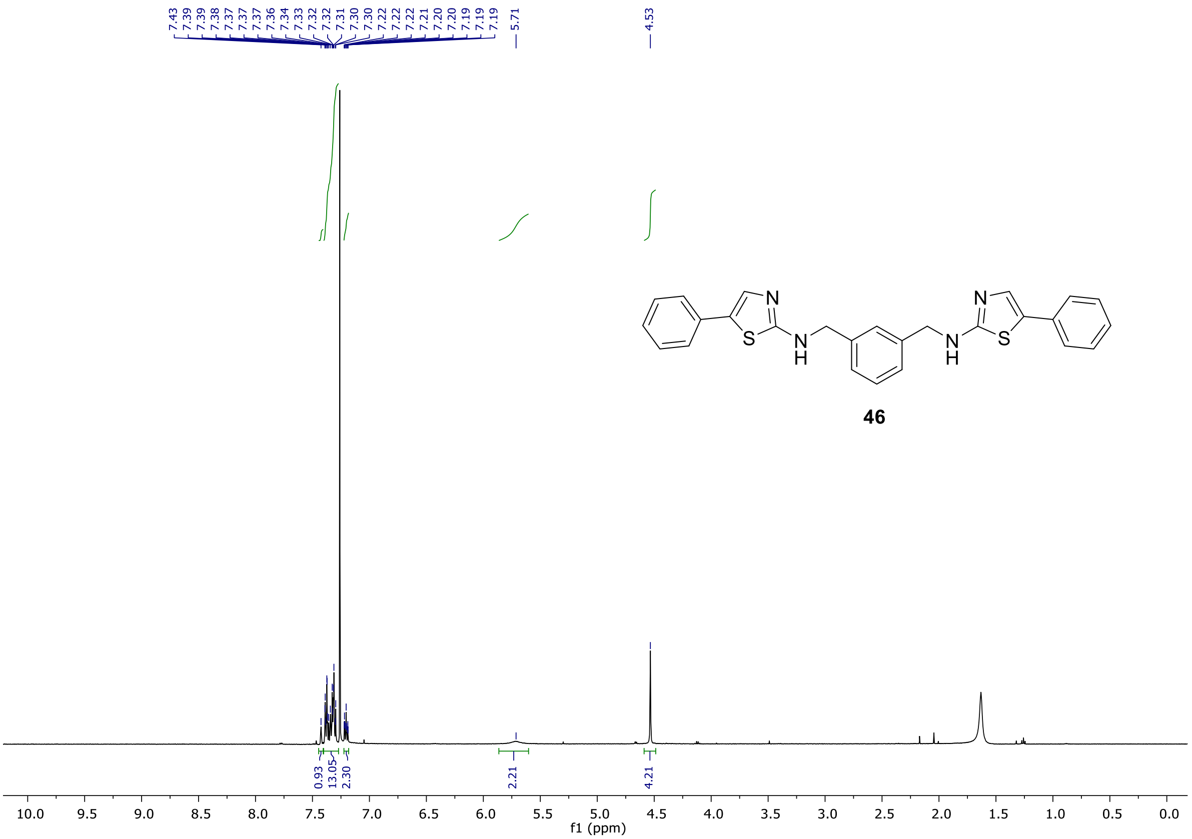

Figure S103. ^{1}H Spectrum of **46** in $CDCl_3$ (500 MHz)

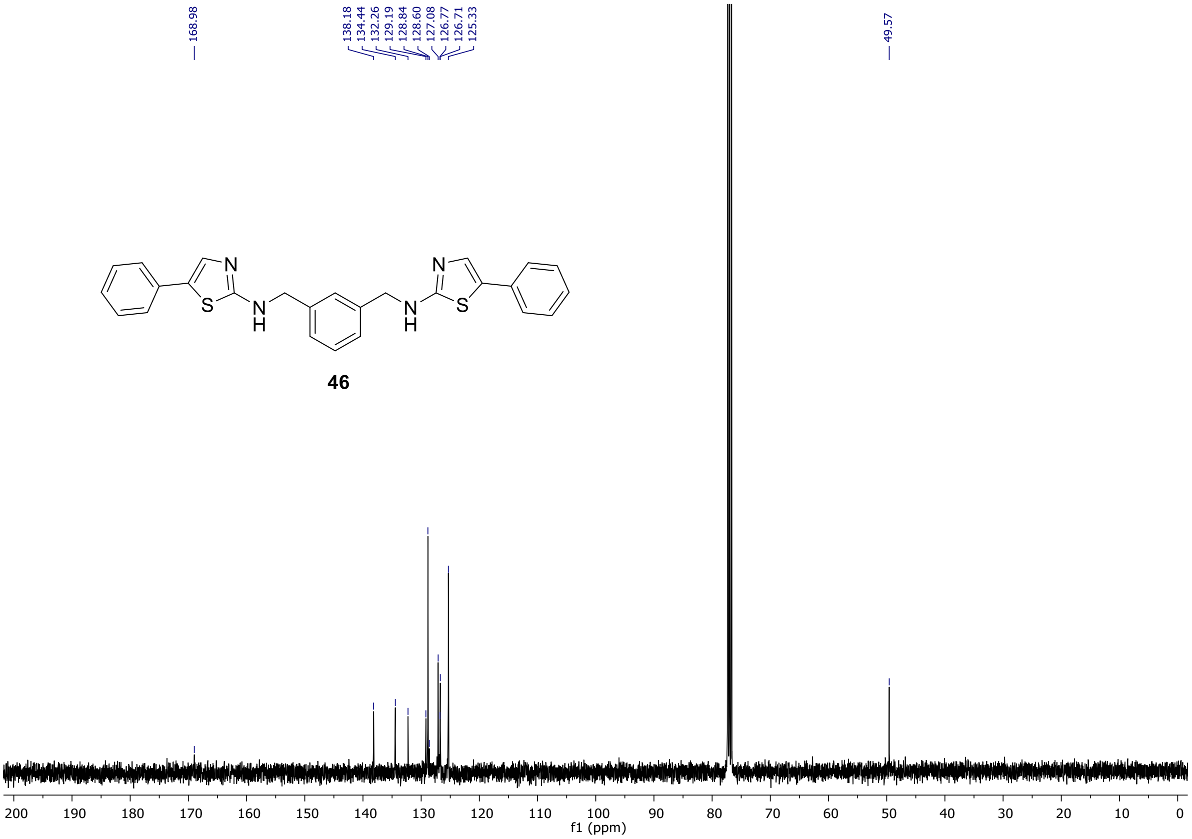

Figure S104. $^{13}C\{^{1}H\}$ Spectrum of **46** in $CDCl_3$ (100 MHz)

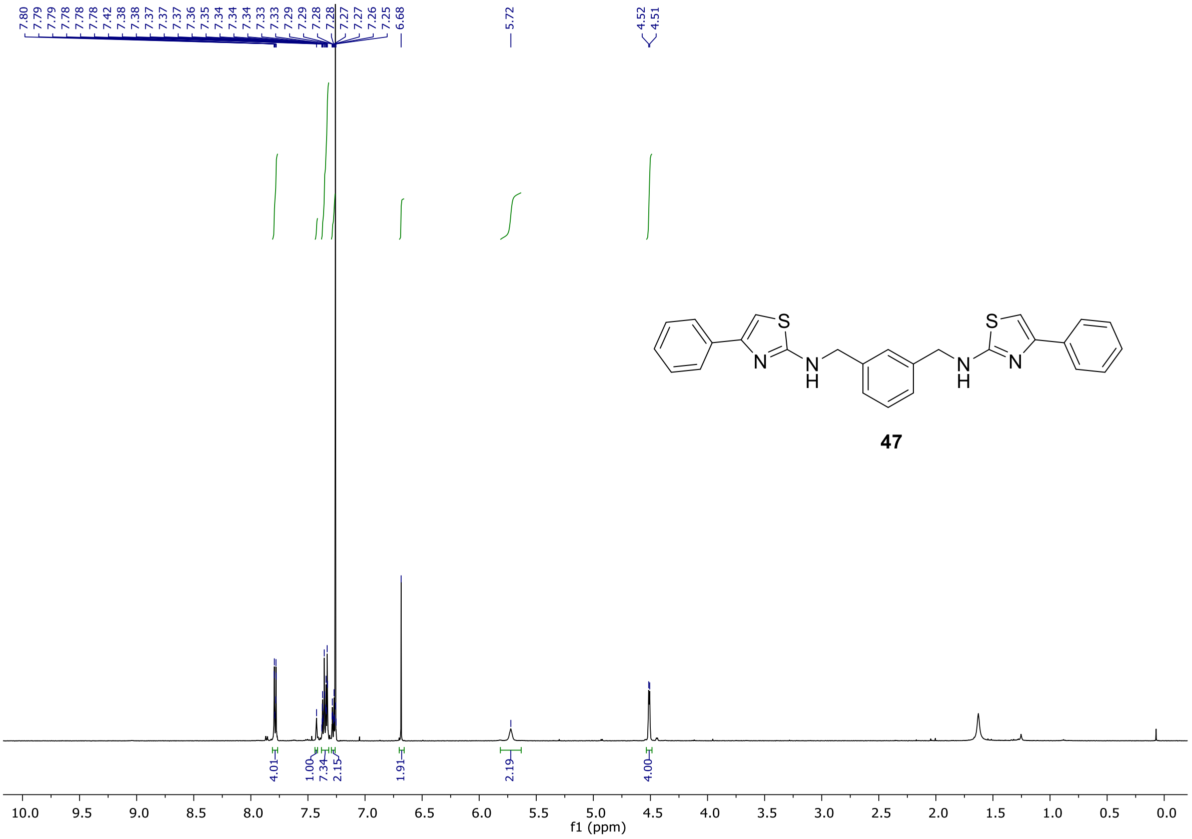

Figure S105. ^{1}H Spectrum of **47** in $CDCl_3$ (500 MHz)

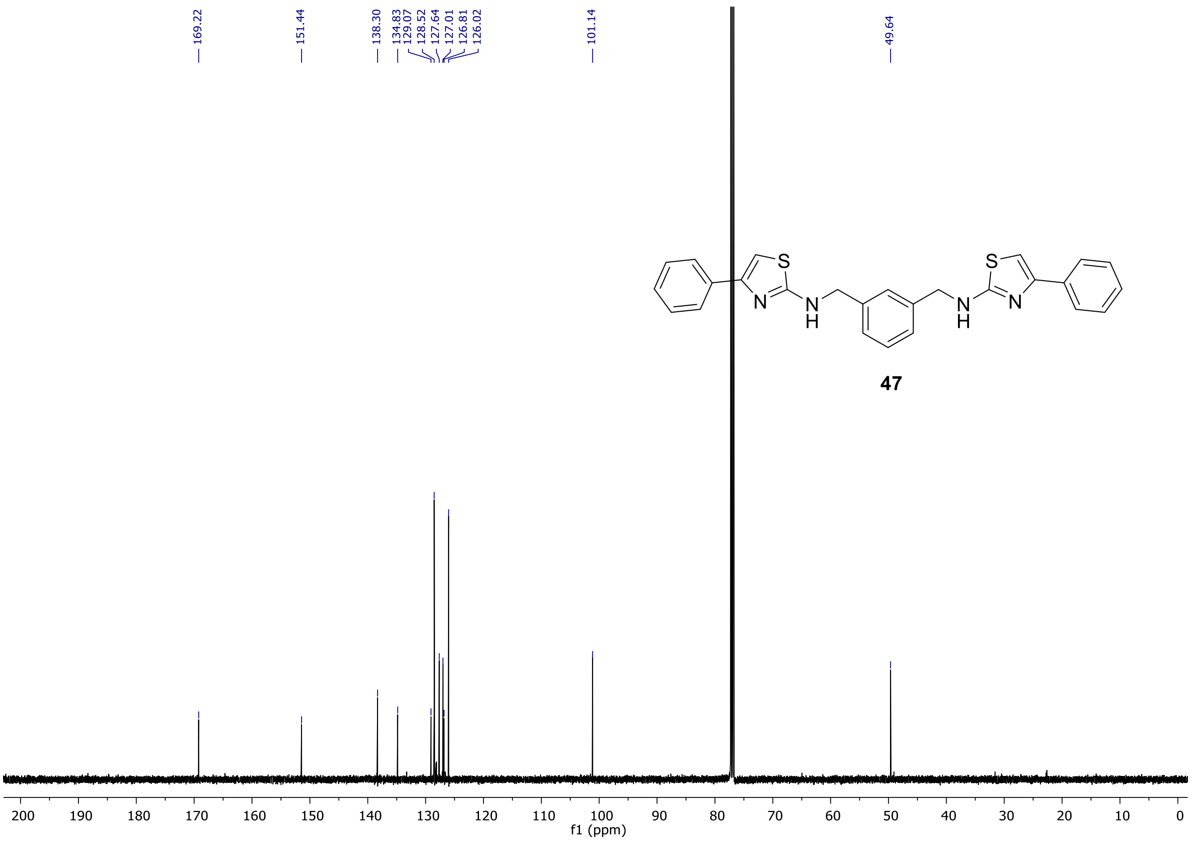

Figure S106. $^{13}C\{^{1}H\}$ Spectrum of **47** in $CDCl_3$ (125 MHz)

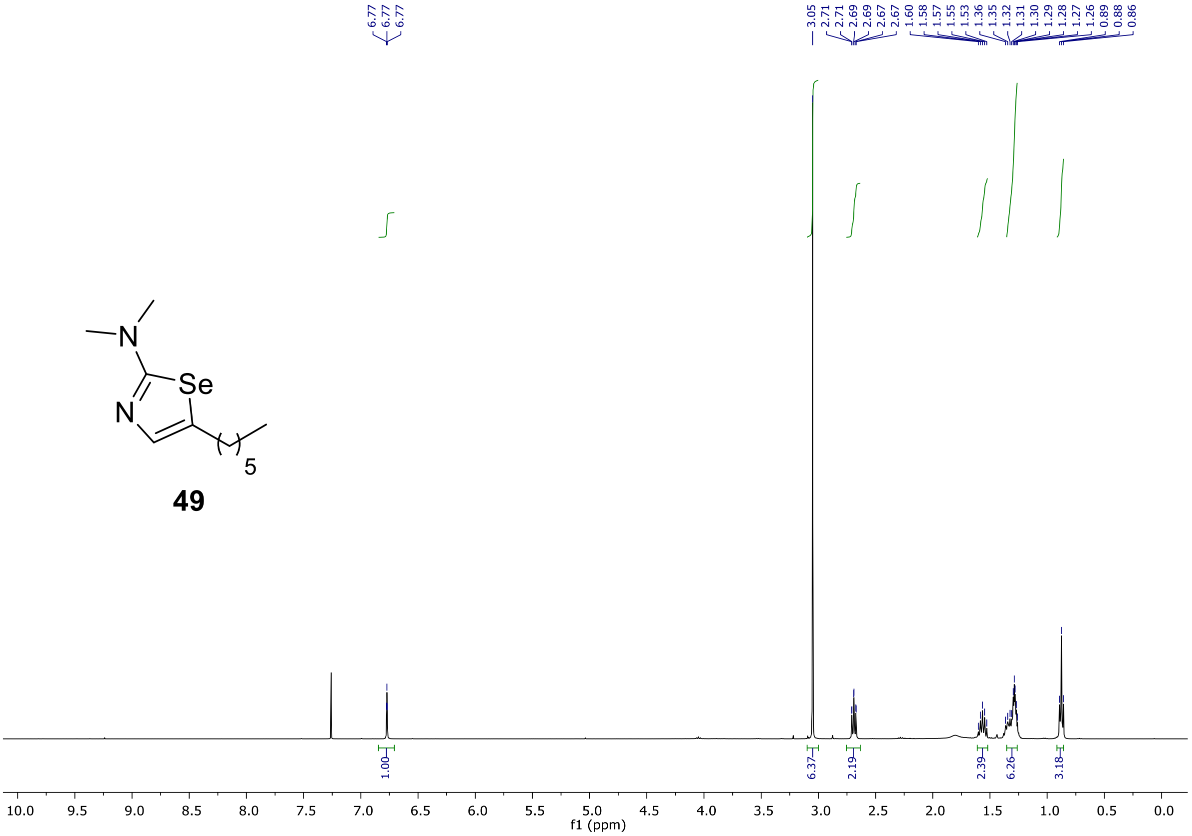

Figure S107. [1]H Spectrum of **49** in $CDCl_3$ (400 MHz)

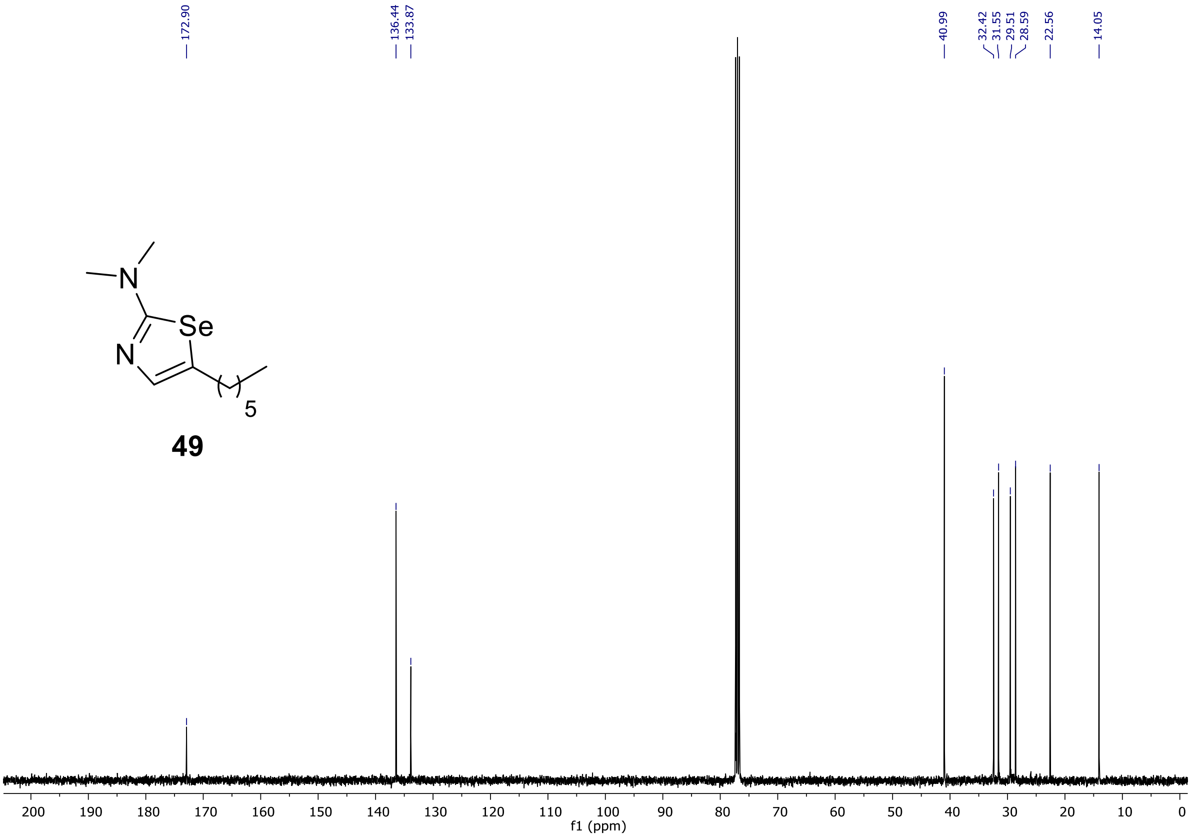

Figure S108. ^{13}C{^{1}H} Spectrum of **49** in $CDCl_3$ (100 MHz)

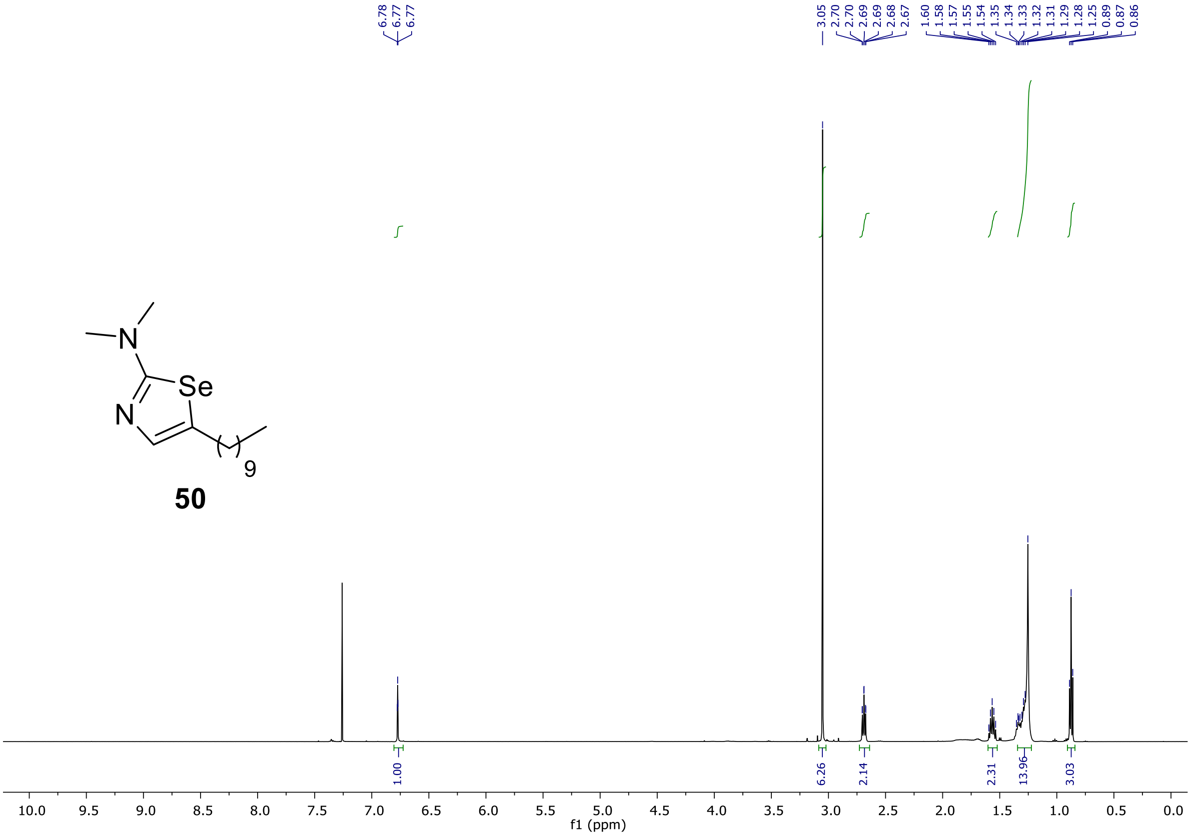

Figure S109. ^{1}H Spectrum of **50** in $CDCl_3$ (500 MHz)

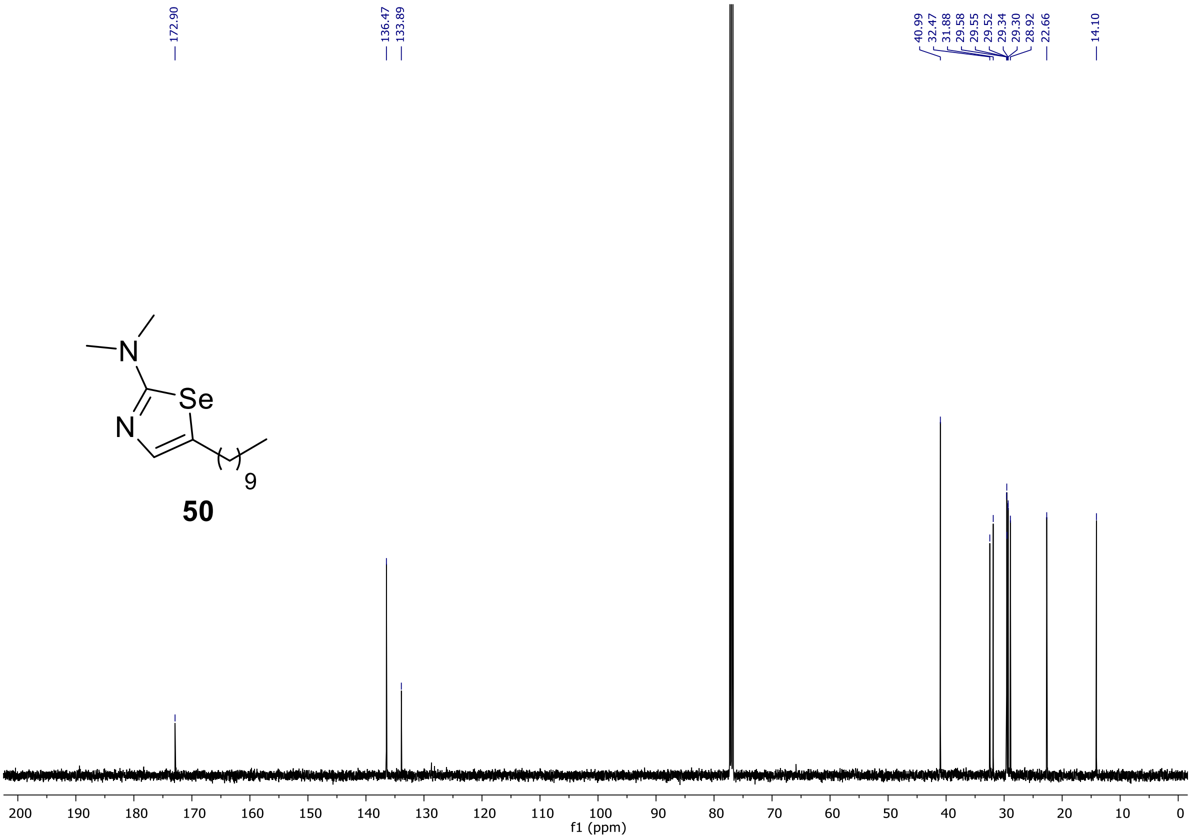

Figure S110. $^{13}C\{^{1}H\}$ Spectrum of **50** in $CDCl_3$ (125 MHz)

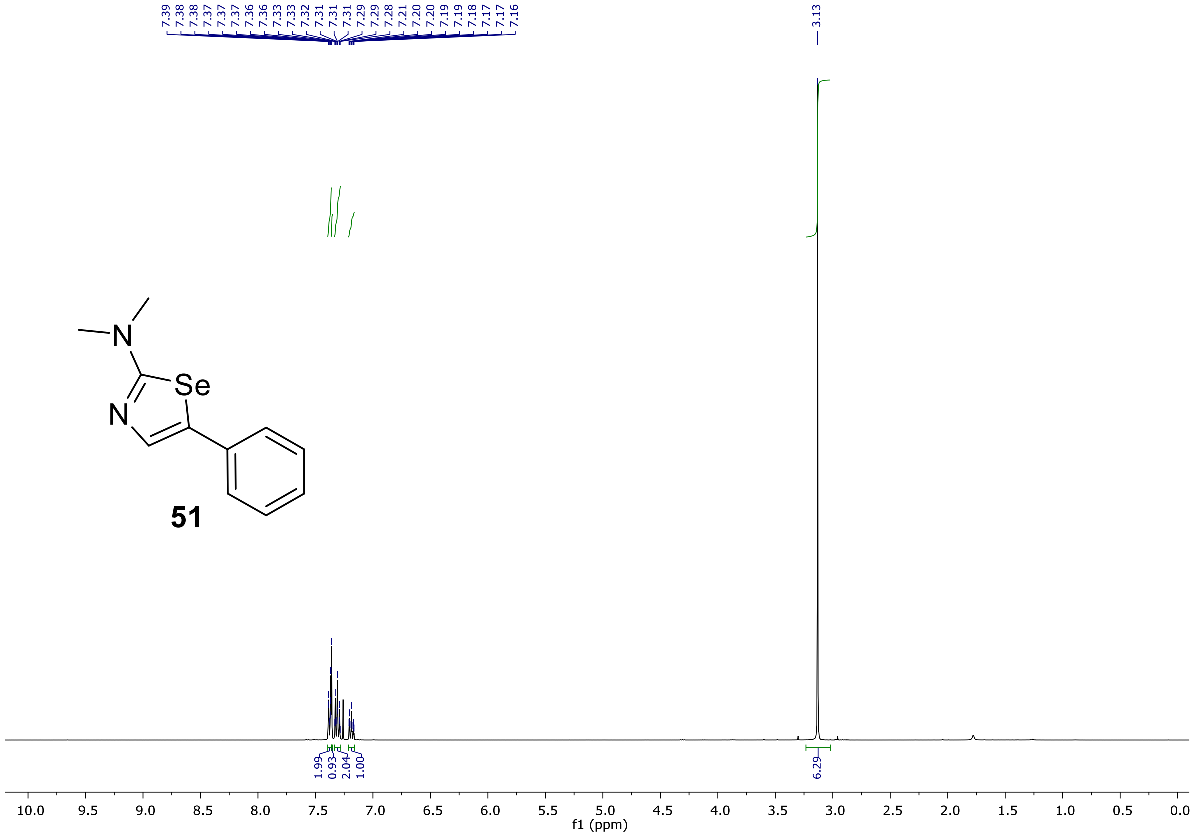

Figure S111. ^{1}H Spectrum of **51** in $CDCl_3$ (400 MHz)

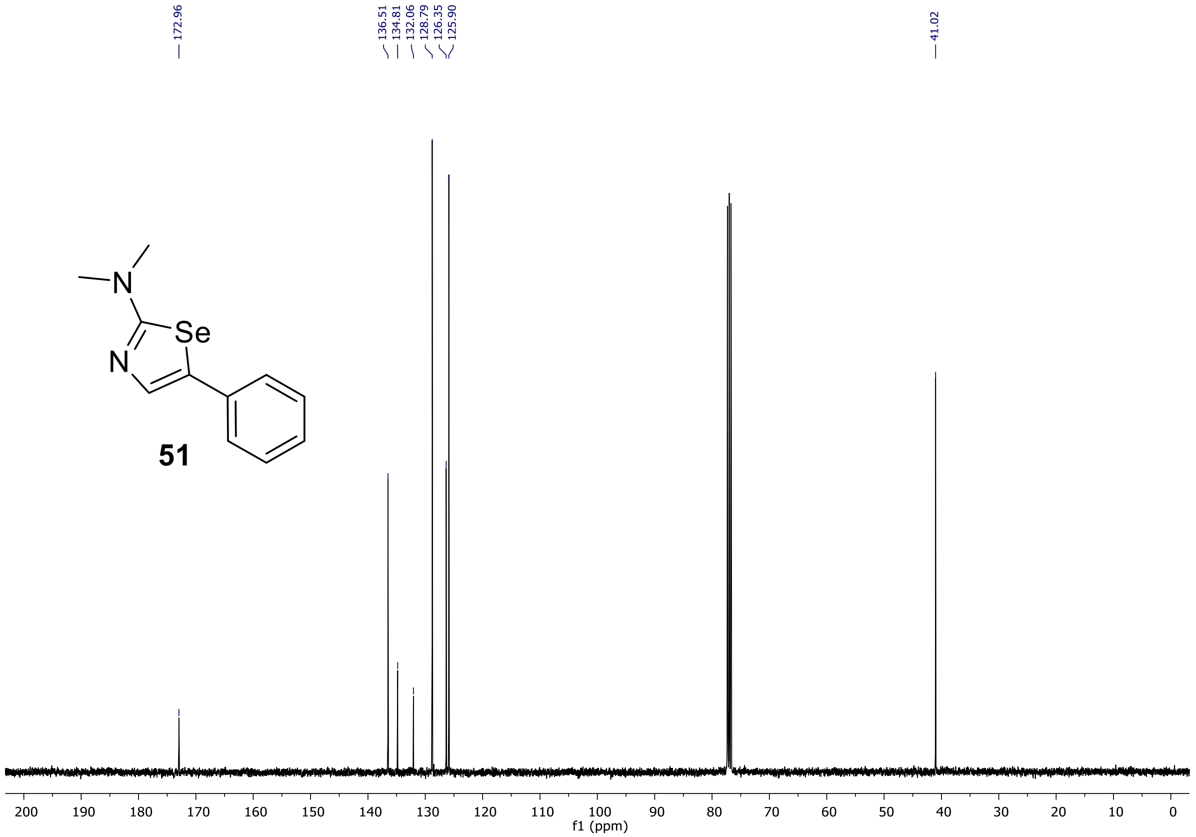

Figure S112. $^{13}C\{^{1}H\}$ Spectrum of **51** in $CDCl_3$ (100 MHz)

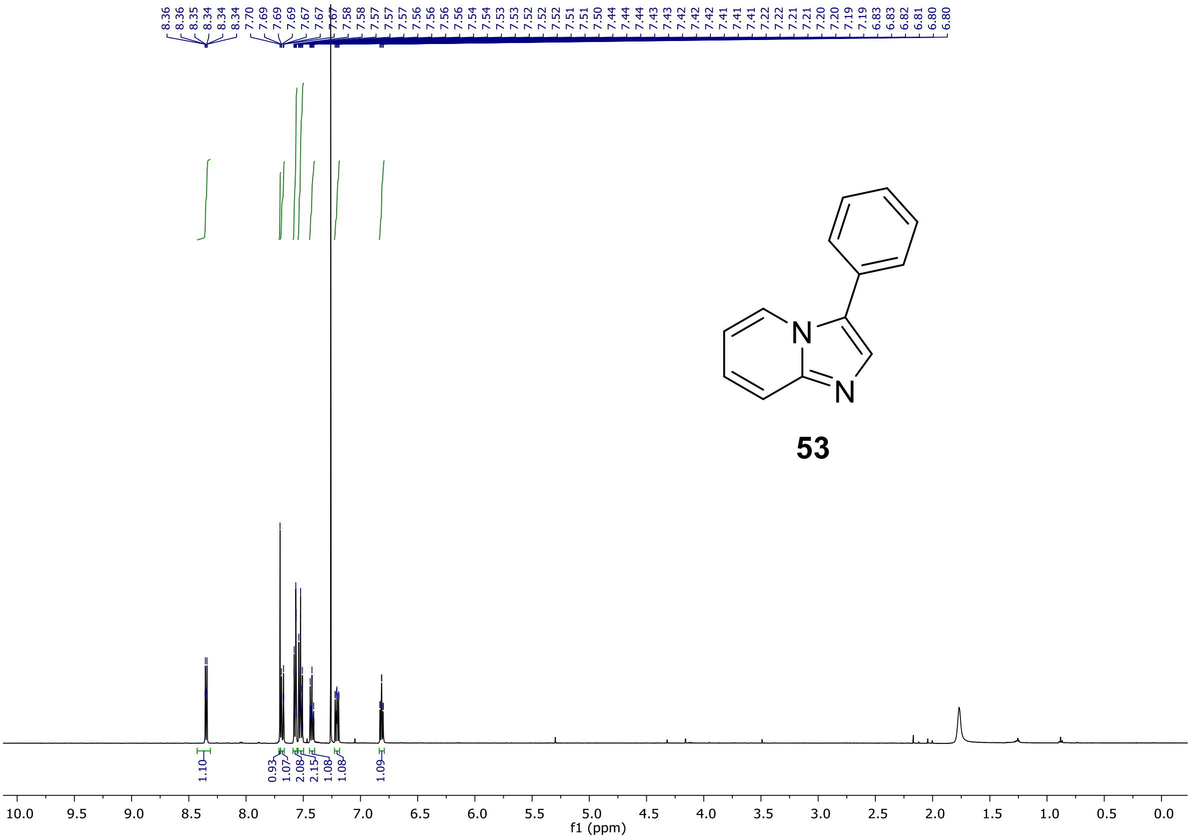

Figure S113. ^{1}H Spectrum of **53** in $CDCl_3$ (500 MHz)

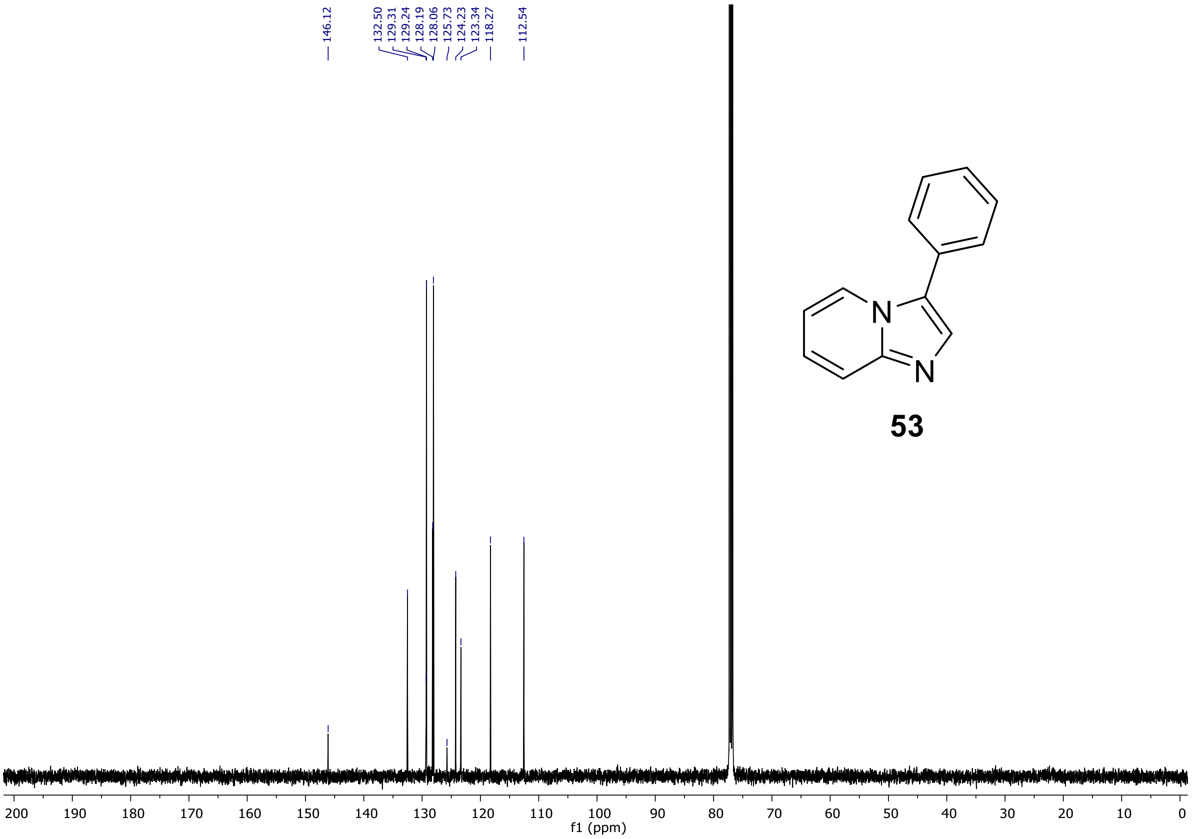

Figure S114. $^{13}C\{^{1}H\}$ Spectrum of **53** in $CDCl_3$ (125 MHz)

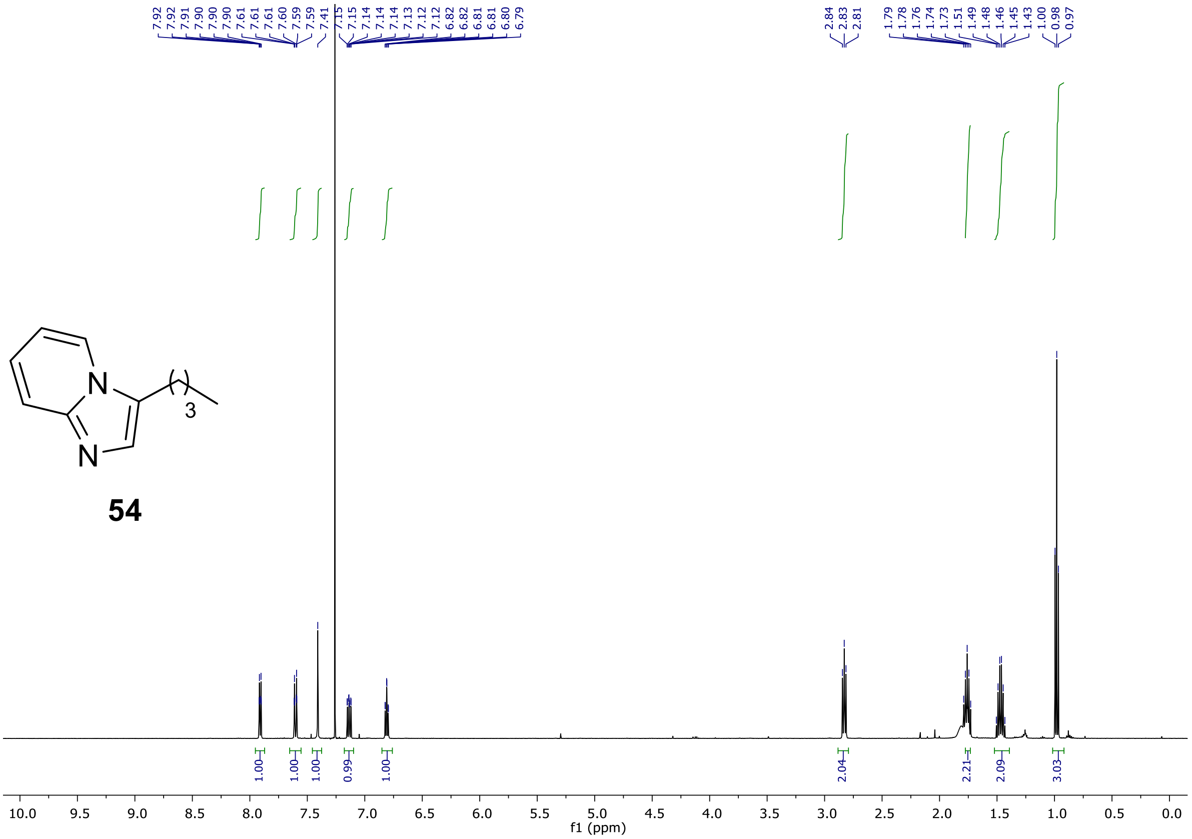

Figure S115. ^{1}H Spectrum of **54** in $CDCl_3$ (500 MHz)

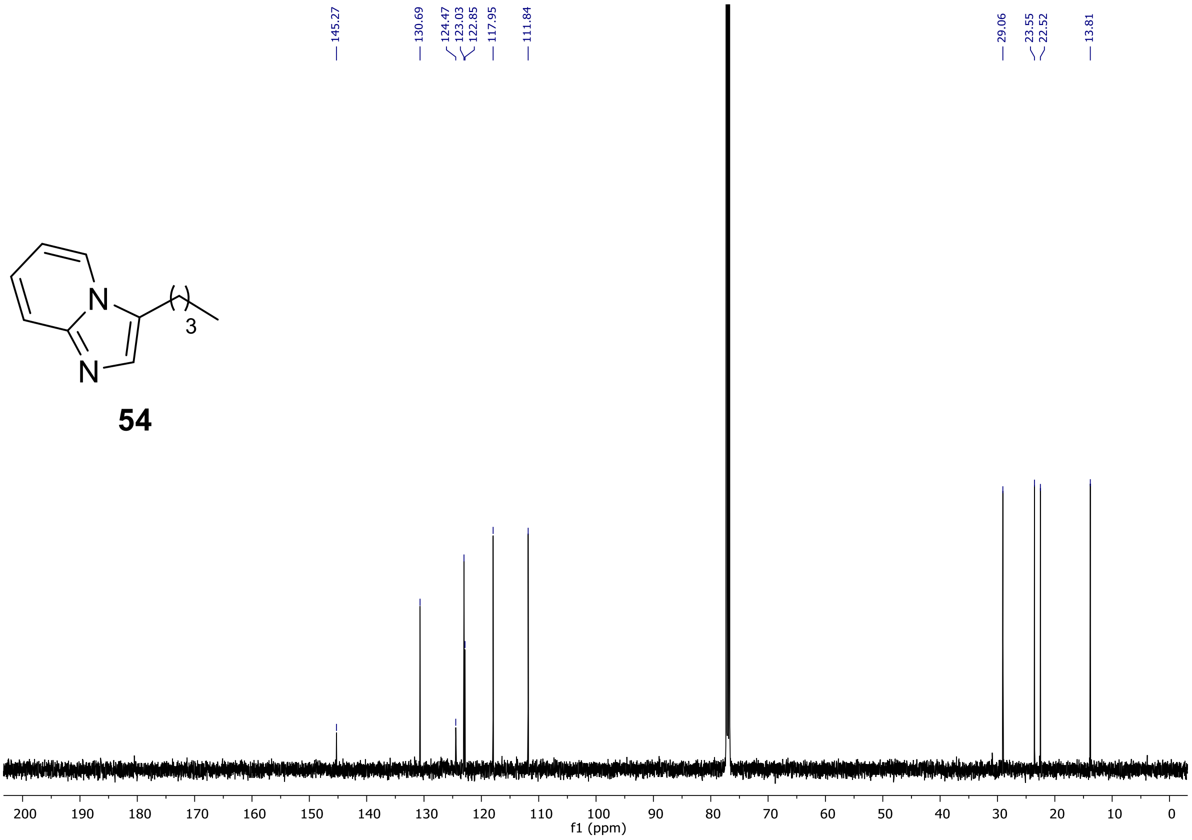

Figure S116. $^{13}C\{^{1}H\}$ Spectrum of **54** in $CDCl_3$ (125 MHz)

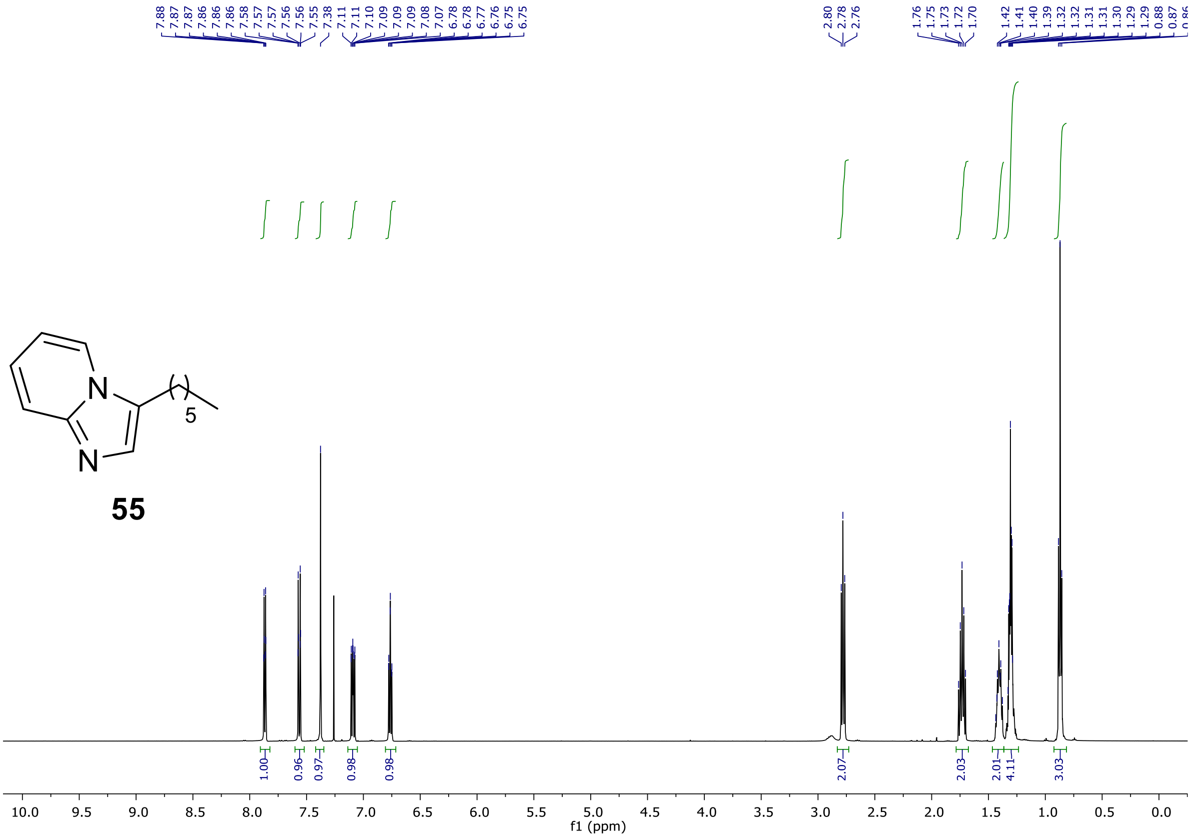

Figure S117. ^{1}H Spectrum of **55** in $CDCl_3$ (500 MHz)

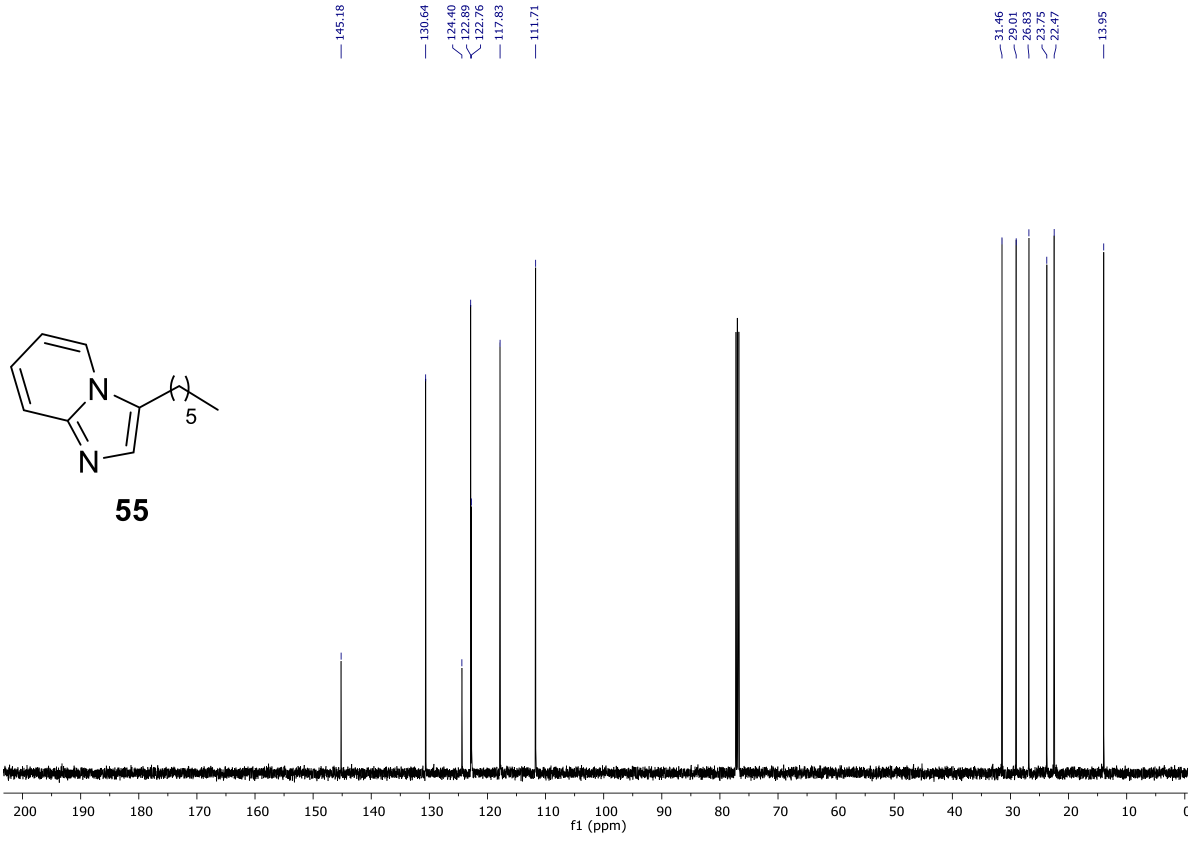

Figure S118. $^{13}C\{^{1}H\}$ Spectrum of **55** in $CDCl_3$ (125 MHz)

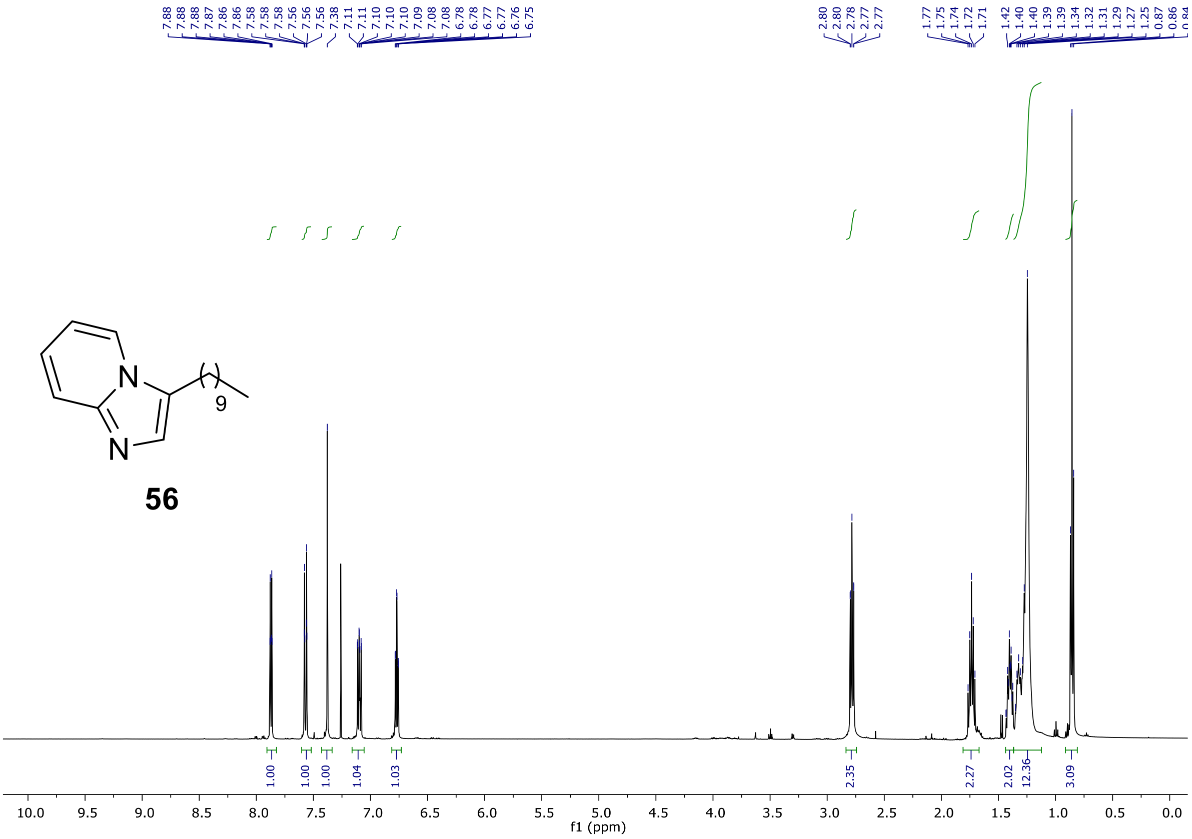

Figure S119. ^{1}H Spectrum of **56** in $CDCl_3$ (500 MHz)

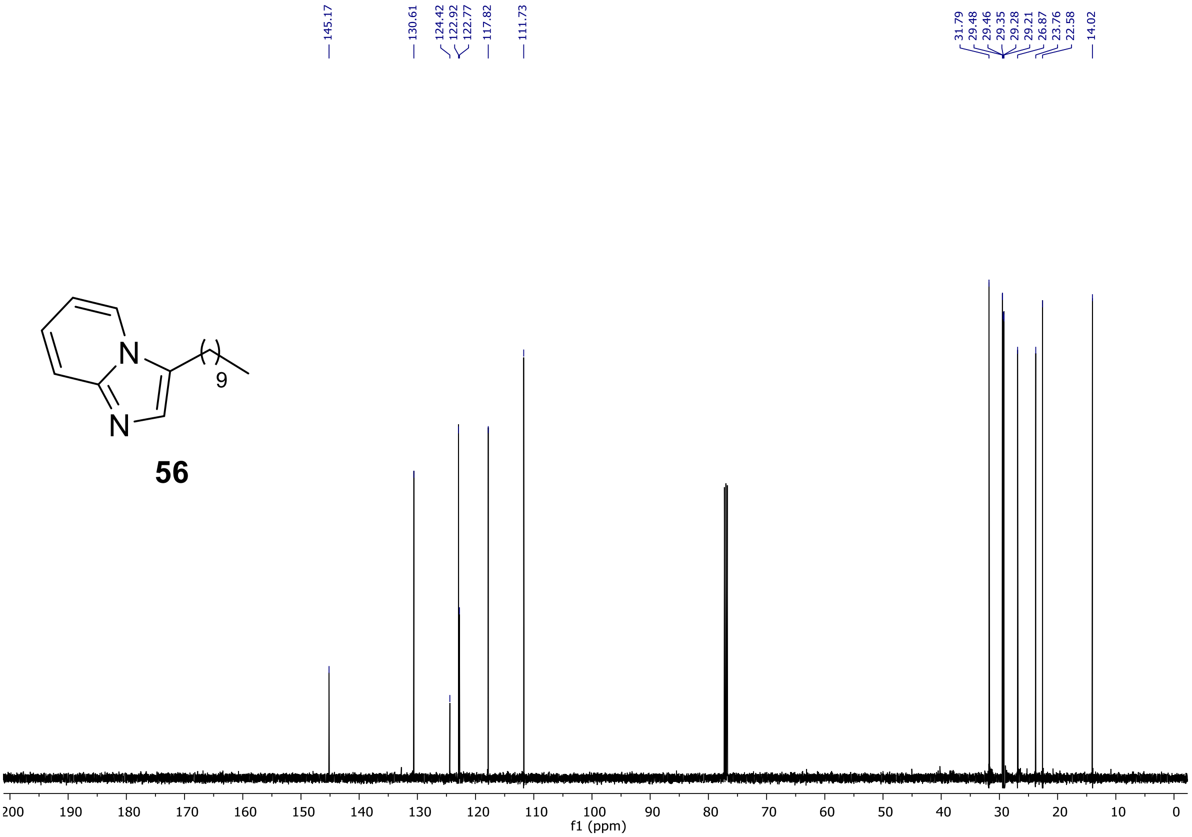

Figure S120. $^{13}C\{^{1}H\}$ Spectrum of **56** in $CDCl_3$ (125 MHz)

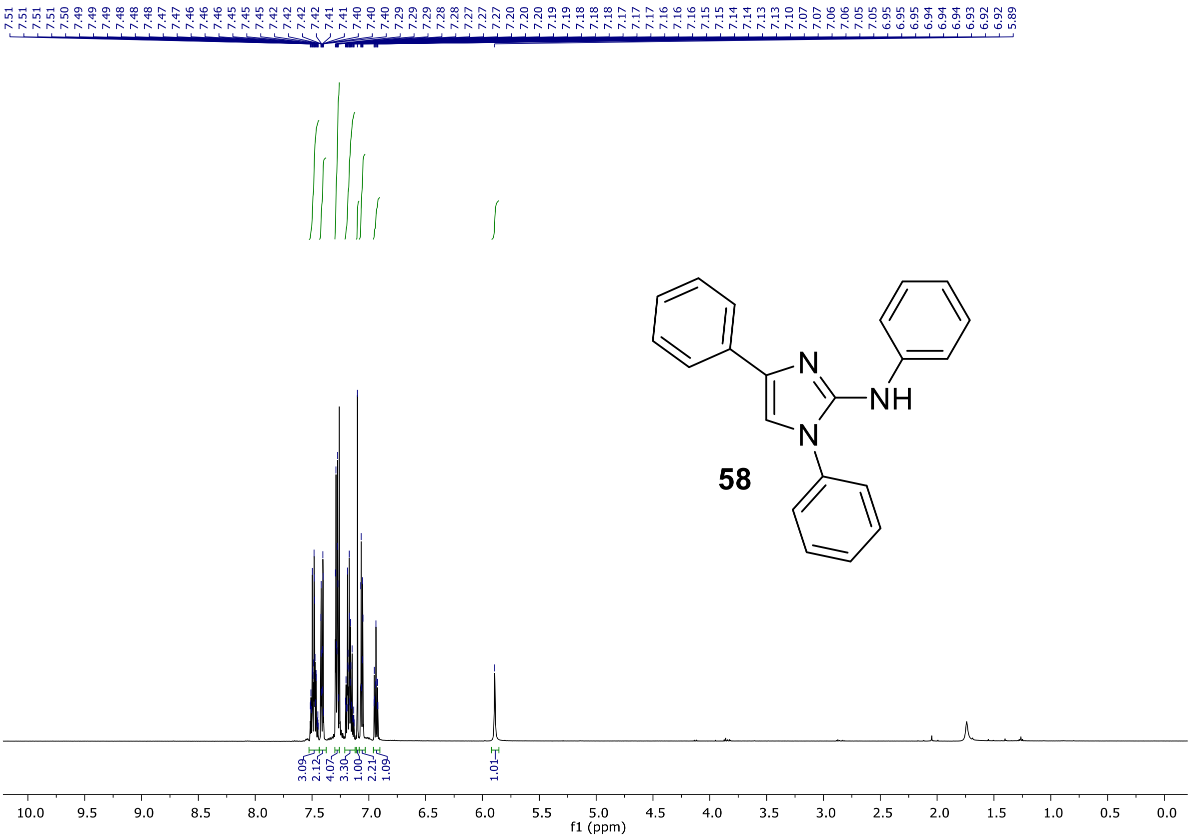

Figure S121. ^{1}H Spectrum of **58** in $CDCl_3$ (500 MHz)

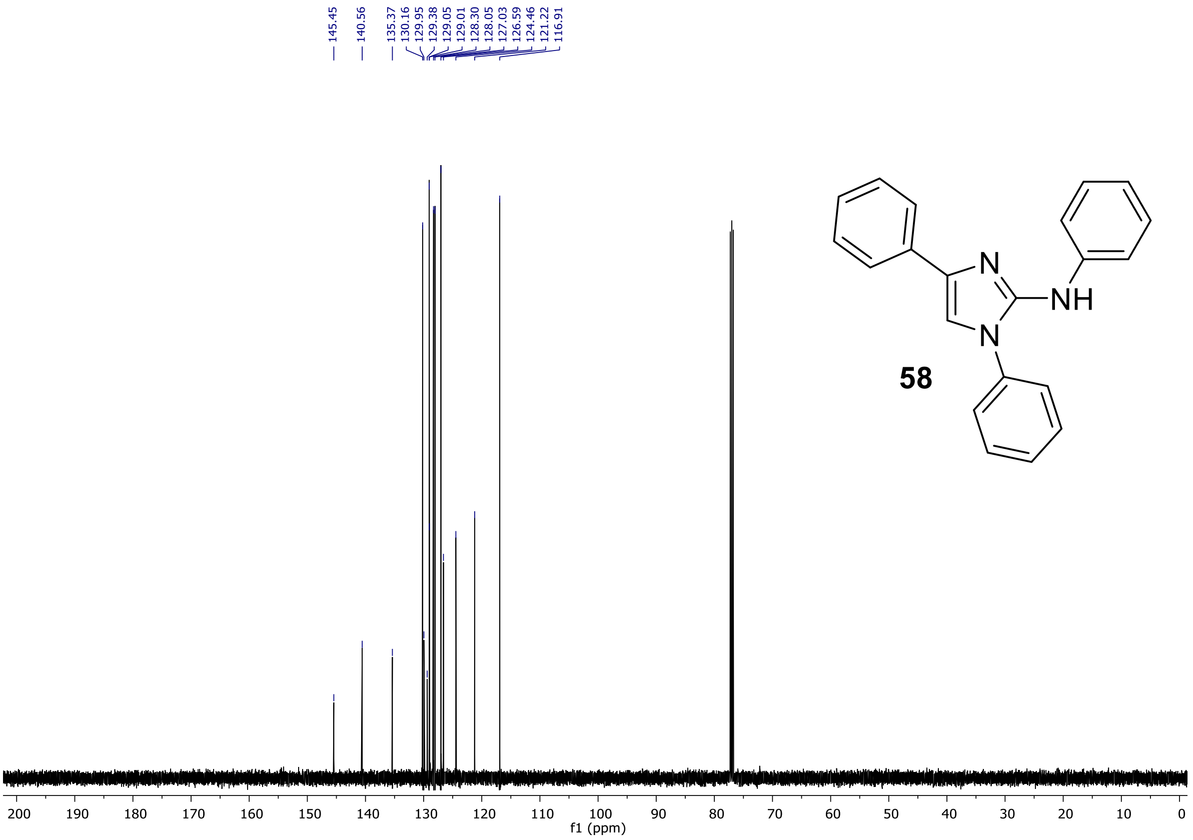

Figure S122. $^{13}C\{^{1}H\}$ Spectrum of **58** in $CDCl_3$ (125 MHz)

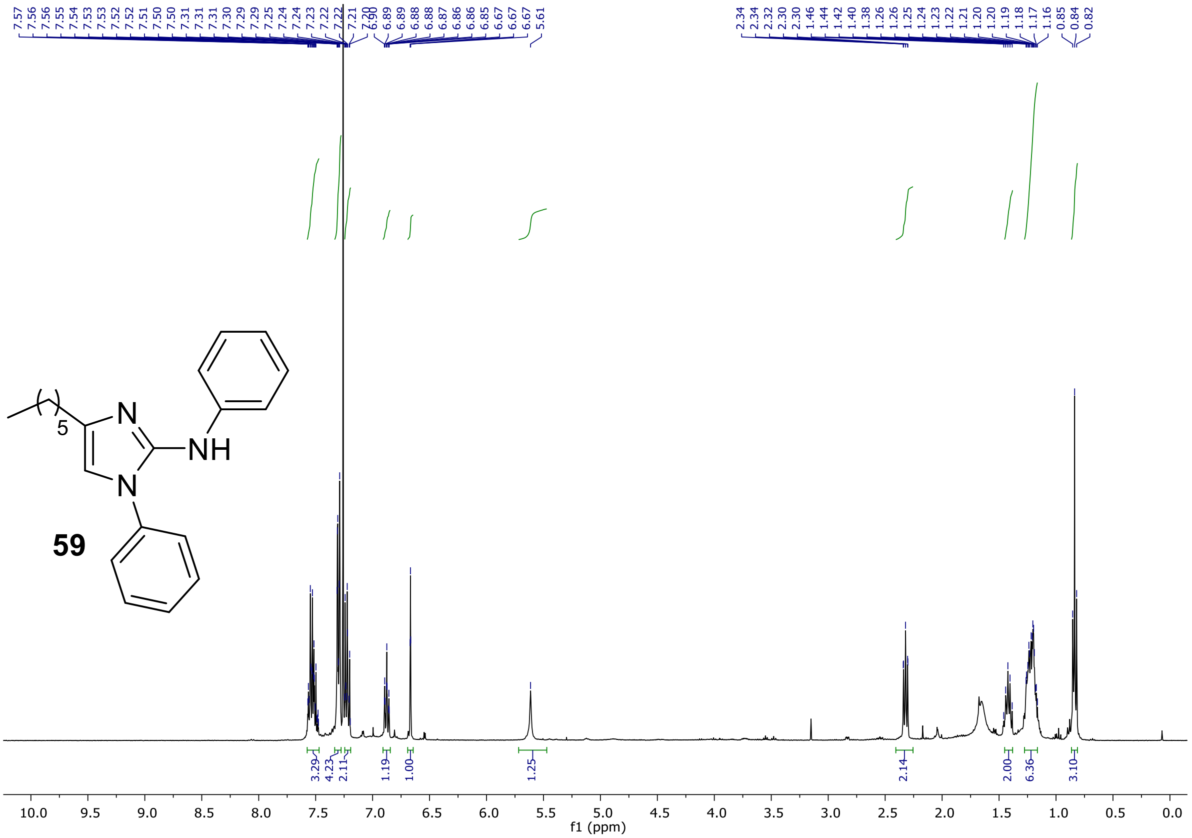

Figure S123. ^{1}H Spectrum of **59** in $CDCl_3$ (400 MHz)

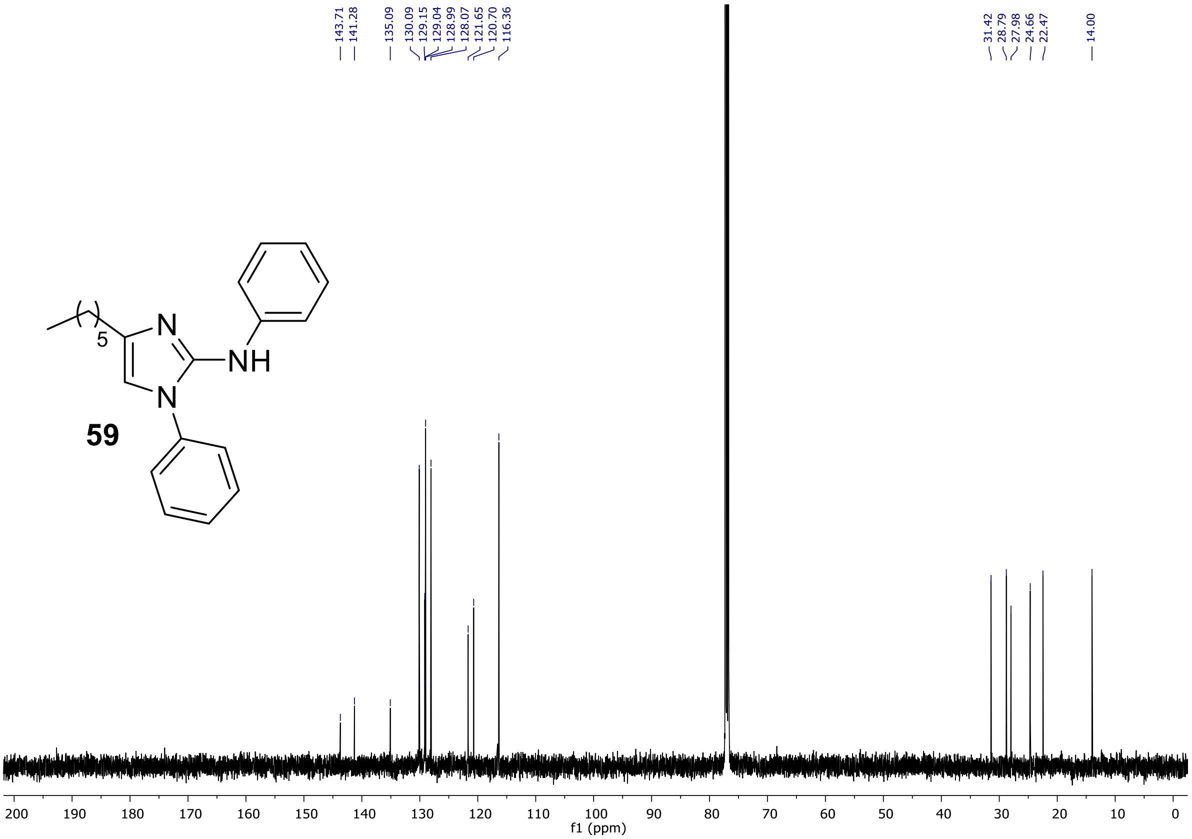

Figure S124. $^{13}C\{^{1}H\}$ Spectrum of **59** in $CDCl_3$ (125 MHz)

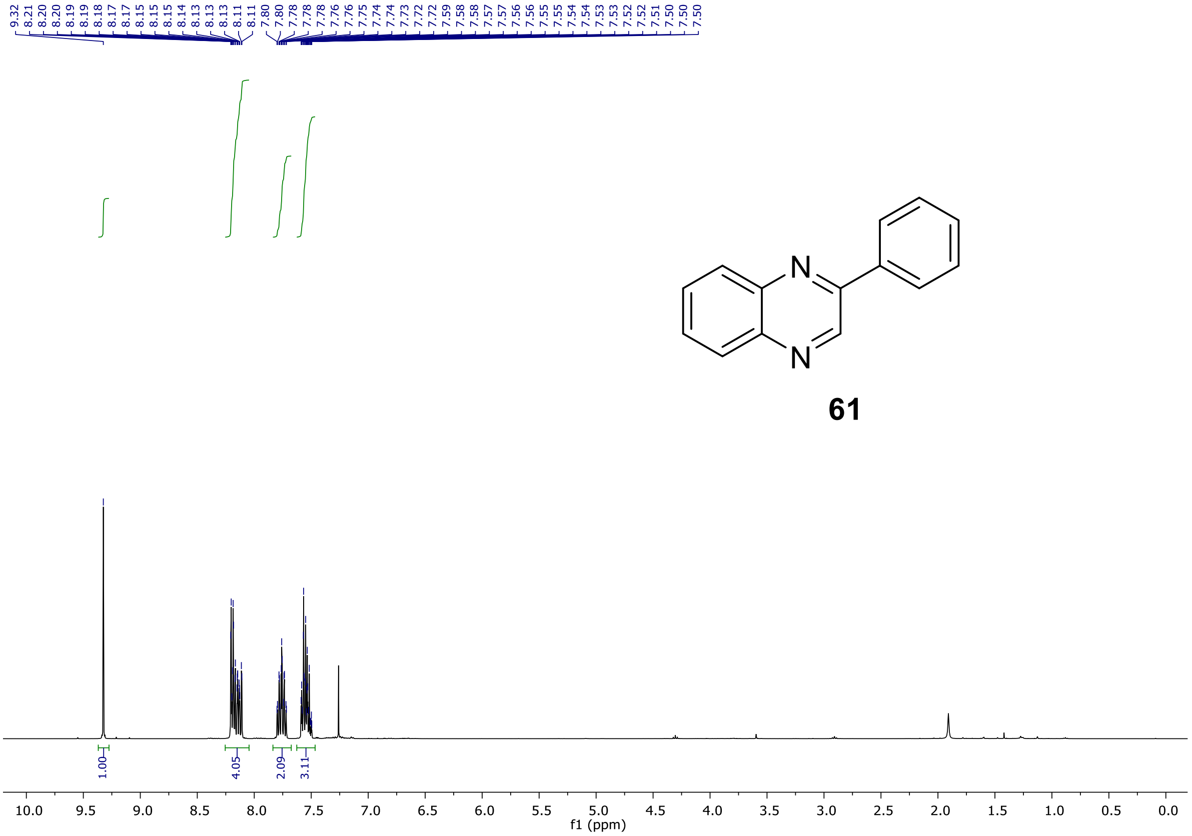

Figure S125. ^{1}H Spectrum of **61** in $CDCl_3$ (400 MHz)

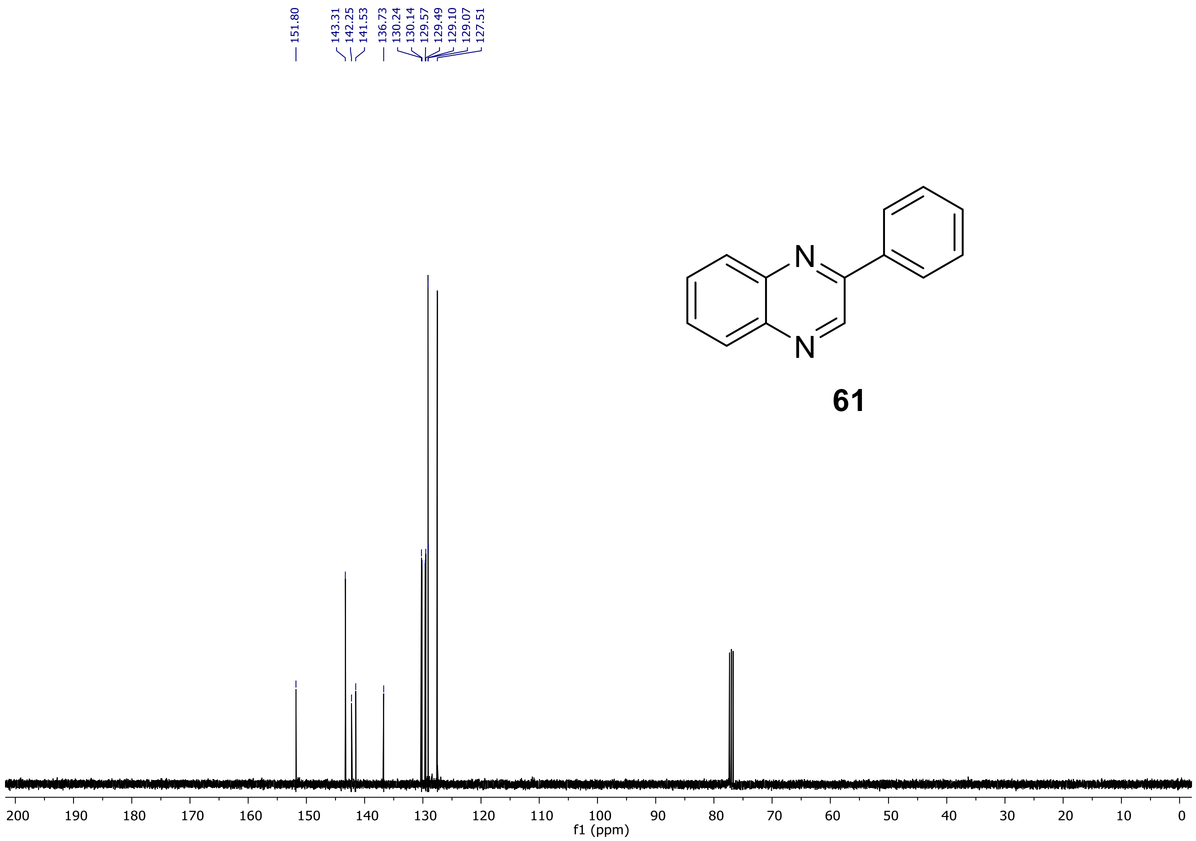

Figure S126. $^{13}C\{^{1}H\}$ Spectrum of **61** in $CDCl_3$ (100 MHz)

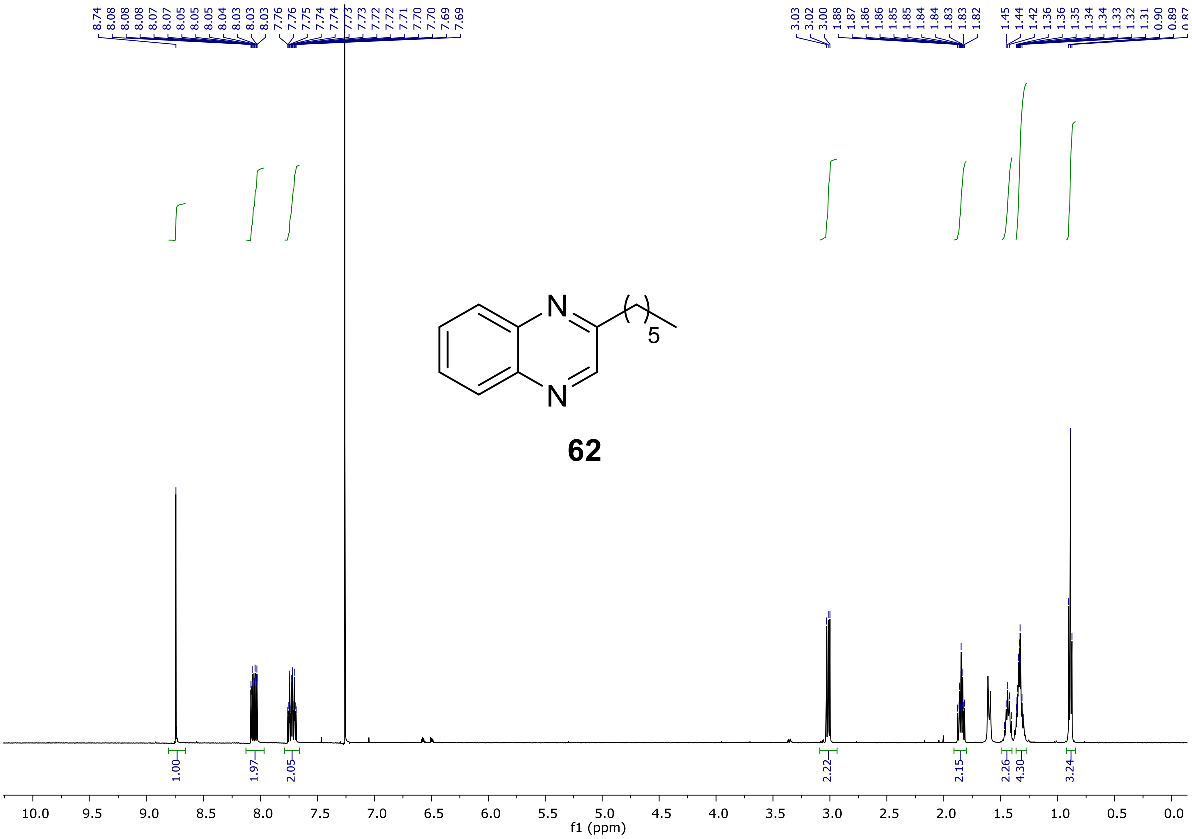

Figure S127. ^{1}H Spectrum of **62** in $CDCl_3$ (500 MHz)

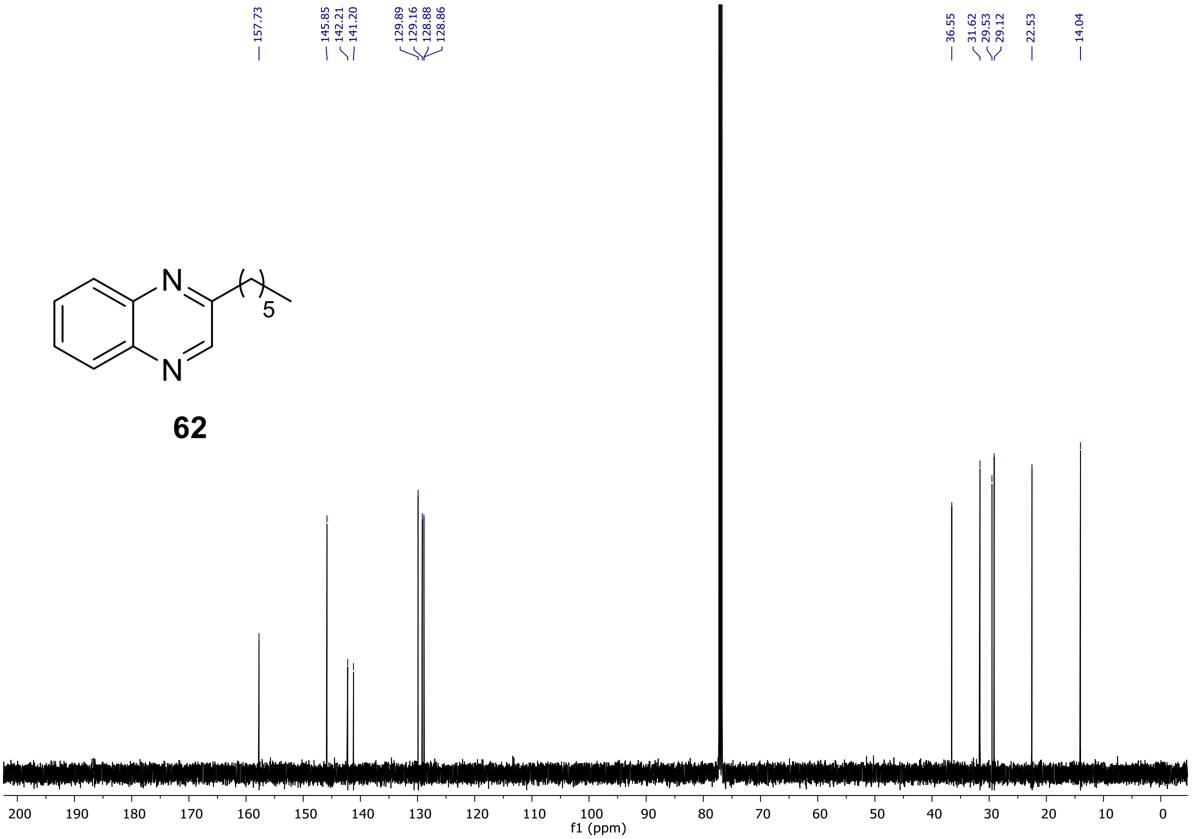

Figure S128. $^{13}C\{^{1}H\}$ Spectrum of **62** in $CDCl_3$ (125 MHz)

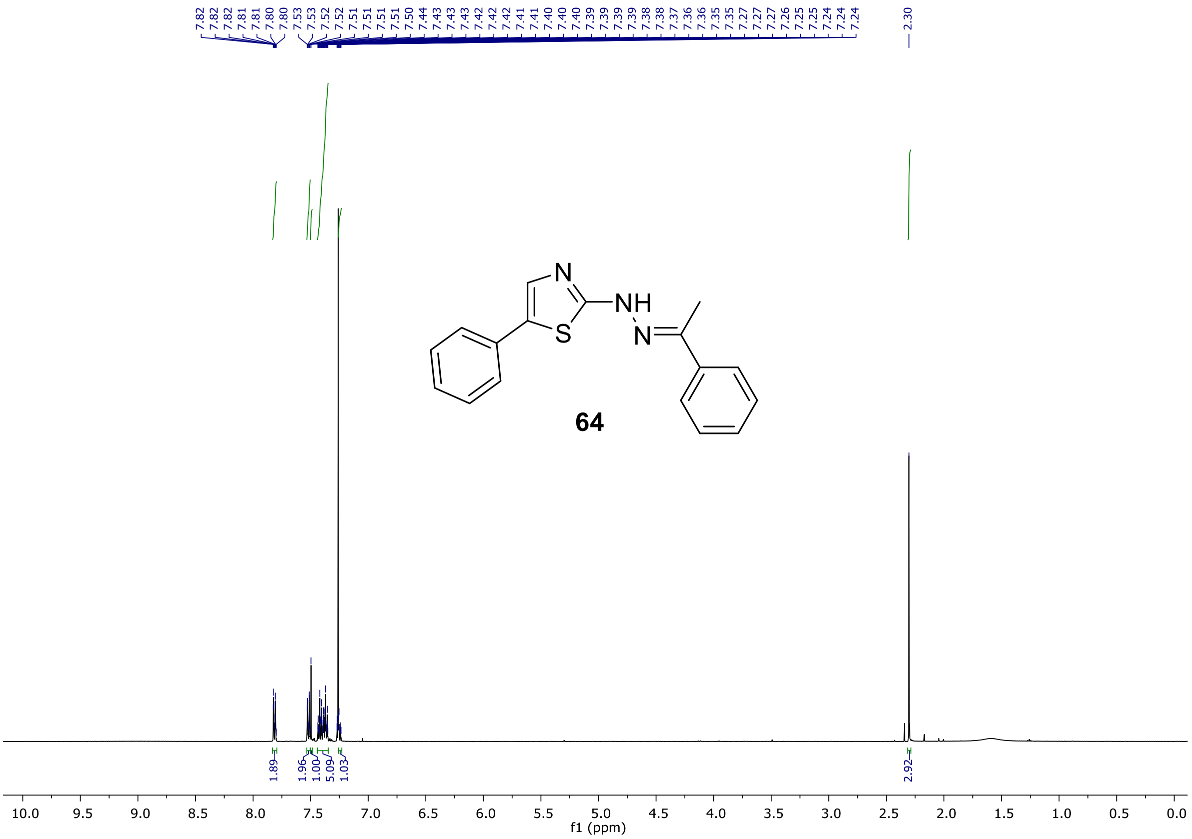

Figure S129. ^{1}H Spectrum of **64** in $CDCl_3$ (400 MHz)

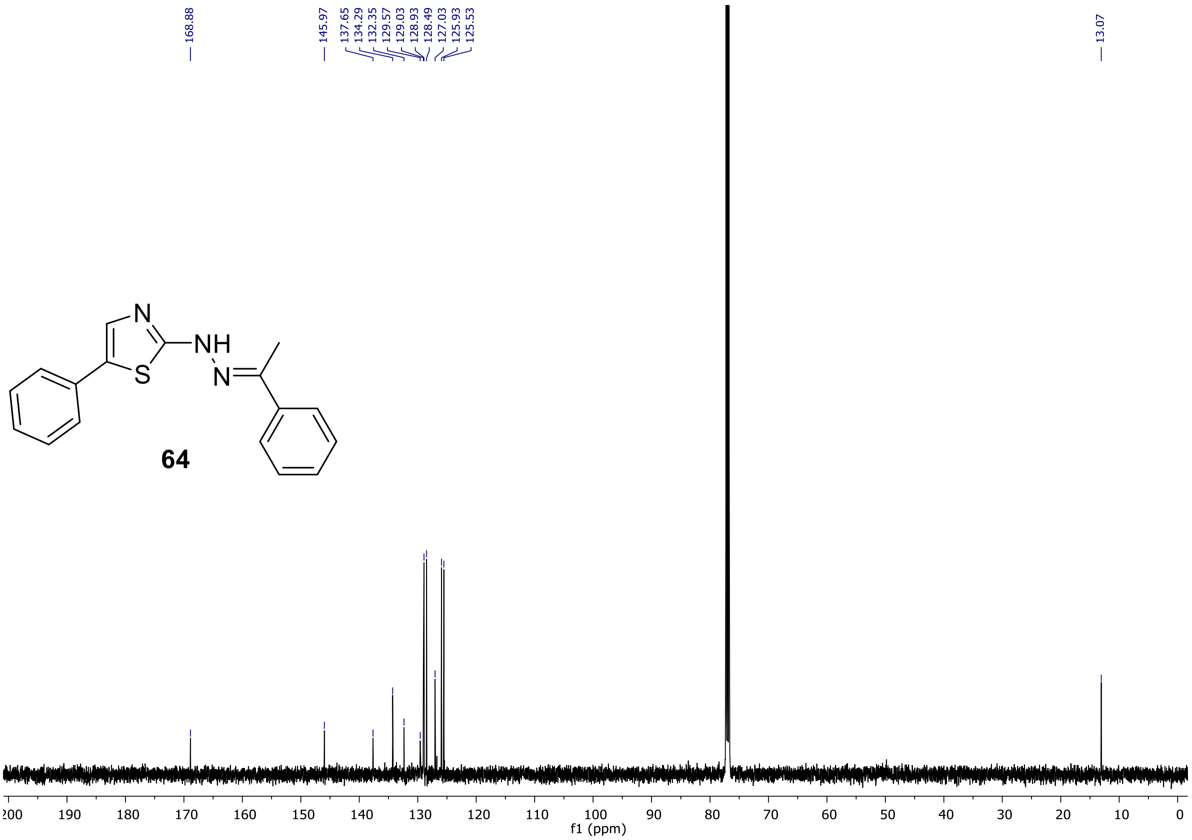

Figure S130. $^{13}C\{^{1}H\}$ Spectrum of **64** in $CDCl_3$ (125 MHz)

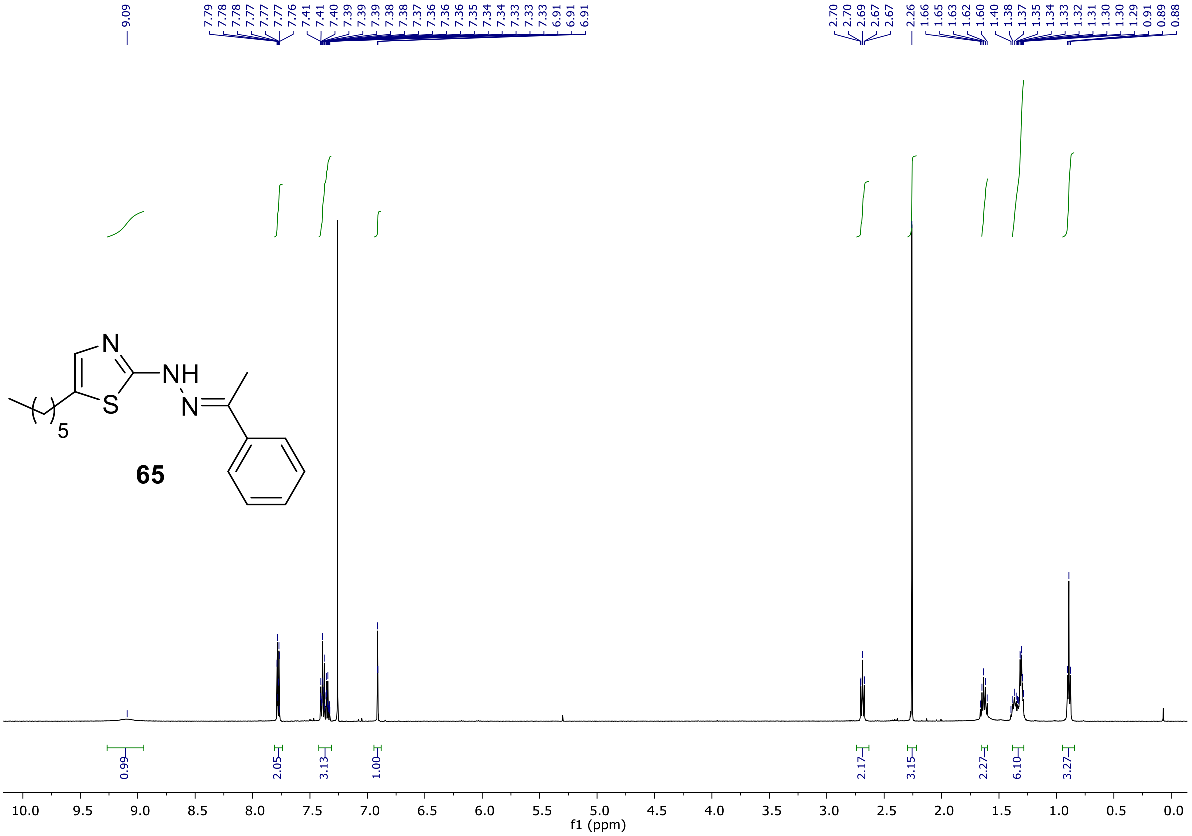

Figure S131. ^{1}H Spectrum of **65** in $CDCl_3$ (500 MHz)

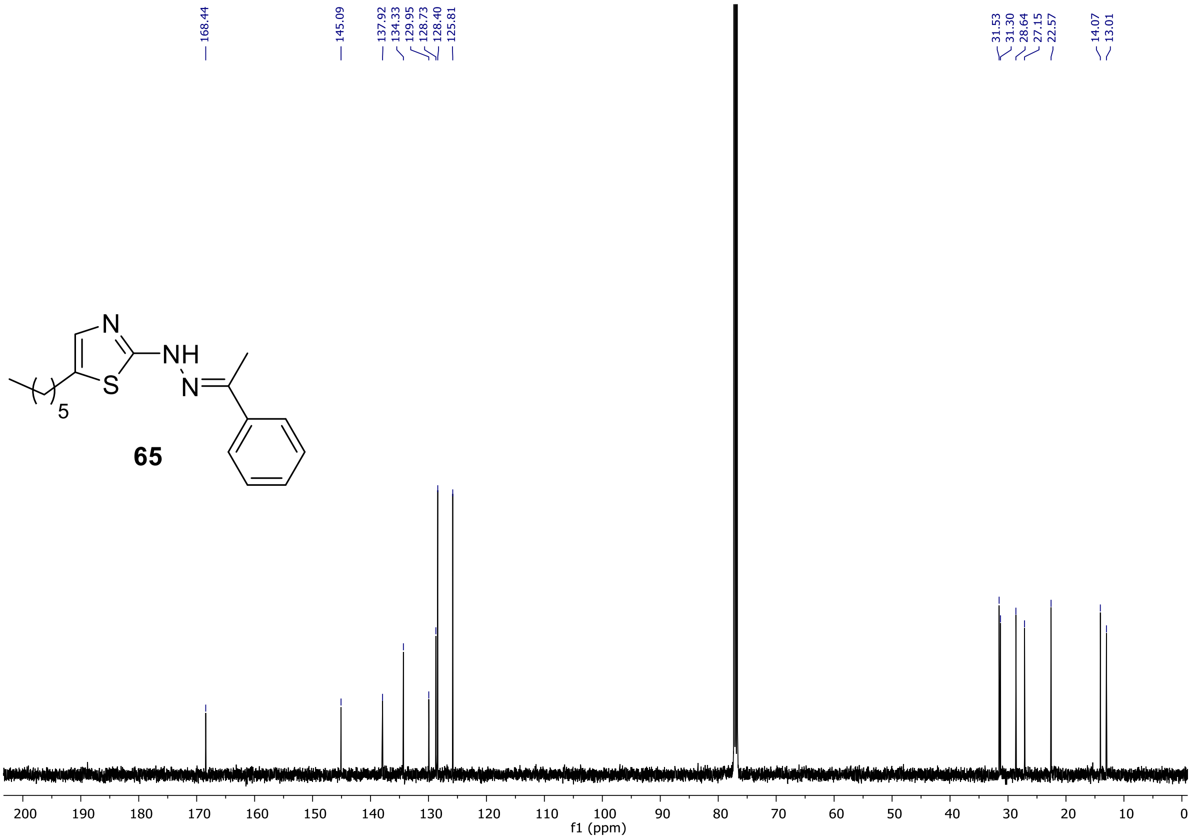

Figure S132. $^{13}C\{^{1}H\}$ Spectrum of **65** in $CDCl_3$ (125 MHz)
